# Supplementary material for: Role and mechanism of NCAPD3 in promoting malignant behaviors in gastric cancer
Source: Front Pharmacol. 2024 Apr 22;15:1341039. doi: 10.3389/fphar.2024.1341039 (PMC11070777; doi:10.3389/fphar.2024.1341039)
Supplement: Supplementary file 11 [file DataSheet2.ZIP › GSEA/immunologic/my_analysis.Gsea.1599462003817/gsea_report_for_NCAPD3_MUT_1599462003817.html]

Report for NCAPD3\_MUT 1599462003817 [GSEA]

| GS  follow link to MSigDB | GS DETAILS | SIZE | ES | NES | NOM p-val | FDR q-val | FWER p-val | RANK AT MAX | LEADING EDGE || 1 | GSE17974\_0H\_VS\_24H\_IN\_VITRO\_ACT\_CD4\_TCELL\_UP | Details ... | 24 | -0.64 | -3.16 | 0.000 | 0.000 | 0.000 | 502 | tags=96%, list=36%, signal=146% |
| 2 | GSE21063\_CTRL\_VS\_ANTI\_IGM\_STIM\_BCELL\_NFATC1\_KO\_8H\_UP | Details ... | 24 | -0.59 | -2.93 | 0.000 | 0.002 | 0.002 | 317 | tags=71%, list=22%, signal=90% |
| 3 | GSE17974\_CTRL\_VS\_ACT\_IL4\_AND\_ANTI\_IL12\_12H\_CD4\_TCELL\_UP | Details ... | 35 | -0.50 | -2.75 | 0.000 | 0.001 | 0.002 | 562 | tags=89%, list=40%, signal=144% |
| 4 | GSE1460\_INTRATHYMIC\_T\_PROGENITOR\_VS\_CD4\_THYMOCYTE\_DN | Details ... | 15 | -0.67 | -2.68 | 0.000 | 0.004 | 0.009 | 242 | tags=73%, list=17%, signal=88% |
| 5 | GSE369\_PRE\_VS\_POST\_IL6\_INJECTION\_SOCS3\_KO\_LIVER\_UP | Details ... | 33 | -0.49 | -2.66 | 0.000 | 0.003 | 0.011 | 547 | tags=85%, list=39%, signal=135% |
| 6 | GSE17974\_CTRL\_VS\_ACT\_IL4\_AND\_ANTI\_IL12\_6H\_CD4\_TCELL\_UP | Details ... | 25 | -0.54 | -2.65 | 0.000 | 0.003 | 0.013 | 555 | tags=92%, list=39%, signal=149% |
| 7 | GSE43955\_1H\_VS\_20H\_ACT\_CD4\_TCELL\_WITH\_TGFB\_IL6\_DN | Details ... | 23 | -0.55 | -2.64 | 0.000 | 0.004 | 0.018 | 476 | tags=87%, list=34%, signal=129% |
| 8 | GSE21360\_PRIMARY\_VS\_TERTIARY\_MEMORY\_CD8\_TCELL\_DN | Details ... | 30 | -0.50 | -2.64 | 0.000 | 0.004 | 0.018 | 541 | tags=87%, list=38%, signal=138% |
| 9 | GSE29618\_BCELL\_VS\_PDC\_UP | Details ... | 27 | -0.52 | -2.61 | 0.000 | 0.006 | 0.032 | 552 | tags=89%, list=39%, signal=143% |
| 10 | GSE24142\_ADULT\_VS\_FETAL\_DN2\_THYMOCYTE\_UP | Details ... | 18 | -0.58 | -2.56 | 0.000 | 0.011 | 0.064 | 485 | tags=89%, list=34%, signal=134% |
| 11 | GSE43863\_NAIVE\_VS\_MEMORY\_TFH\_CD4\_TCELL\_D150\_LCMV\_UP | Details ... | 17 | -0.58 | -2.53 | 0.000 | 0.014 | 0.090 | 414 | tags=88%, list=29%, signal=123% |
| 12 | GSE360\_L\_DONOVANI\_VS\_B\_MALAYI\_HIGH\_DOSE\_DC\_UP | Details ... | 24 | -0.50 | -2.52 | 0.000 | 0.014 | 0.096 | 560 | tags=88%, list=40%, signal=143% |
| 13 | GSE411\_UNSTIM\_VS\_100MIN\_IL6\_STIM\_SOCS3\_KO\_MACROPHAGE\_DN | Details ... | 19 | -0.55 | -2.50 | 0.000 | 0.015 | 0.116 | 643 | tags=100%, list=46%, signal=181% |
| 14 | GSE43863\_LY6C\_INT\_CXCR5POS\_VS\_LY6C\_LOW\_CXCR5NEG\_EFFECTOR\_CD4\_TCELL\_DN | Details ... | 18 | -0.57 | -2.49 | 0.000 | 0.014 | 0.117 | 562 | tags=94%, list=40%, signal=155% |
| 15 | GSE16450\_CTRL\_VS\_IFNA\_6H\_STIM\_IMMATURE\_NEURON\_CELL\_LINE\_DN | Details ... | 27 | -0.48 | -2.48 | 0.003 | 0.015 | 0.127 | 251 | tags=56%, list=18%, signal=66% |
| 16 | GSE29618\_PDC\_VS\_MDC\_DAY7\_FLU\_VACCINE\_DN | Details ... | 18 | -0.56 | -2.47 | 0.000 | 0.014 | 0.132 | 562 | tags=94%, list=40%, signal=155% |
| 17 | GSE26030\_UNSTIM\_VS\_RESTIM\_TH1\_DAY5\_POST\_POLARIZATION\_UP | Details ... | 18 | -0.56 | -2.46 | 0.000 | 0.014 | 0.138 | 436 | tags=89%, list=31%, signal=127% |
| 18 | GSE36888\_UNTREATED\_VS\_IL2\_TREATED\_STAT5\_AB\_KNOCKIN\_TCELL\_6H\_UP | Details ... | 17 | -0.55 | -2.46 | 0.000 | 0.013 | 0.140 | 279 | tags=65%, list=20%, signal=80% |
| 19 | GSE39820\_CTRL\_VS\_IL1B\_IL6\_IL23A\_CD4\_TCELL\_DN | Details ... | 24 | -0.49 | -2.45 | 0.000 | 0.015 | 0.167 | 308 | tags=67%, list=22%, signal=84% |
| 20 | GSE19888\_CTRL\_VS\_A3R\_INHIBITOR\_TREATED\_MAST\_CELL\_DN | Details ... | 42 | -0.41 | -2.44 | 0.000 | 0.016 | 0.180 | 225 | tags=45%, list=16%, signal=52% |
| 21 | GSE12366\_GC\_BCELL\_VS\_PLASMA\_CELL\_DN |  | 19 | -0.54 | -2.44 | 0.000 | 0.015 | 0.181 | 547 | tags=95%, list=39%, signal=153% |
| 22 | GSE12392\_IFNAR\_KO\_VS\_IFNB\_KO\_CD8\_NEG\_SPLEEN\_DC\_UP |  | 28 | -0.46 | -2.43 | 0.000 | 0.017 | 0.205 | 566 | tags=82%, list=40%, signal=134% |
| 23 | GSE16450\_IMMATURE\_VS\_MATURE\_NEURON\_CELL\_LINE\_UP |  | 28 | -0.47 | -2.42 | 0.000 | 0.017 | 0.210 | 414 | tags=71%, list=29%, signal=99% |
| 24 | GSE30971\_CTRL\_VS\_LPS\_STIM\_MACROPHAGE\_WBP7\_KO\_2H\_UP |  | 28 | -0.47 | -2.42 | 0.000 | 0.016 | 0.213 | 301 | tags=57%, list=21%, signal=71% |
| 25 | GSE43863\_DAY6\_EFF\_VS\_DAY150\_MEM\_LY6C\_INT\_CXCR5POS\_CD4\_TCELL\_UP |  | 21 | -0.51 | -2.41 | 0.000 | 0.016 | 0.224 | 556 | tags=90%, list=39%, signal=147% |
| 26 | GSE13547\_WT\_VS\_ZFX\_KO\_BCELL\_UP |  | 22 | -0.51 | -2.40 | 0.000 | 0.018 | 0.256 | 526 | tags=86%, list=37%, signal=136% |
| 27 | GSE7219\_UNSTIM\_VS\_LPS\_AND\_ANTI\_CD40\_STIM\_DC\_DN |  | 20 | -0.52 | -2.37 | 0.000 | 0.020 | 0.294 | 191 | tags=45%, list=14%, signal=51% |
| 28 | GSE17974\_0H\_VS\_12H\_IN\_VITRO\_ACT\_CD4\_TCELL\_UP |  | 33 | -0.43 | -2.37 | 0.000 | 0.020 | 0.295 | 555 | tags=82%, list=39%, signal=132% |
| 29 | GSE39820\_CTRL\_VS\_TGFBETA3\_IL6\_CD4\_TCELL\_DN |  | 25 | -0.49 | -2.37 | 0.000 | 0.020 | 0.311 | 482 | tags=80%, list=34%, signal=119% |
| 30 | GSE7764\_IL15\_TREATED\_VS\_CTRL\_NK\_CELL\_24H\_DN |  | 21 | -0.51 | -2.36 | 0.000 | 0.020 | 0.320 | 314 | tags=62%, list=22%, signal=78% |
| 31 | GSE29618\_BCELL\_VS\_MDC\_UP |  | 23 | -0.50 | -2.36 | 0.000 | 0.020 | 0.320 | 429 | tags=74%, list=30%, signal=104% |
| 32 | GSE30971\_CTRL\_VS\_LPS\_STIM\_MACROPHAGE\_WBP7\_KO\_4H\_UP |  | 30 | -0.44 | -2.35 | 0.000 | 0.021 | 0.347 | 572 | tags=87%, list=41%, signal=143% |
| 33 | GSE360\_DC\_VS\_MAC\_L\_DONOVANI\_UP |  | 22 | -0.48 | -2.33 | 0.000 | 0.024 | 0.395 | 417 | tags=64%, list=30%, signal=89% |
| 34 | GSE30971\_CTRL\_VS\_LPS\_STIM\_MACROPHAGE\_WBP7\_HET\_2H\_UP |  | 33 | -0.42 | -2.33 | 0.000 | 0.024 | 0.399 | 397 | tags=61%, list=28%, signal=82% |
| 35 | GSE9650\_NAIVE\_VS\_MEMORY\_CD8\_TCELL\_DN |  | 22 | -0.50 | -2.33 | 0.000 | 0.023 | 0.399 | 591 | tags=91%, list=42%, signal=154% |
| 36 | GSE26030\_UNSTIM\_VS\_RESTIM\_TH1\_DAY5\_POST\_POLARIZATION\_DN |  | 20 | -0.51 | -2.32 | 0.000 | 0.023 | 0.406 | 422 | tags=70%, list=30%, signal=98% |
| 37 | GSE25677\_MPL\_VS\_R848\_STIM\_BCELL\_UP |  | 22 | -0.49 | -2.32 | 0.000 | 0.023 | 0.416 | 253 | tags=59%, list=18%, signal=71% |
| 38 | GSE17974\_CTRL\_VS\_ACT\_IL4\_AND\_ANTI\_IL12\_24H\_CD4\_TCELL\_UP |  | 30 | -0.44 | -2.31 | 0.000 | 0.024 | 0.434 | 302 | tags=53%, list=21%, signal=66% |
| 39 | GSE29949\_CD8\_NEG\_DC\_SPLEEN\_VS\_MONOCYTE\_BONE\_MARROW\_DN |  | 18 | -0.54 | -2.31 | 0.000 | 0.023 | 0.436 | 509 | tags=89%, list=36%, signal=137% |
| 40 | GSE22601\_IMMATURE\_CD4\_SINGLE\_POSITIVE\_VS\_CD8\_SINGLE\_POSITIVE\_THYMOCYTE\_DN |  | 29 | -0.44 | -2.31 | 0.000 | 0.024 | 0.450 | 395 | tags=69%, list=28%, signal=94% |
| 41 | GSE36476\_CTRL\_VS\_TSST\_ACT\_40H\_MEMORY\_CD4\_TCELL\_OLD\_UP |  | 16 | -0.54 | -2.30 | 0.000 | 0.025 | 0.467 | 555 | tags=94%, list=39%, signal=153% |
| 42 | GSE3920\_UNTREATED\_VS\_IFNA\_TREATED\_FIBROBLAST\_UP |  | 31 | -0.42 | -2.30 | 0.000 | 0.024 | 0.467 | 549 | tags=77%, list=39%, signal=124% |
| 43 | GSE37416\_0H\_VS\_12H\_F\_TULARENSIS\_LVS\_NEUTROPHIL\_DN |  | 27 | -0.44 | -2.28 | 0.000 | 0.026 | 0.508 | 608 | tags=89%, list=43%, signal=153% |
| 44 | GSE21774\_CD56\_BRIGHT\_VS\_DIM\_CD62L\_POSITIVE\_NK\_CELL\_UP |  | 23 | -0.46 | -2.28 | 0.000 | 0.027 | 0.525 | 249 | tags=52%, list=18%, signal=62% |
| 45 | GSE9988\_ANTI\_TREM1\_VS\_CTRL\_TREATED\_MONOCYTES\_UP |  | 41 | -0.38 | -2.28 | 0.003 | 0.027 | 0.525 | 550 | tags=78%, list=39%, signal=124% |
| 46 | GSE3982\_MAC\_VS\_TH2\_UP |  | 22 | -0.48 | -2.27 | 0.000 | 0.028 | 0.547 | 352 | tags=73%, list=25%, signal=95% |
| 47 | GSE360\_CTRL\_VS\_L\_MAJOR\_DC\_DN |  | 19 | -0.50 | -2.27 | 0.000 | 0.028 | 0.556 | 572 | tags=89%, list=41%, signal=148% |
| 48 | GSE21670\_STAT3\_KO\_VS\_WT\_CD4\_TCELL\_TGFB\_IL6\_TREATED\_DN |  | 26 | -0.45 | -2.26 | 0.000 | 0.028 | 0.574 | 408 | tags=65%, list=29%, signal=90% |
| 49 | GSE43955\_10H\_VS\_60H\_ACT\_CD4\_TCELL\_DN |  | 23 | -0.46 | -2.26 | 0.000 | 0.028 | 0.582 | 563 | tags=87%, list=40%, signal=142% |
| 50 | GSE7852\_THYMUS\_VS\_FAT\_TCONV\_DN |  | 31 | -0.42 | -2.26 | 0.000 | 0.028 | 0.588 | 544 | tags=81%, list=39%, signal=128% |
| 51 | GSE22886\_DAY1\_VS\_DAY7\_MONOCYTE\_IN\_CULTURE\_UP |  | 24 | -0.46 | -2.25 | 0.000 | 0.028 | 0.603 | 393 | tags=67%, list=28%, signal=91% |
| 52 | GSE5589\_LPS\_VS\_LPS\_AND\_IL10\_STIM\_IL10\_KO\_MACROPHAGE\_45MIN\_DN |  | 16 | -0.53 | -2.24 | 0.000 | 0.030 | 0.631 | 263 | tags=63%, list=19%, signal=76% |
| 53 | GSE43863\_TH1\_VS\_TFH\_MEMORY\_CD4\_TCELL\_DN |  | 15 | -0.56 | -2.23 | 0.000 | 0.033 | 0.675 | 225 | tags=60%, list=16%, signal=71% |
| 54 | GSE24671\_CTRL\_VS\_SENDAI\_VIRUS\_INFECTED\_MOUSE\_SPLENOCYTES\_UP |  | 15 | -0.54 | -2.23 | 0.000 | 0.033 | 0.683 | 451 | tags=87%, list=32%, signal=126% |
| 55 | GSE15733\_BM\_VS\_SPLEEN\_MEMORY\_CD4\_TCELL\_UP |  | 17 | -0.52 | -2.21 | 0.000 | 0.036 | 0.720 | 245 | tags=53%, list=17%, signal=63% |
| 56 | GSE7852\_TREG\_VS\_TCONV\_THYMUS\_UP |  | 29 | -0.42 | -2.21 | 0.000 | 0.036 | 0.720 | 572 | tags=83%, list=41%, signal=136% |
| 57 | GSE3720\_UNSTIM\_VS\_LPS\_STIM\_VD1\_GAMMADELTA\_TCELL\_DN |  | 15 | -0.54 | -2.21 | 0.002 | 0.035 | 0.726 | 547 | tags=93%, list=39%, signal=151% |
| 58 | GSE32423\_CTRL\_VS\_IL4\_MEMORY\_CD8\_TCELL\_UP |  | 19 | -0.48 | -2.20 | 0.005 | 0.038 | 0.752 | 376 | tags=68%, list=27%, signal=92% |
| 59 | GSE3565\_CTRL\_VS\_LPS\_INJECTED\_DUSP1\_KO\_SPLENOCYTES\_UP |  | 23 | -0.45 | -2.20 | 0.000 | 0.037 | 0.754 | 563 | tags=83%, list=40%, signal=135% |
| 60 | GSE21360\_NAIVE\_VS\_SECONDARY\_MEMORY\_CD8\_TCELL\_UP |  | 19 | -0.49 | -2.19 | 0.000 | 0.038 | 0.763 | 550 | tags=84%, list=39%, signal=136% |
| 61 | GSE7852\_TREG\_VS\_TCONV\_FAT\_UP |  | 17 | -0.50 | -2.19 | 0.000 | 0.038 | 0.763 | 711 | tags=100%, list=50%, signal=199% |
| 62 | GSE6269\_E\_COLI\_VS\_STREP\_PNEUMO\_INF\_PBMC\_DN |  | 17 | -0.50 | -2.19 | 0.000 | 0.037 | 0.765 | 468 | tags=82%, list=33%, signal=122% |
| 63 | GSE2770\_UNTREATED\_VS\_IL12\_TREATED\_ACT\_CD4\_TCELL\_6H\_UP |  | 35 | -0.39 | -2.19 | 0.000 | 0.037 | 0.766 | 556 | tags=77%, list=39%, signal=124% |
| 64 | GSE23568\_ID3\_KO\_VS\_WT\_CD8\_TCELL\_DN |  | 19 | -0.48 | -2.18 | 0.000 | 0.040 | 0.801 | 104 | tags=37%, list=7%, signal=39% |
| 65 | GSE43955\_TH0\_VS\_TGFB\_IL6\_TH17\_ACT\_CD4\_TCELL\_30H\_UP |  | 25 | -0.44 | -2.18 | 0.002 | 0.039 | 0.804 | 662 | tags=92%, list=47%, signal=170% |
| 66 | GSE1432\_1H\_VS\_24H\_IFNG\_MICROGLIA\_UP |  | 17 | -0.50 | -2.18 | 0.000 | 0.039 | 0.807 | 508 | tags=88%, list=36%, signal=136% |
| 67 | GSE17974\_0H\_VS\_6H\_IN\_VITRO\_ACT\_CD4\_TCELL\_UP |  | 25 | -0.44 | -2.17 | 0.000 | 0.040 | 0.822 | 549 | tags=80%, list=39%, signal=129% |
| 68 | GSE1460\_INTRATHYMIC\_T\_PROGENITOR\_VS\_NAIVE\_CD4\_TCELL\_ADULT\_BLOOD\_DN |  | 23 | -0.44 | -2.17 | 0.000 | 0.040 | 0.827 | 348 | tags=61%, list=25%, signal=79% |
| 69 | GSE29617\_CTRL\_VS\_TIV\_FLU\_VACCINE\_PBMC\_2008\_UP |  | 22 | -0.45 | -2.16 | 0.000 | 0.042 | 0.850 | 523 | tags=82%, list=37%, signal=128% |
| 70 | GSE7764\_IL15\_NK\_CELL\_24H\_VS\_SPLENOCYTE\_DN |  | 28 | -0.42 | -2.16 | 0.000 | 0.042 | 0.854 | 712 | tags=93%, list=50%, signal=184% |
| 71 | GSE24210\_RESTING\_TREG\_VS\_TCONV\_UP |  | 22 | -0.46 | -2.16 | 0.002 | 0.042 | 0.857 | 525 | tags=82%, list=37%, signal=128% |
| 72 | GSE25123\_WT\_VS\_PPARG\_KO\_MACROPHAGE\_UP |  | 28 | -0.43 | -2.16 | 0.000 | 0.043 | 0.860 | 297 | tags=57%, list=21%, signal=71% |
| 73 | GSE360\_CTRL\_VS\_L\_DONOVANI\_DC\_DN |  | 23 | -0.45 | -2.15 | 0.002 | 0.043 | 0.869 | 560 | tags=83%, list=40%, signal=135% |
| 74 | GSE42088\_UNINF\_VS\_LEISHMANIA\_INF\_DC\_2H\_UP |  | 28 | -0.42 | -2.15 | 0.002 | 0.044 | 0.878 | 254 | tags=46%, list=18%, signal=55% |
| 75 | GSE22432\_PDC\_VS\_TGFB1\_TREATEDCOMMON\_DC\_PROGENITOR\_DN |  | 19 | -0.47 | -2.15 | 0.000 | 0.044 | 0.878 | 308 | tags=53%, list=22%, signal=66% |
| 76 | GSE6259\_CD4\_TCELL\_VS\_CD8\_TCELL\_DN |  | 28 | -0.40 | -2.14 | 0.000 | 0.045 | 0.886 | 273 | tags=46%, list=19%, signal=56% |
| 77 | GSE37416\_0H\_VS\_24H\_F\_TULARENSIS\_LVS\_NEUTROPHIL\_DN |  | 24 | -0.44 | -2.14 | 0.000 | 0.045 | 0.891 | 539 | tags=83%, list=38%, signal=133% |
| 78 | GSE46606\_DAY1\_VS\_DAY3\_CD40L\_IL2\_IL5\_STIMULATED\_IRF4MID\_BCELL\_DN |  | 16 | -0.50 | -2.14 | 0.007 | 0.046 | 0.895 | 315 | tags=63%, list=22%, signal=80% |
| 79 | GSE27241\_CTRL\_VS\_DIGOXIN\_TREATED\_CD4\_TCELL\_IN\_TH17\_POLARIZING\_CONDITIONS\_UP |  | 19 | -0.46 | -2.14 | 0.002 | 0.046 | 0.897 | 385 | tags=63%, list=27%, signal=86% |
| 80 | GSE30962\_PRIMARY\_VS\_SECONDARY\_ACUTE\_LCMV\_INF\_CD8\_TCELL\_DN |  | 27 | -0.42 | -2.14 | 0.000 | 0.045 | 0.897 | 281 | tags=52%, list=20%, signal=64% |
| 81 | GSE43863\_TFH\_VS\_LY6C\_LOW\_CXCR5NEG\_EFFECTOR\_CD4\_TCELL\_DN |  | 20 | -0.48 | -2.14 | 0.002 | 0.045 | 0.897 | 190 | tags=45%, list=13%, signal=51% |
| 82 | GSE28449\_WT\_VS\_LRF\_KO\_GERMINAL\_CENTER\_BCELL\_UP |  | 17 | -0.49 | -2.13 | 0.000 | 0.045 | 0.900 | 500 | tags=82%, list=35%, signal=126% |
| 83 | GSE45365\_NK\_CELL\_VS\_CD8\_TCELL\_MCMV\_INFECTION\_DN |  | 15 | -0.52 | -2.13 | 0.009 | 0.044 | 0.900 | 572 | tags=93%, list=41%, signal=155% |
| 84 | GSE2585\_CTEC\_VS\_MTEC\_THYMUS\_DN |  | 18 | -0.49 | -2.13 | 0.002 | 0.044 | 0.901 | 565 | tags=89%, list=40%, signal=146% |
| 85 | GSE39916\_B\_CELL\_SPLEEN\_VS\_PLASMA\_CELL\_BONE\_MARROW\_UP |  | 22 | -0.46 | -2.13 | 0.000 | 0.044 | 0.901 | 371 | tags=64%, list=26%, signal=85% |
| 86 | GSE11057\_NAIVE\_VS\_CENT\_MEMORY\_CD4\_TCELL\_DN |  | 29 | -0.40 | -2.13 | 0.000 | 0.044 | 0.901 | 317 | tags=52%, list=22%, signal=65% |
| 87 | GSE29164\_UNTREATED\_VS\_CD8\_TCELL\_TREATED\_MELANOMA\_DAY3\_UP |  | 23 | -0.45 | -2.13 | 0.002 | 0.043 | 0.902 | 321 | tags=61%, list=23%, signal=78% |
| 88 | GSE40273\_EOS\_KO\_VS\_WT\_TREG\_UP |  | 20 | -0.46 | -2.13 | 0.000 | 0.043 | 0.903 | 396 | tags=60%, list=28%, signal=82% |
| 89 | GSE7219\_WT\_VS\_NIK\_NFKB2\_KO\_LPS\_AND\_ANTI\_CD40\_STIM\_DC\_UP |  | 24 | -0.44 | -2.12 | 0.005 | 0.044 | 0.913 | 114 | tags=38%, list=8%, signal=40% |
| 90 | GSE22443\_NAIVE\_VS\_ACT\_AND\_IL2\_TREATED\_CD8\_TCELL\_UP |  | 21 | -0.46 | -2.12 | 0.000 | 0.044 | 0.913 | 356 | tags=62%, list=25%, signal=82% |
| 91 | GSE13738\_TCR\_VS\_BYSTANDER\_ACTIVATED\_CD4\_TCELL\_DN |  | 25 | -0.43 | -2.12 | 0.000 | 0.044 | 0.922 | 250 | tags=52%, list=18%, signal=62% |
| 92 | GSE11057\_NAIVE\_VS\_EFF\_MEMORY\_CD4\_TCELL\_DN |  | 24 | -0.43 | -2.12 | 0.003 | 0.044 | 0.923 | 268 | tags=46%, list=19%, signal=56% |
| 93 | GSE14769\_UNSTIM\_VS\_60MIN\_LPS\_BMDM\_DN |  | 38 | -0.37 | -2.12 | 0.002 | 0.045 | 0.924 | 563 | tags=82%, list=40%, signal=132% |
| 94 | GSE11057\_EFF\_MEM\_VS\_CENT\_MEM\_CD4\_TCELL\_UP |  | 20 | -0.47 | -2.12 | 0.002 | 0.044 | 0.924 | 495 | tags=80%, list=35%, signal=121% |
| 95 | GSE8685\_IL15\_ACT\_IL2\_STARVED\_VS\_IL21\_ACT\_IL2\_STARVED\_CD4\_TCELL\_UP |  | 21 | -0.45 | -2.11 | 0.000 | 0.044 | 0.927 | 327 | tags=62%, list=23%, signal=79% |
| 96 | GSE22935\_UNSTIM\_VS\_24H\_MBOVIS\_BCG\_STIM\_MYD88\_KO\_MACROPHAGE\_UP |  | 28 | -0.40 | -2.11 | 0.000 | 0.044 | 0.927 | 398 | tags=61%, list=28%, signal=83% |
| 97 | GSE28726\_NAIVE\_CD4\_TCELL\_VS\_NAIVE\_VA24NEG\_NKTCELL\_DN |  | 18 | -0.47 | -2.11 | 0.002 | 0.044 | 0.931 | 312 | tags=61%, list=22%, signal=77% |
| 98 | GSE22432\_CDC\_VS\_COMMON\_DC\_PROGENITOR\_UP |  | 18 | -0.49 | -2.11 | 0.000 | 0.044 | 0.932 | 473 | tags=78%, list=34%, signal=116% |
| 99 | GSE2405\_HEAT\_KILLED\_LYSATE\_VS\_LIVE\_A\_PHAGOCYTOPHILUM\_STIM\_NEUTROPHIL\_24H\_UP |  | 21 | -0.46 | -2.11 | 0.000 | 0.044 | 0.933 | 513 | tags=81%, list=36%, signal=125% |
| 100 | GSE20366\_TREG\_VS\_NAIVE\_CD4\_TCELL\_UP |  | 21 | -0.45 | -2.11 | 0.003 | 0.044 | 0.934 | 662 | tags=90%, list=47%, signal=168% |
| 101 | GSE22886\_CD8\_VS\_CD4\_NAIVE\_TCELL\_DN |  | 17 | -0.50 | -2.11 | 0.002 | 0.044 | 0.937 | 348 | tags=65%, list=25%, signal=85% |
| 102 | GSE35825\_UNTREATED\_VS\_IFNG\_STIM\_MACROPHAGE\_UP |  | 31 | -0.39 | -2.11 | 0.000 | 0.044 | 0.939 | 496 | tags=71%, list=35%, signal=107% |
| 103 | GSE41176\_UNSTIM\_VS\_ANTI\_IGM\_STIM\_TAK1\_KO\_BCELL\_24H\_DN |  | 25 | -0.42 | -2.10 | 0.007 | 0.045 | 0.943 | 279 | tags=52%, list=20%, signal=64% |
| 104 | GSE36476\_CTRL\_VS\_TSST\_ACT\_72H\_MEMORY\_CD4\_TCELL\_YOUNG\_UP |  | 35 | -0.37 | -2.10 | 0.000 | 0.044 | 0.944 | 513 | tags=71%, list=36%, signal=109% |
| 105 | GSE40666\_WT\_VS\_STAT1\_KO\_CD8\_TCELL\_UP |  | 25 | -0.42 | -2.10 | 0.002 | 0.044 | 0.944 | 379 | tags=64%, list=27%, signal=86% |
| 106 | GSE40274\_XBP1\_VS\_FOXP3\_AND\_XBP1\_TRANSDUCED\_ACTIVATED\_CD4\_TCELL\_UP |  | 22 | -0.44 | -2.10 | 0.000 | 0.044 | 0.944 | 410 | tags=68%, list=29%, signal=95% |
| 107 | GSE40666\_WT\_VS\_STAT1\_KO\_CD8\_TCELL\_WITH\_IFNA\_STIM\_90MIN\_DN |  | 26 | -0.40 | -2.10 | 0.005 | 0.045 | 0.950 | 698 | tags=88%, list=49%, signal=172% |
| 108 | GSE21546\_UNSTIM\_VS\_ANTI\_CD3\_STIM\_DP\_THYMOCYTES\_UP |  | 20 | -0.46 | -2.10 | 0.003 | 0.045 | 0.950 | 523 | tags=80%, list=37%, signal=125% |
| 109 | GSE36891\_POLYIC\_TLR3\_VS\_PAM\_TLR2\_STIM\_PERITONEAL\_MACROPHAGE\_UP |  | 23 | -0.44 | -2.09 | 0.000 | 0.045 | 0.956 | 278 | tags=52%, list=20%, signal=64% |
| 110 | GSE7852\_LN\_VS\_FAT\_TREG\_DN |  | 20 | -0.46 | -2.09 | 0.000 | 0.045 | 0.956 | 709 | tags=95%, list=50%, signal=188% |
| 111 | GSE36888\_UNTREATED\_VS\_IL2\_TREATED\_TCELL\_6H\_UP |  | 26 | -0.41 | -2.09 | 0.003 | 0.045 | 0.961 | 398 | tags=65%, list=28%, signal=89% |
| 112 | GSE43955\_TH0\_VS\_TGFB\_IL6\_IL23\_TH17\_ACT\_CD4\_TCELL\_60H\_DN |  | 31 | -0.38 | -2.09 | 0.002 | 0.045 | 0.964 | 571 | tags=81%, list=40%, signal=132% |
| 113 | GSE7852\_TREG\_VS\_TCONV\_LN\_UP |  | 27 | -0.41 | -2.09 | 0.005 | 0.046 | 0.970 | 176 | tags=41%, list=12%, signal=46% |
| 114 | GSE19198\_1H\_VS\_24H\_IL21\_TREATED\_TCELL\_DN |  | 19 | -0.46 | -2.08 | 0.000 | 0.046 | 0.972 | 250 | tags=53%, list=18%, signal=63% |
| 115 | GSE26156\_DOUBLE\_POSITIVE\_VS\_CD4\_SINGLE\_POSITIVE\_THYMOCYTE\_UP |  | 18 | -0.49 | -2.08 | 0.005 | 0.046 | 0.972 | 549 | tags=83%, list=39%, signal=135% |
| 116 | GSE7852\_LN\_VS\_THYMUS\_TREG\_UP |  | 28 | -0.39 | -2.08 | 0.000 | 0.048 | 0.975 | 202 | tags=43%, list=14%, signal=49% |
| 117 | GSE22601\_IMMATURE\_CD4\_SINGLE\_POSITIVE\_VS\_DOUBLE\_POSITIVE\_THYMOCYTE\_DN |  | 29 | -0.40 | -2.07 | 0.002 | 0.049 | 0.976 | 452 | tags=69%, list=32%, signal=99% |
| 118 | GOLDRATH\_NAIVE\_VS\_EFF\_CD8\_TCELL\_DN |  | 23 | -0.42 | -2.07 | 0.000 | 0.048 | 0.976 | 591 | tags=87%, list=42%, signal=147% |
| 119 | GSE25088\_IL4\_VS\_IL4\_AND\_ROSIGLITAZONE\_STIM\_STAT6\_KO\_MACROPHAGE\_DAY10\_UP |  | 29 | -0.41 | -2.07 | 0.005 | 0.049 | 0.978 | 169 | tags=38%, list=12%, signal=42% |
| 120 | GSE24142\_EARLY\_THYMIC\_PROGENITOR\_VS\_DN2\_THYMOCYTE\_ADULT\_UP |  | 23 | -0.42 | -2.06 | 0.005 | 0.052 | 0.986 | 513 | tags=74%, list=36%, signal=114% |
| 121 | GSE30971\_WBP7\_HET\_VS\_KO\_MACROPHAGE\_2H\_LPS\_STIM\_DN |  | 28 | -0.40 | -2.06 | 0.002 | 0.052 | 0.987 | 147 | tags=36%, list=10%, signal=39% |
| 122 | GSE9988\_ANTI\_TREM1\_AND\_LPS\_VS\_CTRL\_TREATED\_MONOCYTES\_UP |  | 35 | -0.36 | -2.06 | 0.003 | 0.052 | 0.989 | 606 | tags=83%, list=43%, signal=142% |
| 123 | GSE19888\_ADENOSINE\_A3R\_INH\_PRETREAT\_AND\_ACT\_BY\_A3R\_VS\_A3R\_INH\_AND\_TCELL\_MEMBRANES\_ACT\_MAST\_CELL\_UP |  | 44 | -0.34 | -2.06 | 0.000 | 0.053 | 0.990 | 220 | tags=36%, list=16%, signal=42% |
| 124 | GSE27092\_WT\_VS\_HDAC7\_PHOSPHO\_DEFICIENT\_CD8\_TCELL\_UP |  | 22 | -0.43 | -2.05 | 0.002 | 0.056 | 0.993 | 452 | tags=68%, list=32%, signal=99% |
| 125 | GSE22025\_TGFB1\_VS\_TGFB1\_AND\_PROGESTERONE\_TREATED\_CD4\_TCELL\_UP |  | 18 | -0.46 | -2.04 | 0.000 | 0.057 | 0.994 | 476 | tags=78%, list=34%, signal=116% |
| 126 | GSE7219\_WT\_VS\_NIK\_NFKB2\_KO\_DC\_UP |  | 25 | -0.41 | -2.04 | 0.000 | 0.057 | 0.994 | 456 | tags=68%, list=32%, signal=99% |
| 127 | GSE25085\_FETAL\_LIVER\_VS\_FETAL\_BM\_SP4\_THYMIC\_IMPLANT\_DN |  | 20 | -0.45 | -2.04 | 0.000 | 0.057 | 0.994 | 289 | tags=55%, list=20%, signal=68% |
| 128 | GSE43955\_TH0\_VS\_TGFB\_IL6\_IL23\_TH17\_ACT\_CD4\_TCELL\_60H\_UP |  | 20 | -0.45 | -2.04 | 0.000 | 0.057 | 0.994 | 518 | tags=80%, list=37%, signal=125% |
| 129 | GSE3920\_UNTREATED\_VS\_IFNG\_TREATED\_ENDOTHELIAL\_CELL\_UP |  | 21 | -0.44 | -2.04 | 0.002 | 0.057 | 0.994 | 534 | tags=76%, list=38%, signal=121% |
| 130 | GSE21360\_NAIVE\_VS\_PRIMARY\_MEMORY\_CD8\_TCELL\_DN |  | 18 | -0.47 | -2.04 | 0.002 | 0.056 | 0.994 | 164 | tags=44%, list=12%, signal=50% |
| 131 | GSE23502\_BM\_VS\_COLON\_TUMOR\_MYELOID\_DERIVED\_SUPPRESSOR\_CELL\_UP |  | 24 | -0.42 | -2.04 | 0.003 | 0.056 | 0.994 | 292 | tags=58%, list=21%, signal=72% |
| 132 | GSE34392\_ST2\_KO\_VS\_WT\_DAY8\_LCMV\_EFFECTOR\_CD8\_TCELL\_DN |  | 28 | -0.40 | -2.04 | 0.005 | 0.056 | 0.994 | 224 | tags=46%, list=16%, signal=54% |
| 133 | GSE13547\_2H\_VS\_12\_H\_ANTI\_IGM\_STIM\_ZFX\_KO\_BCELL\_UP |  | 27 | -0.40 | -2.03 | 0.003 | 0.057 | 0.995 | 367 | tags=56%, list=26%, signal=74% |
| 134 | GOLDRATH\_NAIVE\_VS\_MEMORY\_CD8\_TCELL\_DN |  | 20 | -0.44 | -2.03 | 0.000 | 0.059 | 0.997 | 445 | tags=70%, list=32%, signal=101% |
| 135 | GSE13306\_LAMINA\_PROPRIA\_VS\_SPLEEN\_TREG\_DN |  | 15 | -0.50 | -2.02 | 0.002 | 0.060 | 0.997 | 498 | tags=80%, list=35%, signal=122% |
| 136 | GSE9988\_LOW\_LPS\_VS\_VEHICLE\_TREATED\_MONOCYTE\_UP |  | 30 | -0.38 | -2.02 | 0.003 | 0.062 | 0.997 | 563 | tags=80%, list=40%, signal=130% |
| 137 | GSE21063\_CTRL\_VS\_ANTI\_IGM\_STIM\_BCELL\_8H\_UP |  | 20 | -0.43 | -2.01 | 0.007 | 0.065 | 0.998 | 236 | tags=50%, list=17%, signal=59% |
| 138 | GSE6092\_B\_BURGDOFERI\_VS\_B\_BURGDORFERI\_AND\_IFNG\_STIM\_ENDOTHELIAL\_CELL\_UP |  | 26 | -0.40 | -2.01 | 0.000 | 0.064 | 0.998 | 386 | tags=58%, list=27%, signal=78% |
| 139 | GSE21546\_SAP1A\_KO\_VS\_SAP1A\_KO\_AND\_ELK1\_KO\_ANTI\_CD3\_STIM\_DP\_THYMOCYTES\_UP |  | 19 | -0.45 | -2.01 | 0.005 | 0.065 | 0.998 | 153 | tags=37%, list=11%, signal=41% |
| 140 | GSE9988\_ANTI\_TREM1\_VS\_VEHICLE\_TREATED\_MONOCYTES\_UP |  | 35 | -0.36 | -2.01 | 0.003 | 0.065 | 0.998 | 550 | tags=74%, list=39%, signal=119% |
| 141 | GSE32423\_CTRL\_VS\_IL7\_IL4\_MEMORY\_CD8\_TCELL\_UP |  | 20 | -0.44 | -2.00 | 0.002 | 0.067 | 0.998 | 600 | tags=85%, list=43%, signal=146% |
| 142 | GSE25123\_CTRL\_VS\_ROSIGLITAZONE\_STIM\_PPARG\_KO\_MACROPHAGE\_UP |  | 24 | -0.41 | -2.00 | 0.005 | 0.067 | 0.998 | 501 | tags=79%, list=36%, signal=121% |
| 143 | GSE24634\_IL4\_VS\_CTRL\_TREATED\_NAIVE\_CD4\_TCELL\_DAY7\_DN |  | 25 | -0.41 | -2.00 | 0.000 | 0.068 | 0.999 | 599 | tags=88%, list=42%, signal=150% |
| 144 | GSE17721\_PAM3CSK4\_VS\_GADIQUIMOD\_2H\_BMDC\_UP |  | 20 | -0.43 | -1.99 | 0.005 | 0.069 | 0.999 | 500 | tags=75%, list=35%, signal=115% |
| 145 | GSE36476\_CTRL\_VS\_TSST\_ACT\_16H\_MEMORY\_CD4\_TCELL\_YOUNG\_UP |  | 25 | -0.41 | -1.99 | 0.005 | 0.069 | 0.999 | 555 | tags=80%, list=39%, signal=130% |
| 146 | GSE13411\_NAIVE\_VS\_MEMORY\_BCELL\_UP |  | 16 | -0.47 | -1.99 | 0.002 | 0.069 | 0.999 | 202 | tags=50%, list=14%, signal=58% |
| 147 | GSE15930\_NAIVE\_VS\_48H\_IN\_VITRO\_STIM\_IFNAB\_CD8\_TCELL\_UP |  | 16 | -0.48 | -1.99 | 0.000 | 0.069 | 0.999 | 207 | tags=50%, list=15%, signal=58% |
| 148 | GSE9988\_ANTI\_TREM1\_AND\_LPS\_VS\_VEHICLE\_TREATED\_MONOCYTES\_UP |  | 37 | -0.35 | -1.99 | 0.000 | 0.069 | 0.999 | 547 | tags=76%, list=39%, signal=120% |
| 149 | GSE40274\_CTRL\_VS\_GATA1\_TRANSDUCED\_ACTIVATED\_CD4\_TCELL\_DN |  | 20 | -0.42 | -1.99 | 0.002 | 0.069 | 0.999 | 319 | tags=55%, list=23%, signal=70% |
| 150 | GSE7852\_THYMUS\_VS\_FAT\_TREG\_DN |  | 28 | -0.38 | -1.99 | 0.000 | 0.069 | 0.999 | 395 | tags=61%, list=28%, signal=83% |
| 151 | GSE6674\_UNSTIM\_VS\_ANTI\_IGM\_AND\_CPG\_STIM\_BCELL\_UP |  | 32 | -0.36 | -1.99 | 0.003 | 0.068 | 0.999 | 414 | tags=59%, list=29%, signal=82% |
| 152 | GSE21670\_STAT3\_KO\_VS\_WT\_CD4\_TCELL\_TGFB\_TREATED\_DN |  | 17 | -0.47 | -1.99 | 0.003 | 0.068 | 0.999 | 356 | tags=65%, list=25%, signal=85% |
| 153 | GSE29618\_BCELL\_VS\_PDC\_DAY7\_FLU\_VACCINE\_UP |  | 22 | -0.42 | -1.99 | 0.002 | 0.068 | 0.999 | 539 | tags=77%, list=38%, signal=123% |
| 154 | GSE19198\_6H\_VS\_24H\_IL21\_TREATED\_TCELL\_UP |  | 18 | -0.45 | -1.99 | 0.002 | 0.068 | 0.999 | 550 | tags=83%, list=39%, signal=135% |
| 155 | GSE40666\_STAT1\_KO\_VS\_STAT4\_KO\_CD8\_TCELL\_WITH\_IFNA\_STIM\_90MIN\_DN |  | 15 | -0.48 | -1.98 | 0.002 | 0.068 | 0.999 | 258 | tags=53%, list=18%, signal=65% |
| 156 | GSE9988\_LPS\_VS\_VEHICLE\_TREATED\_MONOCYTE\_UP |  | 27 | -0.39 | -1.98 | 0.005 | 0.068 | 0.999 | 548 | tags=81%, list=39%, signal=131% |
| 157 | GSE42021\_TCONV\_PLN\_VS\_TREG\_PRECURSORS\_THYMUS\_DN |  | 39 | -0.34 | -1.98 | 0.000 | 0.068 | 0.999 | 570 | tags=72%, list=40%, signal=117% |
| 158 | GSE42021\_TCONV\_PLN\_VS\_CD24LO\_TCONV\_THYMUS\_DN |  | 24 | -0.41 | -1.98 | 0.000 | 0.068 | 0.999 | 596 | tags=83%, list=42%, signal=142% |
| 159 | GSE21774\_CD62L\_POS\_CD56\_BRIGHT\_VS\_CD62L\_NEG\_CD56\_DIM\_NK\_CELL\_DN |  | 25 | -0.40 | -1.98 | 0.002 | 0.069 | 0.999 | 302 | tags=52%, list=21%, signal=65% |
| 160 | GSE3565\_CTRL\_VS\_LPS\_INJECTED\_SPLENOCYTES\_UP |  | 21 | -0.43 | -1.98 | 0.012 | 0.069 | 0.999 | 557 | tags=81%, list=39%, signal=132% |
| 161 | GSE24142\_DN2\_VS\_DN3\_THYMOCYTE\_ADULT\_UP |  | 15 | -0.48 | -1.98 | 0.000 | 0.069 | 0.999 | 527 | tags=87%, list=37%, signal=137% |
| 162 | GSE25087\_FETAL\_VS\_ADULT\_TREG\_DN |  | 20 | -0.43 | -1.97 | 0.005 | 0.070 | 0.999 | 327 | tags=60%, list=23%, signal=77% |
| 163 | GSE12003\_MIR223\_KO\_VS\_WT\_BM\_PROGENITOR\_4D\_CULTURE\_UP |  | 21 | -0.42 | -1.97 | 0.007 | 0.072 | 1.000 | 332 | tags=52%, list=24%, signal=67% |
| 164 | GSE42021\_TCONV\_PLN\_VS\_CD24HI\_TCONV\_THYMUS\_UP |  | 27 | -0.37 | -1.97 | 0.003 | 0.072 | 1.000 | 528 | tags=74%, list=37%, signal=116% |
| 165 | GSE25147\_UNSTIM\_VS\_HELIOBACTER\_PYLORI\_LPS\_STIM\_MKN45\_CELL\_DN |  | 15 | -0.48 | -1.97 | 0.007 | 0.072 | 1.000 | 180 | tags=47%, list=13%, signal=53% |
| 166 | GSE26030\_TH1\_VS\_TH17\_DAY15\_POST\_POLARIZATION\_DN |  | 17 | -0.46 | -1.96 | 0.000 | 0.078 | 1.000 | 520 | tags=88%, list=37%, signal=138% |
| 167 | GSE29618\_BCELL\_VS\_MDC\_DAY7\_FLU\_VACCINE\_DN |  | 17 | -0.45 | -1.95 | 0.013 | 0.078 | 1.000 | 526 | tags=76%, list=37%, signal=120% |
| 168 | GSE24634\_IL4\_VS\_CTRL\_TREATED\_NAIVE\_CD4\_TCELL\_DAY10\_DN |  | 21 | -0.41 | -1.95 | 0.002 | 0.078 | 1.000 | 591 | tags=86%, list=42%, signal=145% |
| 169 | GSE17721\_CTRL\_VS\_POLYIC\_2H\_BMDC\_UP |  | 23 | -0.40 | -1.95 | 0.008 | 0.078 | 1.000 | 227 | tags=43%, list=16%, signal=51% |
| 170 | GSE17721\_CPG\_VS\_GARDIQUIMOD\_2H\_BMDC\_UP |  | 20 | -0.42 | -1.95 | 0.013 | 0.078 | 1.000 | 549 | tags=85%, list=39%, signal=137% |
| 171 | GSE12366\_NAIVE\_VS\_MEMORY\_BCELL\_DN |  | 25 | -0.39 | -1.95 | 0.010 | 0.078 | 1.000 | 290 | tags=52%, list=21%, signal=64% |
| 172 | GSE6259\_BCELL\_VS\_CD8\_TCELL\_DN |  | 36 | -0.34 | -1.95 | 0.003 | 0.079 | 1.000 | 538 | tags=72%, list=38%, signal=114% |
| 173 | GSE24142\_ADULT\_VS\_FETAL\_EARLY\_THYMIC\_PROGENITOR\_UP |  | 28 | -0.36 | -1.94 | 0.002 | 0.080 | 1.000 | 418 | tags=61%, list=30%, signal=85% |
| 174 | GSE21678\_WT\_VS\_FOXO1\_FOXO3\_KO\_TREG\_UP |  | 20 | -0.42 | -1.94 | 0.007 | 0.081 | 1.000 | 541 | tags=80%, list=38%, signal=128% |
| 175 | GSE23321\_CENTRAL\_MEMORY\_VS\_NAIVE\_CD8\_TCELL\_UP |  | 24 | -0.40 | -1.94 | 0.013 | 0.080 | 1.000 | 539 | tags=75%, list=38%, signal=119% |
| 176 | GSE44649\_WT\_VS\_MIR155\_KO\_ACTIVATED\_CD8\_TCELL\_UP |  | 19 | -0.44 | -1.94 | 0.009 | 0.081 | 1.000 | 491 | tags=79%, list=35%, signal=119% |
| 177 | GSE27241\_CTRL\_VS\_DIGOXIN\_TREATED\_RORGT\_KO\_CD4\_TCELL\_IN\_TH17\_POLARIZING\_CONDITIONS\_DN |  | 17 | -0.45 | -1.94 | 0.009 | 0.080 | 1.000 | 273 | tags=53%, list=19%, signal=65% |
| 178 | GSE13738\_RESTING\_VS\_BYSTANDER\_ACTIVATED\_CD4\_TCELL\_DN |  | 20 | -0.42 | -1.94 | 0.012 | 0.080 | 1.000 | 588 | tags=80%, list=42%, signal=135% |
| 179 | GSE33424\_CD161\_HIGH\_VS\_NEG\_CD8\_TCELL\_UP |  | 21 | -0.41 | -1.94 | 0.010 | 0.080 | 1.000 | 469 | tags=71%, list=33%, signal=105% |
| 180 | GSE26488\_WT\_VS\_HDAC7\_DELTAP\_TG\_OT2\_THYMOCYTE\_WITH\_PEPTIDE\_INJECTION\_DN |  | 25 | -0.40 | -1.94 | 0.007 | 0.080 | 1.000 | 349 | tags=52%, list=25%, signal=68% |
| 181 | GSE4984\_UNTREATED\_VS\_GALECTIN1\_TREATED\_DC\_UP |  | 26 | -0.38 | -1.94 | 0.012 | 0.080 | 1.000 | 419 | tags=69%, list=30%, signal=97% |
| 182 | GSE33425\_CD8\_ALPHAALPHA\_VS\_ALPHABETA\_CD161\_HIGH\_TCELL\_UP |  | 24 | -0.39 | -1.94 | 0.005 | 0.080 | 1.000 | 408 | tags=63%, list=29%, signal=86% |
| 183 | GSE27786\_LSK\_VS\_NKCELL\_DN |  | 21 | -0.41 | -1.94 | 0.007 | 0.080 | 1.000 | 359 | tags=52%, list=25%, signal=69% |
| 184 | GSE21360\_NAIVE\_VS\_PRIMARY\_MEMORY\_CD8\_TCELL\_UP |  | 16 | -0.47 | -1.93 | 0.005 | 0.081 | 1.000 | 482 | tags=81%, list=34%, signal=122% |
| 185 | GSE16385\_MONOCYTE\_VS\_MACROPHAGE\_DN |  | 16 | -0.46 | -1.93 | 0.003 | 0.081 | 1.000 | 247 | tags=56%, list=18%, signal=67% |
| 186 | GSE5542\_UNTREATED\_VS\_IFNG\_TREATED\_EPITHELIAL\_CELLS\_24H\_UP |  | 26 | -0.39 | -1.93 | 0.012 | 0.081 | 1.000 | 376 | tags=54%, list=27%, signal=72% |
| 187 | GSE45365\_WT\_VS\_IFNAR\_KO\_CD11B\_DC\_DN |  | 23 | -0.41 | -1.93 | 0.003 | 0.081 | 1.000 | 302 | tags=57%, list=21%, signal=71% |
| 188 | GSE9650\_EFFECTOR\_VS\_MEMORY\_CD8\_TCELL\_DN |  | 23 | -0.40 | -1.93 | 0.010 | 0.081 | 1.000 | 534 | tags=74%, list=38%, signal=117% |
| 189 | GSE36392\_TYPE\_2\_MYELOID\_VS\_NEUTROPHIL\_IL25\_TREATED\_LUNG\_UP |  | 24 | -0.39 | -1.93 | 0.003 | 0.081 | 1.000 | 561 | tags=83%, list=40%, signal=136% |
| 190 | GSE8384\_CTRL\_VS\_B\_ABORTUS\_4H\_MAC\_CELL\_LINE\_DN |  | 28 | -0.37 | -1.93 | 0.005 | 0.081 | 1.000 | 562 | tags=75%, list=40%, signal=122% |
| 191 | GSE557\_WT\_VS\_CIITA\_KO\_DC\_DN |  | 19 | -0.44 | -1.93 | 0.007 | 0.081 | 1.000 | 350 | tags=68%, list=25%, signal=90% |
| 192 | GSE5589\_LPS\_AND\_IL10\_VS\_LPS\_AND\_IL6\_STIM\_IL10\_KO\_MACROPHAGE\_45MIN\_UP |  | 17 | -0.45 | -1.93 | 0.010 | 0.081 | 1.000 | 713 | tags=94%, list=51%, signal=188% |
| 193 | GSE43863\_DAY6\_EFF\_VS\_DAY150\_MEM\_TH1\_CD4\_TCELL\_UP |  | 20 | -0.43 | -1.93 | 0.010 | 0.081 | 1.000 | 556 | tags=80%, list=39%, signal=130% |
| 194 | GSE17301\_CTRL\_VS\_48H\_IFNA2\_STIM\_CD8\_TCELL\_UP |  | 20 | -0.41 | -1.92 | 0.007 | 0.081 | 1.000 | 188 | tags=45%, list=13%, signal=51% |
| 195 | GSE2935\_UV\_INACTIVATED\_VS\_LIVE\_SENDAI\_VIRUS\_INF\_MACROPHAGE\_DN |  | 23 | -0.39 | -1.92 | 0.007 | 0.082 | 1.000 | 253 | tags=43%, list=18%, signal=52% |
| 196 | GSE17974\_CTRL\_VS\_ACT\_IL4\_AND\_ANTI\_IL12\_48H\_CD4\_TCELL\_UP |  | 24 | -0.40 | -1.92 | 0.003 | 0.082 | 1.000 | 189 | tags=42%, list=13%, signal=47% |
| 197 | GSE26912\_TUMORICIDAL\_VS\_CTRL\_MACROPHAGE\_DN |  | 15 | -0.46 | -1.92 | 0.002 | 0.082 | 1.000 | 328 | tags=60%, list=23%, signal=77% |
| 198 | GSE21063\_CTRL\_VS\_ANTI\_IGM\_STIM\_BCELL\_NFATC1\_KO\_3H\_UP |  | 19 | -0.42 | -1.92 | 0.002 | 0.082 | 1.000 | 455 | tags=74%, list=32%, signal=107% |
| 199 | GSE15930\_STIM\_VS\_STIM\_AND\_TRICHOSTATINA\_48H\_CD8\_T\_CELL\_UP |  | 20 | -0.43 | -1.91 | 0.002 | 0.086 | 1.000 | 499 | tags=80%, list=35%, signal=122% |
| 200 | GSE3982\_EOSINOPHIL\_VS\_CENT\_MEMORY\_CD4\_TCELL\_UP |  | 30 | -0.35 | -1.91 | 0.016 | 0.086 | 1.000 | 501 | tags=70%, list=36%, signal=106% |
| 201 | GSE27670\_CTRL\_VS\_BLIMP1\_TRANSDUCED\_GC\_BCELL\_UP |  | 18 | -0.44 | -1.91 | 0.012 | 0.086 | 1.000 | 645 | tags=89%, list=46%, signal=162% |
| 202 | GSE23308\_WT\_VS\_MINERALCORTICOID\_REC\_KO\_MACROPHAGE\_DN |  | 24 | -0.40 | -1.91 | 0.005 | 0.086 | 1.000 | 527 | tags=79%, list=37%, signal=124% |
| 203 | GSE2706\_R848\_VS\_R848\_AND\_LPS\_8H\_STIM\_DC\_DN |  | 26 | -0.39 | -1.91 | 0.000 | 0.086 | 1.000 | 599 | tags=85%, list=42%, signal=144% |
| 204 | GSE13522\_WT\_VS\_IFNAR\_KO\_SKING\_T\_CRUZI\_Y\_STRAIN\_INF\_UP |  | 29 | -0.37 | -1.91 | 0.002 | 0.086 | 1.000 | 445 | tags=66%, list=32%, signal=94% |
| 205 | GSE19772\_CTRL\_VS\_HCMV\_INF\_MONOCYTES\_UP |  | 24 | -0.39 | -1.91 | 0.013 | 0.086 | 1.000 | 124 | tags=29%, list=9%, signal=31% |
| 206 | GSE4535\_BM\_DERIVED\_DC\_VS\_FOLLICULAR\_DC\_UP |  | 20 | -0.41 | -1.91 | 0.002 | 0.086 | 1.000 | 606 | tags=85%, list=43%, signal=147% |
| 207 | GSE21379\_TFH\_VS\_NON\_TFH\_SAP\_KO\_CD4\_TCELL\_UP |  | 25 | -0.38 | -1.91 | 0.005 | 0.086 | 1.000 | 165 | tags=32%, list=12%, signal=36% |
| 208 | GSE21546\_WT\_VS\_ELK1\_KO\_ANTI\_CD3\_STIM\_DP\_THYMOCYTES\_UP |  | 17 | -0.45 | -1.90 | 0.012 | 0.087 | 1.000 | 537 | tags=76%, list=38%, signal=122% |
| 209 | GSE39152\_BRAIN\_VS\_SPLEEN\_CD103\_NEG\_MEMORY\_CD8\_TCELL\_DN |  | 24 | -0.38 | -1.90 | 0.010 | 0.089 | 1.000 | 731 | tags=92%, list=52%, signal=187% |
| 210 | KAECH\_NAIVE\_VS\_MEMORY\_CD8\_TCELL\_DN |  | 19 | -0.42 | -1.90 | 0.010 | 0.089 | 1.000 | 591 | tags=84%, list=42%, signal=143% |
| 211 | GSE24142\_EARLY\_THYMIC\_PROGENITOR\_VS\_DN3\_THYMOCYTE\_ADULT\_UP |  | 22 | -0.40 | -1.89 | 0.004 | 0.091 | 1.000 | 513 | tags=82%, list=36%, signal=127% |
| 212 | GSE17721\_LPS\_VS\_GARDIQUIMOD\_2H\_BMDC\_UP |  | 27 | -0.37 | -1.89 | 0.008 | 0.091 | 1.000 | 508 | tags=70%, list=36%, signal=108% |
| 213 | GSE3982\_CTRL\_VS\_LPS\_4H\_MAC\_DN |  | 28 | -0.36 | -1.89 | 0.015 | 0.093 | 1.000 | 547 | tags=75%, list=39%, signal=120% |
| 214 | GSE360\_L\_DONOVANI\_VS\_M\_TUBERCULOSIS\_MAC\_DN |  | 25 | -0.38 | -1.89 | 0.005 | 0.093 | 1.000 | 338 | tags=52%, list=24%, signal=67% |
| 215 | GSE37416\_0H\_VS\_48H\_F\_TULARENSIS\_LVS\_NEUTROPHIL\_DN |  | 21 | -0.41 | -1.89 | 0.010 | 0.093 | 1.000 | 548 | tags=76%, list=39%, signal=123% |
| 216 | GSE46606\_IRF4\_KO\_VS\_WT\_CD40L\_IL2\_IL5\_1DAY\_STIMULATED\_BCELL\_DN |  | 17 | -0.45 | -1.89 | 0.002 | 0.092 | 1.000 | 315 | tags=59%, list=22%, signal=75% |
| 217 | GSE21360\_SECONDARY\_VS\_QUATERNARY\_MEMORY\_CD8\_TCELL\_UP |  | 26 | -0.38 | -1.88 | 0.008 | 0.094 | 1.000 | 554 | tags=81%, list=39%, signal=131% |
| 218 | GSE7460\_FOXP3\_MUT\_VS\_WT\_ACT\_TCONV\_UP |  | 17 | -0.43 | -1.88 | 0.005 | 0.094 | 1.000 | 446 | tags=71%, list=32%, signal=102% |
| 219 | GSE19923\_WT\_VS\_HEB\_AND\_E2A\_KO\_DP\_THYMOCYTE\_DN |  | 28 | -0.36 | -1.88 | 0.007 | 0.094 | 1.000 | 278 | tags=46%, list=20%, signal=57% |
| 220 | GSE19923\_E2A\_KO\_VS\_E2A\_AND\_HEB\_KO\_DP\_THYMOCYTE\_DN |  | 27 | -0.38 | -1.88 | 0.003 | 0.096 | 1.000 | 620 | tags=81%, list=44%, signal=143% |
| 221 | GSE5142\_CTRL\_VS\_HTERT\_TRANSDUCED\_CD8\_TCELL\_EARLY\_PASSAGE\_CLONE\_UP |  | 21 | -0.40 | -1.88 | 0.005 | 0.096 | 1.000 | 266 | tags=52%, list=19%, signal=64% |
| 222 | GSE5542\_UNTREATED\_VS\_IFNA\_TREATED\_EPITHELIAL\_CELLS\_6H\_UP |  | 25 | -0.37 | -1.88 | 0.013 | 0.095 | 1.000 | 499 | tags=68%, list=35%, signal=103% |
| 223 | GSE13946\_CTRL\_VS\_DSS\_COLITIS\_GD\_TCELL\_FROM\_COLON\_UP |  | 23 | -0.40 | -1.88 | 0.011 | 0.095 | 1.000 | 508 | tags=74%, list=36%, signal=114% |
| 224 | GSE3982\_BCELL\_VS\_TH2\_UP |  | 20 | -0.42 | -1.88 | 0.012 | 0.095 | 1.000 | 204 | tags=45%, list=14%, signal=52% |
| 225 | GSE42724\_NAIVE\_VS\_MEMORY\_BCELL\_DN |  | 31 | -0.35 | -1.87 | 0.013 | 0.096 | 1.000 | 224 | tags=35%, list=16%, signal=41% |
| 226 | GSE30962\_PRIMARY\_VS\_SECONDARY\_CHRONIC\_LCMV\_INF\_CD8\_TCELL\_DN |  | 18 | -0.43 | -1.87 | 0.013 | 0.096 | 1.000 | 551 | tags=83%, list=39%, signal=135% |
| 227 | GSE37301\_PRO\_BCELL\_VS\_GRANULOCYTE\_MONOCYTE\_PROGENITOR\_UP |  | 18 | -0.42 | -1.87 | 0.007 | 0.096 | 1.000 | 157 | tags=39%, list=11%, signal=43% |
| 228 | GSE37605\_FOXP3\_FUSION\_GFP\_VS\_IRES\_GFP\_TREG\_C57BL6\_UP |  | 25 | -0.38 | -1.87 | 0.005 | 0.095 | 1.000 | 561 | tags=76%, list=40%, signal=124% |
| 229 | GSE37301\_MULTIPOTENT\_PROGENITOR\_VS\_LYMPHOID\_PRIMED\_MPP\_DN |  | 24 | -0.38 | -1.87 | 0.013 | 0.096 | 1.000 | 537 | tags=79%, list=38%, signal=126% |
| 230 | GSE40274\_FOXP3\_VS\_FOXP3\_AND\_IRF4\_TRANSDUCED\_ACTIVATED\_CD4\_TCELL\_UP |  | 17 | -0.43 | -1.87 | 0.005 | 0.099 | 1.000 | 269 | tags=53%, list=19%, signal=65% |
| 231 | GSE19198\_CTRL\_VS\_IL21\_TREATED\_TCELL\_24H\_DN |  | 20 | -0.40 | -1.87 | 0.012 | 0.099 | 1.000 | 613 | tags=85%, list=43%, signal=148% |
| 232 | GSE20366\_CD103\_KLRG1\_DP\_VS\_DN\_TREG\_DN |  | 15 | -0.46 | -1.86 | 0.012 | 0.099 | 1.000 | 448 | tags=73%, list=32%, signal=106% |
| 233 | GSE37532\_TREG\_VS\_TCONV\_PPARG\_KO\_CD4\_TCELL\_FROM\_VISCERAL\_ADIPOSE\_TISSUE\_DN |  | 31 | -0.34 | -1.86 | 0.005 | 0.099 | 1.000 | 523 | tags=71%, list=37%, signal=110% |
| 234 | GSE23505\_IL6\_IL1\_VS\_IL6\_IL1\_IL23\_TREATED\_CD4\_TCELL\_DN |  | 31 | -0.35 | -1.86 | 0.015 | 0.099 | 1.000 | 528 | tags=71%, list=37%, signal=111% |
| 235 | GSE36891\_UNSTIM\_VS\_PAM\_TLR2\_STIM\_PERITONEAL\_MACROPHAGE\_UP |  | 24 | -0.38 | -1.86 | 0.010 | 0.099 | 1.000 | 415 | tags=63%, list=29%, signal=87% |
| 236 | GSE24142\_ADULT\_VS\_FETAL\_DN3\_THYMOCYTE\_DN |  | 21 | -0.40 | -1.86 | 0.007 | 0.100 | 1.000 | 245 | tags=43%, list=17%, signal=51% |
| 237 | GSE1460\_DP\_VS\_CD4\_THYMOCYTE\_DN |  | 18 | -0.42 | -1.86 | 0.002 | 0.102 | 1.000 | 482 | tags=72%, list=34%, signal=108% |
| 238 | GSE43955\_TH0\_VS\_TGFB\_IL6\_TH17\_ACT\_CD4\_TCELL\_1H\_UP |  | 21 | -0.39 | -1.85 | 0.017 | 0.103 | 1.000 | 563 | tags=81%, list=40%, signal=133% |
| 239 | GSE15930\_NAIVE\_VS\_72H\_IN\_VITRO\_STIM\_CD8\_TCELL\_UP |  | 25 | -0.37 | -1.85 | 0.007 | 0.105 | 1.000 | 395 | tags=60%, list=28%, signal=82% |
| 240 | GSE6674\_CPG\_VS\_CPG\_AND\_ANTI\_IGM\_STIM\_BCELL\_DN |  | 37 | -0.32 | -1.85 | 0.005 | 0.105 | 1.000 | 230 | tags=38%, list=16%, signal=44% |
| 241 | GSE37416\_CTRL\_VS\_3H\_F\_TULARENSIS\_LVS\_NEUTROPHIL\_DN |  | 16 | -0.45 | -1.85 | 0.012 | 0.106 | 1.000 | 522 | tags=81%, list=37%, signal=127% |
| 242 | GSE7460\_TREG\_VS\_TCONV\_ACT\_WITH\_TGFB\_UP |  | 15 | -0.45 | -1.85 | 0.015 | 0.106 | 1.000 | 307 | tags=53%, list=22%, signal=67% |
| 243 | GSE11057\_EFF\_MEM\_VS\_CENT\_MEM\_CD4\_TCELL\_DN |  | 25 | -0.37 | -1.84 | 0.017 | 0.107 | 1.000 | 430 | tags=64%, list=30%, signal=90% |
| 244 | GSE29164\_UNTREATED\_VS\_CD8\_TCELL\_AND\_IL12\_TREATED\_MELANOMA\_DAY7\_DN |  | 18 | -0.41 | -1.84 | 0.015 | 0.107 | 1.000 | 680 | tags=89%, list=48%, signal=169% |
| 245 | GSE42021\_TREG\_PLN\_VS\_CD24INT\_TREG\_THYMUS\_UP |  | 41 | -0.30 | -1.84 | 0.011 | 0.109 | 1.000 | 570 | tags=68%, list=40%, signal=111% |
| 246 | GSE2706\_UNSTIM\_VS\_2H\_R848\_DC\_DN |  | 32 | -0.34 | -1.84 | 0.010 | 0.110 | 1.000 | 548 | tags=75%, list=39%, signal=120% |
| 247 | GSE16522\_MEMORY\_VS\_NAIVE\_CD8\_TCELL\_DN |  | 22 | -0.38 | -1.84 | 0.013 | 0.110 | 1.000 | 255 | tags=45%, list=18%, signal=55% |
| 248 | GSE10240\_IL22\_VS\_IL22\_AND\_IL17\_STIM\_PRIMARY\_BRONCHIAL\_EPITHELIAL\_CELLS\_UP |  | 19 | -0.41 | -1.84 | 0.014 | 0.110 | 1.000 | 342 | tags=58%, list=24%, signal=75% |
| 249 | GSE2706\_LPS\_VS\_R848\_AND\_LPS\_8H\_STIM\_DC\_DN |  | 20 | -0.40 | -1.84 | 0.007 | 0.110 | 1.000 | 599 | tags=80%, list=42%, signal=137% |
| 250 | GSE22919\_RESTING\_VS\_IL2\_IL12\_IL15\_STIM\_NK\_CELL\_DN |  | 21 | -0.39 | -1.83 | 0.007 | 0.110 | 1.000 | 250 | tags=48%, list=18%, signal=57% |
| 251 | GSE18893\_TCONV\_VS\_TREG\_2H\_TNF\_STIM\_DN |  | 38 | -0.31 | -1.83 | 0.011 | 0.110 | 1.000 | 299 | tags=45%, list=21%, signal=55% |
| 252 | GSE2770\_UNTREATED\_VS\_IL4\_TREATED\_ACT\_CD4\_TCELL\_6H\_UP |  | 19 | -0.41 | -1.83 | 0.000 | 0.111 | 1.000 | 379 | tags=63%, list=27%, signal=85% |
| 253 | GSE3982\_NEUTROPHIL\_VS\_EFF\_MEMORY\_CD4\_TCELL\_UP |  | 24 | -0.38 | -1.83 | 0.015 | 0.111 | 1.000 | 428 | tags=67%, list=30%, signal=94% |
| 254 | GSE2706\_2H\_VS\_8H\_R848\_AND\_LPS\_STIM\_DC\_DN |  | 20 | -0.39 | -1.83 | 0.019 | 0.111 | 1.000 | 572 | tags=80%, list=41%, signal=133% |
| 255 | GSE19923\_E2A\_KO\_VS\_HEB\_AND\_E2A\_KO\_DP\_THYMOCYTE\_UP |  | 28 | -0.35 | -1.83 | 0.010 | 0.111 | 1.000 | 348 | tags=50%, list=25%, signal=65% |
| 256 | GSE33162\_UNTREATED\_VS\_4H\_LPS\_STIM\_HDAC3\_HET\_MACROPHAGE\_DN |  | 20 | -0.39 | -1.83 | 0.007 | 0.111 | 1.000 | 177 | tags=35%, list=13%, signal=39% |
| 257 | GSE43955\_10H\_VS\_30H\_ACT\_CD4\_TCELL\_WITH\_TGFB\_IL6\_DN |  | 19 | -0.39 | -1.83 | 0.010 | 0.113 | 1.000 | 498 | tags=74%, list=35%, signal=112% |
| 258 | GSE25123\_WT\_VS\_PPARG\_KO\_MACROPHAGE\_IL4\_STIM\_DN |  | 26 | -0.36 | -1.83 | 0.007 | 0.112 | 1.000 | 342 | tags=58%, list=24%, signal=75% |
| 259 | GSE43863\_DAY6\_EFF\_VS\_DAY150\_MEM\_LY6C\_INT\_CXCR5POS\_CD4\_TCELL\_DN |  | 19 | -0.40 | -1.82 | 0.002 | 0.113 | 1.000 | 452 | tags=68%, list=32%, signal=99% |
| 260 | GSE30971\_CTRL\_VS\_LPS\_STIM\_MACROPHAGE\_WBP7\_KO\_4H\_DN |  | 24 | -0.36 | -1.82 | 0.009 | 0.113 | 1.000 | 416 | tags=58%, list=29%, signal=81% |
| 261 | GSE22886\_IGG\_IGA\_MEMORY\_BCELL\_VS\_BM\_PLASMA\_CELL\_DN |  | 20 | -0.40 | -1.82 | 0.012 | 0.113 | 1.000 | 195 | tags=40%, list=14%, signal=46% |
| 262 | GSE22886\_IGG\_IGA\_MEMORY\_BCELL\_VS\_BLOOD\_PLASMA\_CELL\_DN |  | 23 | -0.37 | -1.82 | 0.010 | 0.114 | 1.000 | 430 | tags=61%, list=30%, signal=86% |
| 263 | GSE5542\_UNTREATED\_VS\_IFNA\_TREATED\_EPITHELIAL\_CELLS\_24H\_UP |  | 26 | -0.36 | -1.82 | 0.005 | 0.114 | 1.000 | 368 | tags=50%, list=26%, signal=66% |
| 264 | GSE17721\_POLYIC\_VS\_GARDIQUIMOD\_16H\_BMDC\_UP |  | 20 | -0.39 | -1.82 | 0.015 | 0.114 | 1.000 | 349 | tags=55%, list=25%, signal=72% |
| 265 | GSE24142\_EARLY\_THYMIC\_PROGENITOR\_VS\_DN2\_THYMOCYTE\_UP |  | 15 | -0.44 | -1.82 | 0.022 | 0.114 | 1.000 | 543 | tags=80%, list=38%, signal=129% |
| 266 | GSE10240\_IL22\_VS\_IL22\_AND\_IL17\_STIM\_PRIMARY\_BRONCHIAL\_EPITHELIAL\_CELLS\_DN |  | 20 | -0.40 | -1.82 | 0.010 | 0.114 | 1.000 | 538 | tags=80%, list=38%, signal=127% |
| 267 | GSE15930\_NAIVE\_VS\_48H\_IN\_VITRO\_STIM\_CD8\_TCELL\_UP |  | 19 | -0.40 | -1.82 | 0.012 | 0.115 | 1.000 | 214 | tags=42%, list=15%, signal=49% |
| 268 | GSE24142\_EARLY\_THYMIC\_PROGENITOR\_VS\_DN2\_THYMOCYTE\_FETAL\_UP |  | 20 | -0.40 | -1.82 | 0.020 | 0.115 | 1.000 | 549 | tags=80%, list=39%, signal=129% |
| 269 | GSE43955\_10H\_VS\_60H\_ACT\_CD4\_TCELL\_WITH\_TGFB\_IL6\_DN |  | 17 | -0.44 | -1.81 | 0.012 | 0.115 | 1.000 | 518 | tags=82%, list=37%, signal=129% |
| 270 | GSE2706\_2H\_VS\_8H\_R848\_STIM\_DC\_DN |  | 24 | -0.36 | -1.81 | 0.013 | 0.116 | 1.000 | 236 | tags=46%, list=17%, signal=54% |
| 271 | GSE22935\_UNSTIM\_VS\_12H\_MBOVIS\_BCG\_STIM\_MACROPHAGE\_DN |  | 17 | -0.42 | -1.81 | 0.010 | 0.116 | 1.000 | 158 | tags=35%, list=11%, signal=39% |
| 272 | GSE42088\_2H\_VS\_24H\_LEISHMANIA\_INF\_DC\_UP |  | 16 | -0.44 | -1.81 | 0.019 | 0.115 | 1.000 | 578 | tags=88%, list=41%, signal=147% |
| 273 | GSE2405\_0H\_VS\_3H\_A\_PHAGOCYTOPHILUM\_STIM\_NEUTROPHIL\_UP |  | 21 | -0.38 | -1.81 | 0.019 | 0.118 | 1.000 | 549 | tags=81%, list=39%, signal=131% |
| 274 | GSE9988\_LOW\_LPS\_VS\_ANTI\_TREM1\_AND\_LPS\_MONOCYTE\_DN |  | 26 | -0.36 | -1.81 | 0.013 | 0.118 | 1.000 | 550 | tags=69%, list=39%, signal=111% |
| 275 | GSE22025\_UNTREATED\_VS\_TGFB1\_TREATED\_CD4\_TCELL\_UP |  | 24 | -0.38 | -1.81 | 0.014 | 0.118 | 1.000 | 313 | tags=54%, list=22%, signal=68% |
| 276 | GSE32034\_LY6C\_HIGH\_VS\_LOW\_MONOCYTE\_UP |  | 17 | -0.42 | -1.80 | 0.015 | 0.120 | 1.000 | 558 | tags=82%, list=40%, signal=135% |
| 277 | GSE5679\_CTRL\_VS\_PPARG\_LIGAND\_ROSIGLITAZONE\_AND\_RARA\_AGONIST\_AM580\_TREATED\_DC\_DN |  | 26 | -0.36 | -1.80 | 0.010 | 0.119 | 1.000 | 163 | tags=35%, list=12%, signal=38% |
| 278 | GSE20366\_CD103\_POS\_VS\_CD103\_KLRG1\_DP\_TREG\_DN |  | 21 | -0.38 | -1.80 | 0.017 | 0.119 | 1.000 | 283 | tags=48%, list=20%, signal=59% |
| 279 | GSE39864\_WT\_VS\_GATA3\_KO\_TREG\_UP |  | 20 | -0.38 | -1.80 | 0.015 | 0.120 | 1.000 | 293 | tags=50%, list=21%, signal=62% |
| 280 | GSE26030\_UNSTIM\_VS\_RESTIM\_TH17\_DAY5\_POST\_POLARIZATION\_DN |  | 22 | -0.38 | -1.80 | 0.005 | 0.120 | 1.000 | 387 | tags=59%, list=27%, signal=80% |
| 281 | GSE28726\_NAIVE\_VS\_ACTIVATED\_CD4\_TCELL\_UP |  | 18 | -0.41 | -1.80 | 0.008 | 0.120 | 1.000 | 315 | tags=56%, list=22%, signal=71% |
| 282 | GSE17974\_CTRL\_VS\_ACT\_IL4\_AND\_ANTI\_IL12\_2H\_CD4\_TCELL\_UP |  | 19 | -0.40 | -1.80 | 0.012 | 0.119 | 1.000 | 539 | tags=79%, list=38%, signal=126% |
| 283 | GOLDRATH\_NAIVE\_VS\_MEMORY\_CD8\_TCELL\_UP |  | 26 | -0.35 | -1.80 | 0.007 | 0.120 | 1.000 | 521 | tags=69%, list=37%, signal=108% |
| 284 | GSE6674\_ANTI\_IGM\_VS\_CPG\_STIM\_BCELL\_UP |  | 32 | -0.32 | -1.80 | 0.016 | 0.120 | 1.000 | 414 | tags=59%, list=29%, signal=82% |
| 285 | GSE17721\_4H\_VS\_24H\_POLYIC\_BMDC\_UP |  | 19 | -0.40 | -1.80 | 0.011 | 0.120 | 1.000 | 389 | tags=58%, list=28%, signal=79% |
| 286 | GSE369\_SOCS3\_KO\_VS\_IFNG\_KO\_LIVER\_DN |  | 23 | -0.38 | -1.80 | 0.020 | 0.120 | 1.000 | 358 | tags=57%, list=25%, signal=75% |
| 287 | GSE15330\_LYMPHOID\_MULTIPOTENT\_VS\_MEGAKARYOCYTE\_ERYTHROID\_PROGENITOR\_IKAROS\_KO\_DN |  | 22 | -0.38 | -1.80 | 0.019 | 0.119 | 1.000 | 563 | tags=77%, list=40%, signal=127% |
| 288 | GSE21360\_SECONDARY\_VS\_QUATERNARY\_MEMORY\_CD8\_TCELL\_DN |  | 17 | -0.43 | -1.80 | 0.014 | 0.119 | 1.000 | 482 | tags=76%, list=34%, signal=115% |
| 289 | KAECH\_NAIVE\_VS\_DAY15\_EFF\_CD8\_TCELL\_DN |  | 16 | -0.43 | -1.80 | 0.017 | 0.119 | 1.000 | 617 | tags=88%, list=44%, signal=154% |
| 290 | GSE8678\_IL7R\_LOW\_VS\_HIGH\_EFF\_CD8\_TCELL\_UP |  | 17 | -0.41 | -1.80 | 0.018 | 0.120 | 1.000 | 606 | tags=88%, list=43%, signal=153% |
| 291 | GSE21546\_UNSTIM\_VS\_ANTI\_CD3\_STIM\_SAP1A\_KO\_DP\_THYMOCYTES\_UP |  | 25 | -0.37 | -1.79 | 0.017 | 0.122 | 1.000 | 523 | tags=68%, list=37%, signal=106% |
| 292 | GSE40277\_EOS\_AND\_LEF1\_TRANSDUCED\_VS\_CTRL\_CD4\_TCELL\_UP |  | 16 | -0.43 | -1.79 | 0.014 | 0.122 | 1.000 | 361 | tags=63%, list=26%, signal=83% |
| 293 | GSE7219\_UNSTIM\_VS\_LPS\_AND\_ANTI\_CD40\_STIM\_NIK\_NFKB2\_KO\_DC\_DN |  | 29 | -0.34 | -1.79 | 0.014 | 0.121 | 1.000 | 245 | tags=41%, list=17%, signal=49% |
| 294 | GSE22886\_IGG\_IGA\_MEMORY\_BCELL\_VS\_BLOOD\_PLASMA\_CELL\_UP |  | 21 | -0.38 | -1.79 | 0.019 | 0.121 | 1.000 | 219 | tags=48%, list=16%, signal=56% |
| 295 | GSE22886\_DC\_VS\_MONOCYTE\_DN |  | 20 | -0.39 | -1.79 | 0.010 | 0.121 | 1.000 | 546 | tags=75%, list=39%, signal=121% |
| 296 | GSE39556\_UNTREATED\_VS\_3H\_POLYIC\_INJ\_MOUSE\_CD8A\_DC\_UP |  | 22 | -0.38 | -1.79 | 0.020 | 0.123 | 1.000 | 188 | tags=41%, list=13%, signal=46% |
| 297 | GSE17721\_CTRL\_VS\_POLYIC\_4H\_BMDC\_DN |  | 21 | -0.38 | -1.79 | 0.015 | 0.123 | 1.000 | 513 | tags=71%, list=36%, signal=111% |
| 298 | GSE17721\_ALL\_VS\_24H\_PAM3CSK4\_BMDC\_UP |  | 19 | -0.41 | -1.79 | 0.019 | 0.124 | 1.000 | 159 | tags=32%, list=11%, signal=35% |
| 299 | GSE38304\_MYC\_NEG\_VS\_POS\_GC\_BCELL\_UP |  | 20 | -0.39 | -1.78 | 0.015 | 0.124 | 1.000 | 256 | tags=45%, list=18%, signal=54% |
| 300 | GSE27786\_LIN\_NEG\_VS\_CD4\_TCELL\_DN |  | 20 | -0.40 | -1.78 | 0.012 | 0.124 | 1.000 | 584 | tags=80%, list=41%, signal=135% |
| 301 | GSE20198\_UNTREATED\_VS\_IFNA\_TREATED\_ACT\_CD4\_TCELL\_UP |  | 16 | -0.43 | -1.78 | 0.017 | 0.124 | 1.000 | 276 | tags=50%, list=20%, signal=61% |
| 302 | GSE2770\_IL4\_ACT\_VS\_ACT\_CD4\_TCELL\_48H\_DN |  | 16 | -0.43 | -1.78 | 0.020 | 0.124 | 1.000 | 429 | tags=69%, list=30%, signal=98% |
| 303 | GSE42021\_CD24HI\_VS\_CD24LOW\_TCONV\_THYMUS\_UP |  | 17 | -0.40 | -1.78 | 0.016 | 0.125 | 1.000 | 220 | tags=41%, list=16%, signal=48% |
| 304 | GSE20500\_RETINOIC\_ACID\_VS\_RARA\_ANTAGONIST\_TREATED\_CD4\_TCELL\_UP |  | 24 | -0.36 | -1.78 | 0.009 | 0.125 | 1.000 | 573 | tags=83%, list=41%, signal=138% |
| 305 | GSE39556\_CD8A\_DC\_VS\_NK\_CELL\_UP |  | 24 | -0.36 | -1.78 | 0.012 | 0.125 | 1.000 | 572 | tags=75%, list=41%, signal=124% |
| 306 | GSE14350\_IL2RB\_KO\_VS\_WT\_TREG\_DN |  | 24 | -0.36 | -1.78 | 0.023 | 0.126 | 1.000 | 562 | tags=79%, list=40%, signal=129% |
| 307 | GSE1432\_CTRL\_VS\_IFNG\_6H\_MICROGLIA\_DN |  | 30 | -0.33 | -1.78 | 0.015 | 0.125 | 1.000 | 527 | tags=70%, list=37%, signal=109% |
| 308 | GSE1448\_ANTI\_VALPHA2\_VS\_VBETA5\_DP\_THYMOCYTE\_DN |  | 18 | -0.41 | -1.78 | 0.013 | 0.126 | 1.000 | 158 | tags=39%, list=11%, signal=43% |
| 309 | GSE32164\_ALTERNATIVELY\_ACT\_M2\_VS\_CMYC\_INHIBITED\_MACROPHAGE\_DN |  | 18 | -0.40 | -1.78 | 0.010 | 0.126 | 1.000 | 618 | tags=83%, list=44%, signal=146% |
| 310 | GSE41867\_LCMV\_ARMSTRONG\_VS\_CLONE13\_DAY8\_EFFECTOR\_CD8\_TCELL\_DN |  | 20 | -0.39 | -1.77 | 0.008 | 0.128 | 1.000 | 864 | tags=100%, list=61%, signal=254% |
| 311 | GSE14415\_INDUCED\_TREG\_VS\_FAILED\_INDUCED\_TREG\_DN |  | 15 | -0.44 | -1.77 | 0.010 | 0.128 | 1.000 | 266 | tags=53%, list=19%, signal=65% |
| 312 | GSE7460\_TREG\_VS\_TCONV\_ACT\_UP |  | 23 | -0.36 | -1.77 | 0.015 | 0.128 | 1.000 | 269 | tags=43%, list=19%, signal=53% |
| 313 | GSE3691\_CONVENTIONAL\_VS\_PLASMACYTOID\_DC\_SPLEEN\_DN |  | 15 | -0.44 | -1.77 | 0.009 | 0.130 | 1.000 | 463 | tags=73%, list=33%, signal=108% |
| 314 | GSE23321\_CENTRAL\_VS\_EFFECTOR\_MEMORY\_CD8\_TCELL\_UP |  | 27 | -0.35 | -1.77 | 0.018 | 0.131 | 1.000 | 507 | tags=70%, list=36%, signal=108% |
| 315 | GSE28408\_LY6G\_POS\_VS\_NEG\_DC\_UP |  | 32 | -0.32 | -1.76 | 0.018 | 0.131 | 1.000 | 278 | tags=47%, list=20%, signal=57% |
| 316 | GSE3982\_BCELL\_VS\_NKCELL\_DN |  | 18 | -0.40 | -1.76 | 0.024 | 0.132 | 1.000 | 732 | tags=94%, list=52%, signal=194% |
| 317 | GSE3982\_DC\_VS\_BASOPHIL\_UP |  | 26 | -0.35 | -1.76 | 0.002 | 0.136 | 1.000 | 387 | tags=50%, list=27%, signal=68% |
| 318 | GSE7460\_TCONV\_VS\_TREG\_LN\_DN |  | 29 | -0.33 | -1.76 | 0.015 | 0.136 | 1.000 | 206 | tags=38%, list=15%, signal=44% |
| 319 | GSE37416\_0H\_VS\_6H\_F\_TULARENSIS\_LVS\_NEUTROPHIL\_DN |  | 24 | -0.35 | -1.76 | 0.010 | 0.136 | 1.000 | 539 | tags=75%, list=38%, signal=119% |
| 320 | GSE6259\_33D1\_POS\_VS\_DEC205\_POS\_SPLENIC\_DC\_DN |  | 30 | -0.32 | -1.76 | 0.019 | 0.135 | 1.000 | 386 | tags=57%, list=27%, signal=76% |
| 321 | GSE17974\_CTRL\_VS\_ACT\_IL4\_AND\_ANTI\_IL12\_4H\_CD4\_TCELL\_UP |  | 23 | -0.37 | -1.76 | 0.016 | 0.135 | 1.000 | 539 | tags=74%, list=38%, signal=118% |
| 322 | GSE43863\_DAY6\_EFF\_VS\_DAY150\_MEM\_TFH\_CD4\_TCELL\_UP |  | 24 | -0.36 | -1.75 | 0.014 | 0.136 | 1.000 | 542 | tags=75%, list=38%, signal=120% |
| 323 | GSE13306\_TREG\_VS\_TCONV\_LAMINA\_PROPRIA\_UP |  | 18 | -0.40 | -1.75 | 0.020 | 0.135 | 1.000 | 552 | tags=83%, list=39%, signal=135% |
| 324 | GSE12366\_PLASMA\_CELL\_VS\_MEMORY\_BCELL\_UP |  | 20 | -0.37 | -1.75 | 0.023 | 0.136 | 1.000 | 380 | tags=60%, list=27%, signal=81% |
| 325 | GSE360\_HIGH\_DOSE\_B\_MALAYI\_VS\_M\_TUBERCULOSIS\_MAC\_UP |  | 16 | -0.42 | -1.75 | 0.007 | 0.135 | 1.000 | 825 | tags=100%, list=58%, signal=238% |
| 326 | GSE3982\_MAST\_CELL\_VS\_BASOPHIL\_DN |  | 29 | -0.34 | -1.75 | 0.005 | 0.135 | 1.000 | 473 | tags=66%, list=34%, signal=97% |
| 327 | GSE6566\_STRONG\_VS\_WEAK\_DC\_STIMULATED\_CD4\_TCELL\_DN |  | 17 | -0.40 | -1.75 | 0.026 | 0.137 | 1.000 | 105 | tags=35%, list=7%, signal=38% |
| 328 | GSE22589\_HEALTHY\_VS\_HIV\_AND\_SIV\_INFECTED\_DC\_DN |  | 35 | -0.31 | -1.75 | 0.008 | 0.137 | 1.000 | 221 | tags=34%, list=16%, signal=40% |
| 329 | GSE17301\_IFNA2\_VS\_IFNA2\_AND\_ACD3\_ACD28\_STIM\_CD8\_TCELL\_DN |  | 19 | -0.39 | -1.75 | 0.017 | 0.138 | 1.000 | 482 | tags=68%, list=34%, signal=103% |
| 330 | GSE17721\_PAM3CSK4\_VS\_CPG\_4H\_BMDC\_DN |  | 20 | -0.38 | -1.75 | 0.014 | 0.138 | 1.000 | 527 | tags=75%, list=37%, signal=118% |
| 331 | GSE3982\_NEUTROPHIL\_VS\_BCELL\_UP |  | 23 | -0.37 | -1.75 | 0.023 | 0.138 | 1.000 | 190 | tags=39%, list=13%, signal=44% |
| 332 | GSE43955\_TH0\_VS\_TGFB\_IL6\_IL23\_TH17\_ACT\_CD4\_TCELL\_52H\_DN |  | 22 | -0.38 | -1.75 | 0.007 | 0.138 | 1.000 | 431 | tags=64%, list=31%, signal=90% |
| 333 | GSE5142\_HTERT\_TRANSDUCED\_VS\_CTRL\_CD8\_TCELL\_LATE\_PASSAGE\_CLONE\_DN |  | 23 | -0.36 | -1.75 | 0.015 | 0.138 | 1.000 | 560 | tags=78%, list=40%, signal=128% |
| 334 | GSE17721\_PAM3CSK4\_VS\_CPG\_16H\_BMDC\_DN |  | 16 | -0.42 | -1.75 | 0.018 | 0.137 | 1.000 | 557 | tags=81%, list=39%, signal=133% |
| 335 | GSE36392\_MAC\_VS\_NEUTROPHIL\_IL25\_TREATED\_LUNG\_DN |  | 22 | -0.37 | -1.75 | 0.023 | 0.138 | 1.000 | 572 | tags=82%, list=41%, signal=135% |
| 336 | GSE14415\_INDUCED\_TREG\_VS\_TCONV\_DN |  | 24 | -0.36 | -1.75 | 0.019 | 0.137 | 1.000 | 341 | tags=50%, list=24%, signal=65% |
| 337 | GSE19941\_IL10\_KO\_VS\_IL10\_KO\_AND\_NFKBP50\_KO\_UNSTIM\_MACROPHAGE\_DN |  | 21 | -0.37 | -1.75 | 0.021 | 0.137 | 1.000 | 460 | tags=71%, list=33%, signal=104% |
| 338 | GSE2706\_UNSTIM\_VS\_2H\_LPS\_DC\_DN |  | 27 | -0.33 | -1.74 | 0.033 | 0.137 | 1.000 | 547 | tags=74%, list=39%, signal=119% |
| 339 | GSE9988\_LPS\_VS\_LPS\_AND\_ANTI\_TREM1\_MONOCYTE\_DN |  | 25 | -0.35 | -1.74 | 0.031 | 0.138 | 1.000 | 301 | tags=44%, list=21%, signal=55% |
| 340 | GSE15330\_MEGAKARYOCYTE\_ERYTHROID\_VS\_GRANULOCYTE\_MONOCYTE\_PROGENITOR\_UP |  | 17 | -0.41 | -1.74 | 0.025 | 0.139 | 1.000 | 500 | tags=76%, list=35%, signal=117% |
| 341 | GSE360\_L\_MAJOR\_VS\_B\_MALAYI\_HIGH\_DOSE\_DC\_UP |  | 24 | -0.36 | -1.74 | 0.014 | 0.138 | 1.000 | 267 | tags=42%, list=19%, signal=51% |
| 342 | GSE3982\_MAST\_CELL\_VS\_TH2\_UP |  | 27 | -0.34 | -1.74 | 0.010 | 0.138 | 1.000 | 515 | tags=70%, list=36%, signal=109% |
| 343 | GSE34156\_NOD2\_LIGAND\_VS\_TLR1\_TLR2\_LIGAND\_6H\_TREATED\_MONOCYTE\_UP |  | 25 | -0.36 | -1.74 | 0.034 | 0.138 | 1.000 | 467 | tags=64%, list=33%, signal=94% |
| 344 | GSE40068\_BCL6\_POS\_VS\_NEG\_CXCR5\_POS\_TFH\_DN |  | 32 | -0.31 | -1.74 | 0.018 | 0.138 | 1.000 | 182 | tags=31%, list=13%, signal=35% |
| 345 | GSE26343\_UNSTIM\_VS\_LPS\_STIM\_MACROPHAGE\_DN |  | 15 | -0.43 | -1.74 | 0.012 | 0.138 | 1.000 | 502 | tags=80%, list=36%, signal=123% |
| 346 | GSE17322\_CD103\_POS\_VS\_CD11B\_HIGH\_LUNG\_DC\_UP |  | 15 | -0.43 | -1.74 | 0.021 | 0.138 | 1.000 | 78 | tags=33%, list=6%, signal=35% |
| 347 | GSE3982\_NEUTROPHIL\_VS\_NKCELL\_UP |  | 26 | -0.34 | -1.74 | 0.021 | 0.138 | 1.000 | 547 | tags=73%, list=39%, signal=117% |
| 348 | GSE3039\_NKT\_CELL\_VS\_B2\_BCELL\_DN |  | 35 | -0.31 | -1.74 | 0.015 | 0.138 | 1.000 | 447 | tags=54%, list=32%, signal=77% |
| 349 | GSE25123\_CTRL\_VS\_ROSIGLITAZONE\_STIM\_MACROPHAGE\_UP |  | 25 | -0.35 | -1.74 | 0.014 | 0.138 | 1.000 | 566 | tags=72%, list=40%, signal=118% |
| 350 | GSE14769\_UNSTIM\_VS\_80MIN\_LPS\_BMDM\_DN |  | 37 | -0.31 | -1.74 | 0.011 | 0.137 | 1.000 | 396 | tags=57%, list=28%, signal=77% |
| 351 | GSE22140\_HEALTHY\_VS\_ARTHRITIC\_MOUSE\_CD4\_TCELL\_UP |  | 20 | -0.39 | -1.74 | 0.026 | 0.139 | 1.000 | 613 | tags=80%, list=43%, signal=139% |
| 352 | GSE16266\_CTRL\_VS\_LPS\_STIM\_MEF\_UP |  | 18 | -0.39 | -1.73 | 0.017 | 0.141 | 1.000 | 170 | tags=39%, list=12%, signal=44% |
| 353 | GSE42021\_CD24HI\_TREG\_VS\_CD24HI\_TCONV\_THYMUS\_DN |  | 36 | -0.30 | -1.73 | 0.024 | 0.141 | 1.000 | 572 | tags=72%, list=41%, signal=118% |
| 354 | GSE40274\_CTRL\_VS\_XBP1\_TRANSDUCED\_ACTIVATED\_CD4\_TCELL\_UP |  | 19 | -0.38 | -1.73 | 0.021 | 0.141 | 1.000 | 460 | tags=68%, list=33%, signal=100% |
| 355 | GSE40274\_CTRL\_VS\_FOXP3\_AND\_IRF4\_TRANSDUCED\_ACTIVATED\_CD4\_TCELL\_UP |  | 16 | -0.42 | -1.73 | 0.041 | 0.145 | 1.000 | 313 | tags=56%, list=22%, signal=71% |
| 356 | GSE23321\_EFFECTOR\_MEMORY\_VS\_NAIVE\_CD8\_TCELL\_UP |  | 16 | -0.41 | -1.72 | 0.029 | 0.145 | 1.000 | 244 | tags=44%, list=17%, signal=52% |
| 357 | GSE5503\_MLN\_DC\_VS\_PLN\_DC\_ACTIVATED\_ALLOGENIC\_TCELL\_DN |  | 21 | -0.37 | -1.72 | 0.028 | 0.147 | 1.000 | 395 | tags=57%, list=28%, signal=78% |
| 358 | GSE7852\_TREG\_VS\_TCONV\_LN\_DN |  | 20 | -0.37 | -1.72 | 0.027 | 0.147 | 1.000 | 317 | tags=50%, list=22%, signal=64% |
| 359 | GSE22611\_NOD2\_TRANSDUCED\_VS\_CTRL\_HEK293T\_STIMULATED\_WITH\_MDP\_2H\_UP |  | 22 | -0.37 | -1.72 | 0.024 | 0.147 | 1.000 | 271 | tags=45%, list=19%, signal=55% |
| 360 | KAECH\_DAY15\_EFF\_VS\_MEMORY\_CD8\_TCELL\_UP |  | 17 | -0.41 | -1.72 | 0.022 | 0.147 | 1.000 | 408 | tags=59%, list=29%, signal=82% |
| 361 | GSE27291\_0H\_VS\_7D\_STIM\_GAMMADELTA\_TCELL\_UP |  | 18 | -0.40 | -1.72 | 0.024 | 0.148 | 1.000 | 395 | tags=61%, list=28%, signal=84% |
| 362 | GSE11864\_CSF1\_IFNG\_VS\_CSF1\_IFNG\_PAM3CYS\_IN\_MAC\_DN |  | 21 | -0.37 | -1.72 | 0.035 | 0.148 | 1.000 | 393 | tags=62%, list=28%, signal=85% |
| 363 | GSE5589\_IL6\_KO\_VS\_IL10\_KO\_LPS\_STIM\_MACROPHAGE\_45MIN\_DN |  | 18 | -0.40 | -1.72 | 0.016 | 0.148 | 1.000 | 164 | tags=39%, list=12%, signal=43% |
| 364 | GSE33424\_CD161\_INT\_VS\_NEG\_CD8\_TCELL\_DN |  | 17 | -0.40 | -1.72 | 0.024 | 0.148 | 1.000 | 513 | tags=76%, list=36%, signal=119% |
| 365 | GSE22886\_DAY0\_VS\_DAY7\_MONOCYTE\_IN\_CULTURE\_UP |  | 27 | -0.34 | -1.72 | 0.018 | 0.149 | 1.000 | 552 | tags=74%, list=39%, signal=119% |
| 366 | GSE2585\_CD80\_HIGH\_VS\_LOW\_AIRE\_KO\_MTEC\_UP |  | 16 | -0.41 | -1.72 | 0.021 | 0.148 | 1.000 | 455 | tags=69%, list=32%, signal=100% |
| 367 | GSE36476\_YOUNG\_VS\_OLD\_DONOR\_MEMORY\_CD4\_TCELL\_72H\_TSST\_ACT\_DN |  | 18 | -0.38 | -1.72 | 0.025 | 0.148 | 1.000 | 391 | tags=61%, list=28%, signal=83% |
| 368 | GSE32034\_LY6C\_HIGH\_VS\_LOW\_ROSIGLIZATONE\_TREATED\_MONOCYTE\_DN |  | 33 | -0.31 | -1.72 | 0.007 | 0.148 | 1.000 | 415 | tags=52%, list=29%, signal=71% |
| 369 | GSE339\_EX\_VIVO\_VS\_IN\_CULTURE\_CD8POS\_DC\_DN |  | 25 | -0.35 | -1.71 | 0.020 | 0.149 | 1.000 | 536 | tags=76%, list=38%, signal=120% |
| 370 | GSE31622\_WT\_VS\_KLF3\_KO\_BCELL\_DN |  | 19 | -0.38 | -1.71 | 0.016 | 0.150 | 1.000 | 207 | tags=37%, list=15%, signal=43% |
| 371 | GSE10325\_BCELL\_VS\_LUPUS\_BCELL\_DN |  | 28 | -0.32 | -1.71 | 0.025 | 0.152 | 1.000 | 562 | tags=71%, list=40%, signal=116% |
| 372 | GSE22886\_NAIVE\_CD4\_TCELL\_VS\_48H\_ACT\_TH1\_UP |  | 24 | -0.35 | -1.71 | 0.028 | 0.153 | 1.000 | 64 | tags=25%, list=5%, signal=26% |
| 373 | GSE24574\_BCL6\_HIGH\_VS\_LOW\_TFH\_CD4\_TCELL\_UP |  | 22 | -0.36 | -1.71 | 0.020 | 0.154 | 1.000 | 511 | tags=68%, list=36%, signal=105% |
| 374 | GSE11961\_GERMINAL\_CENTER\_BCELL\_DAY7\_VS\_GERMINAL\_CENTER\_BCELL\_DAY40\_UP |  | 22 | -0.35 | -1.71 | 0.028 | 0.153 | 1.000 | 373 | tags=50%, list=26%, signal=67% |
| 375 | GSE27859\_MACROPHAGE\_VS\_CD11C\_INT\_F480\_HI\_MACROPHAGE\_DN |  | 22 | -0.36 | -1.71 | 0.038 | 0.153 | 1.000 | 326 | tags=50%, list=23%, signal=64% |
| 376 | GSE26030\_TH1\_VS\_TH17\_DAY15\_POST\_POLARIZATION\_UP |  | 19 | -0.39 | -1.70 | 0.010 | 0.153 | 1.000 | 442 | tags=58%, list=31%, signal=83% |
| 377 | GSE21360\_PRIMARY\_VS\_QUATERNARY\_MEMORY\_CD8\_TCELL\_UP |  | 28 | -0.32 | -1.70 | 0.021 | 0.153 | 1.000 | 215 | tags=36%, list=15%, signal=41% |
| 378 | GSE7460\_CTRL\_VS\_TGFB\_TREATED\_ACT\_CD8\_TCELL\_DN |  | 20 | -0.38 | -1.70 | 0.029 | 0.154 | 1.000 | 125 | tags=35%, list=9%, signal=38% |
| 379 | GSE24142\_EARLY\_THYMIC\_PROGENITOR\_VS\_DN3\_THYMOCYTE\_UP |  | 17 | -0.39 | -1.70 | 0.028 | 0.154 | 1.000 | 550 | tags=76%, list=39%, signal=124% |
| 380 | GSE18791\_CTRL\_VS\_NEWCASTLE\_VIRUS\_DC\_18H\_DN |  | 16 | -0.40 | -1.70 | 0.030 | 0.155 | 1.000 | 456 | tags=75%, list=32%, signal=110% |
| 381 | GSE7460\_TCONV\_VS\_TREG\_THYMUS\_DN |  | 26 | -0.34 | -1.70 | 0.021 | 0.155 | 1.000 | 572 | tags=77%, list=41%, signal=127% |
| 382 | GSE360\_DC\_VS\_MAC\_B\_MALAYI\_HIGH\_DOSE\_DN |  | 17 | -0.40 | -1.70 | 0.020 | 0.155 | 1.000 | 576 | tags=82%, list=41%, signal=137% |
| 383 | GSE22935\_WT\_VS\_MYD88\_KO\_MACROPHAGE\_48H\_MBOVIS\_BCG\_STIM\_DN |  | 23 | -0.35 | -1.69 | 0.025 | 0.159 | 1.000 | 379 | tags=52%, list=27%, signal=70% |
| 384 | GSE17721\_PAM3CSK4\_VS\_GADIQUIMOD\_1H\_BMDC\_UP |  | 22 | -0.36 | -1.69 | 0.027 | 0.160 | 1.000 | 563 | tags=77%, list=40%, signal=127% |
| 385 | GSE32164\_RESTING\_DIFFERENTIATED\_VS\_CMYC\_INHIBITED\_MACROPHAGE\_UP |  | 20 | -0.36 | -1.69 | 0.021 | 0.160 | 1.000 | 408 | tags=55%, list=29%, signal=76% |
| 386 | GSE9650\_EXHAUSTED\_VS\_MEMORY\_CD8\_TCELL\_UP |  | 15 | -0.41 | -1.69 | 0.031 | 0.160 | 1.000 | 279 | tags=53%, list=20%, signal=66% |
| 387 | GSE8921\_UNSTIM\_VS\_TLR1\_2\_STIM\_MONOCYTE\_6H\_UP |  | 30 | -0.32 | -1.69 | 0.029 | 0.164 | 1.000 | 124 | tags=27%, list=9%, signal=29% |
| 388 | GSE360\_HIGH\_DOSE\_B\_MALAYI\_VS\_M\_TUBERCULOSIS\_MAC\_DN |  | 26 | -0.34 | -1.69 | 0.023 | 0.165 | 1.000 | 150 | tags=31%, list=11%, signal=34% |
| 389 | GSE22886\_UNSTIM\_VS\_STIM\_MEMORY\_TCELL\_UP |  | 16 | -0.40 | -1.68 | 0.028 | 0.165 | 1.000 | 393 | tags=63%, list=28%, signal=86% |
| 390 | GSE17974\_IL4\_AND\_ANTI\_IL12\_VS\_UNTREATED\_12H\_ACT\_CD4\_TCELL\_UP |  | 23 | -0.35 | -1.68 | 0.041 | 0.165 | 1.000 | 326 | tags=52%, list=23%, signal=67% |
| 391 | GSE7568\_IL4\_VS\_IL4\_AND\_DEXAMETHASONE\_TREATED\_MACROPHAGE\_DN |  | 20 | -0.37 | -1.68 | 0.020 | 0.167 | 1.000 | 355 | tags=50%, list=25%, signal=66% |
| 392 | GSE30971\_2H\_VS\_4H\_LPS\_STIM\_MACROPHAGE\_WBP7\_KO\_UP |  | 15 | -0.42 | -1.68 | 0.021 | 0.167 | 1.000 | 126 | tags=33%, list=9%, signal=36% |
| 393 | GSE39820\_TGFBETA3\_IL6\_VS\_TGFBETA3\_IL6\_IL23A\_TREATED\_CD4\_TCELL\_UP |  | 18 | -0.38 | -1.68 | 0.029 | 0.169 | 1.000 | 416 | tags=67%, list=29%, signal=93% |
| 394 | GSE22886\_NAIVE\_BCELL\_VS\_BM\_PLASMA\_CELL\_DN |  | 21 | -0.35 | -1.68 | 0.028 | 0.168 | 1.000 | 548 | tags=76%, list=39%, signal=123% |
| 395 | GSE4748\_CTRL\_VS\_LPS\_STIM\_DC\_3H\_DN |  | 20 | -0.36 | -1.68 | 0.015 | 0.169 | 1.000 | 607 | tags=80%, list=43%, signal=138% |
| 396 | GSE25123\_WT\_VS\_PPARG\_KO\_MACROPHAGE\_ROSIGLITAZONE\_STIM\_DN |  | 16 | -0.39 | -1.67 | 0.026 | 0.171 | 1.000 | 317 | tags=50%, list=22%, signal=64% |
| 397 | GSE36888\_STAT5\_AB\_KNOCKIN\_VS\_WT\_TCELL\_IL2\_TREATED\_6H\_DN |  | 15 | -0.40 | -1.67 | 0.022 | 0.171 | 1.000 | 357 | tags=60%, list=25%, signal=79% |
| 398 | GSE17974\_CTRL\_VS\_ACT\_IL4\_AND\_ANTI\_IL12\_72H\_CD4\_TCELL\_UP |  | 32 | -0.31 | -1.67 | 0.023 | 0.171 | 1.000 | 211 | tags=38%, list=15%, signal=43% |
| 399 | GSE32164\_RESTING\_DIFFERENTIATED\_VS\_ALTERNATIVELY\_ACT\_M2\_MACROPHAGE\_UP |  | 15 | -0.42 | -1.67 | 0.032 | 0.176 | 1.000 | 830 | tags=100%, list=59%, signal=240% |
| 400 | GSE37301\_MULTIPOTENT\_PROGENITOR\_VS\_PRO\_BCELL\_DN |  | 26 | -0.32 | -1.67 | 0.021 | 0.176 | 1.000 | 279 | tags=42%, list=20%, signal=52% |
| 401 | GSE17721\_LPS\_VS\_GARDIQUIMOD\_4H\_BMDC\_UP |  | 21 | -0.36 | -1.66 | 0.036 | 0.177 | 1.000 | 587 | tags=86%, list=42%, signal=145% |
| 402 | GSE23695\_CD57\_POS\_VS\_NEG\_NK\_CELL\_DN |  | 31 | -0.32 | -1.66 | 0.027 | 0.178 | 1.000 | 437 | tags=55%, list=31%, signal=78% |
| 403 | GSE36826\_WT\_VS\_IL1R\_KO\_SKIN\_UP |  | 25 | -0.33 | -1.66 | 0.035 | 0.178 | 1.000 | 273 | tags=44%, list=19%, signal=54% |
| 404 | GSE9650\_NAIVE\_VS\_MEMORY\_CD8\_TCELL\_UP |  | 27 | -0.32 | -1.66 | 0.034 | 0.179 | 1.000 | 188 | tags=33%, list=13%, signal=38% |
| 405 | GSE25677\_MPL\_VS\_R848\_STIM\_BCELL\_DN |  | 19 | -0.37 | -1.66 | 0.024 | 0.179 | 1.000 | 56 | tags=26%, list=4%, signal=27% |
| 406 | GSE3982\_CENT\_MEMORY\_CD4\_TCELL\_VS\_TH2\_DN |  | 18 | -0.38 | -1.66 | 0.025 | 0.179 | 1.000 | 883 | tags=100%, list=63%, signal=264% |
| 407 | GSE33162\_HDAC3\_KO\_VS\_HDAC3\_KO\_4H\_LPS\_STIM\_MACROPHAGE\_DN |  | 15 | -0.42 | -1.66 | 0.027 | 0.178 | 1.000 | 473 | tags=73%, list=34%, signal=109% |
| 408 | GSE33425\_CD161\_HIGH\_VS\_NEG\_CD8\_TCELL\_DN |  | 18 | -0.37 | -1.66 | 0.038 | 0.179 | 1.000 | 279 | tags=50%, list=20%, signal=62% |
| 409 | GSE20715\_0H\_VS\_24H\_OZONE\_LUNG\_DN |  | 27 | -0.33 | -1.66 | 0.021 | 0.180 | 1.000 | 431 | tags=59%, list=31%, signal=84% |
| 410 | GSE24634\_IL4\_VS\_CTRL\_TREATED\_NAIVE\_CD4\_TCELL\_DAY3\_DN |  | 22 | -0.35 | -1.66 | 0.026 | 0.180 | 1.000 | 547 | tags=73%, list=39%, signal=117% |
| 411 | GSE43863\_TH1\_VS\_TFH\_EFFECTOR\_CD4\_TCELL\_DN |  | 25 | -0.33 | -1.66 | 0.025 | 0.180 | 1.000 | 292 | tags=44%, list=21%, signal=54% |
| 412 | GSE21063\_CTRL\_VS\_ANTI\_IGM\_STIM\_BCELL\_NFATC1\_KO\_3H\_DN |  | 32 | -0.30 | -1.66 | 0.028 | 0.181 | 1.000 | 233 | tags=41%, list=17%, signal=48% |
| 413 | GSE13306\_TREG\_VS\_TCONV\_SPLEEN\_UP |  | 27 | -0.32 | -1.66 | 0.020 | 0.181 | 1.000 | 327 | tags=44%, list=23%, signal=57% |
| 414 | GSE45365\_NK\_CELL\_VS\_BCELL\_DN |  | 17 | -0.39 | -1.66 | 0.041 | 0.180 | 1.000 | 664 | tags=88%, list=47%, signal=165% |
| 415 | GSE7852\_LN\_VS\_THYMUS\_TCONV\_DN |  | 15 | -0.40 | -1.65 | 0.037 | 0.181 | 1.000 | 126 | tags=33%, list=9%, signal=36% |
| 416 | GSE21063\_WT\_VS\_NFATC1\_KO\_16H\_ANTI\_IGM\_STIM\_BCELL\_UP |  | 19 | -0.35 | -1.65 | 0.020 | 0.180 | 1.000 | 415 | tags=63%, list=29%, signal=88% |
| 417 | GSE41176\_UNSTIM\_VS\_ANTI\_IGM\_STIM\_BCELL\_1H\_UP |  | 25 | -0.34 | -1.65 | 0.056 | 0.182 | 1.000 | 572 | tags=76%, list=41%, signal=126% |
| 418 | GSE20715\_0H\_VS\_6H\_OZONE\_TLR4\_KO\_LUNG\_DN |  | 22 | -0.35 | -1.65 | 0.031 | 0.182 | 1.000 | 580 | tags=77%, list=41%, signal=129% |
| 419 | GSE19401\_UNSTIM\_VS\_RETINOIC\_ACID\_STIM\_FOLLICULAR\_DC\_DN |  | 28 | -0.33 | -1.65 | 0.026 | 0.182 | 1.000 | 665 | tags=82%, list=47%, signal=152% |
| 420 | GSE24574\_BCL6\_HIGH\_TFH\_VS\_TCONV\_CD4\_TCELL\_DN |  | 15 | -0.40 | -1.65 | 0.043 | 0.181 | 1.000 | 404 | tags=67%, list=29%, signal=92% |
| 421 | GSE17721\_0.5H\_VS\_4H\_LPS\_BMDC\_DN |  | 25 | -0.34 | -1.65 | 0.028 | 0.181 | 1.000 | 225 | tags=44%, list=16%, signal=51% |
| 422 | GSE5099\_MONOCYTE\_VS\_ALTERNATIVE\_M2\_MACROPHAGE\_DN |  | 22 | -0.35 | -1.65 | 0.028 | 0.181 | 1.000 | 203 | tags=36%, list=14%, signal=42% |
| 423 | GSE3565\_DUSP1\_VS\_WT\_SPLENOCYTES\_UP |  | 22 | -0.36 | -1.65 | 0.037 | 0.180 | 1.000 | 293 | tags=45%, list=21%, signal=56% |
| 424 | GSE15930\_STIM\_VS\_STIM\_AND\_TRICHOSTATINA\_24H\_CD8\_T\_CELL\_DN |  | 16 | -0.39 | -1.65 | 0.028 | 0.180 | 1.000 | 866 | tags=100%, list=61%, signal=256% |
| 425 | GSE17721\_POLYIC\_VS\_CPG\_12H\_BMDC\_UP |  | 17 | -0.38 | -1.65 | 0.024 | 0.180 | 1.000 | 167 | tags=35%, list=12%, signal=40% |
| 426 | GSE22601\_IMMATURE\_CD4\_SINGLE\_POSITIVE\_VS\_CD8\_SINGLE\_POSITIVE\_THYMOCYTE\_UP |  | 24 | -0.34 | -1.65 | 0.029 | 0.180 | 1.000 | 412 | tags=58%, list=29%, signal=81% |
| 427 | GSE22886\_NAIVE\_TCELL\_VS\_NKCELL\_DN |  | 19 | -0.37 | -1.65 | 0.038 | 0.180 | 1.000 | 154 | tags=32%, list=11%, signal=35% |
| 428 | GSE13484\_3H\_UNSTIM\_VS\_YF17D\_VACCINE\_STIM\_PBMC\_DN |  | 28 | -0.33 | -1.65 | 0.025 | 0.181 | 1.000 | 549 | tags=75%, list=39%, signal=120% |
| 429 | GSE17721\_CTRL\_VS\_PAM3CSK4\_1H\_BMDC\_DN |  | 19 | -0.36 | -1.65 | 0.042 | 0.181 | 1.000 | 266 | tags=42%, list=19%, signal=51% |
| 430 | GSE29618\_MONOCYTE\_VS\_MDC\_DAY7\_FLU\_VACCINE\_UP |  | 22 | -0.34 | -1.65 | 0.027 | 0.182 | 1.000 | 421 | tags=59%, list=30%, signal=83% |
| 431 | GSE36826\_NORMAL\_VS\_STAPH\_AUREUS\_INF\_SKIN\_UP |  | 27 | -0.33 | -1.65 | 0.031 | 0.182 | 1.000 | 535 | tags=70%, list=38%, signal=111% |
| 432 | GSE26559\_TCF1\_KO\_VS\_WT\_LIN\_NEG\_CELL\_UP |  | 26 | -0.32 | -1.64 | 0.041 | 0.184 | 1.000 | 303 | tags=46%, list=21%, signal=58% |
| 433 | GSE9988\_LOW\_LPS\_VS\_CTRL\_TREATED\_MONOCYTE\_UP |  | 28 | -0.33 | -1.64 | 0.033 | 0.186 | 1.000 | 588 | tags=79%, list=42%, signal=132% |
| 434 | GSE3982\_BASOPHIL\_VS\_CENT\_MEMORY\_CD4\_TCELL\_UP |  | 24 | -0.33 | -1.64 | 0.038 | 0.186 | 1.000 | 557 | tags=71%, list=39%, signal=115% |
| 435 | GSE27241\_WT\_CTRL\_VS\_DIGOXIN\_TREATED\_RORGT\_KO\_CD4\_TCELL\_IN\_TH17\_POLARIZING\_CONDITIONS\_UP |  | 18 | -0.37 | -1.64 | 0.034 | 0.186 | 1.000 | 292 | tags=50%, list=21%, signal=62% |
| 436 | GSE20366\_TREG\_VS\_TCONV\_DN |  | 21 | -0.35 | -1.64 | 0.027 | 0.189 | 1.000 | 468 | tags=71%, list=33%, signal=105% |
| 437 | GSE32164\_ALTERNATIVELY\_ACT\_M2\_VS\_CMYC\_INHIBITED\_MACROPHAGE\_UP |  | 15 | -0.39 | -1.64 | 0.040 | 0.189 | 1.000 | 218 | tags=47%, list=15%, signal=55% |
| 438 | GSE19198\_CTRL\_VS\_IL21\_TREATED\_TCELL\_1H\_DN |  | 21 | -0.36 | -1.64 | 0.035 | 0.189 | 1.000 | 599 | tags=81%, list=42%, signal=139% |
| 439 | GSE26343\_UNSTIM\_VS\_LPS\_STIM\_NFAT5\_KO\_MACROPHAGE\_DN |  | 26 | -0.33 | -1.63 | 0.045 | 0.190 | 1.000 | 631 | tags=81%, list=45%, signal=143% |
| 440 | GSE17721\_LPS\_VS\_GARDIQUIMOD\_12H\_BMDC\_DN |  | 16 | -0.39 | -1.63 | 0.046 | 0.191 | 1.000 | 867 | tags=100%, list=61%, signal=256% |
| 441 | GSE4748\_CTRL\_VS\_CYANOBACTERIUM\_LPSLIKE\_STIM\_DC\_3H\_UP |  | 17 | -0.37 | -1.63 | 0.037 | 0.194 | 1.000 | 225 | tags=41%, list=16%, signal=48% |
| 442 | GSE37416\_0H\_VS\_3H\_F\_TULARENSIS\_LVS\_NEUTROPHIL\_DN |  | 26 | -0.32 | -1.63 | 0.032 | 0.194 | 1.000 | 633 | tags=77%, list=45%, signal=137% |
| 443 | GSE22140\_GERMFREE\_VS\_SPF\_MOUSE\_CD4\_TCELL\_UP |  | 24 | -0.34 | -1.63 | 0.043 | 0.195 | 1.000 | 527 | tags=71%, list=37%, signal=111% |
| 444 | GSE360\_LOW\_DOSE\_B\_MALAYI\_VS\_M\_TUBERCULOSIS\_DC\_DN |  | 29 | -0.31 | -1.63 | 0.031 | 0.196 | 1.000 | 599 | tags=76%, list=42%, signal=129% |
| 445 | GSE22601\_DOUBLE\_NEGATIVE\_VS\_CD4\_SINGLE\_POSITIVE\_THYMOCYTE\_UP |  | 37 | -0.28 | -1.63 | 0.026 | 0.195 | 1.000 | 578 | tags=68%, list=41%, signal=111% |
| 446 | GSE26030\_TH1\_VS\_TH17\_RESTIMULATED\_DAY5\_POST\_POLARIZATION\_UP |  | 16 | -0.38 | -1.63 | 0.053 | 0.195 | 1.000 | 887 | tags=100%, list=63%, signal=266% |
| 447 | GSE6269\_HEALTHY\_VS\_STAPH\_PNEUMO\_INF\_PBMC\_DN |  | 20 | -0.35 | -1.62 | 0.033 | 0.196 | 1.000 | 460 | tags=70%, list=33%, signal=102% |
| 448 | GSE39110\_UNTREATED\_VS\_IL2\_TREATED\_CD8\_TCELL\_DAY3\_POST\_IMMUNIZATION\_DN |  | 15 | -0.40 | -1.62 | 0.033 | 0.196 | 1.000 | 126 | tags=40%, list=9%, signal=43% |
| 449 | GSE4142\_NAIVE\_VS\_GC\_BCELL\_UP |  | 18 | -0.37 | -1.62 | 0.037 | 0.197 | 1.000 | 499 | tags=72%, list=35%, signal=110% |
| 450 | GSE35685\_CD34POS\_CD38NEG\_VS\_CD34POS\_CD10NEG\_CD62LPOS\_BONE\_MARROW\_DN |  | 19 | -0.36 | -1.62 | 0.035 | 0.196 | 1.000 | 245 | tags=42%, list=17%, signal=50% |
| 451 | GSE20366\_EX\_VIVO\_VS\_DEC205\_CONVERSION\_DN |  | 25 | -0.33 | -1.62 | 0.026 | 0.196 | 1.000 | 527 | tags=68%, list=37%, signal=107% |
| 452 | GSE29618\_PDC\_VS\_MDC\_UP |  | 21 | -0.34 | -1.62 | 0.043 | 0.196 | 1.000 | 560 | tags=76%, list=40%, signal=124% |
| 453 | GSE42021\_TREG\_VS\_TCONV\_PLN\_DN |  | 21 | -0.36 | -1.62 | 0.035 | 0.196 | 1.000 | 316 | tags=48%, list=22%, signal=60% |
| 454 | KAECH\_DAY8\_EFF\_VS\_MEMORY\_CD8\_TCELL\_DN |  | 23 | -0.34 | -1.62 | 0.038 | 0.197 | 1.000 | 214 | tags=39%, list=15%, signal=45% |
| 455 | GSE3982\_NEUTROPHIL\_VS\_CENT\_MEMORY\_CD4\_TCELL\_UP |  | 30 | -0.30 | -1.62 | 0.025 | 0.197 | 1.000 | 417 | tags=57%, list=30%, signal=79% |
| 456 | GSE25088\_IL4\_VS\_IL4\_AND\_ROSIGLITAZONE\_STIM\_MACROPHAGE\_DAY10\_DN |  | 22 | -0.35 | -1.62 | 0.028 | 0.197 | 1.000 | 418 | tags=59%, list=30%, signal=83% |
| 457 | GSE24574\_NAIVE\_VS\_TCONV\_CD4\_TCELL\_UP |  | 21 | -0.35 | -1.62 | 0.029 | 0.198 | 1.000 | 549 | tags=76%, list=39%, signal=123% |
| 458 | GSE41867\_DAY6\_EFFECTOR\_VS\_DAY30\_MEMORY\_CD8\_TCELL\_LCMV\_ARMSTRONG\_UP |  | 21 | -0.34 | -1.62 | 0.029 | 0.198 | 1.000 | 362 | tags=52%, list=26%, signal=69% |
| 459 | GSE18893\_CTRL\_VS\_TNF\_TREATED\_TREG\_24H\_DN |  | 23 | -0.34 | -1.61 | 0.030 | 0.202 | 1.000 | 350 | tags=57%, list=25%, signal=74% |
| 460 | GSE22886\_NAIVE\_CD8\_TCELL\_VS\_NKCELL\_DN |  | 25 | -0.33 | -1.61 | 0.039 | 0.203 | 1.000 | 83 | tags=24%, list=6%, signal=25% |
| 461 | GSE3982\_DC\_VS\_NEUTROPHIL\_LPS\_STIM\_DN |  | 17 | -0.38 | -1.61 | 0.055 | 0.203 | 1.000 | 403 | tags=65%, list=29%, signal=89% |
| 462 | GSE36476\_CTRL\_VS\_TSST\_ACT\_40H\_MEMORY\_CD4\_TCELL\_YOUNG\_UP |  | 28 | -0.32 | -1.61 | 0.043 | 0.203 | 1.000 | 511 | tags=71%, list=36%, signal=110% |
| 463 | GSE17721\_LPS\_VS\_POLYIC\_12H\_BMDC\_DN |  | 15 | -0.40 | -1.61 | 0.056 | 0.204 | 1.000 | 420 | tags=60%, list=30%, signal=85% |
| 464 | GSE14699\_NAIVE\_VS\_ACT\_CD8\_TCELL\_UP |  | 22 | -0.34 | -1.61 | 0.045 | 0.204 | 1.000 | 612 | tags=82%, list=43%, signal=142% |
| 465 | GSE17974\_IL4\_AND\_ANTI\_IL12\_VS\_UNTREATED\_2H\_ACT\_CD4\_TCELL\_UP |  | 17 | -0.37 | -1.61 | 0.035 | 0.204 | 1.000 | 419 | tags=65%, list=30%, signal=91% |
| 466 | GSE37416\_0H\_VS\_48H\_F\_TULARENSIS\_LVS\_NEUTROPHIL\_UP |  | 21 | -0.34 | -1.61 | 0.033 | 0.204 | 1.000 | 192 | tags=38%, list=14%, signal=43% |
| 467 | GSE16385\_ROSIGLITAZONE\_IFNG\_TNF\_VS\_IL4\_STIM\_MACROPHAGE\_UP |  | 32 | -0.30 | -1.61 | 0.038 | 0.204 | 1.000 | 536 | tags=66%, list=38%, signal=103% |
| 468 | GSE3982\_CTRL\_VS\_LPS\_48H\_DC\_DN |  | 26 | -0.32 | -1.61 | 0.026 | 0.204 | 1.000 | 748 | tags=88%, list=53%, signal=185% |
| 469 | GSE37301\_LYMPHOID\_PRIMED\_MPP\_VS\_COMMON\_LYMPHOID\_PROGENITOR\_DN |  | 23 | -0.33 | -1.61 | 0.034 | 0.205 | 1.000 | 857 | tags=96%, list=61%, signal=240% |
| 470 | GSE42088\_UNINF\_VS\_LEISHMANIA\_INF\_DC\_2H\_DN |  | 19 | -0.37 | -1.61 | 0.048 | 0.205 | 1.000 | 500 | tags=68%, list=35%, signal=105% |
| 471 | GSE18791\_CTRL\_VS\_NEWCASTLE\_VIRUS\_DC\_12H\_DN |  | 30 | -0.30 | -1.61 | 0.016 | 0.205 | 1.000 | 288 | tags=40%, list=20%, signal=49% |
| 472 | GSE2128\_C57BL6\_VS\_NOD\_CD4CD8\_DP\_THYMOCYTE\_UP |  | 27 | -0.31 | -1.60 | 0.031 | 0.205 | 1.000 | 322 | tags=44%, list=23%, signal=56% |
| 473 | GSE13306\_RA\_VS\_UNTREATED\_MEM\_CD4\_TCELL\_DN |  | 31 | -0.30 | -1.60 | 0.041 | 0.205 | 1.000 | 547 | tags=68%, list=39%, signal=108% |
| 474 | GSE39820\_CTRL\_VS\_TGFBETA1\_IL6\_CD4\_TCELL\_DN |  | 24 | -0.33 | -1.60 | 0.044 | 0.205 | 1.000 | 552 | tags=71%, list=39%, signal=114% |
| 475 | GSE40274\_FOXP3\_VS\_FOXP3\_AND\_EOS\_TRANSDUCED\_ACTIVATED\_CD4\_TCELL\_UP |  | 23 | -0.34 | -1.60 | 0.035 | 0.207 | 1.000 | 55 | tags=22%, list=4%, signal=22% |
| 476 | GSE360\_L\_DONOVANI\_VS\_B\_MALAYI\_LOW\_DOSE\_DC\_UP |  | 31 | -0.30 | -1.60 | 0.032 | 0.210 | 1.000 | 599 | tags=74%, list=42%, signal=126% |
| 477 | GSE19772\_HCMV\_INFL\_VS\_HCMV\_INF\_MONOCYTES\_AND\_PI3K\_INHIBITION\_DN |  | 24 | -0.33 | -1.60 | 0.029 | 0.211 | 1.000 | 297 | tags=42%, list=21%, signal=52% |
| 478 | GSE3203\_UNTREATED\_VS\_IFNB\_TREATED\_LN\_BCELL\_DN |  | 15 | -0.38 | -1.60 | 0.057 | 0.211 | 1.000 | 276 | tags=47%, list=20%, signal=57% |
| 479 | GSE16385\_ROSIGLITAZONE\_IL4\_VS\_IFNG\_TNF\_STIM\_MACROPHAGE\_UP |  | 23 | -0.32 | -1.60 | 0.040 | 0.211 | 1.000 | 328 | tags=48%, list=23%, signal=61% |
| 480 | GSE4984\_LPS\_VS\_VEHICLE\_CTRL\_TREATED\_DC\_DN |  | 25 | -0.32 | -1.60 | 0.030 | 0.212 | 1.000 | 359 | tags=52%, list=25%, signal=69% |
| 481 | GSE17721\_CTRL\_VS\_POLYIC\_8H\_BMDC\_DN |  | 20 | -0.35 | -1.59 | 0.039 | 0.212 | 1.000 | 433 | tags=65%, list=31%, signal=92% |
| 482 | GSE29949\_MICROGLIA\_VS\_DC\_BRAIN\_DN |  | 17 | -0.37 | -1.59 | 0.038 | 0.213 | 1.000 | 746 | tags=94%, list=53%, signal=197% |
| 483 | GSE40274\_CTRL\_VS\_FOXP3\_AND\_XBP1\_TRANSDUCED\_ACTIVATED\_CD4\_TCELL\_DN |  | 18 | -0.36 | -1.59 | 0.046 | 0.212 | 1.000 | 542 | tags=78%, list=38%, signal=125% |
| 484 | GSE43955\_10H\_VS\_30H\_ACT\_CD4\_TCELL\_DN |  | 16 | -0.37 | -1.59 | 0.050 | 0.212 | 1.000 | 167 | tags=31%, list=12%, signal=35% |
| 485 | GSE39110\_UNTREATED\_VS\_IL2\_TREATED\_CD8\_TCELL\_DAY6\_POST\_IMMUNIZATION\_DN |  | 23 | -0.33 | -1.59 | 0.047 | 0.211 | 1.000 | 483 | tags=65%, list=34%, signal=98% |
| 486 | GSE28726\_ACT\_CD4\_TCELL\_VS\_ACT\_VA24NEG\_NKTCELL\_DN |  | 24 | -0.33 | -1.59 | 0.041 | 0.211 | 1.000 | 214 | tags=38%, list=15%, signal=43% |
| 487 | GSE42088\_UNINF\_VS\_LEISHMANIA\_INF\_DC\_24H\_UP |  | 17 | -0.37 | -1.59 | 0.043 | 0.212 | 1.000 | 500 | tags=71%, list=35%, signal=108% |
| 488 | GSE41087\_WT\_VS\_FOXP3\_MUT\_ANTI\_CD3\_CD28\_STIM\_CD4\_TCELL\_UP |  | 24 | -0.32 | -1.59 | 0.035 | 0.212 | 1.000 | 547 | tags=75%, list=39%, signal=120% |
| 489 | GSE22886\_DAY0\_VS\_DAY1\_MONOCYTE\_IN\_CULTURE\_DN |  | 31 | -0.30 | -1.59 | 0.029 | 0.211 | 1.000 | 560 | tags=71%, list=40%, signal=115% |
| 490 | GSE3720\_UNSTIM\_VS\_LPS\_STIM\_VD2\_GAMMADELTA\_TCELL\_DN |  | 16 | -0.38 | -1.59 | 0.049 | 0.212 | 1.000 | 542 | tags=75%, list=38%, signal=120% |
| 491 | GSE4590\_SMALL\_VS\_LARGE\_PRE\_BCELL\_DN |  | 18 | -0.36 | -1.59 | 0.036 | 0.212 | 1.000 | 465 | tags=67%, list=33%, signal=98% |
| 492 | GSE7460\_WT\_VS\_FOXP3\_HET\_ACT\_TCONV\_UP |  | 18 | -0.36 | -1.59 | 0.051 | 0.213 | 1.000 | 385 | tags=61%, list=27%, signal=83% |
| 493 | GSE26495\_NAIVE\_VS\_PD1HIGH\_CD8\_TCELL\_DN |  | 25 | -0.31 | -1.59 | 0.050 | 0.212 | 1.000 | 252 | tags=36%, list=18%, signal=43% |
| 494 | GSE3982\_CENT\_MEMORY\_CD4\_TCELL\_VS\_TH1\_DN |  | 20 | -0.35 | -1.59 | 0.068 | 0.213 | 1.000 | 683 | tags=85%, list=48%, signal=162% |
| 495 | GSE29618\_MONOCYTE\_VS\_MDC\_UP |  | 24 | -0.32 | -1.59 | 0.048 | 0.214 | 1.000 | 557 | tags=75%, list=39%, signal=122% |
| 496 | GSE6092\_IFNG\_VS\_IFNG\_AND\_B\_BURGDORFERI\_INF\_ENDOTHELIAL\_CELL\_UP |  | 24 | -0.32 | -1.59 | 0.042 | 0.214 | 1.000 | 380 | tags=54%, list=27%, signal=73% |
| 497 | GSE29618\_BCELL\_VS\_MDC\_DAY7\_FLU\_VACCINE\_UP |  | 25 | -0.32 | -1.59 | 0.055 | 0.214 | 1.000 | 242 | tags=40%, list=17%, signal=47% |
| 498 | GSE29618\_MONOCYTE\_VS\_PDC\_UP |  | 22 | -0.33 | -1.58 | 0.040 | 0.215 | 1.000 | 301 | tags=45%, list=21%, signal=57% |
| 499 | GSE33292\_DN3\_THYMOCYTE\_VS\_TCF1\_KO\_TCELL\_LYMPHOMA\_UP |  | 16 | -0.38 | -1.58 | 0.029 | 0.215 | 1.000 | 786 | tags=94%, list=56%, signal=209% |
| 500 | GSE37533\_PPARG1\_FOXP3\_VS\_FOXP3\_TRANSDUCED\_CD4\_TCELL\_PIOGLITAZONE\_TREATED\_UP |  | 27 | -0.31 | -1.58 | 0.039 | 0.215 | 1.000 | 370 | tags=48%, list=26%, signal=64% |
| 501 | GSE21033\_CTRL\_VS\_POLYIC\_STIM\_DC\_1H\_DN |  | 29 | -0.30 | -1.58 | 0.044 | 0.215 | 1.000 | 151 | tags=31%, list=11%, signal=34% |
| 502 | GSE11961\_GERMINAL\_CENTER\_BCELL\_DAY7\_VS\_MEMORY\_BCELL\_DAY40\_DN |  | 24 | -0.32 | -1.58 | 0.069 | 0.216 | 1.000 | 263 | tags=46%, list=19%, signal=55% |
| 503 | GSE5503\_MLN\_DC\_VS\_PLN\_DC\_ACTIVATED\_ALLOGENIC\_TCELL\_UP |  | 16 | -0.37 | -1.58 | 0.044 | 0.215 | 1.000 | 315 | tags=56%, list=22%, signal=72% |
| 504 | GSE29618\_PRE\_VS\_DAY7\_POST\_LAIV\_FLU\_VACCINE\_MONOCYTE\_UP |  | 17 | -0.36 | -1.58 | 0.057 | 0.216 | 1.000 | 550 | tags=76%, list=39%, signal=124% |
| 505 | GSE43955\_TH0\_VS\_TGFB\_IL6\_TH17\_ACT\_CD4\_TCELL\_10H\_DN |  | 17 | -0.36 | -1.58 | 0.040 | 0.217 | 1.000 | 286 | tags=41%, list=20%, signal=51% |
| 506 | GSE11961\_MARGINAL\_ZONE\_BCELL\_VS\_GERMINAL\_CENTER\_BCELL\_DAY7\_UP |  | 23 | -0.33 | -1.58 | 0.059 | 0.217 | 1.000 | 216 | tags=39%, list=15%, signal=45% |
| 507 | GSE19198\_CTRL\_VS\_IL21\_TREATED\_TCELL\_6H\_DN |  | 25 | -0.32 | -1.58 | 0.050 | 0.216 | 1.000 | 251 | tags=40%, list=18%, signal=48% |
| 508 | GSE3982\_NEUTROPHIL\_VS\_TH1\_UP |  | 19 | -0.35 | -1.58 | 0.059 | 0.217 | 1.000 | 440 | tags=63%, list=31%, signal=91% |
| 509 | GSE3982\_DC\_VS\_BCELL\_UP |  | 27 | -0.31 | -1.58 | 0.029 | 0.217 | 1.000 | 613 | tags=78%, list=43%, signal=135% |
| 510 | GSE20366\_TREG\_VS\_NAIVE\_CD4\_TCELL\_HOMEOSTATIC\_CONVERSION\_UP |  | 21 | -0.34 | -1.58 | 0.052 | 0.219 | 1.000 | 161 | tags=29%, list=11%, signal=32% |
| 511 | GSE6259\_CD4\_TCELL\_VS\_CD8\_TCELL\_UP |  | 17 | -0.36 | -1.57 | 0.038 | 0.222 | 1.000 | 513 | tags=71%, list=36%, signal=110% |
| 512 | GSE28783\_ANTI\_MIR33\_VS\_UNTREATED\_ATHEROSCLEROSIS\_MACROPHAGE\_DN |  | 17 | -0.37 | -1.57 | 0.056 | 0.223 | 1.000 | 442 | tags=65%, list=31%, signal=93% |
| 513 | GSE3920\_IFNA\_VS\_IFNG\_TREATED\_FIBROBLAST\_DN |  | 15 | -0.37 | -1.57 | 0.041 | 0.226 | 1.000 | 199 | tags=40%, list=14%, signal=46% |
| 514 | GSE15930\_STIM\_VS\_STIM\_AND\_IFNAB\_72H\_CD8\_T\_CELL\_DN |  | 17 | -0.36 | -1.57 | 0.041 | 0.227 | 1.000 | 909 | tags=100%, list=64%, signal=278% |
| 515 | GSE26912\_TUMORICIDAL\_VS\_CTRL\_MACROPHAGE\_UP |  | 17 | -0.36 | -1.56 | 0.046 | 0.230 | 1.000 | 221 | tags=41%, list=16%, signal=48% |
| 516 | GSE1925\_CTRL\_VS\_IFNG\_PRIMED\_MACROPHAGE\_24H\_IFNG\_STIM\_DN |  | 19 | -0.35 | -1.56 | 0.043 | 0.230 | 1.000 | 533 | tags=74%, list=38%, signal=117% |
| 517 | GSE43260\_BTLA\_POS\_VS\_NEG\_INTRATUMORAL\_CD8\_TCELL\_DN |  | 21 | -0.33 | -1.56 | 0.041 | 0.230 | 1.000 | 788 | tags=90%, list=56%, signal=202% |
| 518 | GSE42021\_TREG\_PLN\_VS\_CD24HI\_TREG\_THYMUS\_UP |  | 39 | -0.27 | -1.56 | 0.042 | 0.230 | 1.000 | 563 | tags=69%, list=40%, signal=112% |
| 519 | GSE5679\_CTRL\_VS\_PPARG\_LIGAND\_ROSIGLITAZONE\_TREATED\_DC\_DN |  | 22 | -0.33 | -1.56 | 0.042 | 0.230 | 1.000 | 289 | tags=45%, list=20%, signal=56% |
| 520 | GSE27786\_CD4\_TCELL\_VS\_NKTCELL\_UP |  | 23 | -0.33 | -1.56 | 0.049 | 0.230 | 1.000 | 380 | tags=52%, list=27%, signal=70% |
| 521 | GSE7509\_UNSTIM\_VS\_IFNA\_STIM\_IMMATURE\_DC\_DN |  | 18 | -0.36 | -1.56 | 0.050 | 0.232 | 1.000 | 310 | tags=56%, list=22%, signal=70% |
| 522 | GSE15930\_NAIVE\_VS\_24H\_IN\_VITRO\_STIM\_IL12\_CD8\_TCELL\_UP |  | 16 | -0.38 | -1.56 | 0.050 | 0.231 | 1.000 | 440 | tags=63%, list=31%, signal=90% |
| 523 | GSE19888\_CTRL\_VS\_TCELL\_MEMBRANES\_ACT\_MAST\_CELL\_PRETREAT\_A3R\_INH\_UP |  | 15 | -0.39 | -1.56 | 0.058 | 0.231 | 1.000 | 339 | tags=53%, list=24%, signal=69% |
| 524 | GSE36527\_CD62L\_HIGH\_VS\_CD62L\_LOW\_TREG\_CD69\_NEG\_KLRG1\_NEG\_UP |  | 16 | -0.39 | -1.56 | 0.059 | 0.231 | 1.000 | 122 | tags=31%, list=9%, signal=34% |
| 525 | GSE10239\_NAIVE\_VS\_KLRG1HIGH\_EFF\_CD8\_TCELL\_DN |  | 18 | -0.35 | -1.56 | 0.032 | 0.231 | 1.000 | 353 | tags=50%, list=25%, signal=66% |
| 526 | GSE20151\_CTRL\_VS\_FUSOBACT\_NUCLEATUM\_NEUTROPHIL\_DN |  | 20 | -0.34 | -1.56 | 0.047 | 0.231 | 1.000 | 222 | tags=35%, list=16%, signal=41% |
| 527 | GSE360\_CTRL\_VS\_T\_GONDII\_DC\_DN |  | 17 | -0.36 | -1.56 | 0.051 | 0.231 | 1.000 | 562 | tags=82%, list=40%, signal=135% |
| 528 | GSE3039\_ALPHABETA\_CD8\_TCELL\_VS\_B1\_BCELL\_UP |  | 22 | -0.33 | -1.56 | 0.040 | 0.231 | 1.000 | 284 | tags=50%, list=20%, signal=62% |
| 529 | GSE29618\_BCELL\_VS\_MONOCYTE\_DAY7\_FLU\_VACCINE\_UP |  | 19 | -0.35 | -1.56 | 0.054 | 0.230 | 1.000 | 242 | tags=42%, list=17%, signal=50% |
| 530 | GSE20152\_SPHK1\_KO\_VS\_WT\_HTNFA\_OVERXPRESS\_ANKLE\_UP |  | 21 | -0.33 | -1.56 | 0.061 | 0.230 | 1.000 | 534 | tags=71%, list=38%, signal=113% |
| 531 | GSE2770\_IL4\_ACT\_VS\_ACT\_CD4\_TCELL\_6H\_UP |  | 21 | -0.33 | -1.56 | 0.046 | 0.231 | 1.000 | 544 | tags=71%, list=39%, signal=115% |
| 532 | GSE26488\_WT\_VS\_VP16\_TRANSGENIC\_HDAC7\_KO\_DOUBLE\_POSITIVE\_THYMOCYTE\_DN |  | 23 | -0.32 | -1.56 | 0.036 | 0.231 | 1.000 | 395 | tags=52%, list=28%, signal=71% |
| 533 | GSE9601\_UNTREATED\_VS\_NFKB\_INHIBITOR\_TREATED\_HCMV\_INF\_MONOCYTE\_DN |  | 16 | -0.37 | -1.56 | 0.057 | 0.232 | 1.000 | 472 | tags=69%, list=33%, signal=102% |
| 534 | GSE17721\_LPS\_VS\_GARDIQUIMOD\_16H\_BMDC\_UP |  | 20 | -0.34 | -1.55 | 0.046 | 0.233 | 1.000 | 945 | tags=100%, list=67%, signal=298% |
| 535 | GSE43863\_NAIVE\_VS\_TH1\_EFF\_CD4\_TCELL\_D6\_LCMV\_DN |  | 17 | -0.36 | -1.55 | 0.060 | 0.233 | 1.000 | 390 | tags=59%, list=28%, signal=80% |
| 536 | GSE24574\_BCL6\_HIGH\_TFH\_VS\_TFH\_CD4\_TCELL\_DN |  | 20 | -0.34 | -1.55 | 0.056 | 0.234 | 1.000 | 167 | tags=35%, list=12%, signal=39% |
| 537 | GSE2128\_C57BL6\_VS\_NOD\_CD4CD8\_DP\_THYMOCYTE\_DN |  | 16 | -0.37 | -1.55 | 0.040 | 0.235 | 1.000 | 187 | tags=38%, list=13%, signal=43% |
| 538 | GSE411\_UNSTIM\_VS\_100MIN\_IL6\_STIM\_MACROPHAGE\_UP |  | 21 | -0.33 | -1.55 | 0.058 | 0.236 | 1.000 | 573 | tags=86%, list=41%, signal=142% |
| 539 | GSE3920\_UNTREATED\_VS\_IFNB\_TREATED\_ENDOTHELIAL\_CELL\_UP |  | 22 | -0.32 | -1.55 | 0.048 | 0.237 | 1.000 | 100 | tags=23%, list=7%, signal=24% |
| 540 | GSE6259\_33D1\_POS\_VS\_DEC205\_POS\_FLT3L\_INDUCED\_SPLENIC\_DC\_UP |  | 19 | -0.35 | -1.55 | 0.052 | 0.236 | 1.000 | 391 | tags=58%, list=28%, signal=79% |
| 541 | GSE32986\_UNSTIM\_VS\_CURDLAN\_LOWDOSE\_STIM\_DC\_DN |  | 27 | -0.30 | -1.55 | 0.061 | 0.237 | 1.000 | 588 | tags=74%, list=42%, signal=125% |
| 542 | GSE19198\_6H\_VS\_24H\_IL21\_TREATED\_TCELL\_DN |  | 25 | -0.31 | -1.55 | 0.047 | 0.236 | 1.000 | 251 | tags=40%, list=18%, signal=48% |
| 543 | GSE21546\_WT\_VS\_SAP1A\_KO\_AND\_ELK1\_KO\_ANTI\_CD3\_STIM\_DP\_THYMOCYTES\_DN |  | 20 | -0.33 | -1.55 | 0.042 | 0.237 | 1.000 | 341 | tags=55%, list=24%, signal=72% |
| 544 | GSE5503\_MLN\_DC\_VS\_SPLEEN\_DC\_ACTIVATED\_ALLOGENIC\_TCELL\_UP |  | 17 | -0.35 | -1.55 | 0.052 | 0.238 | 1.000 | 266 | tags=47%, list=19%, signal=57% |
| 545 | GSE7218\_IGM\_VS\_IGG\_SIGNAL\_THGOUGH\_ANTIGEN\_BCELL\_DN |  | 17 | -0.36 | -1.55 | 0.048 | 0.238 | 1.000 | 350 | tags=53%, list=25%, signal=70% |
| 546 | GSE1460\_INTRATHYMIC\_T\_PROGENITOR\_VS\_THYMIC\_STROMAL\_CELL\_DN |  | 19 | -0.34 | -1.54 | 0.053 | 0.238 | 1.000 | 484 | tags=68%, list=34%, signal=103% |
| 547 | GSE26290\_CTRL\_VS\_AKT\_INHIBITOR\_TREATED\_ANTI\_CD3\_AND\_IL2\_STIM\_CD8\_TCELL\_DN |  | 22 | -0.33 | -1.54 | 0.050 | 0.238 | 1.000 | 460 | tags=64%, list=33%, signal=93% |
| 548 | GSE24972\_MARGINAL\_ZONE\_BCELL\_VS\_FOLLICULAR\_BCELL\_IRF8\_KO\_UP |  | 22 | -0.33 | -1.54 | 0.061 | 0.241 | 1.000 | 579 | tags=77%, list=41%, signal=129% |
| 549 | GSE41176\_UNSTIM\_VS\_ANTI\_IGM\_STIM\_BCELL\_24H\_DN |  | 18 | -0.34 | -1.54 | 0.058 | 0.241 | 1.000 | 562 | tags=78%, list=40%, signal=128% |
| 550 | GSE39382\_IL3\_VS\_IL3\_IL33\_TREATED\_MAST\_CELL\_DN |  | 23 | -0.33 | -1.54 | 0.054 | 0.241 | 1.000 | 456 | tags=61%, list=32%, signal=88% |
| 551 | GSE14000\_4H\_VS\_16H\_LPS\_DC\_TRANSLATED\_RNA\_UP |  | 18 | -0.35 | -1.54 | 0.046 | 0.241 | 1.000 | 328 | tags=50%, list=23%, signal=64% |
| 552 | GSE41867\_NAIVE\_VS\_DAY6\_LCMV\_EFFECTOR\_CD8\_TCELL\_UP |  | 25 | -0.31 | -1.54 | 0.068 | 0.240 | 1.000 | 262 | tags=36%, list=19%, signal=43% |
| 553 | GSE29618\_MONOCYTE\_VS\_MDC\_DAY7\_FLU\_VACCINE\_DN |  | 17 | -0.37 | -1.54 | 0.059 | 0.242 | 1.000 | 469 | tags=65%, list=33%, signal=96% |
| 554 | GSE23502\_WT\_VS\_HDC\_KO\_MYELOID\_DERIVED\_SUPPRESSOR\_CELL\_BM\_UP |  | 19 | -0.34 | -1.54 | 0.057 | 0.242 | 1.000 | 125 | tags=32%, list=9%, signal=34% |
| 555 | GSE41176\_WT\_VS\_TAK1\_KO\_ANTI\_IGM\_STIM\_BCELL\_24H\_UP |  | 27 | -0.31 | -1.54 | 0.050 | 0.244 | 1.000 | 536 | tags=70%, list=38%, signal=111% |
| 556 | GSE360\_L\_DONOVANI\_VS\_T\_GONDII\_MAC\_DN |  | 17 | -0.36 | -1.54 | 0.039 | 0.244 | 1.000 | 914 | tags=100%, list=65%, signal=280% |
| 557 | GSE22501\_PERIPHERAL\_BLOOD\_VS\_CORD\_BLOOD\_TREG\_UP |  | 15 | -0.37 | -1.54 | 0.055 | 0.244 | 1.000 | 128 | tags=33%, list=9%, signal=36% |
| 558 | GSE5679\_CTRL\_VS\_RARA\_AGONIST\_AM580\_TREATED\_DC\_DN |  | 23 | -0.31 | -1.53 | 0.054 | 0.246 | 1.000 | 557 | tags=74%, list=39%, signal=120% |
| 559 | GSE11057\_NAIVE\_VS\_MEMORY\_CD4\_TCELL\_UP |  | 15 | -0.38 | -1.53 | 0.066 | 0.247 | 1.000 | 480 | tags=73%, list=34%, signal=110% |
| 560 | GSE17974\_0H\_VS\_4H\_IN\_VITRO\_ACT\_CD4\_TCELL\_UP |  | 16 | -0.37 | -1.53 | 0.065 | 0.247 | 1.000 | 499 | tags=69%, list=35%, signal=105% |
| 561 | GSE32164\_RESTING\_DIFFERENTIATED\_VS\_ALTERNATIVELY\_ACT\_M2\_MACROPHAGE\_DN |  | 20 | -0.33 | -1.53 | 0.051 | 0.247 | 1.000 | 572 | tags=75%, list=41%, signal=124% |
| 562 | GSE22432\_MULTIPOTENT\_PROGENITOR\_VS\_CDC\_UP |  | 16 | -0.37 | -1.53 | 0.072 | 0.247 | 1.000 | 333 | tags=50%, list=24%, signal=65% |
| 563 | GSE37605\_C57BL6\_VS\_NOD\_FOXP3\_FUSION\_GFP\_TREG\_DN |  | 22 | -0.33 | -1.53 | 0.069 | 0.247 | 1.000 | 561 | tags=77%, list=40%, signal=126% |
| 564 | GSE43955\_TH0\_VS\_TGFB\_IL6\_TH17\_ACT\_CD4\_TCELL\_30H\_DN |  | 15 | -0.36 | -1.53 | 0.045 | 0.247 | 1.000 | 456 | tags=67%, list=32%, signal=97% |
| 565 | GSE7831\_UNSTIM\_VS\_CPG\_STIM\_PDC\_4H\_DN |  | 16 | -0.36 | -1.53 | 0.070 | 0.247 | 1.000 | 483 | tags=69%, list=34%, signal=103% |
| 566 | GSE10325\_CD4\_TCELL\_VS\_BCELL\_UP |  | 26 | -0.30 | -1.53 | 0.060 | 0.247 | 1.000 | 311 | tags=42%, list=22%, signal=53% |
| 567 | GSE18281\_CORTICAL\_VS\_MEDULLARY\_THYMOCYTE\_UP |  | 29 | -0.30 | -1.53 | 0.064 | 0.247 | 1.000 | 557 | tags=72%, list=39%, signal=117% |
| 568 | GSE17974\_0H\_VS\_48H\_IN\_VITRO\_ACT\_CD4\_TCELL\_UP |  | 22 | -0.32 | -1.53 | 0.079 | 0.247 | 1.000 | 555 | tags=77%, list=39%, signal=125% |
| 569 | GSE3691\_IFN\_PRODUCING\_KILLER\_DC\_VS\_CONVENTIONAL\_DC\_SPLEEN\_UP |  | 23 | -0.32 | -1.53 | 0.069 | 0.247 | 1.000 | 504 | tags=70%, list=36%, signal=106% |
| 570 | GSE2585\_THYMIC\_MACROPHAGE\_VS\_MTEC\_DN |  | 24 | -0.31 | -1.53 | 0.051 | 0.247 | 1.000 | 315 | tags=46%, list=22%, signal=58% |
| 571 | GSE30962\_ACUTE\_VS\_CHRONIC\_LCMV\_PRIMARY\_INF\_CD8\_TCELL\_UP |  | 30 | -0.29 | -1.53 | 0.065 | 0.247 | 1.000 | 543 | tags=70%, list=38%, signal=111% |
| 572 | GSE9988\_ANTI\_TREM1\_VS\_ANTI\_TREM1\_AND\_LPS\_MONOCYTE\_DN |  | 24 | -0.31 | -1.53 | 0.061 | 0.247 | 1.000 | 547 | tags=71%, list=39%, signal=114% |
| 573 | GSE27786\_LSK\_VS\_NKTCELL\_DN |  | 20 | -0.34 | -1.53 | 0.055 | 0.246 | 1.000 | 398 | tags=55%, list=28%, signal=76% |
| 574 | GSE17721\_CTRL\_VS\_PAM3CSK4\_1H\_BMDC\_UP |  | 23 | -0.31 | -1.53 | 0.072 | 0.249 | 1.000 | 120 | tags=30%, list=9%, signal=33% |
| 575 | GSE3982\_BCELL\_VS\_TH1\_UP |  | 17 | -0.35 | -1.53 | 0.061 | 0.249 | 1.000 | 204 | tags=35%, list=14%, signal=41% |
| 576 | GSE7568\_CTRL\_VS\_24H\_TGFB\_TREATED\_MACROPHAGES\_WITH\_IL4\_AND\_DEXAMETHASONE\_UP |  | 17 | -0.35 | -1.52 | 0.082 | 0.249 | 1.000 | 218 | tags=35%, list=15%, signal=41% |
| 577 | GSE17301\_CTRL\_VS\_48H\_ACD3\_ACD28\_IFNA5\_STIM\_CD8\_TCELL\_UP |  | 24 | -0.31 | -1.52 | 0.062 | 0.248 | 1.000 | 492 | tags=67%, list=35%, signal=101% |
| 578 | GSE15930\_NAIVE\_VS\_48H\_IN\_VITRO\_STIM\_IFNAB\_CD8\_TCELL\_DN |  | 29 | -0.29 | -1.52 | 0.062 | 0.248 | 1.000 | 946 | tags=97%, list=67%, signal=287% |
| 579 | GSE19825\_CD24LOW\_VS\_IL2RA\_HIGH\_DAY3\_EFF\_CD8\_TCELL\_UP |  | 26 | -0.30 | -1.52 | 0.059 | 0.248 | 1.000 | 398 | tags=54%, list=28%, signal=74% |
| 580 | GSE24634\_TREG\_VS\_TCONV\_POST\_DAY10\_IL4\_CONVERSION\_UP |  | 29 | -0.30 | -1.52 | 0.077 | 0.248 | 1.000 | 575 | tags=69%, list=41%, signal=114% |
| 581 | GSE369\_PRE\_VS\_POST\_IL6\_INJECTION\_IFNG\_WT\_LIVER\_DN |  | 22 | -0.33 | -1.52 | 0.068 | 0.248 | 1.000 | 473 | tags=59%, list=34%, signal=88% |
| 582 | GSE22103\_LPS\_VS\_GMCSF\_AND\_IFNG\_STIM\_NEUTROPHIL\_UP |  | 28 | -0.29 | -1.52 | 0.053 | 0.248 | 1.000 | 114 | tags=25%, list=8%, signal=27% |
| 583 | GSE17721\_CPG\_VS\_GARDIQUIMOD\_4H\_BMDC\_UP |  | 17 | -0.36 | -1.52 | 0.057 | 0.248 | 1.000 | 362 | tags=53%, list=26%, signal=70% |
| 584 | GSE7548\_DAY7\_VS\_DAY28\_PCC\_IMMUNIZATION\_CD4\_TCELL\_UP |  | 19 | -0.34 | -1.52 | 0.049 | 0.248 | 1.000 | 471 | tags=68%, list=33%, signal=101% |
| 585 | GSE17721\_LPS\_VS\_POLYIC\_0.5H\_BMDC\_UP |  | 16 | -0.35 | -1.52 | 0.068 | 0.248 | 1.000 | 754 | tags=94%, list=53%, signal=199% |
| 586 | GSE35825\_IFNA\_VS\_IFNG\_STIM\_MACROPHAGE\_DN |  | 16 | -0.37 | -1.52 | 0.053 | 0.248 | 1.000 | 270 | tags=50%, list=19%, signal=61% |
| 587 | GSE5589\_IL6\_KO\_VS\_IL10\_KO\_LPS\_STIM\_MACROPHAGE\_180MIN\_UP |  | 15 | -0.37 | -1.52 | 0.050 | 0.248 | 1.000 | 69 | tags=27%, list=5%, signal=28% |
| 588 | GSE360\_DC\_VS\_MAC\_M\_TUBERCULOSIS\_DN |  | 25 | -0.31 | -1.52 | 0.039 | 0.248 | 1.000 | 218 | tags=32%, list=15%, signal=37% |
| 589 | GSE42021\_CD24INT\_VS\_CD24LOW\_TCONV\_THYMUS\_UP |  | 16 | -0.36 | -1.52 | 0.075 | 0.248 | 1.000 | 550 | tags=75%, list=39%, signal=122% |
| 590 | GSE29618\_PRE\_VS\_DAY7\_POST\_TIV\_FLU\_VACCINE\_MDC\_DN |  | 24 | -0.31 | -1.52 | 0.058 | 0.249 | 1.000 | 172 | tags=33%, list=12%, signal=37% |
| 591 | GSE22140\_GERMFREE\_VS\_SPF\_ARTHRITIC\_MOUSE\_CD4\_TCELL\_UP |  | 23 | -0.31 | -1.52 | 0.057 | 0.250 | 1.000 | 189 | tags=30%, list=13%, signal=35% |
| 592 | GSE17301\_CTRL\_VS\_48H\_IFNA2\_STIM\_CD8\_TCELL\_DN |  | 15 | -0.37 | -1.52 | 0.061 | 0.250 | 1.000 | 378 | tags=60%, list=27%, signal=81% |
| 593 | GSE22196\_HEALTHY\_VS\_OBESE\_MOUSE\_SKIN\_GAMMADELTA\_TCELL\_DN |  | 27 | -0.29 | -1.52 | 0.068 | 0.251 | 1.000 | 608 | tags=74%, list=43%, signal=128% |
| 594 | GSE41867\_NAIVE\_VS\_DAY8\_LCMV\_EFFECTOR\_CD8\_TCELL\_DN |  | 16 | -0.36 | -1.52 | 0.067 | 0.250 | 1.000 | 548 | tags=75%, list=39%, signal=121% |
| 595 | GSE33425\_CD161\_HIGH\_VS\_INT\_CD8\_TCELL\_UP |  | 23 | -0.31 | -1.52 | 0.051 | 0.251 | 1.000 | 108 | tags=26%, list=8%, signal=28% |
| 596 | GSE43863\_TFH\_VS\_LY6C\_INT\_CXCR5POS\_MEMORY\_CD4\_TCELL\_UP |  | 23 | -0.31 | -1.52 | 0.061 | 0.250 | 1.000 | 189 | tags=35%, list=13%, signal=40% |
| 597 | GSE22140\_HEALTHY\_VS\_ARTHRITIC\_GERMFREE\_MOUSE\_CD4\_TCELL\_DN |  | 22 | -0.32 | -1.52 | 0.053 | 0.250 | 1.000 | 301 | tags=41%, list=21%, signal=51% |
| 598 | GSE6259\_FLT3L\_INDUCED\_33D1\_POS\_DC\_VS\_CD8\_TCELL\_UP |  | 20 | -0.33 | -1.52 | 0.073 | 0.250 | 1.000 | 300 | tags=45%, list=21%, signal=56% |
| 599 | GSE13547\_WT\_VS\_ZFX\_KO\_BCELL\_ANTI\_IGM\_STIM\_12H\_DN |  | 17 | -0.35 | -1.51 | 0.057 | 0.252 | 1.000 | 322 | tags=53%, list=23%, signal=68% |
| 600 | GSE32255\_WT\_UNSTIM\_VS\_JMJD2D\_KNOCKDOWN\_4H\_LPS\_STIM\_DC\_DN |  | 25 | -0.31 | -1.51 | 0.071 | 0.251 | 1.000 | 316 | tags=44%, list=22%, signal=56% |
| 601 | GSE39110\_UNTREATED\_VS\_IL2\_TREATED\_CD8\_TCELL\_DAY6\_POST\_IMMUNIZATION\_UP |  | 20 | -0.33 | -1.51 | 0.052 | 0.251 | 1.000 | 572 | tags=80%, list=41%, signal=133% |
| 602 | GSE5542\_UNTREATED\_VS\_IFNG\_TREATED\_EPITHELIAL\_CELLS\_6H\_DN |  | 27 | -0.30 | -1.51 | 0.070 | 0.251 | 1.000 | 499 | tags=63%, list=35%, signal=96% |
| 603 | GSE35435\_RESTING\_VS\_IL4\_TREATED\_MACROPHAGE\_DN |  | 20 | -0.33 | -1.51 | 0.059 | 0.251 | 1.000 | 440 | tags=60%, list=31%, signal=86% |
| 604 | GSE3039\_NKT\_CELL\_VS\_B1\_BCELL\_DN |  | 20 | -0.33 | -1.51 | 0.088 | 0.252 | 1.000 | 159 | tags=30%, list=11%, signal=33% |
| 605 | GSE3982\_DC\_VS\_NEUTROPHIL\_DN |  | 27 | -0.30 | -1.51 | 0.061 | 0.251 | 1.000 | 256 | tags=37%, list=18%, signal=44% |
| 606 | GSE3039\_CD4\_TCELL\_VS\_NKT\_CELL\_UP |  | 26 | -0.30 | -1.51 | 0.062 | 0.252 | 1.000 | 492 | tags=65%, list=35%, signal=99% |
| 607 | GSE43955\_1H\_VS\_42H\_ACT\_CD4\_TCELL\_WITH\_TGFB\_IL6\_DN |  | 16 | -0.36 | -1.51 | 0.082 | 0.251 | 1.000 | 57 | tags=25%, list=4%, signal=26% |
| 608 | GSE20198\_IL12\_VS\_IFNA\_TREATED\_ACT\_CD4\_TCELL\_UP |  | 22 | -0.31 | -1.51 | 0.063 | 0.252 | 1.000 | 216 | tags=32%, list=15%, signal=37% |
| 609 | GSE22025\_UNTREATED\_VS\_PROGESTERONE\_TREATED\_CD4\_TCELL\_UP |  | 16 | -0.35 | -1.51 | 0.069 | 0.252 | 1.000 | 353 | tags=50%, list=25%, signal=66% |
| 610 | GSE24081\_CONTROLLER\_VS\_PROGRESSOR\_HIV\_SPECIFIC\_CD8\_TCELL\_UP |  | 23 | -0.32 | -1.51 | 0.068 | 0.252 | 1.000 | 522 | tags=70%, list=37%, signal=109% |
| 611 | GSE21546\_WT\_VS\_SAP1A\_KO\_AND\_ELK1\_KO\_ANTI\_CD3\_STIM\_DP\_THYMOCYTES\_UP |  | 24 | -0.31 | -1.51 | 0.054 | 0.252 | 1.000 | 523 | tags=71%, list=37%, signal=111% |
| 612 | GSE32128\_INOS\_DEPENDENT\_VS\_INOS\_INDEPENDENT\_ACTIVATED\_TCELL\_UP |  | 20 | -0.33 | -1.51 | 0.058 | 0.254 | 1.000 | 557 | tags=75%, list=39%, signal=122% |
| 613 | GSE19941\_UNSTIM\_VS\_LPS\_AND\_IL10\_STIM\_IL10\_KO\_MACROPHAGE\_UP |  | 18 | -0.35 | -1.51 | 0.073 | 0.253 | 1.000 | 499 | tags=67%, list=35%, signal=102% |
| 614 | GSE11057\_NAIVE\_VS\_EFF\_MEMORY\_CD4\_TCELL\_UP |  | 25 | -0.31 | -1.51 | 0.060 | 0.254 | 1.000 | 532 | tags=72%, list=38%, signal=114% |
| 615 | GSE24972\_MARGINAL\_ZONE\_BCELL\_VS\_FOLLICULAR\_BCELL\_IRF8\_KO\_DN |  | 24 | -0.30 | -1.51 | 0.056 | 0.255 | 1.000 | 295 | tags=42%, list=21%, signal=52% |
| 616 | GSE21063\_CTRL\_VS\_ANTI\_IGM\_STIM\_BCELL\_NFATC1\_KO\_16H\_UP |  | 18 | -0.34 | -1.51 | 0.073 | 0.255 | 1.000 | 566 | tags=72%, list=40%, signal=119% |
| 617 | GSE17721\_0.5H\_VS\_12H\_PAM3CSK4\_BMDC\_UP |  | 17 | -0.34 | -1.51 | 0.053 | 0.255 | 1.000 | 177 | tags=35%, list=13%, signal=40% |
| 618 | GSE7831\_UNSTIM\_VS\_CPG\_STIM\_PDC\_1H\_UP |  | 17 | -0.34 | -1.50 | 0.055 | 0.257 | 1.000 | 472 | tags=65%, list=33%, signal=96% |
| 619 | GSE6259\_BCELL\_VS\_CD8\_TCELL\_UP |  | 27 | -0.30 | -1.50 | 0.068 | 0.257 | 1.000 | 467 | tags=63%, list=33%, signal=92% |
| 620 | GSE360\_T\_GONDII\_VS\_B\_MALAYI\_HIGH\_DOSE\_MAC\_UP |  | 24 | -0.31 | -1.50 | 0.066 | 0.259 | 1.000 | 254 | tags=33%, list=18%, signal=40% |
| 621 | GSE17721\_0.5H\_VS\_8H\_CPG\_BMDC\_UP |  | 15 | -0.36 | -1.50 | 0.058 | 0.258 | 1.000 | 171 | tags=40%, list=12%, signal=45% |
| 622 | GSE20500\_RETINOIC\_ACID\_VS\_RARA\_ANTAGONIST\_TREATED\_CD4\_TCELL\_DN |  | 27 | -0.29 | -1.50 | 0.060 | 0.259 | 1.000 | 502 | tags=63%, list=36%, signal=96% |
| 623 | GSE40068\_CXCR5POS\_BCL6POS\_TFH\_VS\_CXCR5NEG\_BCL6NEG\_CD4\_TCELL\_DN |  | 20 | -0.33 | -1.50 | 0.064 | 0.259 | 1.000 | 460 | tags=65%, list=33%, signal=95% |
| 624 | GSE14908\_ATOPIC\_VS\_NONATOPIC\_PATIENT\_RESTING\_CD4\_TCELL\_UP |  | 17 | -0.35 | -1.50 | 0.077 | 0.259 | 1.000 | 558 | tags=76%, list=40%, signal=125% |
| 625 | GSE21670\_UNTREATED\_VS\_TGFB\_TREATED\_STAT3\_KO\_CD4\_TCELL\_DN |  | 27 | -0.29 | -1.50 | 0.068 | 0.259 | 1.000 | 300 | tags=41%, list=21%, signal=51% |
| 626 | GSE9037\_CTRL\_VS\_LPS\_4H\_STIM\_BMDM\_UP |  | 17 | -0.35 | -1.50 | 0.069 | 0.260 | 1.000 | 430 | tags=59%, list=30%, signal=84% |
| 627 | GSE40274\_CTRL\_VS\_IRF4\_TRANSDUCED\_ACTIVATED\_CD4\_TCELL\_DN |  | 20 | -0.34 | -1.50 | 0.091 | 0.261 | 1.000 | 447 | tags=65%, list=32%, signal=94% |
| 628 | GSE15930\_NAIVE\_VS\_72H\_IN\_VITRO\_STIM\_TRICHOSTATINA\_CD8\_TCELL\_UP |  | 21 | -0.32 | -1.50 | 0.062 | 0.261 | 1.000 | 420 | tags=62%, list=30%, signal=87% |
| 629 | GSE17301\_CTRL\_VS\_48H\_ACD3\_ACD28\_IFNA5\_STIM\_CD8\_TCELL\_DN |  | 24 | -0.30 | -1.50 | 0.089 | 0.262 | 1.000 | 492 | tags=63%, list=35%, signal=94% |
| 630 | GSE17721\_LPS\_VS\_POLYIC\_6H\_BMDC\_DN |  | 19 | -0.34 | -1.49 | 0.057 | 0.264 | 1.000 | 464 | tags=63%, list=33%, signal=93% |
| 631 | GSE20366\_EX\_VIVO\_VS\_DEC205\_CONVERSION\_NAIVE\_CD4\_TCELL\_DN |  | 23 | -0.30 | -1.49 | 0.082 | 0.264 | 1.000 | 445 | tags=61%, list=32%, signal=87% |
| 632 | GSE2770\_TGFB\_AND\_IL4\_VS\_IL12\_TREATED\_ACT\_CD4\_TCELL\_48H\_DN |  | 23 | -0.31 | -1.49 | 0.078 | 0.264 | 1.000 | 357 | tags=52%, list=25%, signal=69% |
| 633 | GSE43700\_UNTREATED\_VS\_IL10\_TREATED\_PBMC\_DN |  | 21 | -0.33 | -1.49 | 0.063 | 0.265 | 1.000 | 435 | tags=62%, list=31%, signal=88% |
| 634 | GSE3920\_IFNA\_VS\_IFNG\_TREATED\_ENDOTHELIAL\_CELL\_UP |  | 26 | -0.29 | -1.49 | 0.077 | 0.268 | 1.000 | 318 | tags=46%, list=23%, signal=58% |
| 635 | GSE26727\_WT\_VS\_KLF2\_KO\_LPS\_STIM\_MACROPHAGE\_UP |  | 22 | -0.31 | -1.49 | 0.076 | 0.268 | 1.000 | 326 | tags=50%, list=23%, signal=64% |
| 636 | GSE360\_CTRL\_VS\_L\_DONOVANI\_MAC\_DN |  | 21 | -0.33 | -1.49 | 0.054 | 0.268 | 1.000 | 486 | tags=62%, list=34%, signal=93% |
| 637 | GSE22886\_NAIVE\_CD4\_TCELL\_VS\_MONOCYTE\_DN |  | 16 | -0.36 | -1.49 | 0.057 | 0.267 | 1.000 | 276 | tags=44%, list=20%, signal=54% |
| 638 | GSE24634\_TEFF\_VS\_TCONV\_DAY5\_IN\_CULTURE\_DN |  | 18 | -0.33 | -1.49 | 0.064 | 0.268 | 1.000 | 543 | tags=72%, list=38%, signal=116% |
| 639 | GSE17580\_TREG\_VS\_TEFF\_S\_MANSONI\_INF\_UP |  | 25 | -0.30 | -1.49 | 0.081 | 0.269 | 1.000 | 572 | tags=76%, list=41%, signal=126% |
| 640 | GSE12366\_PLASMA\_CELL\_VS\_NAIVE\_BCELL\_UP |  | 17 | -0.35 | -1.49 | 0.082 | 0.269 | 1.000 | 385 | tags=59%, list=27%, signal=80% |
| 641 | GSE17721\_LPS\_VS\_CPG\_12H\_BMDC\_DN |  | 25 | -0.29 | -1.49 | 0.056 | 0.269 | 1.000 | 184 | tags=32%, list=13%, signal=36% |
| 642 | GSE2770\_UNTREATED\_VS\_ACT\_CD4\_TCELL\_2H\_UP |  | 19 | -0.32 | -1.49 | 0.060 | 0.269 | 1.000 | 550 | tags=74%, list=39%, signal=119% |
| 643 | GSE22886\_DAY0\_VS\_DAY7\_MONOCYTE\_IN\_CULTURE\_DN |  | 20 | -0.33 | -1.49 | 0.075 | 0.269 | 1.000 | 226 | tags=35%, list=16%, signal=41% |
| 644 | GSE2706\_UNSTIM\_VS\_2H\_LPS\_AND\_R848\_DC\_DN |  | 29 | -0.28 | -1.48 | 0.061 | 0.269 | 1.000 | 547 | tags=69%, list=39%, signal=110% |
| 645 | GSE3982\_DC\_VS\_NKCELL\_UP |  | 20 | -0.33 | -1.48 | 0.081 | 0.269 | 1.000 | 415 | tags=60%, list=29%, signal=84% |
| 646 | GSE37416\_0H\_VS\_12H\_F\_TULARENSIS\_LVS\_NEUTROPHIL\_UP |  | 17 | -0.35 | -1.48 | 0.083 | 0.269 | 1.000 | 437 | tags=59%, list=31%, signal=84% |
| 647 | GSE20715\_0H\_VS\_48H\_OZONE\_TLR4\_KO\_LUNG\_UP |  | 16 | -0.36 | -1.48 | 0.086 | 0.268 | 1.000 | 435 | tags=63%, list=31%, signal=89% |
| 648 | GSE40666\_UNTREATED\_VS\_IFNA\_STIM\_STAT4\_KO\_EFFECTOR\_CD8\_TCELL\_90MIN\_UP |  | 21 | -0.32 | -1.48 | 0.071 | 0.269 | 1.000 | 549 | tags=71%, list=39%, signal=115% |
| 649 | GSE36888\_UNTREATED\_VS\_IL2\_TREATED\_TCELL\_2H\_UP |  | 20 | -0.33 | -1.48 | 0.068 | 0.269 | 1.000 | 251 | tags=40%, list=18%, signal=48% |
| 650 | GSE29617\_CTRL\_VS\_DAY3\_TIV\_FLU\_VACCINE\_PBMC\_2008\_UP |  | 18 | -0.35 | -1.48 | 0.083 | 0.271 | 1.000 | 539 | tags=72%, list=38%, signal=115% |
| 651 | GSE41176\_WT\_VS\_TAK1\_KO\_ANTI\_IGM\_STIM\_BCELL\_6H\_DN |  | 21 | -0.32 | -1.48 | 0.077 | 0.274 | 1.000 | 787 | tags=90%, list=56%, signal=202% |
| 652 | GSE29949\_MICROGLIA\_VS\_DC\_BRAIN\_UP |  | 25 | -0.29 | -1.48 | 0.068 | 0.274 | 1.000 | 548 | tags=72%, list=39%, signal=116% |
| 653 | GSE3982\_NEUTROPHIL\_VS\_TH2\_UP |  | 18 | -0.34 | -1.48 | 0.080 | 0.273 | 1.000 | 525 | tags=72%, list=37%, signal=114% |
| 654 | GSE30971\_WBP7\_HET\_VS\_KO\_MACROPHAGE\_DN |  | 25 | -0.29 | -1.48 | 0.079 | 0.273 | 1.000 | 204 | tags=32%, list=14%, signal=37% |
| 655 | GSE26669\_CD4\_VS\_CD8\_TCELL\_IN\_MLR\_COSTIM\_BLOCK\_UP |  | 17 | -0.35 | -1.48 | 0.073 | 0.273 | 1.000 | 278 | tags=47%, list=20%, signal=58% |
| 656 | GSE2706\_UNSTIM\_VS\_8H\_LPS\_AND\_R848\_DC\_DN |  | 22 | -0.31 | -1.48 | 0.072 | 0.274 | 1.000 | 562 | tags=73%, list=40%, signal=119% |
| 657 | GSE3982\_MAST\_CELL\_VS\_BASOPHIL\_UP |  | 26 | -0.29 | -1.47 | 0.052 | 0.276 | 1.000 | 252 | tags=35%, list=18%, signal=41% |
| 658 | GSE9006\_TYPE\_1\_DIABETES\_AT\_DX\_VS\_4MONTH\_POST\_DX\_PBMC\_UP |  | 30 | -0.28 | -1.47 | 0.062 | 0.276 | 1.000 | 576 | tags=70%, list=41%, signal=116% |
| 659 | GSE24634\_TEFF\_VS\_TCONV\_DAY10\_IN\_CULTURE\_DN |  | 19 | -0.34 | -1.47 | 0.083 | 0.275 | 1.000 | 233 | tags=42%, list=17%, signal=50% |
| 660 | GSE23114\_PERITONEAL\_CAVITY\_B1A\_BCELL\_VS\_SPLEEN\_BCELL\_DN |  | 32 | -0.27 | -1.47 | 0.071 | 0.277 | 1.000 | 915 | tags=94%, list=65%, signal=261% |
| 661 | GSE1925\_CTRL\_VS\_IFNG\_PRIMED\_MACROPHAGE\_UP |  | 21 | -0.31 | -1.47 | 0.066 | 0.276 | 1.000 | 709 | tags=86%, list=50%, signal=170% |
| 662 | GSE17301\_IFNA2\_VS\_IFNA2\_AND\_ACD3\_ACD28\_STIM\_CD8\_TCELL\_UP |  | 24 | -0.30 | -1.47 | 0.073 | 0.276 | 1.000 | 378 | tags=54%, list=27%, signal=73% |
| 663 | GSE15330\_HSC\_VS\_GRANULOCYTE\_MONOCYTE\_PROGENITOR\_IKAROS\_KO\_DN |  | 15 | -0.36 | -1.47 | 0.087 | 0.277 | 1.000 | 133 | tags=27%, list=9%, signal=29% |
| 664 | GSE17301\_CTRL\_VS\_48H\_ACD3\_ACD28\_STIM\_CD8\_TCELL\_DN |  | 19 | -0.33 | -1.47 | 0.070 | 0.277 | 1.000 | 421 | tags=63%, list=30%, signal=89% |
| 665 | GSE21670\_TGFB\_VS\_TGFB\_AND\_IL6\_TREATED\_STAT3\_KO\_CD4\_TCELL\_DN |  | 23 | -0.30 | -1.47 | 0.062 | 0.277 | 1.000 | 197 | tags=30%, list=14%, signal=35% |
| 666 | GSE39820\_CTRL\_VS\_TGFBETA1\_IL6\_CD4\_TCELL\_UP |  | 24 | -0.30 | -1.47 | 0.098 | 0.277 | 1.000 | 305 | tags=42%, list=22%, signal=52% |
| 667 | GSE27786\_CD4\_VS\_CD8\_TCELL\_UP |  | 29 | -0.28 | -1.47 | 0.067 | 0.277 | 1.000 | 362 | tags=48%, list=26%, signal=64% |
| 668 | GSE13738\_RESTING\_VS\_BYSTANDER\_ACTIVATED\_CD4\_TCELL\_UP |  | 17 | -0.35 | -1.47 | 0.091 | 0.277 | 1.000 | 249 | tags=47%, list=18%, signal=56% |
| 669 | GSE14415\_ACT\_TCONV\_VS\_ACT\_NATURAL\_TREG\_DN |  | 26 | -0.30 | -1.47 | 0.067 | 0.277 | 1.000 | 554 | tags=69%, list=39%, signal=112% |
| 670 | GSE46606\_IRF4\_KO\_VS\_WT\_CD40L\_IL2\_IL5\_3DAY\_STIMULATED\_BCELL\_UP |  | 22 | -0.31 | -1.47 | 0.064 | 0.277 | 1.000 | 331 | tags=45%, list=23%, signal=58% |
| 671 | GSE22103\_LPS\_VS\_GMCSF\_AND\_IFNG\_STIM\_NEUTROPHIL\_DN |  | 27 | -0.29 | -1.47 | 0.081 | 0.277 | 1.000 | 331 | tags=44%, list=23%, signal=57% |
| 672 | GSE3982\_BASOPHIL\_VS\_TH1\_UP |  | 28 | -0.28 | -1.47 | 0.080 | 0.279 | 1.000 | 416 | tags=50%, list=29%, signal=69% |
| 673 | GSE29618\_BCELL\_VS\_MONOCYTE\_DAY7\_FLU\_VACCINE\_DN |  | 19 | -0.33 | -1.47 | 0.062 | 0.280 | 1.000 | 499 | tags=68%, list=35%, signal=104% |
| 674 | GSE11973\_MIR223\_KOVS\_WT\_BONE\_MARROW\_NEUTROPHIL\_UP |  | 16 | -0.35 | -1.47 | 0.066 | 0.279 | 1.000 | 722 | tags=94%, list=51%, signal=190% |
| 675 | GSE2770\_IL12\_AND\_TGFB\_ACT\_VS\_ACT\_CD4\_TCELL\_6H\_UP |  | 18 | -0.34 | -1.46 | 0.087 | 0.280 | 1.000 | 314 | tags=50%, list=22%, signal=63% |
| 676 | GSE46606\_IRF4HIGH\_VS\_IRF4MID\_CD40L\_IL2\_IL5\_DAY1\_STIMULATED\_BCELL\_UP |  | 20 | -0.32 | -1.46 | 0.086 | 0.281 | 1.000 | 270 | tags=40%, list=19%, signal=49% |
| 677 | GSE15750\_DAY6\_VS\_DAY10\_TRAF6KO\_EFF\_CD8\_TCELL\_DN |  | 24 | -0.29 | -1.46 | 0.068 | 0.281 | 1.000 | 551 | tags=71%, list=39%, signal=114% |
| 678 | GSE15330\_MEGAKARYOCYTE\_ERYTHROID\_VS\_GRANULOCYTE\_MONOCYTE\_PROGENITOR\_DN |  | 15 | -0.35 | -1.46 | 0.075 | 0.281 | 1.000 | 354 | tags=60%, list=25%, signal=79% |
| 679 | GSE42021\_CD24LO\_TREG\_VS\_CD24LO\_TCONV\_THYMUS\_UP |  | 18 | -0.33 | -1.46 | 0.065 | 0.282 | 1.000 | 57 | tags=22%, list=4%, signal=23% |
| 680 | GSE23505\_UNTREATED\_VS\_4DAY\_IL6\_IL1\_IL23\_TREATED\_CD4\_TCELL\_UP |  | 29 | -0.27 | -1.46 | 0.081 | 0.281 | 1.000 | 217 | tags=34%, list=15%, signal=40% |
| 681 | GSE40277\_GATA1\_AND\_SATB1\_TRANSDUCED\_VS\_CTRL\_CD4\_TCELL\_DN |  | 23 | -0.30 | -1.46 | 0.081 | 0.281 | 1.000 | 748 | tags=83%, list=53%, signal=173% |
| 682 | GSE15330\_HSC\_VS\_LYMPHOID\_PRIMED\_MULTIPOTENT\_PROGENITOR\_UP |  | 24 | -0.30 | -1.46 | 0.086 | 0.282 | 1.000 | 538 | tags=71%, list=38%, signal=113% |
| 683 | GSE37301\_LYMPHOID\_PRIMED\_MPP\_VS\_PRO\_BCELL\_UP |  | 18 | -0.34 | -1.46 | 0.094 | 0.284 | 1.000 | 532 | tags=67%, list=38%, signal=106% |
| 684 | GSE7852\_LN\_VS\_THYMUS\_TREG\_DN |  | 19 | -0.32 | -1.46 | 0.085 | 0.283 | 1.000 | 335 | tags=47%, list=24%, signal=61% |
| 685 | GSE27896\_HDAC6\_KO\_VS\_WT\_TREG\_UP |  | 17 | -0.34 | -1.46 | 0.106 | 0.284 | 1.000 | 220 | tags=41%, list=16%, signal=48% |
| 686 | GSE22140\_HEALTHY\_VS\_ARTHRITIC\_MOUSE\_CD4\_TCELL\_DN |  | 27 | -0.29 | -1.46 | 0.079 | 0.284 | 1.000 | 243 | tags=33%, list=17%, signal=39% |
| 687 | GSE12392\_CD8A\_POS\_VS\_NEG\_SPLEEN\_DC\_DN |  | 20 | -0.31 | -1.46 | 0.087 | 0.285 | 1.000 | 290 | tags=45%, list=21%, signal=56% |
| 688 | GSE3920\_UNTREATED\_VS\_IFNG\_TREATED\_FIBROBLAST\_DN |  | 26 | -0.28 | -1.46 | 0.075 | 0.285 | 1.000 | 526 | tags=69%, list=37%, signal=108% |
| 689 | GSE7831\_UNSTIM\_VS\_CPG\_STIM\_PDC\_4H\_UP |  | 25 | -0.29 | -1.46 | 0.083 | 0.285 | 1.000 | 171 | tags=24%, list=12%, signal=27% |
| 690 | GSE45365\_NK\_CELL\_VS\_BCELL\_UP |  | 33 | -0.27 | -1.46 | 0.087 | 0.285 | 1.000 | 818 | tags=88%, list=58%, signal=204% |
| 691 | GSE23568\_CTRL\_TRANSDUCED\_VS\_WT\_CD8\_TCELL\_UP |  | 20 | -0.32 | -1.46 | 0.085 | 0.285 | 1.000 | 505 | tags=70%, list=36%, signal=107% |
| 692 | GSE21379\_WT\_VS\_SAP\_KO\_TFH\_CD4\_TCELL\_DN |  | 17 | -0.33 | -1.46 | 0.092 | 0.284 | 1.000 | 225 | tags=41%, list=16%, signal=48% |
| 693 | GSE7460\_CTRL\_VS\_TGFB\_TREATED\_ACT\_TCONV\_UP |  | 22 | -0.32 | -1.46 | 0.095 | 0.285 | 1.000 | 504 | tags=64%, list=36%, signal=97% |
| 694 | GSE14308\_TH17\_VS\_NATURAL\_TREG\_UP |  | 15 | -0.36 | -1.45 | 0.087 | 0.285 | 1.000 | 821 | tags=93%, list=58%, signal=221% |
| 695 | GSE17721\_PAM3CSK4\_VS\_CPG\_12H\_BMDC\_DN |  | 21 | -0.32 | -1.45 | 0.093 | 0.285 | 1.000 | 735 | tags=86%, list=52%, signal=176% |
| 696 | GSE40441\_NRP1\_POS\_INDUCED\_TREG\_VS\_NRP1\_NEG\_NATURAL\_TREG\_UP |  | 21 | -0.31 | -1.45 | 0.068 | 0.285 | 1.000 | 525 | tags=67%, list=37%, signal=105% |
| 697 | GSE40273\_GATA1\_KO\_VS\_WT\_TREG\_UP |  | 17 | -0.35 | -1.45 | 0.079 | 0.285 | 1.000 | 126 | tags=29%, list=9%, signal=32% |
| 698 | GSE15624\_3H\_VS\_6H\_HALOFUGINONE\_TREATED\_CD4\_TCELL\_UP |  | 20 | -0.31 | -1.45 | 0.081 | 0.285 | 1.000 | 315 | tags=50%, list=22%, signal=63% |
| 699 | GSE3982\_EOSINOPHIL\_VS\_BASOPHIL\_UP |  | 21 | -0.31 | -1.45 | 0.067 | 0.286 | 1.000 | 417 | tags=52%, list=30%, signal=73% |
| 700 | GSE22432\_CONVENTIONAL\_CDC\_VS\_PLASMACYTOID\_PDC\_DN |  | 15 | -0.36 | -1.45 | 0.075 | 0.286 | 1.000 | 434 | tags=67%, list=31%, signal=95% |
| 701 | GSE29164\_CD8\_TCELL\_VS\_CD8\_TCELL\_AND\_IL12\_TREATED\_MELANOMA\_DAY7\_DN |  | 16 | -0.34 | -1.45 | 0.078 | 0.285 | 1.000 | 314 | tags=44%, list=22%, signal=56% |
| 702 | GSE6259\_DEC205\_POS\_DC\_VS\_CD4\_TCELL\_UP |  | 16 | -0.35 | -1.45 | 0.072 | 0.285 | 1.000 | 599 | tags=75%, list=42%, signal=129% |
| 703 | GSE17721\_LPS\_VS\_POLYIC\_1H\_BMDC\_UP |  | 21 | -0.32 | -1.45 | 0.082 | 0.286 | 1.000 | 576 | tags=81%, list=41%, signal=135% |
| 704 | GSE37301\_HEMATOPOIETIC\_STEM\_CELL\_VS\_RAG2\_KO\_NK\_CELL\_DN |  | 20 | -0.32 | -1.45 | 0.071 | 0.286 | 1.000 | 558 | tags=75%, list=40%, signal=122% |
| 705 | GSE16451\_CTRL\_VS\_WEST\_EQUINE\_ENC\_VIRUS\_MATURE\_NEURON\_CELL\_LINE\_DN |  | 17 | -0.34 | -1.45 | 0.107 | 0.289 | 1.000 | 606 | tags=82%, list=43%, signal=143% |
| 706 | GSE36888\_STAT5\_AB\_KNOCKIN\_VS\_WT\_TCELL\_IL2\_TREATED\_2H\_DN |  | 24 | -0.29 | -1.45 | 0.070 | 0.289 | 1.000 | 248 | tags=42%, list=18%, signal=50% |
| 707 | GSE17721\_0.5H\_VS\_24H\_CPG\_BMDC\_DN |  | 20 | -0.32 | -1.45 | 0.079 | 0.290 | 1.000 | 515 | tags=70%, list=36%, signal=109% |
| 708 | GSE22935\_UNSTIM\_VS\_24H\_MBOVIS\_BCG\_STIM\_MACROPHAGE\_DN |  | 27 | -0.28 | -1.45 | 0.107 | 0.291 | 1.000 | 211 | tags=33%, list=15%, signal=38% |
| 709 | GSE9601\_NFKB\_INHIBITOR\_VS\_PI3K\_INHIBITOR\_TREATED\_HCMV\_INF\_MONOCYTE\_DN |  | 27 | -0.28 | -1.44 | 0.083 | 0.292 | 1.000 | 393 | tags=52%, list=28%, signal=70% |
| 710 | GSE5099\_DAY3\_VS\_DAY7\_MCSF\_TREATED\_MACROPHAGE\_UP |  | 19 | -0.33 | -1.44 | 0.077 | 0.292 | 1.000 | 322 | tags=53%, list=23%, signal=67% |
| 711 | GSE45881\_CXCR6HI\_VS\_CXCR1LO\_COLONIC\_LAMINA\_PROPRIA\_UP |  | 18 | -0.33 | -1.44 | 0.085 | 0.293 | 1.000 | 272 | tags=44%, list=19%, signal=54% |
| 712 | GSE7460\_CD8\_TCELL\_VS\_TREG\_ACT\_UP |  | 22 | -0.30 | -1.44 | 0.105 | 0.297 | 1.000 | 385 | tags=55%, list=27%, signal=74% |
| 713 | GSE36078\_WT\_VS\_IL1R\_KO\_LUNG\_DC\_AFTER\_AD5\_INF\_UP |  | 18 | -0.33 | -1.44 | 0.080 | 0.297 | 1.000 | 572 | tags=78%, list=41%, signal=129% |
| 714 | GSE46606\_DAY1\_VS\_DAY3\_CD40L\_IL2\_IL5\_STIMULATED\_IRF4HIGH\_BCELL\_DN |  | 24 | -0.29 | -1.44 | 0.074 | 0.297 | 1.000 | 359 | tags=46%, list=25%, signal=60% |
| 715 | GSE19923\_E2A\_KO\_VS\_E2A\_AND\_HEB\_KO\_DP\_THYMOCYTE\_UP |  | 23 | -0.30 | -1.44 | 0.087 | 0.297 | 1.000 | 676 | tags=78%, list=48%, signal=148% |
| 716 | GSE28726\_ACT\_CD4\_TCELL\_VS\_ACT\_NKTCELL\_DN |  | 26 | -0.28 | -1.44 | 0.086 | 0.300 | 1.000 | 299 | tags=38%, list=21%, signal=48% |
| 717 | GSE16385\_IFNG\_TNF\_VS\_IL4\_STIM\_MACROPHAGE\_UP |  | 25 | -0.29 | -1.44 | 0.090 | 0.300 | 1.000 | 395 | tags=52%, list=28%, signal=71% |
| 718 | GSE1925\_CTRL\_VS\_3H\_IFNG\_STIM\_IFNG\_PRIMED\_MACROPHAGE\_DN |  | 17 | -0.33 | -1.44 | 0.089 | 0.300 | 1.000 | 168 | tags=29%, list=12%, signal=33% |
| 719 | GSE11864\_CSF1\_PAM3CYS\_VS\_CSF1\_IFNG\_PAM3CYS\_IN\_MAC\_DN |  | 19 | -0.32 | -1.44 | 0.084 | 0.299 | 1.000 | 417 | tags=53%, list=30%, signal=74% |
| 720 | GSE17721\_0.5H\_VS\_8H\_POLYIC\_BMDC\_DN |  | 20 | -0.32 | -1.44 | 0.077 | 0.300 | 1.000 | 362 | tags=55%, list=26%, signal=73% |
| 721 | GSE18281\_SUBCAPSULAR\_VS\_PERIMEDULLARY\_CORTICAL\_REGION\_OF\_THYMUS\_DN |  | 16 | -0.35 | -1.44 | 0.110 | 0.300 | 1.000 | 388 | tags=56%, list=27%, signal=77% |
| 722 | GSE9988\_LOW\_LPS\_VS\_ANTI\_TREM1\_AND\_LPS\_MONOCYTE\_UP |  | 23 | -0.30 | -1.43 | 0.098 | 0.301 | 1.000 | 245 | tags=39%, list=17%, signal=47% |
| 723 | GSE1112\_HY\_CD8AB\_VS\_HY\_CD8AA\_THYMOCYTE\_RTOC\_CULTURE\_DN |  | 22 | -0.31 | -1.43 | 0.075 | 0.301 | 1.000 | 370 | tags=50%, list=26%, signal=67% |
| 724 | GSE3691\_IFN\_PRODUCING\_KILLER\_DC\_VS\_PLASMACYTOID\_DC\_SPLEEN\_UP |  | 21 | -0.30 | -1.43 | 0.099 | 0.301 | 1.000 | 408 | tags=57%, list=29%, signal=79% |
| 725 | GSE12845\_IGD\_NEG\_BLOOD\_VS\_PRE\_GC\_TONSIL\_BCELL\_DN |  | 16 | -0.34 | -1.43 | 0.083 | 0.300 | 1.000 | 936 | tags=100%, list=66%, signal=294% |
| 726 | GSE7852\_LN\_VS\_THYMUS\_TCONV\_UP |  | 30 | -0.27 | -1.43 | 0.086 | 0.300 | 1.000 | 70 | tags=20%, list=5%, signal=21% |
| 727 | GSE11961\_UNSTIM\_VS\_ANTI\_IGM\_AND\_CD40\_STIM\_6H\_FOLLICULAR\_BCELL\_DN |  | 19 | -0.32 | -1.43 | 0.093 | 0.300 | 1.000 | 412 | tags=58%, list=29%, signal=81% |
| 728 | GSE19512\_NAUTRAL\_VS\_INDUCED\_TREG\_DN |  | 23 | -0.30 | -1.43 | 0.097 | 0.300 | 1.000 | 468 | tags=57%, list=33%, signal=83% |
| 729 | GSE36476\_YOUNG\_VS\_OLD\_DONOR\_MEMORY\_CD4\_TCELL\_UP |  | 17 | -0.33 | -1.43 | 0.100 | 0.300 | 1.000 | 357 | tags=59%, list=25%, signal=78% |
| 730 | GSE19941\_IL10\_KO\_VS\_IL10\_KO\_AND\_NFKBP50\_KO\_LPS\_AND\_IL10\_STIM\_MACROPHAGE\_UP |  | 24 | -0.29 | -1.43 | 0.078 | 0.300 | 1.000 | 469 | tags=63%, list=33%, signal=92% |
| 731 | GSE41867\_NAIVE\_VS\_DAY30\_LCMV\_CLONE13\_EXHAUSTED\_CD8\_TCELL\_UP |  | 22 | -0.30 | -1.43 | 0.106 | 0.301 | 1.000 | 393 | tags=45%, list=28%, signal=62% |
| 732 | GSE23308\_CTRL\_VS\_CORTICOSTERONE\_TREATED\_MACROPHAGE\_MINERALCORTICOID\_REC\_KO\_DN |  | 25 | -0.28 | -1.43 | 0.081 | 0.303 | 1.000 | 284 | tags=40%, list=20%, signal=49% |
| 733 | GSE30971\_2H\_VS\_4H\_LPS\_STIM\_MACROPHAGE\_WBP7\_HET\_DN |  | 18 | -0.32 | -1.43 | 0.079 | 0.304 | 1.000 | 346 | tags=44%, list=25%, signal=58% |
| 734 | GSE9509\_LPS\_VS\_LPS\_AND\_IL10\_STIM\_IL10\_KO\_MACROPHAGE\_10MIN\_UP |  | 30 | -0.27 | -1.43 | 0.078 | 0.304 | 1.000 | 395 | tags=53%, list=28%, signal=72% |
| 735 | GSE8921\_UNSTIM\_0H\_VS\_TLR1\_2\_STIM\_MONOCYTE\_6H\_UP |  | 19 | -0.31 | -1.43 | 0.102 | 0.305 | 1.000 | 236 | tags=37%, list=17%, signal=44% |
| 736 | GSE2770\_TGFB\_AND\_IL4\_VS\_IL4\_TREATED\_ACT\_CD4\_TCELL\_6H\_DN |  | 18 | -0.32 | -1.43 | 0.096 | 0.306 | 1.000 | 270 | tags=44%, list=19%, signal=54% |
| 737 | GSE19941\_UNSTIM\_VS\_LPS\_STIM\_IL10\_KO\_MACROPHAGE\_UP |  | 17 | -0.33 | -1.43 | 0.088 | 0.306 | 1.000 | 357 | tags=53%, list=25%, signal=70% |
| 738 | GSE3982\_DC\_VS\_TH2\_DN |  | 19 | -0.32 | -1.42 | 0.082 | 0.306 | 1.000 | 816 | tags=89%, list=58%, signal=209% |
| 739 | GSE41867\_DAY8\_EFFECTOR\_VS\_DAY30\_EXHAUSTED\_CD8\_TCELL\_LCMV\_CLONE13\_DN |  | 17 | -0.34 | -1.42 | 0.106 | 0.306 | 1.000 | 221 | tags=41%, list=16%, signal=48% |
| 740 | GSE5542\_UNTREATED\_VS\_IFNG\_TREATED\_EPITHELIAL\_CELLS\_6H\_UP |  | 15 | -0.36 | -1.42 | 0.090 | 0.307 | 1.000 | 31 | tags=20%, list=2%, signal=20% |
| 741 | GSE7460\_FOXP3\_MUT\_VS\_WT\_ACT\_WITH\_TGFB\_TCONV\_DN |  | 29 | -0.27 | -1.42 | 0.097 | 0.307 | 1.000 | 259 | tags=34%, list=18%, signal=41% |
| 742 | GSE40274\_CTRL\_VS\_FOXP3\_AND\_EOS\_TRANSDUCED\_ACTIVATED\_CD4\_TCELL\_UP |  | 18 | -0.33 | -1.42 | 0.104 | 0.308 | 1.000 | 164 | tags=33%, list=12%, signal=37% |
| 743 | GSE2706\_UNSTIM\_VS\_8H\_LPS\_DC\_DN |  | 25 | -0.29 | -1.42 | 0.084 | 0.308 | 1.000 | 514 | tags=64%, list=36%, signal=99% |
| 744 | GSE21927\_UNTREATED\_VS\_GMCSF\_IL6\_TREATED\_BONE\_MARROW\_DN |  | 17 | -0.34 | -1.42 | 0.111 | 0.309 | 1.000 | 333 | tags=47%, list=24%, signal=61% |
| 745 | GSE1448\_CTRL\_VS\_ANTI\_VBETA5\_DP\_THYMOCYTE\_UP |  | 18 | -0.33 | -1.42 | 0.095 | 0.309 | 1.000 | 455 | tags=61%, list=32%, signal=89% |
| 746 | GSE37301\_LYMPHOID\_PRIMED\_MPP\_VS\_PRO\_BCELL\_DN |  | 16 | -0.34 | -1.42 | 0.121 | 0.309 | 1.000 | 350 | tags=56%, list=25%, signal=74% |
| 747 | GSE37416\_CTRL\_VS\_24H\_F\_TULARENSIS\_LVS\_NEUTROPHIL\_DN |  | 28 | -0.27 | -1.42 | 0.094 | 0.312 | 1.000 | 534 | tags=64%, list=38%, signal=101% |
| 748 | GSE3720\_VD1\_VS\_VD2\_GAMMADELTA\_TCELL\_DN |  | 21 | -0.30 | -1.42 | 0.074 | 0.312 | 1.000 | 426 | tags=62%, list=30%, signal=87% |
| 749 | GSE27241\_WT\_VS\_RORGT\_KO\_TH17\_POLARIZED\_CD4\_TCELL\_TREATED\_WITH\_DIGOXIN\_DN |  | 22 | -0.30 | -1.42 | 0.100 | 0.313 | 1.000 | 504 | tags=68%, list=36%, signal=104% |
| 750 | GSE34205\_HEALTHY\_VS\_RSV\_INF\_INFANT\_PBMC\_DN |  | 21 | -0.30 | -1.42 | 0.094 | 0.313 | 1.000 | 485 | tags=71%, list=34%, signal=107% |
| 751 | GSE13522\_CTRL\_VS\_T\_CRUZI\_G\_STRAIN\_INF\_SKIN\_UP |  | 19 | -0.32 | -1.42 | 0.096 | 0.313 | 1.000 | 162 | tags=32%, list=11%, signal=35% |
| 752 | GSE25088\_ROSIGLITAZONE\_VS\_IL4\_AND\_ROSIGLITAZONE\_STIM\_STAT6\_KO\_MACROPHAGE\_DAY10\_UP |  | 24 | -0.28 | -1.42 | 0.093 | 0.314 | 1.000 | 485 | tags=63%, list=34%, signal=94% |
| 753 | GSE15330\_HSC\_VS\_GRANULOCYTE\_MONOCYTE\_PROGENITOR\_IKAROS\_KO\_UP |  | 27 | -0.27 | -1.41 | 0.098 | 0.313 | 1.000 | 633 | tags=74%, list=45%, signal=132% |
| 754 | GSE15930\_STIM\_VS\_STIM\_AND\_IL12\_72H\_CD8\_T\_CELL\_UP |  | 18 | -0.32 | -1.41 | 0.081 | 0.313 | 1.000 | 866 | tags=94%, list=61%, signal=241% |
| 755 | GSE32986\_CURDLAN\_LOWDOSE\_VS\_CURDLAN\_HIGHDOSE\_STIM\_DC\_DN |  | 34 | -0.26 | -1.41 | 0.092 | 0.313 | 1.000 | 315 | tags=41%, list=22%, signal=52% |
| 756 | GSE17721\_CPG\_VS\_GARDIQUIMOD\_16H\_BMDC\_UP |  | 16 | -0.33 | -1.41 | 0.115 | 0.313 | 1.000 | 385 | tags=56%, list=27%, signal=76% |
| 757 | GSE17721\_12H\_VS\_24H\_LPS\_BMDC\_UP |  | 20 | -0.31 | -1.41 | 0.095 | 0.313 | 1.000 | 414 | tags=55%, list=29%, signal=77% |
| 758 | GSE20198\_IL12\_VS\_IFNA\_TREATED\_ACT\_CD4\_TCELL\_DN |  | 24 | -0.29 | -1.41 | 0.075 | 0.313 | 1.000 | 376 | tags=50%, list=27%, signal=67% |
| 759 | GSE22886\_NAIVE\_CD8\_TCELL\_VS\_MEMORY\_TCELL\_DN |  | 24 | -0.28 | -1.41 | 0.102 | 0.314 | 1.000 | 300 | tags=42%, list=21%, signal=52% |
| 760 | GSE10325\_LUPUS\_CD4\_TCELL\_VS\_LUPUS\_BCELL\_UP |  | 25 | -0.28 | -1.41 | 0.097 | 0.314 | 1.000 | 311 | tags=44%, list=22%, signal=55% |
| 761 | GSE24142\_DN2\_VS\_DN3\_THYMOCYTE\_UP |  | 22 | -0.30 | -1.41 | 0.088 | 0.313 | 1.000 | 415 | tags=55%, list=29%, signal=76% |
| 762 | GSE9988\_LPS\_VS\_CTRL\_TREATED\_MONOCYTE\_UP |  | 22 | -0.29 | -1.41 | 0.090 | 0.314 | 1.000 | 460 | tags=59%, list=33%, signal=86% |
| 763 | GSE1791\_CTRL\_VS\_NEUROMEDINU\_IN\_T\_CELL\_LINE\_6H\_UP |  | 27 | -0.27 | -1.41 | 0.096 | 0.317 | 1.000 | 293 | tags=37%, list=21%, signal=46% |
| 764 | GSE360\_L\_MAJOR\_VS\_M\_TUBERCULOSIS\_MAC\_UP |  | 15 | -0.35 | -1.41 | 0.123 | 0.317 | 1.000 | 35 | tags=20%, list=2%, signal=20% |
| 765 | GSE10325\_BCELL\_VS\_MYELOID\_DN |  | 16 | -0.34 | -1.41 | 0.124 | 0.316 | 1.000 | 562 | tags=75%, list=40%, signal=123% |
| 766 | GSE19923\_HEB\_KO\_VS\_HEB\_AND\_E2A\_KO\_DP\_THYMOCYTE\_DN |  | 25 | -0.28 | -1.41 | 0.107 | 0.317 | 1.000 | 300 | tags=40%, list=21%, signal=50% |
| 767 | GSE21360\_TERTIARY\_VS\_QUATERNARY\_MEMORY\_CD8\_TCELL\_DN |  | 25 | -0.28 | -1.41 | 0.090 | 0.316 | 1.000 | 550 | tags=68%, list=39%, signal=109% |
| 768 | GSE37301\_LYMPHOID\_PRIMED\_MPP\_VS\_COMMON\_LYMPHOID\_PROGENITOR\_UP |  | 17 | -0.34 | -1.41 | 0.114 | 0.316 | 1.000 | 469 | tags=65%, list=33%, signal=96% |
| 769 | GSE2585\_CTEC\_VS\_THYMIC\_DC\_DN |  | 20 | -0.31 | -1.41 | 0.089 | 0.317 | 1.000 | 572 | tags=80%, list=41%, signal=133% |
| 770 | GSE14415\_NATURAL\_TREG\_VS\_TCONV\_UP |  | 19 | -0.32 | -1.41 | 0.069 | 0.317 | 1.000 | 398 | tags=58%, list=28%, signal=80% |
| 771 | GSE19198\_1H\_VS\_6H\_IL21\_TREATED\_TCELL\_DN |  | 29 | -0.26 | -1.41 | 0.086 | 0.317 | 1.000 | 857 | tags=90%, list=61%, signal=224% |
| 772 | GSE3982\_DC\_VS\_BASOPHIL\_DN |  | 29 | -0.27 | -1.41 | 0.090 | 0.318 | 1.000 | 464 | tags=55%, list=33%, signal=81% |
| 773 | GSE23398\_WT\_VS\_IL2\_KO\_CD4\_TCELL\_SCURFY\_MOUSE\_DN |  | 21 | -0.31 | -1.41 | 0.097 | 0.319 | 1.000 | 259 | tags=38%, list=18%, signal=46% |
| 774 | GSE22886\_NAIVE\_CD4\_TCELL\_VS\_NEUTROPHIL\_DN |  | 20 | -0.30 | -1.40 | 0.085 | 0.319 | 1.000 | 550 | tags=70%, list=39%, signal=113% |
| 775 | GSE15271\_CXCR4\_POS\_VS\_NEG\_GC\_BCELL\_DN |  | 20 | -0.30 | -1.40 | 0.109 | 0.320 | 1.000 | 341 | tags=50%, list=24%, signal=65% |
| 776 | GSE26030\_UNSTIM\_VS\_RESTIM\_TH1\_DAY15\_POST\_POLARIZATION\_UP |  | 22 | -0.29 | -1.40 | 0.104 | 0.321 | 1.000 | 581 | tags=77%, list=41%, signal=129% |
| 777 | GSE2128\_CTRL\_VS\_MIMETOPE\_NEGATIVE\_SELECTION\_DP\_THYMOCYTE\_NOD\_UP |  | 17 | -0.33 | -1.40 | 0.112 | 0.321 | 1.000 | 541 | tags=71%, list=38%, signal=113% |
| 778 | GSE27434\_WT\_VS\_DNMT1\_KO\_TREG\_DN |  | 41 | -0.24 | -1.40 | 0.098 | 0.323 | 1.000 | 253 | tags=32%, list=18%, signal=38% |
| 779 | GSE26343\_WT\_VS\_NFAT5\_KO\_MACROPHAGE\_LPS\_STIM\_UP |  | 32 | -0.26 | -1.40 | 0.099 | 0.324 | 1.000 | 255 | tags=34%, list=18%, signal=41% |
| 780 | GSE7831\_UNSTIM\_VS\_INFLUENZA\_STIM\_PDC\_1H\_UP |  | 15 | -0.34 | -1.40 | 0.125 | 0.324 | 1.000 | 154 | tags=33%, list=11%, signal=37% |
| 781 | GSE2706\_R848\_VS\_R848\_AND\_LPS\_2H\_STIM\_DC\_DN |  | 21 | -0.30 | -1.40 | 0.115 | 0.327 | 1.000 | 255 | tags=43%, list=18%, signal=52% |
| 782 | GSE37533\_UNTREATED\_VS\_PIOGLIZATONE\_TREATED\_CD4\_TCELL\_PPARG1\_AND\_FOXP3\_TRASDUCED\_UP |  | 33 | -0.26 | -1.40 | 0.085 | 0.327 | 1.000 | 550 | tags=64%, list=39%, signal=102% |
| 783 | GSE17721\_ALL\_VS\_24H\_PAM3CSK4\_BMDC\_DN |  | 19 | -0.31 | -1.40 | 0.109 | 0.327 | 1.000 | 543 | tags=68%, list=38%, signal=110% |
| 784 | GSE7852\_TREG\_VS\_TCONV\_THYMUS\_DN |  | 22 | -0.29 | -1.40 | 0.100 | 0.327 | 1.000 | 455 | tags=64%, list=32%, signal=92% |
| 785 | GSE4590\_SMALL\_VS\_VPREB\_POS\_LARGE\_PRE\_BCELL\_DN |  | 18 | -0.32 | -1.39 | 0.137 | 0.329 | 1.000 | 341 | tags=44%, list=24%, signal=58% |
| 786 | GSE8921\_3H\_VS\_24H\_TLR1\_2\_STIM\_MONOCYTE\_UP |  | 18 | -0.32 | -1.39 | 0.119 | 0.329 | 1.000 | 251 | tags=44%, list=18%, signal=53% |
| 787 | GSE24726\_WT\_VS\_E2\_2\_KO\_PDC\_DAY6\_POST\_DELETION\_DN |  | 19 | -0.31 | -1.39 | 0.110 | 0.329 | 1.000 | 251 | tags=37%, list=18%, signal=44% |
| 788 | GSE21670\_UNTREATED\_VS\_IL6\_TREATED\_STAT3\_KO\_CD4\_TCELL\_DN |  | 39 | -0.23 | -1.39 | 0.093 | 0.328 | 1.000 | 140 | tags=21%, list=10%, signal=22% |
| 789 | GSE3982\_BCELL\_VS\_BASOPHIL\_DN |  | 22 | -0.29 | -1.39 | 0.112 | 0.331 | 1.000 | 153 | tags=27%, list=11%, signal=30% |
| 790 | GSE5099\_UNSTIM\_VS\_MCSF\_TREATED\_MONOCYTE\_DAY3\_DN |  | 20 | -0.30 | -1.39 | 0.131 | 0.332 | 1.000 | 341 | tags=50%, list=24%, signal=65% |
| 791 | GSE2770\_UNTREATED\_VS\_TGFB\_AND\_IL4\_TREATED\_ACT\_CD4\_TCELL\_48H\_DN |  | 25 | -0.28 | -1.39 | 0.110 | 0.332 | 1.000 | 471 | tags=56%, list=33%, signal=83% |
| 792 | GSE42021\_TCONV\_PLN\_VS\_CD24HI\_TCONV\_THYMUS\_DN |  | 23 | -0.28 | -1.39 | 0.116 | 0.332 | 1.000 | 32 | tags=17%, list=2%, signal=18% |
| 793 | GSE39556\_UNTREATED\_VS\_3H\_POLYIC\_INJ\_MOUSE\_NK\_CELL\_DN |  | 25 | -0.28 | -1.39 | 0.124 | 0.333 | 1.000 | 266 | tags=36%, list=19%, signal=44% |
| 794 | GSE36476\_CTRL\_VS\_TSST\_ACT\_72H\_MEMORY\_CD4\_TCELL\_OLD\_UP |  | 17 | -0.33 | -1.39 | 0.124 | 0.333 | 1.000 | 498 | tags=71%, list=35%, signal=108% |
| 795 | GSE17301\_ACD3\_ACD28\_VS\_ACD3\_ACD28\_AND\_IFNA5\_STIM\_CD8\_TCELL\_DN |  | 21 | -0.31 | -1.39 | 0.112 | 0.334 | 1.000 | 456 | tags=57%, list=32%, signal=83% |
| 796 | GSE25087\_FETAL\_VS\_ADULT\_TCONV\_DN |  | 15 | -0.35 | -1.39 | 0.118 | 0.334 | 1.000 | 416 | tags=60%, list=29%, signal=84% |
| 797 | GSE13738\_TCR\_VS\_BYSTANDER\_ACTIVATED\_CD4\_TCELL\_UP |  | 26 | -0.27 | -1.39 | 0.091 | 0.336 | 1.000 | 55 | tags=19%, list=4%, signal=20% |
| 798 | GSE17721\_POLYIC\_VS\_CPG\_6H\_BMDC\_UP |  | 18 | -0.31 | -1.38 | 0.120 | 0.337 | 1.000 | 173 | tags=33%, list=12%, signal=38% |
| 799 | GSE14699\_DELETIONAL\_TOLERANCE\_VS\_ACTIVATED\_CD8\_TCELL\_UP |  | 26 | -0.28 | -1.38 | 0.095 | 0.337 | 1.000 | 293 | tags=38%, list=21%, signal=48% |
| 800 | GSE11961\_UNSTIM\_VS\_ANTI\_IGM\_AND\_CD40\_STIM\_6H\_FOLLICULAR\_BCELL\_UP |  | 23 | -0.28 | -1.38 | 0.103 | 0.337 | 1.000 | 196 | tags=30%, list=14%, signal=35% |
| 801 | GSE7460\_CD8\_TCELL\_VS\_TREG\_ACT\_DN |  | 16 | -0.33 | -1.38 | 0.124 | 0.337 | 1.000 | 283 | tags=44%, list=20%, signal=54% |
| 802 | GSE23308\_CTRL\_VS\_CORTICOSTERONE\_TREATED\_MACROPHAGE\_DN |  | 16 | -0.33 | -1.38 | 0.129 | 0.338 | 1.000 | 210 | tags=38%, list=15%, signal=44% |
| 803 | GSE22886\_NAIVE\_CD4\_TCELL\_VS\_DC\_UP |  | 16 | -0.33 | -1.38 | 0.116 | 0.337 | 1.000 | 416 | tags=56%, list=29%, signal=79% |
| 804 | GSE3982\_DC\_VS\_TH2\_UP |  | 22 | -0.29 | -1.38 | 0.116 | 0.337 | 1.000 | 515 | tags=64%, list=36%, signal=99% |
| 805 | GSE41176\_UNSTIM\_VS\_ANTI\_IGM\_STIM\_TAK1\_KO\_BCELL\_24H\_UP |  | 18 | -0.31 | -1.38 | 0.105 | 0.337 | 1.000 | 349 | tags=50%, list=25%, signal=66% |
| 806 | GSE39820\_CTRL\_VS\_IL1B\_IL6\_CD4\_TCELL\_DN |  | 26 | -0.27 | -1.38 | 0.113 | 0.337 | 1.000 | 561 | tags=73%, list=40%, signal=119% |
| 807 | GSE5589\_WT\_VS\_IL10\_KO\_LPS\_AND\_IL10\_STIM\_MACROPHAGE\_45MIN\_DN |  | 19 | -0.31 | -1.38 | 0.099 | 0.337 | 1.000 | 792 | tags=89%, list=56%, signal=201% |
| 808 | GSE36392\_TYPE\_2\_MYELOID\_VS\_MAC\_IL25\_TREATED\_LUNG\_UP |  | 17 | -0.32 | -1.38 | 0.090 | 0.337 | 1.000 | 294 | tags=47%, list=21%, signal=59% |
| 809 | GSE24102\_GRANULOCYSTIC\_MDSC\_VS\_NEUTROPHIL\_UP |  | 20 | -0.29 | -1.38 | 0.099 | 0.338 | 1.000 | 1004 | tags=100%, list=71%, signal=342% |
| 810 | GSE34006\_UNTREATED\_VS\_A2AR\_AGONIST\_TREATED\_TREG\_DN |  | 24 | -0.28 | -1.38 | 0.098 | 0.337 | 1.000 | 563 | tags=71%, list=40%, signal=116% |
| 811 | GSE17721\_LPS\_VS\_PAM3CSK4\_4H\_BMDC\_UP |  | 25 | -0.27 | -1.38 | 0.123 | 0.337 | 1.000 | 440 | tags=56%, list=31%, signal=80% |
| 812 | GSE1112\_HY\_CD8AB\_VS\_HY\_CD8AA\_THYMOCYTE\_RTOC\_CULTURE\_UP |  | 15 | -0.34 | -1.38 | 0.126 | 0.337 | 1.000 | 599 | tags=80%, list=42%, signal=138% |
| 813 | GSE23984\_CTRL\_VS\_HYPOCALEMIC\_VITAMIND\_ANALOG\_TCELL\_DN |  | 18 | -0.31 | -1.38 | 0.116 | 0.337 | 1.000 | 532 | tags=72%, list=38%, signal=114% |
| 814 | GSE17721\_POLYIC\_VS\_PAM3CSK4\_6H\_BMDC\_UP |  | 22 | -0.29 | -1.38 | 0.113 | 0.337 | 1.000 | 228 | tags=36%, list=16%, signal=43% |
| 815 | GSE27786\_BCELL\_VS\_CD4\_TCELL\_DN |  | 18 | -0.30 | -1.38 | 0.134 | 0.338 | 1.000 | 769 | tags=89%, list=55%, signal=193% |
| 816 | GSE37301\_COMMON\_LYMPHOID\_PROGENITOR\_VS\_RAG2\_KO\_NK\_CELL\_UP |  | 18 | -0.31 | -1.38 | 0.104 | 0.339 | 1.000 | 522 | tags=67%, list=37%, signal=104% |
| 817 | GSE39820\_IL1B\_IL6\_VS\_IL1B\_IL6\_IL23A\_TREATED\_CD4\_TCELL\_DN |  | 16 | -0.33 | -1.38 | 0.099 | 0.340 | 1.000 | 103 | tags=25%, list=7%, signal=27% |
| 818 | GSE17721\_CTRL\_VS\_PAM3CSK4\_8H\_BMDC\_DN |  | 15 | -0.34 | -1.38 | 0.126 | 0.342 | 1.000 | 537 | tags=73%, list=38%, signal=117% |
| 819 | GSE19198\_1H\_VS\_24H\_IL21\_TREATED\_TCELL\_UP |  | 15 | -0.34 | -1.38 | 0.132 | 0.342 | 1.000 | 605 | tags=80%, list=43%, signal=139% |
| 820 | GSE41176\_UNSTIM\_VS\_ANTI\_IGM\_STIM\_BCELL\_3H\_UP |  | 21 | -0.30 | -1.37 | 0.100 | 0.342 | 1.000 | 411 | tags=52%, list=29%, signal=73% |
| 821 | GSE17721\_CTRL\_VS\_LPS\_12H\_BMDC\_DN |  | 16 | -0.33 | -1.37 | 0.104 | 0.343 | 1.000 | 713 | tags=88%, list=51%, signal=175% |
| 822 | GSE14415\_INDUCED\_TREG\_VS\_FOXP3\_KO\_INDUCED\_TREG\_DN |  | 17 | -0.32 | -1.37 | 0.132 | 0.343 | 1.000 | 253 | tags=35%, list=18%, signal=42% |
| 823 | GSE12366\_GC\_VS\_NAIVE\_BCELL\_DN |  | 17 | -0.32 | -1.37 | 0.105 | 0.343 | 1.000 | 423 | tags=59%, list=30%, signal=83% |
| 824 | GSE3039\_NKT\_CELL\_VS\_ALPHABETA\_CD8\_TCELL\_DN |  | 19 | -0.31 | -1.37 | 0.113 | 0.344 | 1.000 | 327 | tags=47%, list=23%, signal=61% |
| 825 | GSE22886\_NAIVE\_BCELL\_VS\_DC\_DN |  | 19 | -0.31 | -1.37 | 0.118 | 0.344 | 1.000 | 535 | tags=68%, list=38%, signal=109% |
| 826 | GSE22342\_CD11C\_HIGH\_VS\_LOW\_DECIDUAL\_MACROPHAGES\_UP |  | 19 | -0.31 | -1.37 | 0.122 | 0.345 | 1.000 | 451 | tags=58%, list=32%, signal=84% |
| 827 | GSE21546\_SAP1A\_KO\_VS\_SAP1A\_KO\_AND\_ELK1\_KO\_DP\_THYMOCYTES\_DN |  | 19 | -0.31 | -1.37 | 0.132 | 0.345 | 1.000 | 366 | tags=53%, list=26%, signal=70% |
| 828 | GSE46242\_TH1\_VS\_ANERGIC\_TH1\_CD4\_TCELL\_WITH\_EGR2\_DELETED\_DN |  | 18 | -0.31 | -1.37 | 0.128 | 0.345 | 1.000 | 399 | tags=56%, list=28%, signal=76% |
| 829 | GSE1448\_CTRL\_VS\_ANTI\_VBETA5\_DP\_THYMOCYTE\_DN |  | 18 | -0.32 | -1.37 | 0.126 | 0.346 | 1.000 | 145 | tags=28%, list=10%, signal=31% |
| 830 | GSE13411\_NAIVE\_BCELL\_VS\_PLASMA\_CELL\_UP |  | 30 | -0.25 | -1.37 | 0.127 | 0.346 | 1.000 | 464 | tags=57%, list=33%, signal=83% |
| 831 | GSE20715\_WT\_VS\_TLR4\_KO\_48H\_OZONE\_LUNG\_UP |  | 18 | -0.31 | -1.37 | 0.116 | 0.347 | 1.000 | 381 | tags=56%, list=27%, signal=75% |
| 832 | GSE12198\_CTRL\_VS\_LOW\_IL2\_STIM\_NK\_CELL\_UP |  | 21 | -0.29 | -1.37 | 0.122 | 0.346 | 1.000 | 155 | tags=24%, list=11%, signal=26% |
| 833 | GSE21670\_IL6\_VS\_TGFB\_AND\_IL6\_TREATED\_STAT3\_KO\_CD4\_TCELL\_DN |  | 20 | -0.29 | -1.37 | 0.098 | 0.347 | 1.000 | 168 | tags=35%, list=12%, signal=39% |
| 834 | GSE22886\_NAIVE\_TCELL\_VS\_MONOCYTE\_DN |  | 18 | -0.30 | -1.37 | 0.123 | 0.348 | 1.000 | 276 | tags=39%, list=20%, signal=48% |
| 835 | GSE17721\_PAM3CSK4\_VS\_GADIQUIMOD\_0.5H\_BMDC\_DN |  | 18 | -0.31 | -1.37 | 0.135 | 0.349 | 1.000 | 754 | tags=89%, list=53%, signal=188% |
| 836 | GSE27786\_CD8\_TCELL\_VS\_NKCELL\_DN |  | 25 | -0.28 | -1.36 | 0.134 | 0.351 | 1.000 | 418 | tags=52%, list=30%, signal=73% |
| 837 | GSE26928\_NAIVE\_VS\_CENT\_MEMORY\_CD4\_TCELL\_DN |  | 17 | -0.31 | -1.36 | 0.133 | 0.351 | 1.000 | 42 | tags=18%, list=3%, signal=18% |
| 838 | GSE15930\_STIM\_VS\_STIM\_AND\_TRICHOSTATINA\_48H\_CD8\_T\_CELL\_DN |  | 21 | -0.30 | -1.36 | 0.116 | 0.351 | 1.000 | 562 | tags=71%, list=40%, signal=117% |
| 839 | GSE17721\_LPS\_VS\_PAM3CSK4\_2H\_BMDC\_UP |  | 23 | -0.28 | -1.36 | 0.121 | 0.352 | 1.000 | 101 | tags=26%, list=7%, signal=28% |
| 840 | GSE2826\_WT\_VS\_XID\_BCELL\_DN |  | 19 | -0.31 | -1.36 | 0.131 | 0.352 | 1.000 | 573 | tags=74%, list=41%, signal=122% |
| 841 | GSE35543\_IN\_VITRO\_ITREG\_VS\_CONVERTED\_EX\_ITREG\_DN |  | 16 | -0.32 | -1.36 | 0.128 | 0.352 | 1.000 | 416 | tags=56%, list=29%, signal=79% |
| 842 | GSE14308\_TH2\_VS\_TH1\_DN |  | 21 | -0.29 | -1.36 | 0.143 | 0.352 | 1.000 | 572 | tags=71%, list=41%, signal=118% |
| 843 | GSE9960\_GRAM\_NEG\_VS\_GRAM\_POS\_SEPSIS\_PBMC\_UP |  | 23 | -0.28 | -1.36 | 0.113 | 0.357 | 1.000 | 547 | tags=70%, list=39%, signal=112% |
| 844 | GSE339\_EX\_VIVO\_VS\_IN\_CULTURE\_CD8POS\_DC\_UP |  | 18 | -0.30 | -1.36 | 0.115 | 0.357 | 1.000 | 880 | tags=94%, list=62%, signal=248% |
| 845 | GSE17721\_LPS\_VS\_POLYIC\_6H\_BMDC\_UP |  | 15 | -0.34 | -1.36 | 0.120 | 0.357 | 1.000 | 639 | tags=80%, list=45%, signal=145% |
| 846 | GSE17721\_POLYIC\_VS\_PAM3CSK4\_1H\_BMDC\_DN |  | 28 | -0.26 | -1.36 | 0.135 | 0.357 | 1.000 | 243 | tags=36%, list=17%, signal=42% |
| 847 | GSE22103\_UNSTIM\_VS\_LPS\_STIM\_NEUTROPHIL\_DN |  | 26 | -0.27 | -1.36 | 0.108 | 0.357 | 1.000 | 120 | tags=23%, list=9%, signal=25% |
| 848 | GSE17721\_CTRL\_VS\_POLYIC\_1H\_BMDC\_DN |  | 20 | -0.31 | -1.36 | 0.115 | 0.357 | 1.000 | 985 | tags=100%, list=70%, signal=327% |
| 849 | GSE25087\_TREG\_VS\_TCONV\_ADULT\_UP |  | 27 | -0.26 | -1.36 | 0.137 | 0.357 | 1.000 | 210 | tags=30%, list=15%, signal=34% |
| 850 | GSE21360\_NAIVE\_VS\_SECONDARY\_MEMORY\_CD8\_TCELL\_DN |  | 26 | -0.27 | -1.36 | 0.101 | 0.357 | 1.000 | 289 | tags=38%, list=20%, signal=47% |
| 851 | GSE28408\_LY6G\_POS\_VS\_NEG\_DC\_DN |  | 22 | -0.29 | -1.36 | 0.118 | 0.356 | 1.000 | 766 | tags=86%, list=54%, signal=186% |
| 852 | GSE10240\_IL22\_VS\_IL17\_STIM\_PRIMARY\_BRONCHIAL\_EPITHELIAL\_CELLS\_DN |  | 17 | -0.31 | -1.36 | 0.141 | 0.356 | 1.000 | 515 | tags=65%, list=36%, signal=101% |
| 853 | GSE32986\_CURDLAN\_LOWDOSE\_VS\_CURDLAN\_HIGHDOSE\_STIM\_DC\_UP |  | 30 | -0.26 | -1.36 | 0.136 | 0.356 | 1.000 | 252 | tags=37%, list=18%, signal=44% |
| 854 | GSE46242\_CTRL\_VS\_EGR2\_DELETED\_ANERGIC\_TH1\_CD4\_TCELL\_UP |  | 28 | -0.26 | -1.35 | 0.121 | 0.359 | 1.000 | 144 | tags=25%, list=10%, signal=27% |
| 855 | GSE41867\_DAY6\_VS\_DAY8\_LCMV\_CLONE13\_EFFECTOR\_CD8\_TCELL\_UP |  | 15 | -0.33 | -1.35 | 0.114 | 0.359 | 1.000 | 131 | tags=27%, list=9%, signal=29% |
| 856 | GSE16450\_CTRL\_VS\_IFNA\_6H\_STIM\_MATURE\_NEURON\_CELL\_LINE\_UP |  | 18 | -0.30 | -1.35 | 0.109 | 0.359 | 1.000 | 310 | tags=44%, list=22%, signal=56% |
| 857 | GSE17721\_PAM3CSK4\_VS\_GADIQUIMOD\_24H\_BMDC\_DN |  | 16 | -0.32 | -1.35 | 0.112 | 0.359 | 1.000 | 178 | tags=31%, list=13%, signal=35% |
| 858 | GSE34515\_CD16\_POS\_MONOCYTE\_VS\_DC\_DN |  | 22 | -0.29 | -1.35 | 0.122 | 0.360 | 1.000 | 452 | tags=55%, list=32%, signal=79% |
| 859 | GSE36888\_UNTREATED\_VS\_IL2\_TREATED\_TCELL\_17H\_DN |  | 26 | -0.27 | -1.35 | 0.146 | 0.360 | 1.000 | 572 | tags=73%, list=41%, signal=121% |
| 860 | GSE15330\_WT\_VS\_IKAROS\_KO\_HSC\_DN |  | 21 | -0.29 | -1.35 | 0.126 | 0.360 | 1.000 | 644 | tags=76%, list=46%, signal=138% |
| 861 | GSE13306\_TREG\_VS\_TCONV\_UP |  | 17 | -0.32 | -1.35 | 0.112 | 0.361 | 1.000 | 315 | tags=47%, list=22%, signal=60% |
| 862 | GSE45739\_NRAS\_KO\_VS\_WT\_UNSTIM\_CD4\_TCELL\_DN |  | 21 | -0.29 | -1.35 | 0.132 | 0.360 | 1.000 | 174 | tags=38%, list=12%, signal=43% |
| 863 | GSE3982\_DC\_VS\_MAC\_UP |  | 25 | -0.27 | -1.35 | 0.143 | 0.361 | 1.000 | 455 | tags=60%, list=32%, signal=87% |
| 864 | GSE41867\_MEMORY\_VS\_EXHAUSTED\_CD8\_TCELL\_DAY30\_LCMV\_DN |  | 17 | -0.31 | -1.35 | 0.131 | 0.361 | 1.000 | 433 | tags=53%, list=31%, signal=75% |
| 865 | GSE32986\_UNSTIM\_VS\_GMCSF\_AND\_CURDLAN\_LOWDOSE\_STIM\_DC\_DN |  | 29 | -0.26 | -1.35 | 0.152 | 0.361 | 1.000 | 259 | tags=41%, list=18%, signal=50% |
| 866 | GSE17721\_CPG\_VS\_GARDIQUIMOD\_4H\_BMDC\_DN |  | 17 | -0.32 | -1.35 | 0.125 | 0.361 | 1.000 | 572 | tags=76%, list=41%, signal=127% |
| 867 | GSE20715\_0H\_VS\_24H\_OZONE\_LUNG\_UP |  | 15 | -0.33 | -1.35 | 0.145 | 0.361 | 1.000 | 253 | tags=40%, list=18%, signal=48% |
| 868 | GSE5589\_LPS\_AND\_IL10\_VS\_LPS\_AND\_IL6\_STIM\_IL6\_KO\_MACROPHAGE\_45MIN\_UP |  | 18 | -0.32 | -1.35 | 0.129 | 0.361 | 1.000 | 391 | tags=50%, list=28%, signal=68% |
| 869 | GSE40068\_CXCR5POS\_BCL6POS\_TFH\_VS\_CXCR5NEG\_BCL6NEG\_CD4\_TCELL\_UP |  | 25 | -0.27 | -1.35 | 0.136 | 0.361 | 1.000 | 349 | tags=44%, list=25%, signal=57% |
| 870 | GSE37301\_MULTIPOTENT\_PROGENITOR\_VS\_GRAN\_MONO\_PROGENITOR\_DN |  | 24 | -0.28 | -1.35 | 0.132 | 0.361 | 1.000 | 512 | tags=67%, list=36%, signal=103% |
| 871 | GSE17721\_LPS\_VS\_GARDIQUIMOD\_0.5H\_BMDC\_DN |  | 21 | -0.28 | -1.35 | 0.119 | 0.362 | 1.000 | 1020 | tags=100%, list=72%, signal=355% |
| 872 | GSE360\_L\_DONOVANI\_VS\_L\_MAJOR\_DC\_DN |  | 16 | -0.32 | -1.35 | 0.152 | 0.362 | 1.000 | 190 | tags=38%, list=13%, signal=43% |
| 873 | GSE20715\_0H\_VS\_48H\_OZONE\_LUNG\_DN |  | 30 | -0.25 | -1.35 | 0.127 | 0.362 | 1.000 | 289 | tags=37%, list=20%, signal=45% |
| 874 | GSE32986\_UNSTIM\_VS\_CURDLAN\_HIGHDOSE\_STIM\_DC\_DN |  | 20 | -0.29 | -1.35 | 0.153 | 0.362 | 1.000 | 173 | tags=30%, list=12%, signal=34% |
| 875 | GSE1448\_CTRL\_VS\_ANTI\_VALPHA2\_DP\_THYMOCYTE\_DN |  | 19 | -0.30 | -1.34 | 0.149 | 0.362 | 1.000 | 819 | tags=89%, list=58%, signal=210% |
| 876 | GSE4590\_LARGE\_PRE\_BCELL\_VS\_VPREB\_POS\_LARGE\_PRE\_BCELL\_DN |  | 16 | -0.32 | -1.34 | 0.138 | 0.365 | 1.000 | 553 | tags=75%, list=39%, signal=122% |
| 877 | GSE37301\_HEMATOPOIETIC\_STEM\_CELL\_VS\_GRAN\_MONO\_PROGENITOR\_DN |  | 25 | -0.27 | -1.34 | 0.123 | 0.365 | 1.000 | 251 | tags=32%, list=18%, signal=38% |
| 878 | GSE9509\_LPS\_VS\_LPS\_AND\_IL10\_STIM\_IL10\_KO\_MACROPHAGE\_30MIN\_DN |  | 20 | -0.29 | -1.34 | 0.126 | 0.365 | 1.000 | 517 | tags=65%, list=37%, signal=101% |
| 879 | GSE22025\_UNTREATED\_VS\_TGFB1\_AND\_PROGESTERONE\_TREATED\_CD4\_TCELL\_DN |  | 19 | -0.30 | -1.34 | 0.117 | 0.365 | 1.000 | 170 | tags=32%, list=12%, signal=35% |
| 880 | GSE17721\_CTRL\_VS\_POLYIC\_0.5H\_BMDC\_UP |  | 19 | -0.30 | -1.34 | 0.130 | 0.365 | 1.000 | 774 | tags=89%, list=55%, signal=196% |
| 881 | GSE16522\_ANTI\_CD3CD28\_STIM\_VS\_UNSTIM\_MEMORY\_CD8\_TCELL\_UP |  | 28 | -0.26 | -1.34 | 0.099 | 0.365 | 1.000 | 990 | tags=96%, list=70%, signal=317% |
| 882 | GSE19941\_LPS\_VS\_LPS\_AND\_IL10\_STIM\_IL10\_KO\_NFKBP50\_KO\_MACROPHAGE\_DN |  | 20 | -0.29 | -1.34 | 0.134 | 0.365 | 1.000 | 474 | tags=60%, list=34%, signal=89% |
| 883 | GSE21379\_WT\_VS\_SAP\_KO\_CD4\_TCELL\_DN |  | 21 | -0.28 | -1.34 | 0.123 | 0.365 | 1.000 | 353 | tags=52%, list=25%, signal=69% |
| 884 | GOLDRATH\_EFF\_VS\_MEMORY\_CD8\_TCELL\_UP |  | 23 | -0.28 | -1.34 | 0.145 | 0.365 | 1.000 | 549 | tags=65%, list=39%, signal=105% |
| 885 | GSE5589\_IL6\_KO\_VS\_IL10\_KO\_LPS\_AND\_IL10\_STIM\_MACROPHAGE\_45MIN\_DN |  | 18 | -0.31 | -1.34 | 0.135 | 0.366 | 1.000 | 437 | tags=56%, list=31%, signal=79% |
| 886 | GSE36888\_UNTREATED\_VS\_IL2\_TREATED\_STAT5\_AB\_KNOCKIN\_TCELL\_17H\_UP |  | 25 | -0.27 | -1.34 | 0.134 | 0.365 | 1.000 | 538 | tags=68%, list=38%, signal=108% |
| 887 | GSE17301\_CTRL\_VS\_48H\_ACD3\_ACD28\_IFNA2\_STIM\_CD8\_TCELL\_DN |  | 15 | -0.33 | -1.34 | 0.144 | 0.366 | 1.000 | 306 | tags=47%, list=22%, signal=59% |
| 888 | GSE22140\_GERMFREE\_VS\_SPF\_ARTHRITIC\_MOUSE\_CD4\_TCELL\_DN |  | 27 | -0.26 | -1.34 | 0.118 | 0.368 | 1.000 | 273 | tags=37%, list=19%, signal=45% |
| 889 | GSE21774\_CD62L\_POS\_CD56\_DIM\_VS\_CD62L\_NEG\_CD56\_DIM\_NK\_CELL\_DN |  | 16 | -0.32 | -1.34 | 0.135 | 0.368 | 1.000 | 298 | tags=44%, list=21%, signal=55% |
| 890 | GSE21927\_SPLEEN\_VS\_TUMOR\_MONOCYTE\_BALBC\_DN |  | 21 | -0.28 | -1.34 | 0.133 | 0.369 | 1.000 | 213 | tags=29%, list=15%, signal=33% |
| 891 | GSE32986\_CURDLAN\_HIGHDOSE\_VS\_GMCSF\_AND\_CURDLAN\_HIGHDOSE\_STIM\_DC\_DN |  | 29 | -0.26 | -1.34 | 0.128 | 0.370 | 1.000 | 557 | tags=72%, list=39%, signal=117% |
| 892 | GSE19401\_RETINOIC\_ACID\_VS\_RETINOIC\_ACID\_AND\_PAM2CSK4\_STIM\_FOLLICULAR\_DC\_UP |  | 28 | -0.26 | -1.33 | 0.138 | 0.374 | 1.000 | 447 | tags=57%, list=32%, signal=82% |
| 893 | GSE3982\_NEUTROPHIL\_VS\_BASOPHIL\_UP |  | 23 | -0.28 | -1.33 | 0.129 | 0.374 | 1.000 | 417 | tags=57%, list=30%, signal=79% |
| 894 | GSE40274\_FOXP3\_VS\_FOXP3\_AND\_LEF1\_TRANSDUCED\_ACTIVATED\_CD4\_TCELL\_DN |  | 17 | -0.30 | -1.33 | 0.139 | 0.374 | 1.000 | 247 | tags=35%, list=18%, signal=42% |
| 895 | GSE3039\_ALPHAALPHA\_CD8\_TCELL\_VS\_B2\_BCELL\_DN |  | 28 | -0.25 | -1.33 | 0.165 | 0.374 | 1.000 | 1066 | tags=100%, list=76%, signal=401% |
| 896 | GSE9988\_ANTI\_TREM1\_VS\_LPS\_MONOCYTE\_UP |  | 32 | -0.24 | -1.33 | 0.119 | 0.374 | 1.000 | 470 | tags=56%, list=33%, signal=82% |
| 897 | GSE3039\_CD4\_TCELL\_VS\_ALPHABETA\_CD8\_TCELL\_DN |  | 18 | -0.30 | -1.33 | 0.146 | 0.374 | 1.000 | 84 | tags=22%, list=6%, signal=23% |
| 898 | GSE5589\_LPS\_VS\_LPS\_AND\_IL10\_STIM\_IL10\_KO\_MACROPHAGE\_45MIN\_UP |  | 31 | -0.25 | -1.33 | 0.127 | 0.374 | 1.000 | 382 | tags=48%, list=27%, signal=65% |
| 899 | GSE22886\_NAIVE\_CD4\_TCELL\_VS\_NKCELL\_DN |  | 25 | -0.27 | -1.33 | 0.147 | 0.374 | 1.000 | 83 | tags=20%, list=6%, signal=21% |
| 900 | GSE22527\_ANTI\_CD3\_INVIVO\_VS\_UNTREATED\_MOUSE\_TREG\_UP |  | 19 | -0.29 | -1.33 | 0.115 | 0.374 | 1.000 | 372 | tags=53%, list=26%, signal=71% |
| 901 | GSE36476\_CTRL\_VS\_TSST\_ACT\_16H\_MEMORY\_CD4\_TCELL\_OLD\_UP |  | 17 | -0.30 | -1.33 | 0.131 | 0.375 | 1.000 | 555 | tags=76%, list=39%, signal=125% |
| 902 | GSE32901\_TH17\_EMRICHED\_VS\_TH17\_NEG\_CD4\_TCELL\_DN |  | 18 | -0.30 | -1.33 | 0.154 | 0.377 | 1.000 | 325 | tags=44%, list=23%, signal=57% |
| 903 | GSE2405\_S\_AUREUS\_VS\_A\_PHAGOCYTOPHILUM\_NEUTROPHIL\_UP |  | 19 | -0.29 | -1.33 | 0.134 | 0.381 | 1.000 | 557 | tags=68%, list=39%, signal=112% |
| 904 | GSE36527\_CD62L\_HIGH\_CD69\_NEG\_VS\_CD62L\_LOW\_CD69\_POS\_TREG\_KLRG1\_NEG\_UP |  | 15 | -0.33 | -1.32 | 0.157 | 0.381 | 1.000 | 317 | tags=47%, list=22%, signal=60% |
| 905 | GSE21033\_CTRL\_VS\_POLYIC\_STIM\_DC\_6H\_UP |  | 28 | -0.25 | -1.32 | 0.123 | 0.381 | 1.000 | 576 | tags=68%, list=41%, signal=112% |
| 906 | GSE30962\_ACUTE\_VS\_CHRONIC\_LCMV\_PRIMARY\_INF\_CD8\_TCELL\_DN |  | 22 | -0.28 | -1.32 | 0.155 | 0.381 | 1.000 | 662 | tags=77%, list=47%, signal=143% |
| 907 | GSE339\_CD8POS\_VS\_CD4CD8DN\_DC\_IN\_CULTURE\_DN |  | 15 | -0.33 | -1.32 | 0.162 | 0.383 | 1.000 | 122 | tags=27%, list=9%, signal=29% |
| 908 | GSE11386\_NAIVE\_VS\_MEMORY\_BCELL\_UP |  | 16 | -0.31 | -1.32 | 0.150 | 0.383 | 1.000 | 690 | tags=81%, list=49%, signal=157% |
| 909 | GSE7831\_UNSTIM\_VS\_CPG\_STIM\_PDC\_1H\_DN |  | 20 | -0.29 | -1.32 | 0.148 | 0.383 | 1.000 | 140 | tags=30%, list=10%, signal=33% |
| 910 | GSE13411\_PLASMA\_CELL\_VS\_MEMORY\_BCELL\_UP |  | 20 | -0.29 | -1.32 | 0.149 | 0.383 | 1.000 | 306 | tags=40%, list=22%, signal=50% |
| 911 | GSE23568\_ID3\_TRANSDUCED\_VS\_ID3\_KO\_CD8\_TCELL\_DN |  | 23 | -0.28 | -1.32 | 0.140 | 0.384 | 1.000 | 557 | tags=65%, list=39%, signal=106% |
| 912 | GSE1432\_1H\_VS\_6H\_IFNG\_MICROGLIA\_DN |  | 24 | -0.27 | -1.32 | 0.136 | 0.384 | 1.000 | 512 | tags=63%, list=36%, signal=96% |
| 913 | GSE4142\_NAIVE\_BCELL\_VS\_PLASMA\_CELL\_UP |  | 21 | -0.28 | -1.32 | 0.136 | 0.384 | 1.000 | 469 | tags=62%, list=33%, signal=91% |
| 914 | GSE35685\_CD34POS\_CD38NEG\_VS\_CD34POS\_CD10POS\_BONE\_MARROW\_UP |  | 16 | -0.32 | -1.32 | 0.170 | 0.385 | 1.000 | 270 | tags=44%, list=19%, signal=53% |
| 915 | GSE6269\_HEALTHY\_VS\_E\_COLI\_INF\_PBMC\_DN |  | 22 | -0.28 | -1.32 | 0.140 | 0.385 | 1.000 | 245 | tags=41%, list=17%, signal=49% |
| 916 | GSE20715\_0H\_VS\_6H\_OZONE\_LUNG\_DN |  | 26 | -0.26 | -1.32 | 0.160 | 0.386 | 1.000 | 563 | tags=69%, list=40%, signal=113% |
| 917 | GSE3982\_BCELL\_VS\_CENT\_MEMORY\_CD4\_TCELL\_UP |  | 24 | -0.27 | -1.32 | 0.131 | 0.386 | 1.000 | 205 | tags=29%, list=15%, signal=34% |
| 918 | GSE28726\_NAIVE\_CD4\_TCELL\_VS\_NAIVE\_VA24NEG\_NKTCELL\_UP |  | 18 | -0.31 | -1.32 | 0.150 | 0.386 | 1.000 | 576 | tags=72%, list=41%, signal=120% |
| 919 | GSE3982\_EOSINOPHIL\_VS\_EFF\_MEMORY\_CD4\_TCELL\_UP |  | 31 | -0.25 | -1.32 | 0.135 | 0.389 | 1.000 | 428 | tags=52%, list=30%, signal=72% |
| 920 | GSE3982\_EFF\_MEMORY\_CD4\_TCELL\_VS\_TH2\_UP |  | 20 | -0.28 | -1.32 | 0.164 | 0.389 | 1.000 | 511 | tags=65%, list=36%, signal=100% |
| 921 | GSE28737\_WT\_VS\_BCL6\_HET\_MARGINAL\_ZONE\_BCELL\_DN |  | 27 | -0.26 | -1.32 | 0.133 | 0.389 | 1.000 | 245 | tags=33%, list=17%, signal=40% |
| 922 | GSE42021\_TREG\_PLN\_VS\_CD24HI\_TREG\_THYMUS\_DN |  | 20 | -0.29 | -1.31 | 0.149 | 0.388 | 1.000 | 383 | tags=45%, list=27%, signal=61% |
| 923 | GSE26023\_PHD3\_KO\_VS\_WT\_NEUTROPHIL\_HYPOXIA\_UP |  | 19 | -0.30 | -1.31 | 0.154 | 0.389 | 1.000 | 368 | tags=47%, list=26%, signal=63% |
| 924 | GSE42021\_CD24INT\_TREG\_VS\_CD24INT\_TCONV\_THYMUS\_UP |  | 22 | -0.28 | -1.31 | 0.173 | 0.390 | 1.000 | 880 | tags=91%, list=62%, signal=238% |
| 925 | GSE1460\_CD4\_THYMOCYTE\_VS\_NAIVE\_CD4\_TCELL\_ADULT\_BLOOD\_DN |  | 19 | -0.29 | -1.31 | 0.152 | 0.390 | 1.000 | 180 | tags=32%, list=13%, signal=36% |
| 926 | GSE18893\_TCONV\_VS\_TREG\_24H\_CULTURE\_UP |  | 16 | -0.31 | -1.31 | 0.164 | 0.391 | 1.000 | 498 | tags=63%, list=35%, signal=95% |
| 927 | GSE5542\_UNTREATED\_VS\_IFNA\_AND\_IFNG\_TREATED\_EPITHELIAL\_CELLS\_6H\_DN |  | 25 | -0.27 | -1.31 | 0.157 | 0.390 | 1.000 | 379 | tags=44%, list=27%, signal=59% |
| 928 | GSE32986\_CURDLAN\_HIGHDOSE\_VS\_GMCSF\_AND\_CURDLAN\_HIGHDOSE\_STIM\_DC\_UP |  | 25 | -0.26 | -1.31 | 0.157 | 0.390 | 1.000 | 429 | tags=56%, list=30%, signal=79% |
| 929 | GSE14000\_UNSTIM\_VS\_4H\_LPS\_DC\_TRANSLATED\_RNA\_DN |  | 27 | -0.26 | -1.31 | 0.126 | 0.391 | 1.000 | 563 | tags=67%, list=40%, signal=109% |
| 930 | GSE32986\_GMCSF\_VS\_GMCSF\_AND\_CURDLAN\_LOWDOSE\_STIM\_DC\_DN |  | 26 | -0.26 | -1.31 | 0.144 | 0.390 | 1.000 | 322 | tags=46%, list=23%, signal=59% |
| 931 | GSE17301\_IFNA2\_VS\_IFNA5\_STIM\_ACD3\_ACD28\_ACT\_CD8\_TCELL\_UP |  | 16 | -0.32 | -1.31 | 0.167 | 0.390 | 1.000 | 252 | tags=44%, list=18%, signal=53% |
| 932 | GSE15930\_NAIVE\_VS\_24H\_IN\_VITRO\_STIM\_CD8\_TCELL\_UP |  | 15 | -0.32 | -1.31 | 0.174 | 0.391 | 1.000 | 306 | tags=47%, list=22%, signal=59% |
| 933 | GSE15324\_ELF4\_KO\_VS\_WT\_NAIVE\_CD8\_TCELL\_DN |  | 18 | -0.30 | -1.31 | 0.176 | 0.392 | 1.000 | 561 | tags=72%, list=40%, signal=118% |
| 934 | GSE37301\_MULTIPOTENT\_PROGENITOR\_VS\_CD4\_TCELL\_DN |  | 15 | -0.31 | -1.31 | 0.136 | 0.392 | 1.000 | 973 | tags=100%, list=69%, signal=319% |
| 935 | GSE17721\_POLYIC\_VS\_PAM3CSK4\_4H\_BMDC\_UP |  | 19 | -0.30 | -1.31 | 0.163 | 0.392 | 1.000 | 513 | tags=63%, list=36%, signal=98% |
| 936 | GSE40274\_CTRL\_VS\_EOS\_TRANSDUCED\_ACTIVATED\_CD4\_TCELL\_UP |  | 15 | -0.32 | -1.31 | 0.150 | 0.391 | 1.000 | 30 | tags=20%, list=2%, signal=20% |
| 937 | GSE27859\_DC\_VS\_CD11C\_INT\_F480\_INT\_DC\_UP |  | 18 | -0.30 | -1.31 | 0.148 | 0.392 | 1.000 | 504 | tags=72%, list=36%, signal=111% |
| 938 | GSE7568\_CTRL\_VS\_3H\_TGFB\_TREATED\_MACROPHAGES\_WITH\_IL4\_AND\_DEXAMETHASONE\_DN |  | 19 | -0.29 | -1.31 | 0.157 | 0.392 | 1.000 | 105 | tags=21%, list=7%, signal=22% |
| 939 | GSE18804\_SPLEEN\_MACROPHAGE\_VS\_TUMORAL\_MACROPHAGE\_DN |  | 18 | -0.31 | -1.31 | 0.184 | 0.392 | 1.000 | 21 | tags=17%, list=1%, signal=17% |
| 940 | GSE21063\_WT\_VS\_NFATC1\_KO\_3H\_ANTI\_IGM\_STIM\_BCELL\_DN |  | 17 | -0.30 | -1.31 | 0.152 | 0.392 | 1.000 | 329 | tags=47%, list=23%, signal=61% |
| 941 | GSE24726\_WT\_VS\_E2\_2\_KO\_PDC\_DN |  | 26 | -0.25 | -1.31 | 0.144 | 0.392 | 1.000 | 147 | tags=23%, list=10%, signal=25% |
| 942 | GSE36891\_UNSTIM\_VS\_POLYIC\_TLR3\_STIM\_PERITONEAL\_MACROPHAGE\_UP |  | 15 | -0.32 | -1.31 | 0.155 | 0.391 | 1.000 | 373 | tags=53%, list=26%, signal=72% |
| 943 | GSE36888\_UNTREATED\_VS\_IL2\_TREATED\_STAT5\_AB\_KNOCKIN\_TCELL\_2H\_UP |  | 22 | -0.27 | -1.31 | 0.157 | 0.391 | 1.000 | 279 | tags=41%, list=20%, signal=50% |
| 944 | GSE27786\_BCELL\_VS\_NKTCELL\_DN |  | 17 | -0.31 | -1.31 | 0.152 | 0.391 | 1.000 | 638 | tags=76%, list=45%, signal=138% |
| 945 | GSE41867\_DAY6\_VS\_DAY15\_LCMV\_ARMSTRONG\_EFFECTOR\_CD8\_TCELL\_UP |  | 16 | -0.31 | -1.31 | 0.160 | 0.394 | 1.000 | 107 | tags=25%, list=8%, signal=27% |
| 946 | GSE37534\_UNTREATED\_VS\_ROSIGLITAZONE\_TREATED\_CD4\_TCELL\_PPARG1\_AND\_FOXP3\_TRASDUCED\_DN |  | 30 | -0.25 | -1.31 | 0.134 | 0.394 | 1.000 | 373 | tags=47%, list=26%, signal=62% |
| 947 | GSE7831\_1H\_VS\_4H\_INFLUENZA\_STIM\_PDC\_DN |  | 17 | -0.31 | -1.30 | 0.150 | 0.395 | 1.000 | 395 | tags=59%, list=28%, signal=81% |
| 948 | GSE22886\_NAIVE\_VS\_IGM\_MEMORY\_BCELL\_UP |  | 27 | -0.25 | -1.30 | 0.150 | 0.395 | 1.000 | 140 | tags=26%, list=10%, signal=28% |
| 949 | GSE40274\_FOXP3\_VS\_FOXP3\_AND\_LEF1\_TRANSDUCED\_ACTIVATED\_CD4\_TCELL\_UP |  | 21 | -0.28 | -1.30 | 0.180 | 0.395 | 1.000 | 269 | tags=38%, list=19%, signal=46% |
| 950 | GSE36392\_TYPE\_2\_MYELOID\_VS\_EOSINOPHIL\_IL25\_TREATED\_LUNG\_DN |  | 20 | -0.28 | -1.30 | 0.167 | 0.395 | 1.000 | 712 | tags=80%, list=50%, signal=159% |
| 951 | GSE8515\_CTRL\_VS\_IL6\_4H\_STIM\_MAC\_UP |  | 18 | -0.29 | -1.30 | 0.156 | 0.395 | 1.000 | 270 | tags=39%, list=19%, signal=47% |
| 952 | GSE17974\_IL4\_AND\_ANTI\_IL12\_VS\_UNTREATED\_6H\_ACT\_CD4\_TCELL\_DN |  | 24 | -0.27 | -1.30 | 0.156 | 0.394 | 1.000 | 294 | tags=42%, list=21%, signal=52% |
| 953 | GSE6092\_B\_BURGDOFERI\_VS\_B\_BURGDORFERI\_AND\_IFNG\_STIM\_ENDOTHELIAL\_CELL\_DN |  | 25 | -0.26 | -1.30 | 0.163 | 0.397 | 1.000 | 253 | tags=40%, list=18%, signal=48% |
| 954 | GSE29618\_BCELL\_VS\_MONOCYTE\_DN |  | 23 | -0.28 | -1.30 | 0.160 | 0.398 | 1.000 | 269 | tags=39%, list=19%, signal=48% |
| 955 | GSE32901\_TH1\_VS\_TH17\_NEG\_CD4\_TCELL\_DN |  | 18 | -0.30 | -1.30 | 0.177 | 0.398 | 1.000 | 387 | tags=56%, list=27%, signal=76% |
| 956 | GSE24634\_TREG\_VS\_TCONV\_POST\_DAY7\_IL4\_CONVERSION\_DN |  | 23 | -0.28 | -1.30 | 0.165 | 0.401 | 1.000 | 172 | tags=26%, list=12%, signal=29% |
| 957 | GSE1432\_CTRL\_VS\_IFNG\_24H\_MICROGLIA\_UP |  | 23 | -0.26 | -1.30 | 0.174 | 0.404 | 1.000 | 490 | tags=61%, list=35%, signal=92% |
| 958 | GSE2770\_UNTREATED\_VS\_IL12\_TREATED\_ACT\_CD4\_TCELL\_2H\_UP |  | 18 | -0.30 | -1.30 | 0.160 | 0.405 | 1.000 | 298 | tags=44%, list=21%, signal=56% |
| 959 | GSE15930\_NAIVE\_VS\_72H\_IN\_VITRO\_STIM\_IL12\_CD8\_TCELL\_DN |  | 23 | -0.26 | -1.29 | 0.144 | 0.405 | 1.000 | 576 | tags=70%, list=41%, signal=116% |
| 960 | GSE17721\_CTRL\_VS\_GARDIQUIMOD\_8H\_BMDC\_UP |  | 16 | -0.31 | -1.29 | 0.176 | 0.406 | 1.000 | 593 | tags=75%, list=42%, signal=128% |
| 961 | GSE17721\_CTRL\_VS\_LPS\_1H\_BMDC\_DN |  | 23 | -0.27 | -1.29 | 0.132 | 0.406 | 1.000 | 593 | tags=74%, list=42%, signal=125% |
| 962 | GSE360\_HIGH\_DOSE\_B\_MALAYI\_VS\_M\_TUBERCULOSIS\_DC\_DN |  | 22 | -0.27 | -1.29 | 0.159 | 0.407 | 1.000 | 562 | tags=73%, list=40%, signal=119% |
| 963 | GSE17721\_4\_VS\_24H\_CPG\_BMDC\_DN |  | 19 | -0.29 | -1.29 | 0.150 | 0.407 | 1.000 | 79 | tags=21%, list=6%, signal=22% |
| 964 | GSE8621\_UNSTIM\_VS\_LPS\_PRIMED\_AND\_LPS\_STIM\_MACROPHAGE\_DN |  | 21 | -0.27 | -1.29 | 0.177 | 0.407 | 1.000 | 395 | tags=52%, list=28%, signal=72% |
| 965 | GSE17721\_0.5H\_VS\_12H\_CPG\_BMDC\_DN |  | 29 | -0.24 | -1.29 | 0.163 | 0.408 | 1.000 | 501 | tags=59%, list=36%, signal=89% |
| 966 | GSE15330\_LYMPHOID\_MULTIPOTENT\_VS\_PRO\_BCELL\_DN |  | 25 | -0.25 | -1.29 | 0.159 | 0.409 | 1.000 | 373 | tags=44%, list=26%, signal=59% |
| 967 | GSE15330\_HSC\_VS\_MEGAKARYOCYTE\_ERYTHROID\_PROGENITOR\_IKAROS\_KO\_DN |  | 17 | -0.31 | -1.29 | 0.175 | 0.410 | 1.000 | 504 | tags=65%, list=36%, signal=99% |
| 968 | GSE40443\_INDUCED\_VS\_TOTAL\_TREG\_UP |  | 22 | -0.27 | -1.29 | 0.167 | 0.412 | 1.000 | 528 | tags=64%, list=37%, signal=100% |
| 969 | GSE2770\_TGFB\_AND\_IL4\_ACT\_VS\_ACT\_CD4\_TCELL\_2H\_DN |  | 25 | -0.26 | -1.29 | 0.170 | 0.412 | 1.000 | 458 | tags=56%, list=32%, signal=81% |
| 970 | GSE42021\_TCONV\_PLN\_VS\_CD24INT\_TCONV\_THYMUS\_UP |  | 18 | -0.30 | -1.29 | 0.170 | 0.413 | 1.000 | 319 | tags=44%, list=23%, signal=57% |
| 971 | GSE11057\_NAIVE\_VS\_CENT\_MEMORY\_CD4\_TCELL\_UP |  | 15 | -0.31 | -1.29 | 0.156 | 0.414 | 1.000 | 430 | tags=67%, list=30%, signal=95% |
| 972 | GSE8685\_IL2\_ACT\_IL2\_STARVED\_VS\_IL21\_ACT\_IL2\_STARVED\_CD4\_TCELL\_UP |  | 21 | -0.28 | -1.29 | 0.161 | 0.414 | 1.000 | 496 | tags=62%, list=35%, signal=94% |
| 973 | GSE3982\_BASOPHIL\_VS\_TH2\_DN |  | 27 | -0.25 | -1.29 | 0.183 | 0.414 | 1.000 | 931 | tags=93%, list=66%, signal=267% |
| 974 | GSE29949\_DC\_BRAIN\_VS\_MONOCYTE\_BONE\_MARROW\_DN |  | 18 | -0.29 | -1.28 | 0.185 | 0.414 | 1.000 | 270 | tags=39%, list=19%, signal=47% |
| 975 | GSE41176\_WT\_VS\_TAK1\_KO\_UNSTIM\_BCELL\_UP |  | 17 | -0.30 | -1.28 | 0.205 | 0.419 | 1.000 | 528 | tags=65%, list=37%, signal=102% |
| 976 | GSE39820\_TGFBETA1\_IL6\_VS\_TGFBETA1\_IL6\_IL23A\_TREATED\_CD4\_TCELL\_UP |  | 20 | -0.28 | -1.28 | 0.179 | 0.424 | 1.000 | 542 | tags=70%, list=38%, signal=112% |
| 977 | GSE5589\_LPS\_VS\_LPS\_AND\_IL6\_STIM\_IL6\_KO\_MACROPHAGE\_45MIN\_UP |  | 16 | -0.30 | -1.28 | 0.168 | 0.424 | 1.000 | 132 | tags=31%, list=9%, signal=34% |
| 978 | GSE22432\_MULTIPOTENT\_VS\_COMMON\_DC\_PROGENITOR\_DN |  | 16 | -0.30 | -1.28 | 0.163 | 0.424 | 1.000 | 334 | tags=50%, list=24%, signal=65% |
| 979 | GSE3982\_MAC\_VS\_BASOPHIL\_DN |  | 31 | -0.24 | -1.28 | 0.155 | 0.424 | 1.000 | 187 | tags=29%, list=13%, signal=33% |
| 980 | GSE45739\_UNSTIM\_VS\_ACD3\_ACD28\_STIM\_WT\_CD4\_TCELL\_UP |  | 18 | -0.29 | -1.28 | 0.179 | 0.425 | 1.000 | 372 | tags=56%, list=26%, signal=74% |
| 981 | GSE13411\_PLASMA\_CELL\_VS\_MEMORY\_BCELL\_DN |  | 24 | -0.26 | -1.27 | 0.175 | 0.428 | 1.000 | 398 | tags=54%, list=28%, signal=74% |
| 982 | GSE26495\_NAIVE\_VS\_PD1LOW\_CD8\_TCELL\_DN |  | 20 | -0.28 | -1.27 | 0.185 | 0.428 | 1.000 | 568 | tags=70%, list=40%, signal=116% |
| 983 | GSE40666\_UNTREATED\_VS\_IFNA\_STIM\_STAT1\_KO\_CD8\_TCELL\_90MIN\_UP |  | 15 | -0.31 | -1.27 | 0.208 | 0.428 | 1.000 | 456 | tags=60%, list=32%, signal=88% |
| 984 | GSE13493\_DP\_VS\_CD4INTCD8POS\_THYMOCYTE\_UP |  | 23 | -0.26 | -1.27 | 0.169 | 0.430 | 1.000 | 441 | tags=57%, list=31%, signal=81% |
| 985 | GSE3982\_EOSINOPHIL\_VS\_NKCELL\_UP |  | 25 | -0.26 | -1.27 | 0.181 | 0.432 | 1.000 | 497 | tags=64%, list=35%, signal=97% |
| 986 | GSE17186\_CD21LOW\_VS\_CD21HIGH\_TRANSITIONAL\_BCELL\_UP |  | 29 | -0.24 | -1.27 | 0.177 | 0.433 | 1.000 | 543 | tags=62%, list=38%, signal=99% |
| 987 | GSE25123\_CTRL\_VS\_ROSIGLITAZONE\_STIM\_PPARG\_KO\_MACROPHAGE\_DN |  | 22 | -0.27 | -1.27 | 0.166 | 0.434 | 1.000 | 352 | tags=50%, list=25%, signal=66% |
| 988 | GSE22886\_UNSTIM\_VS\_IL15\_STIM\_NKCELL\_UP |  | 23 | -0.27 | -1.27 | 0.164 | 0.434 | 1.000 | 256 | tags=39%, list=18%, signal=47% |
| 989 | GSE29618\_PRE\_VS\_DAY7\_POST\_LAIV\_FLU\_VACCINE\_PDC\_UP |  | 16 | -0.30 | -1.27 | 0.168 | 0.433 | 1.000 | 410 | tags=56%, list=29%, signal=78% |
| 990 | GSE16755\_CTRL\_VS\_IFNA\_TREATED\_MAC\_DN |  | 21 | -0.27 | -1.27 | 0.173 | 0.433 | 1.000 | 281 | tags=38%, list=20%, signal=47% |
| 991 | GSE37533\_PPARG1\_FOXP3\_VS\_PPARG2\_FOXP3\_TRANSDUCED\_CD4\_TCELL\_PIOGLITAZONE\_TREATED\_DN |  | 30 | -0.23 | -1.27 | 0.171 | 0.433 | 1.000 | 276 | tags=37%, list=20%, signal=45% |
| 992 | GSE6674\_ANTI\_IGM\_VS\_ANTI\_IGM\_AND\_CPG\_STIM\_BCELL\_UP |  | 24 | -0.25 | -1.27 | 0.195 | 0.433 | 1.000 | 382 | tags=46%, list=27%, signal=62% |
| 993 | GSE29618\_PRE\_VS\_DAY7\_FLU\_VACCINE\_MONOCYTE\_UP |  | 16 | -0.30 | -1.27 | 0.174 | 0.435 | 1.000 | 150 | tags=31%, list=11%, signal=35% |
| 994 | GSE41867\_NAIVE\_VS\_DAY8\_LCMV\_CLONE13\_EFFECTOR\_CD8\_TCELL\_UP |  | 19 | -0.28 | -1.27 | 0.169 | 0.435 | 1.000 | 251 | tags=42%, list=18%, signal=51% |
| 995 | GSE36078\_WT\_VS\_IL1R\_KO\_LUNG\_DC\_AFTER\_AD5\_INF\_DN |  | 21 | -0.27 | -1.27 | 0.169 | 0.435 | 1.000 | 442 | tags=57%, list=31%, signal=82% |
| 996 | GSE37301\_CD4\_TCELL\_VS\_GRANULOCYTE\_MONOCYTE\_PROGENITOR\_DN |  | 15 | -0.31 | -1.27 | 0.171 | 0.435 | 1.000 | 387 | tags=60%, list=27%, signal=82% |
| 997 | GSE22886\_NAIVE\_CD4\_TCELL\_VS\_48H\_ACT\_TH2\_UP |  | 17 | -0.29 | -1.27 | 0.186 | 0.436 | 1.000 | 443 | tags=65%, list=31%, signal=93% |
| 998 | GSE12392\_IFNAR\_KO\_VS\_IFNB\_KO\_CD8\_NEG\_SPLEEN\_DC\_DN |  | 30 | -0.23 | -1.27 | 0.176 | 0.436 | 1.000 | 393 | tags=43%, list=28%, signal=59% |
| 999 | GSE43700\_UNTREATED\_VS\_IL10\_TREATED\_PBMC\_UP |  | 18 | -0.28 | -1.27 | 0.193 | 0.436 | 1.000 | 276 | tags=44%, list=20%, signal=55% |
| 1000 | GSE29618\_MONOCYTE\_VS\_PDC\_DAY7\_FLU\_VACCINE\_UP |  | 19 | -0.28 | -1.26 | 0.178 | 0.436 | 1.000 | 562 | tags=68%, list=40%, signal=112% |
| 1001 | GSE19888\_ADENOSINE\_A3R\_ACT\_VS\_TCELL\_MEMBRANES\_ACT\_AND\_A3R\_INH\_PRETREAT\_IN\_MAST\_CELL\_DN |  | 22 | -0.26 | -1.26 | 0.192 | 0.436 | 1.000 | 298 | tags=36%, list=21%, signal=45% |
| 1002 | GSE42724\_MEMORY\_BCELL\_VS\_PLASMABLAST\_DN |  | 25 | -0.25 | -1.26 | 0.168 | 0.436 | 1.000 | 242 | tags=36%, list=17%, signal=43% |
| 1003 | GSE27786\_LSK\_VS\_CD8\_TCELL\_DN |  | 15 | -0.31 | -1.26 | 0.197 | 0.436 | 1.000 | 393 | tags=53%, list=28%, signal=73% |
| 1004 | GSE5542\_IFNG\_VS\_IFNA\_AND\_IFNG\_TREATED\_EPITHELIAL\_CELLS\_24H\_DN |  | 18 | -0.29 | -1.26 | 0.181 | 0.435 | 1.000 | 324 | tags=39%, list=23%, signal=50% |
| 1005 | GSE30971\_WBP7\_HET\_VS\_KO\_MACROPHAGE\_UP |  | 15 | -0.32 | -1.26 | 0.194 | 0.437 | 1.000 | 317 | tags=47%, list=22%, signal=60% |
| 1006 | GSE19888\_CTRL\_VS\_T\_CELL\_MEMBRANES\_ACT\_MAST\_CELL\_DN |  | 17 | -0.29 | -1.26 | 0.192 | 0.437 | 1.000 | 346 | tags=53%, list=25%, signal=69% |
| 1007 | GSE12845\_NAIVE\_VS\_DARKZONE\_GC\_TONSIL\_BCELL\_UP |  | 20 | -0.27 | -1.26 | 0.202 | 0.437 | 1.000 | 191 | tags=30%, list=14%, signal=34% |
| 1008 | GSE29164\_DAY3\_VS\_DAY7\_CD8\_TCELL\_AND\_IL12\_TREATED\_MELANOMA\_UP |  | 24 | -0.26 | -1.26 | 0.189 | 0.438 | 1.000 | 125 | tags=29%, list=9%, signal=31% |
| 1009 | GSE17721\_POLYIC\_VS\_PAM3CSK4\_8H\_BMDC\_UP |  | 23 | -0.26 | -1.26 | 0.150 | 0.440 | 1.000 | 266 | tags=39%, list=19%, signal=47% |
| 1010 | GSE22601\_DOUBLE\_NEGATIVE\_VS\_CD4\_SINGLE\_POSITIVE\_THYMOCYTE\_DN |  | 18 | -0.29 | -1.26 | 0.181 | 0.439 | 1.000 | 276 | tags=44%, list=20%, signal=55% |
| 1011 | GSE22025\_PROGESTERONE\_VS\_TGFB1\_AND\_PROGESTERONE\_TREATED\_CD4\_TCELL\_UP |  | 17 | -0.29 | -1.26 | 0.204 | 0.440 | 1.000 | 423 | tags=53%, list=30%, signal=75% |
| 1012 | GSE32901\_TH1\_VS\_TH17\_ENRICHED\_CD4\_TCELL\_DN |  | 16 | -0.31 | -1.26 | 0.191 | 0.440 | 1.000 | 984 | tags=100%, list=70%, signal=327% |
| 1013 | GSE23398\_WT\_VS\_IL2\_KO\_CD4\_TCELL\_SCURFY\_MOUSE\_UP |  | 17 | -0.29 | -1.26 | 0.202 | 0.440 | 1.000 | 228 | tags=35%, list=16%, signal=42% |
| 1014 | GSE2770\_TGFB\_AND\_IL4\_VS\_IL12\_TREATED\_ACT\_CD4\_TCELL\_48H\_UP |  | 23 | -0.26 | -1.26 | 0.185 | 0.443 | 1.000 | 500 | tags=61%, list=35%, signal=93% |
| 1015 | GSE3982\_EOSINOPHIL\_VS\_MAC\_UP |  | 27 | -0.25 | -1.26 | 0.194 | 0.443 | 1.000 | 492 | tags=63%, list=35%, signal=95% |
| 1016 | GSE16385\_ROSIGLITAZONE\_IL4\_VS\_ROSIGLITAZONE\_ALONE\_STIM\_MACROPHAGE\_UP |  | 19 | -0.28 | -1.25 | 0.199 | 0.446 | 1.000 | 125 | tags=26%, list=9%, signal=28% |
| 1017 | GSE1448\_ANTI\_VALPHA2\_VS\_VBETA5\_DP\_THYMOCYTE\_UP |  | 20 | -0.28 | -1.25 | 0.194 | 0.448 | 1.000 | 374 | tags=45%, list=27%, signal=60% |
| 1018 | GSE46606\_IRF4\_KO\_VS\_WT\_CD40L\_IL2\_IL5\_1DAY\_STIMULATED\_BCELL\_UP |  | 19 | -0.28 | -1.25 | 0.182 | 0.451 | 1.000 | 469 | tags=58%, list=33%, signal=86% |
| 1019 | GSE24634\_NAIVE\_CD4\_TCELL\_VS\_DAY7\_IL4\_CONV\_TREG\_UP |  | 24 | -0.25 | -1.25 | 0.202 | 0.452 | 1.000 | 513 | tags=63%, list=36%, signal=97% |
| 1020 | GSE31082\_CD4\_VS\_CD8\_SP\_THYMOCYTE\_DN |  | 22 | -0.27 | -1.25 | 0.217 | 0.452 | 1.000 | 163 | tags=27%, list=12%, signal=30% |
| 1021 | GSE41978\_KLRG1\_HIGH\_VS\_LOW\_EFFECTOR\_CD8\_TCELL\_UP |  | 25 | -0.25 | -1.25 | 0.167 | 0.453 | 1.000 | 341 | tags=44%, list=24%, signal=57% |
| 1022 | GSE11057\_NAIVE\_VS\_MEMORY\_CD4\_TCELL\_DN |  | 22 | -0.27 | -1.25 | 0.185 | 0.454 | 1.000 | 317 | tags=36%, list=22%, signal=46% |
| 1023 | GSE29617\_DAY3\_VS\_DAY7\_TIV\_FLU\_VACCINE\_PBMC\_2008\_UP |  | 15 | -0.31 | -1.25 | 0.180 | 0.453 | 1.000 | 225 | tags=33%, list=16%, signal=39% |
| 1024 | GSE7831\_CPG\_VS\_INFLUENZA\_STIM\_PDC\_1H\_UP |  | 18 | -0.29 | -1.25 | 0.204 | 0.453 | 1.000 | 51 | tags=17%, list=4%, signal=17% |
| 1025 | GSE3565\_CTRL\_VS\_LPS\_INJECTED\_DUSP1\_KO\_SPLENOCYTES\_DN |  | 24 | -0.25 | -1.25 | 0.183 | 0.453 | 1.000 | 159 | tags=25%, list=11%, signal=28% |
| 1026 | GSE37301\_GRANULOCYTE\_MONOCYTE\_PROGENITOR\_VS\_RAG2\_KO\_NK\_CELL\_DN |  | 27 | -0.24 | -1.25 | 0.234 | 0.453 | 1.000 | 916 | tags=93%, list=65%, signal=259% |
| 1027 | GSE42724\_NAIVE\_BCELL\_VS\_PLASMABLAST\_DN |  | 20 | -0.27 | -1.25 | 0.220 | 0.455 | 1.000 | 326 | tags=45%, list=23%, signal=58% |
| 1028 | GSE22432\_MULTIPOTENT\_PROGENITOR\_VS\_PDC\_DN |  | 19 | -0.27 | -1.25 | 0.197 | 0.455 | 1.000 | 150 | tags=26%, list=11%, signal=29% |
| 1029 | GSE27786\_LIN\_NEG\_VS\_NEUTROPHIL\_DN |  | 16 | -0.31 | -1.25 | 0.212 | 0.457 | 1.000 | 563 | tags=75%, list=40%, signal=123% |
| 1030 | GSE21927\_SPLENIC\_C26GM\_TUMOROUS\_VS\_4T1\_TUMOR\_MONOCYTES\_UP |  | 18 | -0.28 | -1.24 | 0.199 | 0.457 | 1.000 | 408 | tags=56%, list=29%, signal=77% |
| 1031 | GSE37416\_CTRL\_VS\_6H\_F\_TULARENSIS\_LVS\_NEUTROPHIL\_DN |  | 24 | -0.25 | -1.24 | 0.193 | 0.458 | 1.000 | 492 | tags=58%, list=35%, signal=88% |
| 1032 | GSE23502\_BM\_VS\_COLON\_TUMOR\_HDC\_KO\_MYELOID\_DERIVED\_SUPPRESSOR\_CELL\_DN |  | 29 | -0.24 | -1.24 | 0.187 | 0.458 | 1.000 | 219 | tags=28%, list=16%, signal=32% |
| 1033 | GSE17721\_CPG\_VS\_GARDIQUIMOD\_24H\_BMDC\_DN |  | 20 | -0.27 | -1.24 | 0.201 | 0.459 | 1.000 | 737 | tags=85%, list=52%, signal=175% |
| 1034 | GSE37416\_0H\_VS\_24H\_F\_TULARENSIS\_LVS\_NEUTROPHIL\_UP |  | 23 | -0.26 | -1.24 | 0.212 | 0.460 | 1.000 | 437 | tags=52%, list=31%, signal=74% |
| 1035 | GSE5589\_IL6\_KO\_VS\_IL10\_KO\_LPS\_AND\_IL6\_STIM\_MACROPHAGE\_45MIN\_DN |  | 22 | -0.25 | -1.24 | 0.197 | 0.460 | 1.000 | 269 | tags=41%, list=19%, signal=50% |
| 1036 | GSE27670\_CTRL\_VS\_LMP1\_TRANSDUCED\_GC\_BCELL\_UP |  | 20 | -0.27 | -1.24 | 0.200 | 0.460 | 1.000 | 564 | tags=70%, list=40%, signal=115% |
| 1037 | GSE5589\_LPS\_VS\_LPS\_AND\_IL10\_STIM\_MACROPHAGE\_180MIN\_DN |  | 23 | -0.25 | -1.24 | 0.211 | 0.461 | 1.000 | 353 | tags=43%, list=25%, signal=57% |
| 1038 | GSE29617\_CTRL\_VS\_DAY7\_TIV\_FLU\_VACCINE\_PBMC\_2008\_UP |  | 23 | -0.26 | -1.24 | 0.216 | 0.461 | 1.000 | 562 | tags=74%, list=40%, signal=121% |
| 1039 | GSE17721\_LPS\_VS\_POLYIC\_2H\_BMDC\_UP |  | 17 | -0.29 | -1.24 | 0.193 | 0.461 | 1.000 | 338 | tags=47%, list=24%, signal=61% |
| 1040 | GSE14769\_UNSTIM\_VS\_360MIN\_LPS\_BMDM\_DN |  | 22 | -0.26 | -1.24 | 0.207 | 0.460 | 1.000 | 468 | tags=55%, list=33%, signal=80% |
| 1041 | GSE360\_CTRL\_VS\_M\_TUBERCULOSIS\_DC\_DN |  | 26 | -0.24 | -1.24 | 0.205 | 0.461 | 1.000 | 183 | tags=27%, list=13%, signal=30% |
| 1042 | GSE7852\_LN\_VS\_FAT\_TCONV\_DN |  | 28 | -0.24 | -1.24 | 0.183 | 0.461 | 1.000 | 104 | tags=18%, list=7%, signal=19% |
| 1043 | GSE13306\_TREG\_VS\_TCONV\_SPLEEN\_DN |  | 21 | -0.27 | -1.24 | 0.188 | 0.460 | 1.000 | 280 | tags=43%, list=20%, signal=53% |
| 1044 | GSE45365\_NK\_CELL\_VS\_BCELL\_MCMV\_INFECTION\_DN |  | 20 | -0.27 | -1.24 | 0.231 | 0.460 | 1.000 | 564 | tags=70%, list=40%, signal=115% |
| 1045 | GSE11818\_WT\_VS\_DICER\_KO\_TREG\_DN |  | 18 | -0.28 | -1.24 | 0.209 | 0.461 | 1.000 | 250 | tags=39%, list=18%, signal=47% |
| 1046 | GSE7460\_TCONV\_VS\_TREG\_LN\_UP |  | 22 | -0.26 | -1.24 | 0.195 | 0.464 | 1.000 | 433 | tags=55%, list=31%, signal=77% |
| 1047 | GSE36888\_STAT5\_AB\_KNOCKIN\_VS\_WT\_TCELL\_IL2\_TREATED\_17H\_DN |  | 22 | -0.25 | -1.24 | 0.195 | 0.463 | 1.000 | 528 | tags=68%, list=37%, signal=107% |
| 1048 | GSE29618\_PRE\_VS\_DAY7\_POST\_TIV\_FLU\_VACCINE\_MDC\_UP |  | 30 | -0.24 | -1.24 | 0.189 | 0.465 | 1.000 | 137 | tags=20%, list=10%, signal=22% |
| 1049 | GSE15330\_WT\_VS\_IKAROS\_KO\_HSC\_UP |  | 16 | -0.29 | -1.24 | 0.210 | 0.465 | 1.000 | 104 | tags=25%, list=7%, signal=27% |
| 1050 | GSE41867\_NAIVE\_VS\_DAY30\_LCMV\_CLONE13\_EXHAUSTED\_CD8\_TCELL\_DN |  | 24 | -0.26 | -1.23 | 0.216 | 0.466 | 1.000 | 248 | tags=33%, list=18%, signal=40% |
| 1051 | GSE22025\_UNTREATED\_VS\_PROGESTERONE\_TREATED\_CD4\_TCELL\_DN |  | 26 | -0.24 | -1.23 | 0.204 | 0.467 | 1.000 | 313 | tags=42%, list=22%, signal=53% |
| 1052 | GSE32164\_RESTING\_DIFFERENTIATED\_VS\_CMYC\_INHIBITED\_MACROPHAGE\_DN |  | 19 | -0.27 | -1.23 | 0.206 | 0.469 | 1.000 | 253 | tags=32%, list=18%, signal=38% |
| 1053 | GSE2405\_S\_AUREUS\_VS\_UNTREATED\_NEUTROPHIL\_DN |  | 16 | -0.29 | -1.23 | 0.206 | 0.470 | 1.000 | 709 | tags=88%, list=50%, signal=174% |
| 1054 | GSE5589\_LPS\_VS\_LPS\_AND\_IL10\_STIM\_IL6\_KO\_MACROPHAGE\_45MIN\_DN |  | 21 | -0.27 | -1.23 | 0.222 | 0.470 | 1.000 | 498 | tags=67%, list=35%, signal=101% |
| 1055 | GSE17301\_CTRL\_VS\_48H\_ACD3\_ACD28\_IFNA2\_STIM\_CD8\_TCELL\_UP |  | 23 | -0.25 | -1.23 | 0.214 | 0.470 | 1.000 | 188 | tags=30%, list=13%, signal=35% |
| 1056 | GSE26495\_NAIVE\_VS\_PD1LOW\_CD8\_TCELL\_UP |  | 18 | -0.28 | -1.23 | 0.208 | 0.470 | 1.000 | 476 | tags=61%, list=34%, signal=91% |
| 1057 | GSE17812\_WT\_VS\_THPOK\_KO\_MEMORY\_CD8\_TCELL\_DN |  | 20 | -0.28 | -1.23 | 0.201 | 0.469 | 1.000 | 939 | tags=95%, list=67%, signal=280% |
| 1058 | GSE40274\_FOXP3\_VS\_FOXP3\_AND\_PBX1\_TRANSDUCED\_ACTIVATED\_CD4\_TCELL\_UP |  | 19 | -0.28 | -1.23 | 0.202 | 0.470 | 1.000 | 349 | tags=47%, list=25%, signal=62% |
| 1059 | GSE13522\_WT\_VS\_IFNAR\_KO\_SKIN\_DN |  | 19 | -0.28 | -1.23 | 0.216 | 0.469 | 1.000 | 542 | tags=74%, list=38%, signal=118% |
| 1060 | GSE19401\_NAIVE\_VS\_IMMUNIZED\_MOUSE\_PLN\_FOLLICULAR\_DC\_UP |  | 32 | -0.23 | -1.23 | 0.186 | 0.470 | 1.000 | 548 | tags=66%, list=39%, signal=105% |
| 1061 | GSE17301\_IFNA2\_VS\_IFNA5\_STIM\_ACD3\_ACD28\_ACT\_CD8\_TCELL\_DN |  | 18 | -0.28 | -1.23 | 0.217 | 0.470 | 1.000 | 630 | tags=78%, list=45%, signal=139% |
| 1062 | GSE22601\_DOUBLE\_POSITIVE\_VS\_CD8\_SINGLE\_POSITIVE\_THYMOCYTE\_UP |  | 17 | -0.29 | -1.23 | 0.217 | 0.470 | 1.000 | 225 | tags=35%, list=16%, signal=41% |
| 1063 | GSE36392\_EOSINOPHIL\_VS\_NEUTROPHIL\_IL25\_TREATED\_LUNG\_UP |  | 24 | -0.25 | -1.23 | 0.214 | 0.470 | 1.000 | 527 | tags=63%, list=37%, signal=98% |
| 1064 | GSE22886\_NAIVE\_BCELL\_VS\_NEUTROPHIL\_DN |  | 15 | -0.30 | -1.23 | 0.199 | 0.470 | 1.000 | 547 | tags=67%, list=39%, signal=108% |
| 1065 | GSE15930\_STIM\_VS\_STIM\_AND\_IL12\_48H\_CD8\_T\_CELL\_DN |  | 24 | -0.25 | -1.23 | 0.211 | 0.470 | 1.000 | 393 | tags=50%, list=28%, signal=68% |
| 1066 | GSE17721\_PAM3CSK4\_VS\_CPG\_1H\_BMDC\_DN |  | 18 | -0.28 | -1.23 | 0.211 | 0.470 | 1.000 | 548 | tags=78%, list=39%, signal=126% |
| 1067 | GSE11961\_FOLLICULAR\_BCELL\_VS\_MEMORY\_BCELL\_DAY40\_DN |  | 18 | -0.27 | -1.23 | 0.223 | 0.472 | 1.000 | 238 | tags=33%, list=17%, signal=40% |
| 1068 | GSE27786\_CD4\_TCELL\_VS\_MONO\_MAC\_UP |  | 22 | -0.26 | -1.23 | 0.201 | 0.473 | 1.000 | 225 | tags=32%, list=16%, signal=37% |
| 1069 | GSE1925\_CTRL\_VS\_IFNG\_PRIMED\_MACROPHAGE\_24H\_IFNG\_STIM\_UP |  | 20 | -0.27 | -1.23 | 0.199 | 0.473 | 1.000 | 158 | tags=25%, list=11%, signal=28% |
| 1070 | GSE18281\_SUBCAPSULAR\_VS\_PERIMEDULLARY\_CORTICAL\_REGION\_OF\_THYMUS\_UP |  | 15 | -0.30 | -1.23 | 0.201 | 0.473 | 1.000 | 380 | tags=53%, list=27%, signal=72% |
| 1071 | GSE42724\_MEMORY\_VS\_B1\_BCELL\_DN |  | 21 | -0.26 | -1.23 | 0.213 | 0.473 | 1.000 | 576 | tags=71%, list=41%, signal=119% |
| 1072 | GSE5589\_WT\_VS\_IL6\_KO\_LPS\_AND\_IL6\_STIM\_MACROPHAGE\_45MIN\_DN |  | 28 | -0.24 | -1.22 | 0.217 | 0.474 | 1.000 | 480 | tags=61%, list=34%, signal=90% |
| 1073 | GSE16450\_IMMATURE\_VS\_MATURE\_NEURON\_CELL\_LINE\_6H\_IFNA\_STIM\_DN |  | 15 | -0.30 | -1.22 | 0.209 | 0.477 | 1.000 | 460 | tags=60%, list=33%, signal=88% |
| 1074 | GSE9988\_ANTI\_TREM1\_VS\_LOW\_LPS\_MONOCYTE\_UP |  | 29 | -0.23 | -1.22 | 0.204 | 0.477 | 1.000 | 668 | tags=76%, list=47%, signal=141% |
| 1075 | GSE5589\_UNSTIM\_VS\_180MIN\_LPS\_STIM\_MACROPHAGE\_DN |  | 27 | -0.23 | -1.22 | 0.213 | 0.477 | 1.000 | 552 | tags=63%, list=39%, signal=101% |
| 1076 | GSE20715\_0H\_VS\_48H\_OZONE\_TLR4\_KO\_LUNG\_DN |  | 34 | -0.22 | -1.22 | 0.197 | 0.478 | 1.000 | 588 | tags=62%, list=42%, signal=103% |
| 1077 | GSE40274\_CTRL\_VS\_SATB1\_TRANSDUCED\_ACTIVATED\_CD4\_TCELL\_DN |  | 28 | -0.23 | -1.22 | 0.203 | 0.477 | 1.000 | 714 | tags=79%, list=51%, signal=156% |
| 1078 | GSE37605\_FOXP3\_FUSION\_GFP\_VS\_IRES\_GFP\_TREG\_C57BL6\_DN |  | 19 | -0.28 | -1.22 | 0.210 | 0.477 | 1.000 | 238 | tags=32%, list=17%, signal=37% |
| 1079 | GSE17721\_CTRL\_VS\_LPS\_4H\_BMDC\_DN |  | 19 | -0.28 | -1.22 | 0.211 | 0.478 | 1.000 | 219 | tags=37%, list=16%, signal=43% |
| 1080 | GSE14000\_4H\_VS\_16H\_LPS\_DC\_UP |  | 21 | -0.27 | -1.22 | 0.202 | 0.478 | 1.000 | 235 | tags=33%, list=17%, signal=39% |
| 1081 | GSE27786\_BCELL\_VS\_CD4\_TCELL\_UP |  | 18 | -0.28 | -1.22 | 0.232 | 0.479 | 1.000 | 1024 | tags=100%, list=73%, signal=360% |
| 1082 | GSE22432\_UNTREATED\_VS\_TGFB1\_TREATED\_COMMON\_DC\_PROGENITOR\_DN |  | 24 | -0.24 | -1.22 | 0.204 | 0.479 | 1.000 | 320 | tags=42%, list=23%, signal=53% |
| 1083 | GSE9988\_ANTI\_TREM1\_VS\_LPS\_MONOCYTE\_DN |  | 20 | -0.26 | -1.22 | 0.222 | 0.479 | 1.000 | 547 | tags=65%, list=39%, signal=105% |
| 1084 | GSE16385\_ROSIGLITAZONE\_IL4\_VS\_IL4\_ALONE\_STIM\_MACROPHAGE\_12H\_DN |  | 21 | -0.27 | -1.22 | 0.212 | 0.479 | 1.000 | 127 | tags=24%, list=9%, signal=26% |
| 1085 | GSE29618\_BCELL\_VS\_MONOCYTE\_UP |  | 20 | -0.26 | -1.22 | 0.227 | 0.478 | 1.000 | 219 | tags=35%, list=16%, signal=41% |
| 1086 | GSE32986\_UNSTIM\_VS\_GMCSF\_AND\_CURDLAN\_HIGHDOSE\_STIM\_DC\_DN |  | 25 | -0.24 | -1.22 | 0.209 | 0.478 | 1.000 | 220 | tags=36%, list=16%, signal=42% |
| 1087 | GSE17721\_PAM3CSK4\_VS\_CPG\_1H\_BMDC\_UP |  | 20 | -0.26 | -1.22 | 0.226 | 0.478 | 1.000 | 235 | tags=35%, list=17%, signal=41% |
| 1088 | GSE20366\_TREG\_VS\_NAIVE\_CD4\_TCELL\_DEC205\_CONVERSION\_UP |  | 22 | -0.26 | -1.22 | 0.208 | 0.478 | 1.000 | 50 | tags=18%, list=4%, signal=19% |
| 1089 | GSE2128\_C57BL6\_VS\_NOD\_THYMOCYTE\_MIMETOPE\_NEGATIVE\_SELECTION\_DN |  | 25 | -0.24 | -1.22 | 0.196 | 0.478 | 1.000 | 433 | tags=48%, list=31%, signal=68% |
| 1090 | GSE22589\_HIV\_VS\_HIV\_AND\_SIV\_INFECTED\_DC\_UP |  | 15 | -0.30 | -1.22 | 0.227 | 0.478 | 1.000 | 286 | tags=47%, list=20%, signal=58% |
| 1091 | GSE43955\_TH0\_VS\_TGFB\_IL6\_TH17\_ACT\_CD4\_TCELL\_60H\_DN |  | 17 | -0.28 | -1.22 | 0.224 | 0.479 | 1.000 | 733 | tags=82%, list=52%, signal=169% |
| 1092 | GSE37301\_COMMON\_LYMPHOID\_PROGENITOR\_VS\_GRAN\_MONO\_PROGENITOR\_UP |  | 28 | -0.24 | -1.22 | 0.220 | 0.479 | 1.000 | 947 | tags=93%, list=67%, signal=277% |
| 1093 | GSE24634\_TREG\_VS\_TCONV\_POST\_DAY5\_IL4\_CONVERSION\_DN |  | 15 | -0.30 | -1.22 | 0.230 | 0.480 | 1.000 | 398 | tags=53%, list=28%, signal=73% |
| 1094 | GSE23505\_UNTREATED\_VS\_4DAY\_IL6\_IL1\_TREATED\_CD4\_TCELL\_DN |  | 22 | -0.25 | -1.21 | 0.224 | 0.481 | 1.000 | 905 | tags=91%, list=64%, signal=250% |
| 1095 | GSE28737\_FOLLICULAR\_VS\_MARGINAL\_ZONE\_BCELL\_UP |  | 20 | -0.27 | -1.21 | 0.226 | 0.483 | 1.000 | 347 | tags=40%, list=25%, signal=52% |
| 1096 | GSE10239\_MEMORY\_VS\_KLRG1HIGH\_EFF\_CD8\_TCELL\_DN |  | 22 | -0.25 | -1.21 | 0.226 | 0.483 | 1.000 | 794 | tags=86%, list=56%, signal=194% |
| 1097 | GSE2770\_UNTREATED\_VS\_ACT\_CD4\_TCELL\_48H\_DN |  | 20 | -0.27 | -1.21 | 0.224 | 0.483 | 1.000 | 157 | tags=30%, list=11%, signal=33% |
| 1098 | GSE36888\_STAT5\_AB\_KNOCKIN\_VS\_WT\_TCELL\_IL2\_TREATED\_17H\_UP |  | 26 | -0.24 | -1.21 | 0.198 | 0.483 | 1.000 | 327 | tags=38%, list=23%, signal=49% |
| 1099 | GSE24210\_IL35\_TREATED\_VS\_UNTREATED\_TCONV\_CD4\_TCELL\_DN |  | 18 | -0.28 | -1.21 | 0.196 | 0.482 | 1.000 | 222 | tags=39%, list=16%, signal=46% |
| 1100 | GSE34515\_CD16\_NEG\_MONOCYTE\_VS\_DC\_UP |  | 26 | -0.24 | -1.21 | 0.231 | 0.485 | 1.000 | 543 | tags=65%, list=38%, signal=104% |
| 1101 | GSE40273\_XBP1\_KO\_VS\_WT\_TREG\_UP |  | 22 | -0.25 | -1.21 | 0.237 | 0.484 | 1.000 | 164 | tags=23%, list=12%, signal=25% |
| 1102 | GSE38304\_MYC\_NEG\_VS\_POS\_GC\_BCELL\_DN |  | 19 | -0.27 | -1.21 | 0.224 | 0.485 | 1.000 | 551 | tags=68%, list=39%, signal=111% |
| 1103 | GSE26290\_CTRL\_VS\_AKT\_INHIBITOR\_TREATED\_ANTI\_CD3\_AND\_IL2\_STIM\_CD8\_TCELL\_UP |  | 21 | -0.26 | -1.21 | 0.202 | 0.484 | 1.000 | 373 | tags=52%, list=26%, signal=70% |
| 1104 | GSE37605\_FOXP3\_FUSION\_GFP\_VS\_IRES\_GFP\_TREG\_NOD\_DN |  | 20 | -0.27 | -1.21 | 0.212 | 0.484 | 1.000 | 161 | tags=25%, list=11%, signal=28% |
| 1105 | GSE9316\_IL6\_KO\_VS\_IFNG\_KO\_INVIVO\_EXPANDED\_CD4\_TCELL\_DN |  | 27 | -0.24 | -1.21 | 0.237 | 0.486 | 1.000 | 587 | tags=70%, list=42%, signal=118% |
| 1106 | GSE24210\_CTRL\_VS\_IL35\_TREATED\_TCONV\_CD4\_TCELL\_UP |  | 15 | -0.30 | -1.21 | 0.230 | 0.486 | 1.000 | 320 | tags=40%, list=23%, signal=51% |
| 1107 | GSE14769\_20MIN\_VS\_360MIN\_LPS\_BMDM\_DN |  | 17 | -0.29 | -1.21 | 0.248 | 0.488 | 1.000 | 1014 | tags=100%, list=72%, signal=351% |
| 1108 | GSE557\_CIITA\_KO\_VS\_I\_AB\_KO\_DC\_UP |  | 15 | -0.30 | -1.21 | 0.232 | 0.490 | 1.000 | 455 | tags=60%, list=32%, signal=88% |
| 1109 | GSE17721\_CPG\_VS\_GARDIQUIMOD\_6H\_BMDC\_UP |  | 17 | -0.28 | -1.21 | 0.233 | 0.492 | 1.000 | 399 | tags=53%, list=28%, signal=73% |
| 1110 | GSE4590\_SMALL\_VS\_VPREB\_POS\_LARGE\_PRE\_BCELL\_UP |  | 29 | -0.24 | -1.20 | 0.234 | 0.493 | 1.000 | 168 | tags=24%, list=12%, signal=27% |
| 1111 | GSE339\_CD4POS\_VS\_CD8POS\_DC\_IN\_CULTURE\_UP |  | 21 | -0.26 | -1.20 | 0.236 | 0.493 | 1.000 | 956 | tags=95%, list=68%, signal=291% |
| 1112 | GSE6259\_33D1\_POS\_VS\_DEC205\_POS\_FLT3L\_INDUCED\_SPLENIC\_DC\_DN |  | 21 | -0.26 | -1.20 | 0.251 | 0.495 | 1.000 | 386 | tags=48%, list=27%, signal=65% |
| 1113 | GSE24142\_DN2\_VS\_DN3\_THYMOCYTE\_ADULT\_DN |  | 18 | -0.27 | -1.20 | 0.233 | 0.495 | 1.000 | 284 | tags=44%, list=20%, signal=55% |
| 1114 | GSE26727\_WT\_VS\_KLF2\_KO\_LPS\_STIM\_MACROPHAGE\_DN |  | 17 | -0.28 | -1.20 | 0.247 | 0.496 | 1.000 | 702 | tags=82%, list=50%, signal=162% |
| 1115 | GSE3982\_MAST\_CELL\_VS\_TH1\_DN |  | 15 | -0.31 | -1.20 | 0.235 | 0.496 | 1.000 | 737 | tags=87%, list=52%, signal=180% |
| 1116 | GSE14308\_TH2\_VS\_TH1\_UP |  | 24 | -0.24 | -1.20 | 0.232 | 0.498 | 1.000 | 247 | tags=29%, list=18%, signal=35% |
| 1117 | GSE22611\_UNSTIM\_VS\_2H\_MDP\_STIM\_NOD2\_TRANSDUCED\_HEK293T\_CELL\_UP |  | 17 | -0.28 | -1.20 | 0.238 | 0.498 | 1.000 | 195 | tags=29%, list=14%, signal=34% |
| 1118 | GSE13522\_CTRL\_VS\_T\_CRUZI\_Y\_STRAIN\_INF\_SKIN\_IFNAR\_KO\_DN |  | 18 | -0.27 | -1.20 | 0.227 | 0.499 | 1.000 | 499 | tags=67%, list=35%, signal=102% |
| 1119 | GSE17721\_CTRL\_VS\_LPS\_2H\_BMDC\_DN |  | 21 | -0.26 | -1.20 | 0.235 | 0.498 | 1.000 | 513 | tags=67%, list=36%, signal=103% |
| 1120 | GSE42021\_CD24HI\_VS\_CD24INT\_TREG\_THYMUS\_UP |  | 19 | -0.28 | -1.20 | 0.210 | 0.498 | 1.000 | 319 | tags=42%, list=23%, signal=54% |
| 1121 | GSE17721\_LPS\_VS\_PAM3CSK4\_1H\_BMDC\_DN |  | 27 | -0.23 | -1.20 | 0.228 | 0.498 | 1.000 | 266 | tags=33%, list=19%, signal=40% |
| 1122 | GSE23321\_CD8\_STEM\_CELL\_MEMORY\_VS\_EFFECTOR\_MEMORY\_CD8\_TCELL\_DN |  | 23 | -0.25 | -1.20 | 0.254 | 0.498 | 1.000 | 150 | tags=26%, list=11%, signal=29% |
| 1123 | GSE39556\_UNTREATED\_VS\_3H\_POLYIC\_INJ\_MOUSE\_CD8A\_DC\_DN |  | 22 | -0.25 | -1.20 | 0.236 | 0.499 | 1.000 | 346 | tags=41%, list=25%, signal=53% |
| 1124 | GSE4535\_BM\_DERIVED\_DC\_VS\_FOLLICULAR\_DC\_DN |  | 25 | -0.24 | -1.20 | 0.238 | 0.499 | 1.000 | 211 | tags=28%, list=15%, signal=32% |
| 1125 | GSE15330\_HSC\_VS\_GRANULOCYTE\_MONOCYTE\_PROGENITOR\_DN |  | 19 | -0.27 | -1.20 | 0.238 | 0.499 | 1.000 | 32 | tags=16%, list=2%, signal=16% |
| 1126 | GSE26156\_DOUBLE\_POSITIVE\_VS\_CD4\_SINGLE\_POSITIVE\_THYMOCYTE\_DN |  | 20 | -0.26 | -1.20 | 0.248 | 0.499 | 1.000 | 760 | tags=85%, list=54%, signal=182% |
| 1127 | GSE15330\_HSC\_VS\_LYMPHOID\_PRIMED\_MULTIPOTENT\_PROGENITOR\_DN |  | 16 | -0.28 | -1.20 | 0.236 | 0.499 | 1.000 | 542 | tags=69%, list=38%, signal=110% |
| 1128 | GSE21774\_CD56\_BRIGHT\_VS\_DIM\_CD62L\_POSITIVE\_NK\_CELL\_DN |  | 22 | -0.25 | -1.20 | 0.235 | 0.499 | 1.000 | 153 | tags=23%, list=11%, signal=25% |
| 1129 | GSE339\_EX\_VIVO\_VS\_IN\_CULTURE\_CD4CD8DN\_DC\_DN |  | 16 | -0.28 | -1.19 | 0.225 | 0.501 | 1.000 | 537 | tags=69%, list=38%, signal=110% |
| 1130 | GSE10325\_BCELL\_VS\_MYELOID\_UP |  | 18 | -0.27 | -1.19 | 0.273 | 0.501 | 1.000 | 334 | tags=50%, list=24%, signal=65% |
| 1131 | GSE19198\_CTRL\_VS\_IL21\_TREATED\_TCELL\_6H\_UP |  | 21 | -0.26 | -1.19 | 0.248 | 0.501 | 1.000 | 393 | tags=48%, list=28%, signal=65% |
| 1132 | GSE41867\_NAIVE\_VS\_DAY6\_LCMV\_ARMSTRONG\_EFFECTOR\_CD8\_TCELL\_DN |  | 17 | -0.28 | -1.19 | 0.242 | 0.501 | 1.000 | 266 | tags=35%, list=19%, signal=43% |
| 1133 | GSE17721\_POLYIC\_VS\_GARDIQUIMOD\_6H\_BMDC\_UP |  | 17 | -0.28 | -1.19 | 0.229 | 0.502 | 1.000 | 217 | tags=35%, list=15%, signal=41% |
| 1134 | GSE16451\_CTRL\_VS\_WEST\_EQUINE\_ENC\_VIRUS\_MATURE\_NEURON\_CELL\_LINE\_UP |  | 21 | -0.25 | -1.19 | 0.233 | 0.503 | 1.000 | 466 | tags=57%, list=33%, signal=84% |
| 1135 | GSE24634\_IL4\_VS\_CTRL\_TREATED\_NAIVE\_CD4\_TCELL\_DAY5\_UP |  | 18 | -0.27 | -1.19 | 0.230 | 0.504 | 1.000 | 469 | tags=61%, list=33%, signal=90% |
| 1136 | GSE11864\_CSF1\_VS\_CSF1\_IFNG\_IN\_MAC\_UP |  | 17 | -0.28 | -1.19 | 0.279 | 0.504 | 1.000 | 218 | tags=35%, list=15%, signal=41% |
| 1137 | GSE22886\_NAIVE\_CD8\_TCELL\_VS\_NEUTROPHIL\_DN |  | 16 | -0.28 | -1.19 | 0.232 | 0.505 | 1.000 | 562 | tags=69%, list=40%, signal=113% |
| 1138 | GSE27670\_BLIMP1\_VS\_LMP1\_TRANSDUCED\_GC\_BCELL\_DN |  | 16 | -0.28 | -1.19 | 0.253 | 0.506 | 1.000 | 543 | tags=75%, list=38%, signal=121% |
| 1139 | GSE10239\_NAIVE\_VS\_KLRG1INT\_EFF\_CD8\_TCELL\_DN |  | 19 | -0.27 | -1.19 | 0.243 | 0.506 | 1.000 | 353 | tags=47%, list=25%, signal=62% |
| 1140 | GSE9650\_NAIVE\_VS\_EXHAUSTED\_CD8\_TCELL\_DN |  | 16 | -0.29 | -1.19 | 0.253 | 0.507 | 1.000 | 266 | tags=38%, list=19%, signal=46% |
| 1141 | GSE28783\_CTRL\_ANTI\_MIR\_VS\_UNTREATED\_ATHEROSCLEROSIS\_MACROPHAGE\_DN |  | 16 | -0.28 | -1.19 | 0.243 | 0.506 | 1.000 | 508 | tags=63%, list=36%, signal=97% |
| 1142 | GSE360\_L\_DONOVANI\_VS\_M\_TUBERCULOSIS\_MAC\_UP |  | 29 | -0.23 | -1.19 | 0.249 | 0.506 | 1.000 | 591 | tags=66%, list=42%, signal=110% |
| 1143 | GSE10325\_LUPUS\_CD4\_TCELL\_VS\_LUPUS\_MYELOID\_UP |  | 15 | -0.29 | -1.19 | 0.228 | 0.506 | 1.000 | 378 | tags=47%, list=27%, signal=63% |
| 1144 | GSE25123\_CTRL\_VS\_IL4\_STIM\_MACROPHAGE\_DN |  | 19 | -0.27 | -1.19 | 0.224 | 0.506 | 1.000 | 250 | tags=37%, list=18%, signal=44% |
| 1145 | GSE19923\_WT\_VS\_HEB\_AND\_E2A\_KO\_DP\_THYMOCYTE\_UP |  | 19 | -0.27 | -1.19 | 0.230 | 0.507 | 1.000 | 846 | tags=89%, list=60%, signal=220% |
| 1146 | GSE19941\_IL10\_KO\_VS\_IL10\_KO\_AND\_NFKBP50\_KO\_LPS\_AND\_IL10\_STIM\_MACROPHAGE\_DN |  | 18 | -0.27 | -1.19 | 0.231 | 0.506 | 1.000 | 290 | tags=44%, list=21%, signal=55% |
| 1147 | GSE19401\_UNSTIM\_VS\_RETINOIC\_ACID\_AND\_PAM2CSK4\_STIM\_FOLLICULAR\_DC\_DN |  | 21 | -0.25 | -1.19 | 0.247 | 0.507 | 1.000 | 219 | tags=29%, list=16%, signal=33% |
| 1148 | GSE29618\_MONOCYTE\_VS\_PDC\_DN |  | 24 | -0.24 | -1.19 | 0.252 | 0.507 | 1.000 | 575 | tags=67%, list=41%, signal=111% |
| 1149 | GSE14026\_TH1\_VS\_TH17\_DN |  | 18 | -0.27 | -1.19 | 0.241 | 0.507 | 1.000 | 338 | tags=44%, list=24%, signal=58% |
| 1150 | GSE360\_DC\_VS\_MAC\_M\_TUBERCULOSIS\_UP |  | 20 | -0.26 | -1.19 | 0.233 | 0.507 | 1.000 | 926 | tags=95%, list=66%, signal=272% |
| 1151 | GSE17721\_POLYIC\_VS\_GARDIQUIMOD\_8H\_BMDC\_UP |  | 23 | -0.25 | -1.19 | 0.243 | 0.507 | 1.000 | 255 | tags=35%, list=18%, signal=42% |
| 1152 | GSE32986\_GMCSF\_VS\_GMCSF\_AND\_CURDLAN\_LOWDOSE\_STIM\_DC\_UP |  | 22 | -0.25 | -1.19 | 0.251 | 0.509 | 1.000 | 588 | tags=68%, list=42%, signal=115% |
| 1153 | GSE3982\_CTRL\_VS\_PMA\_STIM\_EOSINOPHIL\_DN |  | 22 | -0.24 | -1.18 | 0.232 | 0.510 | 1.000 | 346 | tags=45%, list=25%, signal=59% |
| 1154 | GSE40277\_EOS\_AND\_LEF1\_TRANSDUCED\_VS\_CTRL\_CD4\_TCELL\_DN |  | 20 | -0.27 | -1.18 | 0.207 | 0.511 | 1.000 | 939 | tags=95%, list=67%, signal=280% |
| 1155 | GSE3337\_CTRL\_VS\_16H\_IFNG\_IN\_CD8POS\_DC\_UP |  | 25 | -0.23 | -1.18 | 0.251 | 0.513 | 1.000 | 383 | tags=48%, list=27%, signal=65% |
| 1156 | GSE40274\_CTRL\_VS\_HELIOS\_TRANSDUCED\_ACTIVATED\_CD4\_TCELL\_DN |  | 23 | -0.25 | -1.18 | 0.240 | 0.515 | 1.000 | 716 | tags=83%, list=51%, signal=165% |
| 1157 | GSE3982\_MAST\_CELL\_VS\_MAC\_UP |  | 21 | -0.26 | -1.18 | 0.255 | 0.517 | 1.000 | 492 | tags=62%, list=35%, signal=94% |
| 1158 | GSE39022\_LN\_VS\_SPLEEN\_DC\_UP |  | 19 | -0.26 | -1.18 | 0.262 | 0.517 | 1.000 | 415 | tags=53%, list=29%, signal=74% |
| 1159 | GSE3982\_BCELL\_VS\_NKCELL\_UP |  | 21 | -0.26 | -1.18 | 0.247 | 0.516 | 1.000 | 331 | tags=43%, list=23%, signal=55% |
| 1160 | GSE5589\_UNSTIM\_VS\_45MIN\_LPS\_AND\_IL10\_STIM\_MACROPHAGE\_UP |  | 19 | -0.25 | -1.18 | 0.233 | 0.516 | 1.000 | 6 | tags=11%, list=0%, signal=10% |
| 1161 | GSE17721\_4\_VS\_24H\_GARDIQUIMOD\_BMDC\_UP |  | 20 | -0.26 | -1.18 | 0.257 | 0.516 | 1.000 | 183 | tags=25%, list=13%, signal=28% |
| 1162 | GSE3982\_DC\_VS\_CENT\_MEMORY\_CD4\_TCELL\_UP |  | 18 | -0.27 | -1.18 | 0.265 | 0.518 | 1.000 | 605 | tags=72%, list=43%, signal=125% |
| 1163 | GSE32034\_UNTREATED\_VS\_ROSIGLIZATONE\_TREATED\_LY6C\_HIGH\_MONOCYTE\_UP |  | 27 | -0.23 | -1.18 | 0.238 | 0.518 | 1.000 | 596 | tags=67%, list=42%, signal=113% |
| 1164 | GSE7460\_CTRL\_VS\_FOXP3\_OVEREXPR\_TCONV\_1\_DN |  | 20 | -0.26 | -1.18 | 0.246 | 0.519 | 1.000 | 541 | tags=70%, list=38%, signal=112% |
| 1165 | GSE2706\_UNSTIM\_VS\_8H\_R848\_DC\_DN |  | 22 | -0.25 | -1.18 | 0.272 | 0.519 | 1.000 | 682 | tags=77%, list=48%, signal=147% |
| 1166 | GSE10325\_CD4\_TCELL\_VS\_MYELOID\_DN |  | 17 | -0.27 | -1.18 | 0.254 | 0.518 | 1.000 | 276 | tags=35%, list=20%, signal=43% |
| 1167 | GSE46606\_UNSTIM\_VS\_CD40L\_IL2\_IL5\_1DAY\_STIMULATED\_IRF4HIGH\_SORTED\_BCELL\_DN |  | 26 | -0.23 | -1.18 | 0.241 | 0.518 | 1.000 | 253 | tags=31%, list=18%, signal=37% |
| 1168 | GSE30962\_ACUTE\_VS\_CHRONIC\_LCMV\_SECONDARY\_INF\_CD8\_TCELL\_DN |  | 21 | -0.25 | -1.18 | 0.254 | 0.518 | 1.000 | 730 | tags=81%, list=52%, signal=165% |
| 1169 | GSE17721\_LPS\_VS\_POLYIC\_16H\_BMDC\_DN |  | 18 | -0.27 | -1.18 | 0.249 | 0.518 | 1.000 | 492 | tags=61%, list=35%, signal=93% |
| 1170 | GSE43955\_TH0\_VS\_TGFB\_IL6\_TH17\_ACT\_CD4\_TCELL\_42H\_DN |  | 22 | -0.25 | -1.17 | 0.249 | 0.519 | 1.000 | 249 | tags=32%, list=18%, signal=38% |
| 1171 | GSE13411\_NAIVE\_VS\_IGM\_MEMORY\_BCELL\_UP |  | 22 | -0.25 | -1.17 | 0.245 | 0.521 | 1.000 | 348 | tags=50%, list=25%, signal=65% |
| 1172 | GSE3920\_IFNA\_VS\_IFNB\_TREATED\_ENDOTHELIAL\_CELL\_UP |  | 28 | -0.22 | -1.17 | 0.244 | 0.521 | 1.000 | 384 | tags=46%, list=27%, signal=63% |
| 1173 | GSE6259\_33D1\_POS\_VS\_DEC205\_POS\_SPLENIC\_DC\_UP |  | 33 | -0.22 | -1.17 | 0.259 | 0.524 | 1.000 | 531 | tags=61%, list=38%, signal=95% |
| 1174 | GSE8921\_UNSTIM\_0H\_VS\_TLR1\_2\_STIM\_MONOCYTE\_6H\_DN |  | 20 | -0.26 | -1.17 | 0.264 | 0.525 | 1.000 | 469 | tags=55%, list=33%, signal=81% |
| 1175 | GSE6674\_ANTI\_IGM\_VS\_PL2\_3\_STIM\_BCELL\_UP |  | 20 | -0.26 | -1.17 | 0.248 | 0.524 | 1.000 | 382 | tags=50%, list=27%, signal=68% |
| 1176 | GSE27786\_CD8\_TCELL\_VS\_NKTCELL\_DN |  | 15 | -0.29 | -1.17 | 0.251 | 0.524 | 1.000 | 360 | tags=47%, list=26%, signal=62% |
| 1177 | GSE15930\_NAIVE\_VS\_48H\_IN\_VITRO\_STIM\_IL12\_CD8\_TCELL\_DN |  | 23 | -0.24 | -1.17 | 0.256 | 0.524 | 1.000 | 1078 | tags=100%, list=76%, signal=417% |
| 1178 | GSE27859\_MACROPHAGE\_VS\_DC\_UP |  | 18 | -0.27 | -1.17 | 0.255 | 0.524 | 1.000 | 363 | tags=50%, list=26%, signal=66% |
| 1179 | GSE46606\_UNSTIM\_VS\_CD40L\_IL2\_IL5\_3DAY\_STIMULATED\_IRF4MID\_SORTED\_BCELL\_DN |  | 19 | -0.26 | -1.17 | 0.239 | 0.524 | 1.000 | 393 | tags=53%, list=28%, signal=72% |
| 1180 | GSE17721\_LPS\_VS\_PAM3CSK4\_24H\_BMDC\_UP |  | 17 | -0.27 | -1.17 | 0.288 | 0.525 | 1.000 | 924 | tags=94%, list=65%, signal=269% |
| 1181 | GSE25088\_IL4\_VS\_IL4\_AND\_ROSIGLITAZONE\_STIM\_STAT6\_KO\_MACROPHAGE\_DAY10\_DN |  | 18 | -0.26 | -1.17 | 0.234 | 0.525 | 1.000 | 266 | tags=33%, list=19%, signal=41% |
| 1182 | GSE33292\_WT\_VS\_TCF1\_KO\_DN3\_THYMOCYTE\_UP |  | 30 | -0.22 | -1.17 | 0.272 | 0.526 | 1.000 | 368 | tags=43%, list=26%, signal=57% |
| 1183 | GSE14769\_40MIN\_VS\_360MIN\_LPS\_BMDM\_DN |  | 16 | -0.28 | -1.17 | 0.298 | 0.526 | 1.000 | 266 | tags=38%, list=19%, signal=46% |
| 1184 | GSE22935\_WT\_VS\_MYD88\_KO\_MACROPHAGE\_24H\_MBOVIS\_BCG\_STIM\_DN |  | 23 | -0.25 | -1.17 | 0.246 | 0.528 | 1.000 | 679 | tags=78%, list=48%, signal=148% |
| 1185 | GSE27786\_CD8\_TCELL\_VS\_NEUTROPHIL\_DN |  | 20 | -0.25 | -1.17 | 0.264 | 0.529 | 1.000 | 362 | tags=45%, list=26%, signal=60% |
| 1186 | GSE20152\_SPHK1\_KO\_VS\_HTNFA\_OVEREXPRESS\_ANKLE\_UP |  | 23 | -0.24 | -1.16 | 0.288 | 0.531 | 1.000 | 119 | tags=22%, list=8%, signal=23% |
| 1187 | GSE22601\_DOUBLE\_NEGATIVE\_VS\_DOUBLE\_POSITIVE\_THYMOCYTE\_DN |  | 23 | -0.24 | -1.16 | 0.253 | 0.530 | 1.000 | 310 | tags=39%, list=22%, signal=49% |
| 1188 | GSE36888\_UNTREATED\_VS\_IL2\_TREATED\_TCELL\_2H\_DN |  | 18 | -0.27 | -1.16 | 0.287 | 0.530 | 1.000 | 306 | tags=33%, list=22%, signal=42% |
| 1189 | GSE28726\_NAIVE\_VS\_ACTIVATED\_NKTCELL\_DN |  | 27 | -0.23 | -1.16 | 0.262 | 0.532 | 1.000 | 731 | tags=78%, list=52%, signal=158% |
| 1190 | GSE40274\_GATA1\_VS\_FOXP3\_AND\_GATA1\_TRANSDUCED\_ACTIVATED\_CD4\_TCELL\_DN |  | 26 | -0.22 | -1.16 | 0.266 | 0.533 | 1.000 | 508 | tags=54%, list=36%, signal=83% |
| 1191 | GSE3039\_NKT\_CELL\_VS\_ALPHAALPHA\_CD8\_TCELL\_UP |  | 38 | -0.20 | -1.16 | 0.301 | 0.533 | 1.000 | 478 | tags=55%, list=34%, signal=81% |
| 1192 | GSE22229\_RENAL\_TRANSPLANT\_VS\_HEALTHY\_PBMC\_DN |  | 24 | -0.24 | -1.16 | 0.270 | 0.533 | 1.000 | 382 | tags=46%, list=27%, signal=62% |
| 1193 | GSE17186\_CD21LOW\_VS\_CD21HIGH\_TRANSITIONAL\_BCELL\_CORD\_BLOOD\_UP |  | 17 | -0.28 | -1.16 | 0.259 | 0.533 | 1.000 | 396 | tags=47%, list=28%, signal=65% |
| 1194 | GSE9006\_HEALTHY\_VS\_TYPE\_1\_DIABETES\_PBMC\_AT\_DX\_UP |  | 17 | -0.27 | -1.16 | 0.278 | 0.533 | 1.000 | 356 | tags=47%, list=25%, signal=62% |
| 1195 | GSE21033\_3H\_VS\_12H\_POLYIC\_STIM\_DC\_UP |  | 15 | -0.28 | -1.16 | 0.265 | 0.532 | 1.000 | 1015 | tags=100%, list=72%, signal=353% |
| 1196 | GSE18281\_PERIMEDULLARY\_CORTICAL\_REGION\_VS\_WHOLE\_CORTEX\_THYMUS\_DN |  | 15 | -0.28 | -1.16 | 0.269 | 0.533 | 1.000 | 387 | tags=53%, list=27%, signal=73% |
| 1197 | GSE21670\_UNTREATED\_VS\_TGFB\_IL6\_TREATED\_STAT3\_KO\_CD4\_TCELL\_UP |  | 30 | -0.22 | -1.16 | 0.292 | 0.533 | 1.000 | 524 | tags=63%, list=37%, signal=99% |
| 1198 | GSE17974\_0H\_VS\_72H\_IN\_VITRO\_ACT\_CD4\_TCELL\_UP |  | 29 | -0.22 | -1.16 | 0.247 | 0.532 | 1.000 | 502 | tags=59%, list=36%, signal=89% |
| 1199 | GSE40274\_FOXP3\_VS\_FOXP3\_AND\_IRF4\_TRANSDUCED\_ACTIVATED\_CD4\_TCELL\_DN |  | 21 | -0.25 | -1.16 | 0.278 | 0.533 | 1.000 | 482 | tags=57%, list=34%, signal=85% |
| 1200 | GSE41176\_UNSTIM\_VS\_ANTI\_IGM\_STIM\_BCELL\_3H\_DN |  | 23 | -0.24 | -1.16 | 0.283 | 0.533 | 1.000 | 804 | tags=83%, list=57%, signal=189% |
| 1201 | GSE2197\_IMMUNOSUPPRESSIVE\_DNA\_VS\_UNTREATED\_IN\_DC\_UP |  | 18 | -0.27 | -1.16 | 0.274 | 0.532 | 1.000 | 857 | tags=89%, list=61%, signal=224% |
| 1202 | GSE9650\_EFFECTOR\_VS\_EXHAUSTED\_CD8\_TCELL\_UP |  | 15 | -0.29 | -1.16 | 0.280 | 0.533 | 1.000 | 1009 | tags=100%, list=72%, signal=347% |
| 1203 | GSE7219\_WT\_VS\_NIK\_NFKB2\_KO\_DC\_DN |  | 18 | -0.26 | -1.16 | 0.277 | 0.533 | 1.000 | 514 | tags=61%, list=36%, signal=95% |
| 1204 | GSE16522\_MEMORY\_VS\_NAIVE\_CD8\_TCELL\_UP |  | 16 | -0.27 | -1.16 | 0.265 | 0.533 | 1.000 | 279 | tags=44%, list=20%, signal=54% |
| 1205 | GSE45365\_CD8A\_DC\_VS\_CD11B\_DC\_IFNAR\_KO\_MCMV\_INFECTION\_DN |  | 15 | -0.28 | -1.16 | 0.260 | 0.532 | 1.000 | 585 | tags=73%, list=41%, signal=124% |
| 1206 | GSE43955\_1H\_VS\_60H\_ACT\_CD4\_TCELL\_UP |  | 17 | -0.27 | -1.16 | 0.285 | 0.536 | 1.000 | 482 | tags=59%, list=34%, signal=88% |
| 1207 | GSE360\_CTRL\_VS\_M\_TUBERCULOSIS\_MAC\_DN |  | 17 | -0.27 | -1.16 | 0.278 | 0.535 | 1.000 | 227 | tags=35%, list=16%, signal=42% |
| 1208 | GSE45365\_NK\_CELL\_VS\_BCELL\_MCMV\_INFECTION\_UP |  | 30 | -0.22 | -1.16 | 0.277 | 0.535 | 1.000 | 442 | tags=50%, list=31%, signal=71% |
| 1209 | GSE8384\_CTRL\_VS\_B\_ABORTUS\_4H\_MAC\_CELL\_LINE\_UP |  | 19 | -0.26 | -1.16 | 0.262 | 0.535 | 1.000 | 242 | tags=37%, list=17%, signal=44% |
| 1210 | GSE13522\_CTRL\_VS\_T\_CRUZI\_Y\_STRAIN\_INF\_SKIN\_129\_MOUSE\_DN |  | 21 | -0.25 | -1.16 | 0.280 | 0.536 | 1.000 | 973 | tags=95%, list=69%, signal=302% |
| 1211 | GSE1460\_CD4\_THYMOCYTE\_VS\_NAIVE\_CD4\_TCELL\_ADULT\_BLOOD\_UP |  | 16 | -0.28 | -1.16 | 0.289 | 0.536 | 1.000 | 414 | tags=56%, list=29%, signal=79% |
| 1212 | GSE3982\_DC\_VS\_EFF\_MEMORY\_CD4\_TCELL\_DN |  | 15 | -0.28 | -1.16 | 0.277 | 0.536 | 1.000 | 781 | tags=87%, list=55%, signal=192% |
| 1213 | GSE8921\_3H\_VS\_24H\_TLR1\_2\_STIM\_MONOCYTE\_DN |  | 29 | -0.22 | -1.16 | 0.286 | 0.536 | 1.000 | 522 | tags=55%, list=37%, signal=86% |
| 1214 | GSE43955\_TGFB\_IL6\_VS\_TGFB\_IL6\_IL23\_TH17\_ACT\_CD4\_TCELL\_60H\_UP |  | 15 | -0.28 | -1.15 | 0.253 | 0.538 | 1.000 | 443 | tags=60%, list=31%, signal=87% |
| 1215 | GSE15930\_NAIVE\_VS\_72H\_IN\_VITRO\_STIM\_IL12\_CD8\_TCELL\_UP |  | 16 | -0.28 | -1.15 | 0.268 | 0.539 | 1.000 | 440 | tags=50%, list=31%, signal=72% |
| 1216 | GSE40277\_GATA1\_AND\_SATB1\_TRANSDUCED\_VS\_CTRL\_CD4\_TCELL\_UP |  | 23 | -0.24 | -1.15 | 0.262 | 0.539 | 1.000 | 253 | tags=35%, list=18%, signal=42% |
| 1217 | GSE36527\_CD62L\_HIGH\_CD69\_NEG\_VS\_CD62L\_LOW\_CD69\_POS\_TREG\_KLRG1\_NEG\_DN |  | 22 | -0.24 | -1.15 | 0.271 | 0.539 | 1.000 | 278 | tags=36%, list=20%, signal=45% |
| 1218 | GSE24574\_BCL6\_HIGH\_TFH\_VS\_NAIVE\_CD4\_TCELL\_UP |  | 21 | -0.24 | -1.15 | 0.267 | 0.539 | 1.000 | 860 | tags=90%, list=61%, signal=228% |
| 1219 | GSE3982\_BASOPHIL\_VS\_NKCELL\_UP |  | 26 | -0.23 | -1.15 | 0.272 | 0.539 | 1.000 | 557 | tags=65%, list=39%, signal=106% |
| 1220 | GSE17721\_CPG\_VS\_GARDIQUIMOD\_16H\_BMDC\_DN |  | 20 | -0.25 | -1.15 | 0.284 | 0.541 | 1.000 | 490 | tags=60%, list=35%, signal=91% |
| 1221 | GSE45365\_NK\_CELL\_VS\_CD11B\_DC\_DN |  | 52 | -0.18 | -1.15 | 0.247 | 0.541 | 1.000 | 618 | tags=67%, list=44%, signal=115% |
| 1222 | GSE36826\_WT\_VS\_IL1R\_KO\_SKIN\_STAPH\_AUREUS\_INF\_DN |  | 24 | -0.23 | -1.15 | 0.277 | 0.544 | 1.000 | 372 | tags=42%, list=26%, signal=56% |
| 1223 | GSE9988\_ANTI\_TREM1\_VS\_LOW\_LPS\_MONOCYTE\_DN |  | 22 | -0.25 | -1.15 | 0.267 | 0.546 | 1.000 | 547 | tags=68%, list=39%, signal=110% |
| 1224 | GSE360\_L\_MAJOR\_VS\_M\_TUBERCULOSIS\_MAC\_DN |  | 15 | -0.28 | -1.15 | 0.314 | 0.545 | 1.000 | 344 | tags=40%, list=24%, signal=52% |
| 1225 | GSE7348\_UNSTIM\_VS\_TOLERIZED\_AND\_LPS\_STIM\_MACROPHAGE\_DN |  | 18 | -0.25 | -1.15 | 0.287 | 0.547 | 1.000 | 357 | tags=50%, list=25%, signal=66% |
| 1226 | GSE39152\_CD103\_NEG\_VS\_POS\_MEMORY\_CD8\_TCELL\_DN |  | 25 | -0.23 | -1.15 | 0.282 | 0.548 | 1.000 | 603 | tags=72%, list=43%, signal=124% |
| 1227 | GSE22589\_SIV\_VS\_HIV\_AND\_SIV\_INFECTED\_DC\_UP |  | 17 | -0.27 | -1.15 | 0.270 | 0.547 | 1.000 | 259 | tags=41%, list=18%, signal=50% |
| 1228 | GSE37534\_GW1929\_VS\_PIOGLITAZONE\_TREATED\_CD4\_TCELL\_PPARG1\_FOXP3\_TRANSDUCED\_DN |  | 22 | -0.24 | -1.15 | 0.303 | 0.547 | 1.000 | 182 | tags=23%, list=13%, signal=26% |
| 1229 | GSE3203\_INFLUENZA\_INF\_VS\_IFNB\_TREATED\_LN\_BCELL\_DN |  | 27 | -0.22 | -1.15 | 0.283 | 0.547 | 1.000 | 295 | tags=37%, list=21%, signal=46% |
| 1230 | GSE26928\_EFF\_MEMORY\_VS\_CXCR5\_POS\_CD4\_TCELL\_DN |  | 24 | -0.23 | -1.15 | 0.265 | 0.547 | 1.000 | 163 | tags=29%, list=12%, signal=32% |
| 1231 | GSE20500\_CTRL\_VS\_RARA\_ANTAGONIST\_TREATED\_CD4\_TCELL\_UP |  | 17 | -0.27 | -1.14 | 0.302 | 0.548 | 1.000 | 236 | tags=35%, list=17%, signal=42% |
| 1232 | GSE12392\_WT\_VS\_IFNAR\_KO\_CD8A\_NEG\_SPLEEN\_DC\_UP |  | 20 | -0.25 | -1.14 | 0.308 | 0.547 | 1.000 | 573 | tags=70%, list=41%, signal=116% |
| 1233 | GSE11924\_TH1\_VS\_TH2\_CD4\_TCELL\_DN |  | 17 | -0.26 | -1.14 | 0.265 | 0.547 | 1.000 | 18 | tags=12%, list=1%, signal=12% |
| 1234 | GSE25087\_TREG\_VS\_TCONV\_FETUS\_UP |  | 19 | -0.26 | -1.14 | 0.299 | 0.547 | 1.000 | 572 | tags=68%, list=41%, signal=114% |
| 1235 | GSE360\_CTRL\_VS\_T\_GONDII\_MAC\_DN |  | 24 | -0.23 | -1.14 | 0.280 | 0.547 | 1.000 | 480 | tags=54%, list=34%, signal=81% |
| 1236 | GSE13547\_2H\_VS\_12\_H\_ANTI\_IGM\_STIM\_BCELL\_UP |  | 20 | -0.25 | -1.14 | 0.254 | 0.547 | 1.000 | 685 | tags=80%, list=49%, signal=153% |
| 1237 | GSE9960\_HEALTHY\_VS\_GRAM\_NEG\_SEPSIS\_PBMC\_DN |  | 21 | -0.24 | -1.14 | 0.295 | 0.547 | 1.000 | 572 | tags=67%, list=41%, signal=110% |
| 1238 | GSE14769\_UNSTIM\_VS\_40MIN\_LPS\_BMDM\_DN |  | 32 | -0.21 | -1.14 | 0.305 | 0.547 | 1.000 | 563 | tags=66%, list=40%, signal=107% |
| 1239 | GSE33292\_WT\_VS\_TCF1\_KO\_DN3\_THYMOCYTE\_DN |  | 18 | -0.26 | -1.14 | 0.298 | 0.547 | 1.000 | 114 | tags=22%, list=8%, signal=24% |
| 1240 | GSE15624\_CTRL\_VS\_3H\_HALOFUGINONE\_TREATED\_CD4\_TCELL\_DN |  | 17 | -0.26 | -1.14 | 0.263 | 0.548 | 1.000 | 914 | tags=94%, list=65%, signal=264% |
| 1241 | GSE22313\_HEALTHY\_VS\_SLE\_MOUSE\_CD4\_TCELL\_UP |  | 16 | -0.27 | -1.14 | 0.270 | 0.553 | 1.000 | 608 | tags=75%, list=43%, signal=130% |
| 1242 | GSE36476\_YOUNG\_VS\_OLD\_DONOR\_MEMORY\_CD4\_TCELL\_16H\_TSST\_ACT\_DN |  | 22 | -0.24 | -1.14 | 0.266 | 0.553 | 1.000 | 450 | tags=55%, list=32%, signal=79% |
| 1243 | GSE25123\_CTRL\_VS\_IL4\_STIM\_PPARG\_KO\_MACROPHAGE\_UP |  | 15 | -0.28 | -1.14 | 0.284 | 0.555 | 1.000 | 202 | tags=27%, list=14%, signal=31% |
| 1244 | GSE33162\_UNTREATED\_VS\_4H\_LPS\_STIM\_HDAC3\_HET\_MACROPHAGE\_UP |  | 21 | -0.25 | -1.14 | 0.291 | 0.556 | 1.000 | 182 | tags=29%, list=13%, signal=32% |
| 1245 | GSE1740\_UNSTIM\_VS\_IFNA\_STIMULATED\_MCSF\_IFNG\_DERIVED\_MACROPHAGE\_UP |  | 15 | -0.27 | -1.14 | 0.290 | 0.556 | 1.000 | 403 | tags=53%, list=29%, signal=74% |
| 1246 | GSE12198\_CTRL\_VS\_LOW\_IL2\_STIM\_NK\_CELL\_DN |  | 24 | -0.23 | -1.14 | 0.286 | 0.556 | 1.000 | 402 | tags=46%, list=28%, signal=63% |
| 1247 | GSE1460\_CORD\_VS\_ADULT\_BLOOD\_NAIVE\_CD4\_TCELL\_UP |  | 22 | -0.24 | -1.14 | 0.286 | 0.556 | 1.000 | 334 | tags=41%, list=24%, signal=53% |
| 1248 | GSE29164\_DAY3\_VS\_DAY7\_CD8\_TCELL\_TREATED\_MELANOMA\_DN |  | 16 | -0.27 | -1.14 | 0.293 | 0.558 | 1.000 | 382 | tags=44%, list=27%, signal=59% |
| 1249 | GSE29618\_PDC\_VS\_MDC\_DAY7\_FLU\_VACCINE\_UP |  | 19 | -0.25 | -1.14 | 0.283 | 0.558 | 1.000 | 299 | tags=37%, list=21%, signal=46% |
| 1250 | GSE2770\_IL12\_VS\_TGFB\_AND\_IL12\_TREATED\_ACT\_CD4\_TCELL\_2H\_DN |  | 16 | -0.28 | -1.13 | 0.302 | 0.560 | 1.000 | 301 | tags=44%, list=21%, signal=55% |
| 1251 | GSE1791\_CTRL\_VS\_NEUROMEDINU\_IN\_T\_CELL\_LINE\_3H\_UP |  | 17 | -0.27 | -1.13 | 0.303 | 0.561 | 1.000 | 512 | tags=65%, list=36%, signal=100% |
| 1252 | GSE23568\_CTRL\_TRANSDUCED\_VS\_WT\_CD8\_TCELL\_DN |  | 15 | -0.27 | -1.13 | 0.281 | 0.560 | 1.000 | 32 | tags=13%, list=2%, signal=13% |
| 1253 | GSE22589\_HEALTHY\_VS\_HIV\_INFECTED\_DC\_DN |  | 18 | -0.26 | -1.13 | 0.307 | 0.561 | 1.000 | 317 | tags=44%, list=22%, signal=57% |
| 1254 | GSE2405\_HEAT\_KILLED\_VS\_LIVE\_A\_PHAGOCYTOPHILUM\_STIM\_NEUTROPHIL\_9H\_DN |  | 21 | -0.25 | -1.13 | 0.286 | 0.562 | 1.000 | 263 | tags=38%, list=19%, signal=46% |
| 1255 | GSE45739\_UNSTIM\_VS\_ACD3\_ACD28\_STIM\_NRAS\_KO\_CD4\_TCELL\_UP |  | 20 | -0.25 | -1.13 | 0.285 | 0.563 | 1.000 | 35 | tags=15%, list=2%, signal=15% |
| 1256 | GSE23502\_BM\_VS\_COLON\_TUMOR\_MYELOID\_DERIVED\_SUPPRESSOR\_CELL\_DN |  | 22 | -0.24 | -1.13 | 0.303 | 0.564 | 1.000 | 117 | tags=27%, list=8%, signal=29% |
| 1257 | KAECH\_DAY8\_EFF\_VS\_MEMORY\_CD8\_TCELL\_UP |  | 16 | -0.26 | -1.13 | 0.283 | 0.564 | 1.000 | 59 | tags=19%, list=4%, signal=19% |
| 1258 | GSE7348\_UNSTIM\_VS\_LPS\_STIM\_MACROPHAGE\_DN |  | 22 | -0.24 | -1.13 | 0.308 | 0.564 | 1.000 | 309 | tags=41%, list=22%, signal=52% |
| 1259 | GSE17974\_IL4\_AND\_ANTI\_IL12\_VS\_UNTREATED\_4H\_ACT\_CD4\_TCELL\_UP |  | 16 | -0.27 | -1.13 | 0.296 | 0.564 | 1.000 | 169 | tags=31%, list=12%, signal=35% |
| 1260 | GSE35685\_CD34POS\_CD38NEG\_VS\_CD34POS\_CD10POS\_BONE\_MARROW\_DN |  | 36 | -0.19 | -1.13 | 0.317 | 0.564 | 1.000 | 678 | tags=72%, list=48%, signal=135% |
| 1261 | GSE26488\_CTRL\_VS\_PEPTIDE\_INJECTION\_OT2\_THYMOCYTE\_DN |  | 16 | -0.27 | -1.13 | 0.293 | 0.563 | 1.000 | 476 | tags=69%, list=34%, signal=103% |
| 1262 | GSE26669\_CTRL\_VS\_COSTIM\_BLOCK\_MLR\_CD8\_TCELL\_DN |  | 21 | -0.24 | -1.13 | 0.322 | 0.563 | 1.000 | 108 | tags=19%, list=8%, signal=20% |
| 1263 | GSE22886\_UNSTIM\_VS\_IL15\_STIM\_NKCELL\_DN |  | 22 | -0.24 | -1.13 | 0.293 | 0.564 | 1.000 | 681 | tags=73%, list=48%, signal=138% |
| 1264 | GSE13484\_UNSTIM\_VS\_YF17D\_VACCINE\_STIM\_PBMC\_DN |  | 26 | -0.22 | -1.13 | 0.295 | 0.565 | 1.000 | 370 | tags=42%, list=26%, signal=56% |
| 1265 | GSE15330\_HSC\_VS\_MEGAKARYOCYTE\_ERYTHROID\_PROGENITOR\_IKAROS\_KO\_UP |  | 18 | -0.26 | -1.13 | 0.310 | 0.565 | 1.000 | 469 | tags=56%, list=33%, signal=82% |
| 1266 | GSE23925\_DARK\_ZONE\_VS\_NAIVE\_BCELL\_UP |  | 19 | -0.26 | -1.13 | 0.311 | 0.565 | 1.000 | 171 | tags=26%, list=12%, signal=30% |
| 1267 | GSE37533\_PPARG1\_FOXP3\_VS\_FOXP3\_TRANSDUCED\_CD4\_TCELL\_UP |  | 19 | -0.25 | -1.13 | 0.287 | 0.565 | 1.000 | 259 | tags=37%, list=18%, signal=45% |
| 1268 | GSE25123\_CTRL\_VS\_IL4\_STIM\_PPARG\_KO\_MACROPHAGE\_DN |  | 15 | -0.27 | -1.13 | 0.299 | 0.569 | 1.000 | 391 | tags=53%, list=28%, signal=73% |
| 1269 | GSE7460\_CTRL\_VS\_TGFB\_TREATED\_ACT\_FOXP3\_HET\_TCONV\_DN |  | 24 | -0.23 | -1.12 | 0.290 | 0.568 | 1.000 | 584 | tags=67%, list=41%, signal=112% |
| 1270 | GSE411\_WT\_VS\_SOCS3\_KO\_MACROPHAGE\_UP |  | 20 | -0.24 | -1.12 | 0.346 | 0.568 | 1.000 | 289 | tags=35%, list=20%, signal=43% |
| 1271 | GSE7219\_UNSTIM\_VS\_LPS\_AND\_ANTI\_CD40\_STIM\_DC\_UP |  | 22 | -0.23 | -1.12 | 0.300 | 0.570 | 1.000 | 523 | tags=59%, list=37%, signal=92% |
| 1272 | GSE23502\_WT\_VS\_HDC\_KO\_MYELOID\_DERIVED\_SUPPRESSOR\_CELL\_COLON\_TUMOR\_UP |  | 18 | -0.25 | -1.12 | 0.312 | 0.570 | 1.000 | 350 | tags=50%, list=25%, signal=66% |
| 1273 | GSE5589\_UNSTIM\_VS\_45MIN\_LPS\_STIM\_MACROPHAGE\_UP |  | 18 | -0.26 | -1.12 | 0.310 | 0.571 | 1.000 | 175 | tags=28%, list=12%, signal=31% |
| 1274 | GSE2770\_TGFB\_AND\_IL4\_VS\_IL12\_TREATED\_ACT\_CD4\_TCELL\_2H\_DN |  | 27 | -0.21 | -1.12 | 0.292 | 0.571 | 1.000 | 221 | tags=30%, list=16%, signal=34% |
| 1275 | GSE19401\_UNSTIM\_VS\_PAM2CSK4\_STIM\_FOLLICULAR\_DC\_UP |  | 22 | -0.23 | -1.12 | 0.301 | 0.571 | 1.000 | 1087 | tags=100%, list=77%, signal=429% |
| 1276 | GSE17974\_IL4\_AND\_ANTI\_IL12\_VS\_UNTREATED\_72H\_ACT\_CD4\_TCELL\_DN |  | 18 | -0.26 | -1.12 | 0.304 | 0.571 | 1.000 | 373 | tags=50%, list=26%, signal=67% |
| 1277 | GSE12003\_4D\_VS\_8D\_CULTURE\_BM\_PROGENITOR\_UP |  | 23 | -0.23 | -1.12 | 0.287 | 0.571 | 1.000 | 864 | tags=87%, list=61%, signal=221% |
| 1278 | GSE37301\_MULTIPOTENT\_PROGENITOR\_VS\_RAG2\_KO\_NK\_CELL\_UP |  | 19 | -0.25 | -1.12 | 0.303 | 0.571 | 1.000 | 45 | tags=16%, list=3%, signal=16% |
| 1279 | GSE13738\_RESTING\_VS\_TCR\_ACTIVATED\_CD4\_TCELL\_UP |  | 26 | -0.22 | -1.12 | 0.317 | 0.571 | 1.000 | 184 | tags=27%, list=13%, signal=30% |
| 1280 | GSE18791\_CTRL\_VS\_NEWCASTLE\_VIRUS\_DC\_14H\_DN |  | 18 | -0.26 | -1.12 | 0.312 | 0.573 | 1.000 | 342 | tags=44%, list=24%, signal=58% |
| 1281 | GSE17721\_CTRL\_VS\_GARDIQUIMOD\_6H\_BMDC\_UP |  | 17 | -0.27 | -1.12 | 0.286 | 0.573 | 1.000 | 308 | tags=41%, list=22%, signal=52% |
| 1282 | GSE24814\_STAT5\_KO\_VS\_WT\_PRE\_BCELL\_DN |  | 24 | -0.23 | -1.12 | 0.317 | 0.573 | 1.000 | 161 | tags=25%, list=11%, signal=28% |
| 1283 | GSE28726\_NAIVE\_CD4\_TCELL\_VS\_NAIVE\_NKTCELL\_UP |  | 23 | -0.23 | -1.12 | 0.323 | 0.573 | 1.000 | 15 | tags=9%, list=1%, signal=9% |
| 1284 | GSE36826\_WT\_VS\_IL1R\_KO\_SKIN\_DN |  | 22 | -0.24 | -1.12 | 0.333 | 0.573 | 1.000 | 293 | tags=36%, list=21%, signal=45% |
| 1285 | GSE7460\_FOXP3\_MUT\_VS\_WT\_ACT\_WITH\_TGFB\_TCONV\_UP |  | 19 | -0.25 | -1.12 | 0.296 | 0.575 | 1.000 | 802 | tags=84%, list=57%, signal=192% |
| 1286 | GSE17721\_POLYIC\_VS\_GARDIQUIMOD\_24H\_BMDC\_UP |  | 17 | -0.26 | -1.12 | 0.308 | 0.575 | 1.000 | 527 | tags=65%, list=37%, signal=102% |
| 1287 | GSE3039\_B2\_VS\_B1\_BCELL\_UP |  | 17 | -0.26 | -1.12 | 0.287 | 0.575 | 1.000 | 521 | tags=71%, list=37%, signal=111% |
| 1288 | GSE8921\_UNSTIM\_VS\_TLR1\_2\_STIM\_MONOCYTE\_12H\_DN |  | 24 | -0.22 | -1.12 | 0.311 | 0.575 | 1.000 | 74 | tags=17%, list=5%, signal=17% |
| 1289 | GSE29618\_BCELL\_VS\_PDC\_DAY7\_FLU\_VACCINE\_DN |  | 19 | -0.26 | -1.12 | 0.300 | 0.575 | 1.000 | 560 | tags=68%, list=40%, signal=112% |
| 1290 | KAECH\_NAIVE\_VS\_MEMORY\_CD8\_TCELL\_UP |  | 26 | -0.22 | -1.12 | 0.317 | 0.577 | 1.000 | 90 | tags=19%, list=6%, signal=20% |
| 1291 | GSE14415\_TCONV\_VS\_FOXP3\_KO\_INDUCED\_TREG\_DN |  | 20 | -0.24 | -1.12 | 0.301 | 0.576 | 1.000 | 1081 | tags=100%, list=77%, signal=422% |
| 1292 | GSE13306\_RA\_VS\_UNTREATED\_TREG\_DN |  | 15 | -0.28 | -1.12 | 0.302 | 0.577 | 1.000 | 310 | tags=47%, list=22%, signal=59% |
| 1293 | GSE17301\_ACD3\_ACD28\_VS\_ACD3\_ACD28\_AND\_IFNA2\_STIM\_CD8\_TCELL\_DN |  | 24 | -0.23 | -1.11 | 0.316 | 0.577 | 1.000 | 346 | tags=46%, list=25%, signal=60% |
| 1294 | GSE22103\_UNSTIM\_VS\_LPS\_STIM\_NEUTROPHIL\_UP |  | 21 | -0.24 | -1.11 | 0.312 | 0.578 | 1.000 | 183 | tags=29%, list=13%, signal=32% |
| 1295 | GSE27241\_CTRL\_VS\_DIGOXIN\_TREATED\_CD4\_TCELL\_IN\_TH17\_POLARIZING\_CONDITIONS\_DN |  | 22 | -0.23 | -1.11 | 0.316 | 0.578 | 1.000 | 497 | tags=59%, list=35%, signal=90% |
| 1296 | GSE360\_T\_GONDII\_VS\_M\_TUBERCULOSIS\_MAC\_UP |  | 25 | -0.22 | -1.11 | 0.294 | 0.578 | 1.000 | 604 | tags=68%, list=43%, signal=117% |
| 1297 | GSE29618\_BCELL\_VS\_MDC\_DN |  | 19 | -0.25 | -1.11 | 0.309 | 0.579 | 1.000 | 455 | tags=53%, list=32%, signal=77% |
| 1298 | GSE22601\_DOUBLE\_POSITIVE\_VS\_CD4\_SINGLE\_POSITIVE\_THYMOCYTE\_UP |  | 17 | -0.26 | -1.11 | 0.290 | 0.579 | 1.000 | 145 | tags=29%, list=10%, signal=32% |
| 1299 | GSE2770\_IL4\_ACT\_VS\_ACT\_CD4\_TCELL\_48H\_UP |  | 18 | -0.26 | -1.11 | 0.305 | 0.579 | 1.000 | 221 | tags=33%, list=16%, signal=39% |
| 1300 | GSE14308\_TH1\_VS\_NAIVE\_CD4\_TCELL\_UP |  | 19 | -0.25 | -1.11 | 0.327 | 0.579 | 1.000 | 960 | tags=95%, list=68%, signal=292% |
| 1301 | GSE6269\_FLU\_VS\_STAPH\_AUREUS\_INF\_PBMC\_DN |  | 17 | -0.26 | -1.11 | 0.324 | 0.580 | 1.000 | 576 | tags=76%, list=41%, signal=128% |
| 1302 | GSE360\_T\_GONDII\_VS\_B\_MALAYI\_HIGH\_DOSE\_DC\_UP |  | 19 | -0.25 | -1.11 | 0.298 | 0.581 | 1.000 | 598 | tags=74%, list=42%, signal=126% |
| 1303 | GSE21379\_WT\_VS\_SAP\_KO\_TFH\_CD4\_TCELL\_UP |  | 21 | -0.24 | -1.11 | 0.320 | 0.581 | 1.000 | 561 | tags=67%, list=40%, signal=109% |
| 1304 | GSE17721\_PAM3CSK4\_VS\_CPG\_16H\_BMDC\_UP |  | 15 | -0.27 | -1.11 | 0.322 | 0.581 | 1.000 | 671 | tags=80%, list=48%, signal=151% |
| 1305 | GSE11864\_UNTREATED\_VS\_CSF1\_PAM3CYS\_IN\_MAC\_UP |  | 20 | -0.24 | -1.11 | 0.334 | 0.581 | 1.000 | 279 | tags=40%, list=20%, signal=49% |
| 1306 | GSE17721\_CTRL\_VS\_GARDIQUIMOD\_2H\_BMDC\_UP |  | 15 | -0.27 | -1.11 | 0.320 | 0.583 | 1.000 | 73 | tags=20%, list=5%, signal=21% |
| 1307 | GSE6092\_CTRL\_VS\_BORRELIA\_BIRGDOFERI\_INF\_ENDOTHELIAL\_CELL\_DN |  | 16 | -0.27 | -1.11 | 0.318 | 0.583 | 1.000 | 558 | tags=75%, list=40%, signal=123% |
| 1308 | GSE3982\_BCELL\_VS\_TH2\_DN |  | 17 | -0.26 | -1.11 | 0.320 | 0.583 | 1.000 | 132 | tags=24%, list=9%, signal=26% |
| 1309 | GSE18203\_CTRL\_VS\_INTRATUMORAL\_CPG\_INJ\_MC38\_TUMOR\_DN |  | 18 | -0.25 | -1.11 | 0.297 | 0.584 | 1.000 | 508 | tags=67%, list=36%, signal=103% |
| 1310 | GSE22886\_IGM\_MEMORY\_BCELL\_VS\_BLOOD\_PLASMA\_CELL\_UP |  | 17 | -0.26 | -1.11 | 0.338 | 0.584 | 1.000 | 156 | tags=29%, list=11%, signal=33% |
| 1311 | GSE36476\_CTRL\_VS\_TSST\_ACT\_40H\_MEMORY\_CD4\_TCELL\_OLD\_DN |  | 28 | -0.21 | -1.11 | 0.329 | 0.585 | 1.000 | 683 | tags=71%, list=48%, signal=136% |
| 1312 | GSE42021\_CD24HI\_VS\_CD24INT\_TCONV\_THYMUS\_UP |  | 24 | -0.23 | -1.11 | 0.317 | 0.585 | 1.000 | 195 | tags=25%, list=14%, signal=29% |
| 1313 | GSE12366\_GC\_VS\_NAIVE\_BCELL\_UP |  | 16 | -0.26 | -1.11 | 0.312 | 0.585 | 1.000 | 439 | tags=56%, list=31%, signal=81% |
| 1314 | GSE23321\_CENTRAL\_VS\_EFFECTOR\_MEMORY\_CD8\_TCELL\_DN |  | 15 | -0.28 | -1.11 | 0.331 | 0.584 | 1.000 | 599 | tags=73%, list=42%, signal=126% |
| 1315 | GSE20727\_H2O2\_VS\_ROS\_INHIBITOR\_TREATED\_DC\_DN |  | 35 | -0.20 | -1.11 | 0.340 | 0.585 | 1.000 | 182 | tags=20%, list=13%, signal=22% |
| 1316 | GSE17721\_LPS\_VS\_PAM3CSK4\_24H\_BMDC\_DN |  | 18 | -0.25 | -1.10 | 0.318 | 0.586 | 1.000 | 35 | tags=17%, list=2%, signal=17% |
| 1317 | GSE12845\_IGD\_POS\_BLOOD\_VS\_DARKZONE\_GC\_TONSIL\_BCELL\_UP |  | 18 | -0.26 | -1.10 | 0.314 | 0.586 | 1.000 | 206 | tags=33%, list=15%, signal=39% |
| 1318 | GSE25890\_CTRL\_VS\_IL33\_IL7\_TREATED\_NUOCYTES\_DN |  | 33 | -0.20 | -1.10 | 0.312 | 0.587 | 1.000 | 201 | tags=21%, list=14%, signal=24% |
| 1319 | GSE7460\_TCONV\_VS\_TREG\_THYMUS\_UP |  | 25 | -0.22 | -1.10 | 0.306 | 0.587 | 1.000 | 379 | tags=48%, list=27%, signal=64% |
| 1320 | GSE22886\_NAIVE\_CD4\_TCELL\_VS\_12H\_ACT\_TH2\_UP |  | 15 | -0.27 | -1.10 | 0.318 | 0.588 | 1.000 | 133 | tags=27%, list=9%, signal=29% |
| 1321 | GSE31082\_DN\_VS\_CD4\_SP\_THYMOCYTE\_UP |  | 25 | -0.22 | -1.10 | 0.337 | 0.587 | 1.000 | 176 | tags=24%, list=12%, signal=27% |
| 1322 | GSE32034\_UNTREATED\_VS\_ROSIGLIZATONE\_TREATED\_LY6C\_HIGH\_MONOCYTE\_DN |  | 20 | -0.24 | -1.10 | 0.303 | 0.587 | 1.000 | 845 | tags=90%, list=60%, signal=221% |
| 1323 | GSE3982\_EOSINOPHIL\_VS\_TH1\_UP |  | 17 | -0.26 | -1.10 | 0.347 | 0.589 | 1.000 | 498 | tags=65%, list=35%, signal=99% |
| 1324 | GSE29615\_CTRL\_VS\_DAY3\_LAIV\_IFLU\_VACCINE\_PBMC\_UP |  | 18 | -0.25 | -1.10 | 0.323 | 0.590 | 1.000 | 552 | tags=67%, list=39%, signal=108% |
| 1325 | GSE2405\_0H\_VS\_24H\_A\_PHAGOCYTOPHILUM\_STIM\_NEUTROPHIL\_DN |  | 15 | -0.27 | -1.10 | 0.310 | 0.591 | 1.000 | 70 | tags=20%, list=5%, signal=21% |
| 1326 | GSE40666\_WT\_VS\_STAT4\_KO\_CD8\_TCELL\_WITH\_IFNA\_STIM\_90MIN\_UP |  | 25 | -0.22 | -1.10 | 0.332 | 0.591 | 1.000 | 395 | tags=48%, list=28%, signal=65% |
| 1327 | GSE3982\_MAC\_VS\_TH2\_DN |  | 20 | -0.24 | -1.10 | 0.327 | 0.591 | 1.000 | 961 | tags=95%, list=68%, signal=294% |
| 1328 | GSE45739\_NRAS\_KO\_VS\_WT\_ACD3\_ACD28\_STIM\_CD4\_TCELL\_DN |  | 15 | -0.28 | -1.10 | 0.340 | 0.591 | 1.000 | 310 | tags=47%, list=22%, signal=59% |
| 1329 | GSE12845\_NAIVE\_VS\_DARKZONE\_GC\_TONSIL\_BCELL\_DN |  | 18 | -0.25 | -1.10 | 0.324 | 0.593 | 1.000 | 961 | tags=94%, list=68%, signal=292% |
| 1330 | GSE37534\_UNTREATED\_VS\_GW1929\_TREATED\_CD4\_TCELL\_PPARG1\_AND\_FOXP3\_TRASDUCED\_UP |  | 29 | -0.21 | -1.10 | 0.333 | 0.593 | 1.000 | 830 | tags=86%, list=59%, signal=205% |
| 1331 | GSE5679\_PPARG\_LIGAND\_ROSIGLITAZONE\_VS\_ROSIGLITAZONE\_AND\_RARA\_AGONIST\_AM580\_TREATED\_DC\_UP |  | 17 | -0.25 | -1.10 | 0.348 | 0.593 | 1.000 | 611 | tags=76%, list=43%, signal=133% |
| 1332 | GSE20500\_CTRL\_VS\_RETINOIC\_ACID\_TREATED\_CD4\_TCELL\_DN |  | 19 | -0.24 | -1.10 | 0.322 | 0.594 | 1.000 | 550 | tags=63%, list=39%, signal=102% |
| 1333 | GSE339\_CD8POS\_VS\_CD4CD8DN\_DC\_DN |  | 20 | -0.24 | -1.10 | 0.335 | 0.595 | 1.000 | 455 | tags=55%, list=32%, signal=80% |
| 1334 | GSE360\_L\_DONOVANI\_VS\_M\_TUBERCULOSIS\_DC\_UP |  | 19 | -0.24 | -1.10 | 0.329 | 0.595 | 1.000 | 140 | tags=26%, list=10%, signal=29% |
| 1335 | GSE3982\_MAST\_CELL\_VS\_NEUTROPHIL\_DN |  | 25 | -0.22 | -1.10 | 0.313 | 0.595 | 1.000 | 315 | tags=36%, list=22%, signal=46% |
| 1336 | GSE3982\_DC\_VS\_BCELL\_DN |  | 23 | -0.23 | -1.10 | 0.342 | 0.595 | 1.000 | 204 | tags=26%, list=14%, signal=30% |
| 1337 | GSE17721\_LPS\_VS\_POLYIC\_1H\_BMDC\_DN |  | 18 | -0.25 | -1.10 | 0.325 | 0.595 | 1.000 | 356 | tags=39%, list=25%, signal=51% |
| 1338 | GSE16385\_ROSIGLITAZONE\_IL4\_VS\_ROSIGLITAZONE\_ALONE\_STIM\_MACROPHAGE\_DN |  | 18 | -0.25 | -1.09 | 0.313 | 0.596 | 1.000 | 209 | tags=28%, list=15%, signal=32% |
| 1339 | GSE27786\_ERYTHROBLAST\_VS\_NEUTROPHIL\_UP |  | 16 | -0.27 | -1.09 | 0.328 | 0.595 | 1.000 | 1040 | tags=100%, list=74%, signal=376% |
| 1340 | GSE9988\_LPS\_VS\_LPS\_AND\_ANTI\_TREM1\_MONOCYTE\_UP |  | 24 | -0.23 | -1.09 | 0.346 | 0.596 | 1.000 | 266 | tags=33%, list=19%, signal=40% |
| 1341 | GSE10239\_NAIVE\_VS\_KLRG1INT\_EFF\_CD8\_TCELL\_UP |  | 25 | -0.22 | -1.09 | 0.332 | 0.596 | 1.000 | 538 | tags=64%, list=38%, signal=102% |
| 1342 | GSE41176\_WT\_VS\_TAK1\_KO\_ANTI\_IGM\_STIM\_BCELL\_24H\_DN |  | 25 | -0.22 | -1.09 | 0.326 | 0.596 | 1.000 | 335 | tags=40%, list=24%, signal=52% |
| 1343 | GSE40274\_HELIOS\_VS\_FOXP3\_AND\_HELIOS\_TRANSDUCED\_ACTIVATED\_CD4\_TCELL\_UP |  | 16 | -0.26 | -1.09 | 0.348 | 0.596 | 1.000 | 8 | tags=13%, list=1%, signal=12% |
| 1344 | GSE20152\_HTNFA\_OVERXPRESS\_ANKLE\_VS\_CTRL\_SPHK1\_KO\_ANKLE\_UP |  | 20 | -0.24 | -1.09 | 0.345 | 0.596 | 1.000 | 128 | tags=20%, list=9%, signal=22% |
| 1345 | GSE1925\_3H\_VS\_24H\_IFNG\_STIM\_IFNG\_PRIMED\_MACROPHAGE\_DN |  | 20 | -0.24 | -1.09 | 0.333 | 0.597 | 1.000 | 295 | tags=40%, list=21%, signal=50% |
| 1346 | GSE17721\_CTRL\_VS\_GARDIQUIMOD\_8H\_BMDC\_DN |  | 16 | -0.26 | -1.09 | 0.336 | 0.596 | 1.000 | 202 | tags=31%, list=14%, signal=36% |
| 1347 | GSE3982\_MAST\_CELL\_VS\_TH1\_UP |  | 15 | -0.27 | -1.09 | 0.341 | 0.599 | 1.000 | 242 | tags=33%, list=17%, signal=40% |
| 1348 | GSE37534\_GW1929\_VS\_PIOGLITAZONE\_TREATED\_CD4\_TCELL\_PPARG1\_FOXP3\_TRANSDUCED\_UP |  | 18 | -0.25 | -1.09 | 0.328 | 0.599 | 1.000 | 315 | tags=44%, list=22%, signal=56% |
| 1349 | GSE32533\_WT\_VS\_MIR17\_KO\_ACT\_CD4\_TCELL\_UP |  | 25 | -0.22 | -1.09 | 0.341 | 0.601 | 1.000 | 183 | tags=28%, list=13%, signal=32% |
| 1350 | GSE29949\_CD8\_POS\_DC\_SPLEEN\_VS\_MONOCYTE\_BONE\_MARROW\_UP |  | 15 | -0.27 | -1.09 | 0.341 | 0.601 | 1.000 | 781 | tags=87%, list=55%, signal=192% |
| 1351 | GSE7348\_LPS\_VS\_TOLERIZED\_AND\_LPS\_STIM\_MACROPHAGE\_UP |  | 27 | -0.22 | -1.09 | 0.335 | 0.601 | 1.000 | 1107 | tags=100%, list=78%, signal=455% |
| 1352 | GSE16697\_CD4\_TCELL\_VS\_TFH\_CD4\_TCELL\_DN |  | 18 | -0.25 | -1.09 | 0.341 | 0.600 | 1.000 | 525 | tags=67%, list=37%, signal=105% |
| 1353 | GSE33425\_CD161\_HIGH\_VS\_NEG\_CD8\_TCELL\_UP |  | 15 | -0.27 | -1.09 | 0.342 | 0.600 | 1.000 | 303 | tags=40%, list=21%, signal=50% |
| 1354 | GSE9650\_EXHAUSTED\_VS\_MEMORY\_CD8\_TCELL\_DN |  | 18 | -0.24 | -1.09 | 0.352 | 0.600 | 1.000 | 1073 | tags=100%, list=76%, signal=412% |
| 1355 | GSE21774\_CD62L\_POS\_CD56\_BRIGHT\_VS\_CD62L\_NEG\_CD56\_DIM\_NK\_CELL\_UP |  | 16 | -0.25 | -1.09 | 0.326 | 0.601 | 1.000 | 298 | tags=38%, list=21%, signal=47% |
| 1356 | GSE10239\_MEMORY\_VS\_KLRG1HIGH\_EFF\_CD8\_TCELL\_UP |  | 18 | -0.25 | -1.09 | 0.341 | 0.603 | 1.000 | 1067 | tags=100%, list=76%, signal=405% |
| 1357 | GSE12198\_CTRL\_VS\_HIGH\_IL2\_STIM\_NK\_CELL\_DN |  | 15 | -0.26 | -1.09 | 0.366 | 0.604 | 1.000 | 914 | tags=93%, list=65%, signal=262% |
| 1358 | GSE11961\_MEMORY\_BCELL\_DAY7\_VS\_GERMINAL\_CENTER\_BCELL\_DAY7\_DN |  | 15 | -0.27 | -1.09 | 0.351 | 0.603 | 1.000 | 448 | tags=60%, list=32%, signal=87% |
| 1359 | GSE360\_DC\_VS\_MAC\_B\_MALAYI\_LOW\_DOSE\_DN |  | 16 | -0.26 | -1.09 | 0.339 | 0.603 | 1.000 | 851 | tags=88%, list=60%, signal=218% |
| 1360 | GSE45365\_WT\_VS\_IFNAR\_KO\_CD8A\_DC\_MCMV\_INFECTION\_UP |  | 25 | -0.22 | -1.09 | 0.354 | 0.604 | 1.000 | 240 | tags=36%, list=17%, signal=43% |
| 1361 | GSE17721\_CTRL\_VS\_PAM3CSK4\_4H\_BMDC\_DN |  | 16 | -0.26 | -1.08 | 0.340 | 0.605 | 1.000 | 78 | tags=19%, list=6%, signal=20% |
| 1362 | GSE17721\_LPS\_VS\_POLYIC\_8H\_BMDC\_DN |  | 20 | -0.24 | -1.08 | 0.343 | 0.605 | 1.000 | 496 | tags=55%, list=35%, signal=84% |
| 1363 | GSE7852\_TREG\_VS\_TCONV\_UP |  | 26 | -0.21 | -1.08 | 0.364 | 0.609 | 1.000 | 387 | tags=46%, list=27%, signal=62% |
| 1364 | GSE5589\_LPS\_VS\_LPS\_AND\_IL6\_STIM\_IL6\_KO\_MACROPHAGE\_45MIN\_DN |  | 17 | -0.25 | -1.08 | 0.344 | 0.611 | 1.000 | 188 | tags=29%, list=13%, signal=34% |
| 1365 | GSE37301\_HEMATOPOIETIC\_STEM\_CELL\_VS\_PRO\_BCELL\_UP |  | 18 | -0.25 | -1.08 | 0.351 | 0.611 | 1.000 | 547 | tags=67%, list=39%, signal=107% |
| 1366 | GSE15330\_WT\_VS\_IKAROS\_KO\_GRANULOCYTE\_MONOCYTE\_PROGENITOR\_DN |  | 17 | -0.25 | -1.08 | 0.359 | 0.612 | 1.000 | 599 | tags=76%, list=42%, signal=131% |
| 1367 | GSE14308\_TH2\_VS\_NATURAL\_TREG\_UP |  | 17 | -0.25 | -1.08 | 0.364 | 0.612 | 1.000 | 857 | tags=88%, list=61%, signal=222% |
| 1368 | GSE360\_CTRL\_VS\_B\_MALAYI\_LOW\_DOSE\_DC\_DN |  | 18 | -0.25 | -1.08 | 0.355 | 0.612 | 1.000 | 341 | tags=44%, list=24%, signal=58% |
| 1369 | GSE17721\_0.5H\_VS\_4H\_POLYIC\_BMDC\_DN |  | 21 | -0.23 | -1.08 | 0.357 | 0.611 | 1.000 | 578 | tags=71%, list=41%, signal=119% |
| 1370 | GSE3982\_BASOPHIL\_VS\_TH2\_UP |  | 29 | -0.21 | -1.08 | 0.365 | 0.612 | 1.000 | 498 | tags=55%, list=35%, signal=84% |
| 1371 | GSE42724\_NAIVE\_BCELL\_VS\_PLASMABLAST\_UP |  | 23 | -0.22 | -1.08 | 0.345 | 0.614 | 1.000 | 456 | tags=52%, list=32%, signal=76% |
| 1372 | GSE28737\_WT\_VS\_BCL6\_KO\_FOLLICULAR\_BCELL\_UP |  | 23 | -0.22 | -1.08 | 0.363 | 0.614 | 1.000 | 73 | tags=17%, list=5%, signal=18% |
| 1373 | GSE17721\_PAM3CSK4\_VS\_CPG\_8H\_BMDC\_UP |  | 29 | -0.21 | -1.08 | 0.340 | 0.614 | 1.000 | 45 | tags=14%, list=3%, signal=14% |
| 1374 | GSE24574\_BCL6\_LOW\_TFH\_VS\_TCONV\_CD4\_TCELL\_DN |  | 17 | -0.25 | -1.08 | 0.329 | 0.614 | 1.000 | 929 | tags=94%, list=66%, signal=272% |
| 1375 | GSE40274\_GATA1\_VS\_FOXP3\_AND\_GATA1\_TRANSDUCED\_ACTIVATED\_CD4\_TCELL\_UP |  | 23 | -0.22 | -1.08 | 0.349 | 0.614 | 1.000 | 327 | tags=39%, list=23%, signal=50% |
| 1376 | GSE3720\_UNSTIM\_VS\_PMA\_STIM\_VD2\_GAMMADELTA\_TCELL\_DN |  | 16 | -0.26 | -1.08 | 0.357 | 0.614 | 1.000 | 150 | tags=25%, list=11%, signal=28% |
| 1377 | GSE22886\_NAIVE\_BCELL\_VS\_BLOOD\_PLASMA\_CELL\_UP |  | 27 | -0.21 | -1.08 | 0.360 | 0.613 | 1.000 | 511 | tags=59%, list=36%, signal=91% |
| 1378 | GSE11961\_FOLLICULAR\_BCELL\_VS\_GERMINAL\_CENTER\_BCELL\_DAY40\_DN |  | 17 | -0.25 | -1.08 | 0.338 | 0.614 | 1.000 | 61 | tags=18%, list=4%, signal=18% |
| 1379 | GSE19923\_HEB\_KO\_VS\_HEB\_AND\_E2A\_KO\_DP\_THYMOCYTE\_UP |  | 27 | -0.21 | -1.07 | 0.353 | 0.616 | 1.000 | 515 | tags=56%, list=36%, signal=86% |
| 1380 | GSE14699\_NAIVE\_VS\_DELETIONAL\_TOLERANCE\_CD8\_TCELL\_UP |  | 17 | -0.25 | -1.07 | 0.352 | 0.616 | 1.000 | 6 | tags=12%, list=0%, signal=12% |
| 1381 | GSE37563\_WT\_VS\_CTLA4\_KO\_CD4\_TCELL\_D4\_POST\_IMMUNIZATION\_DN |  | 21 | -0.23 | -1.07 | 0.345 | 0.616 | 1.000 | 162 | tags=24%, list=11%, signal=26% |
| 1382 | GSE360\_T\_GONDII\_VS\_B\_MALAYI\_LOW\_DOSE\_DC\_UP |  | 19 | -0.24 | -1.07 | 0.350 | 0.616 | 1.000 | 562 | tags=68%, list=40%, signal=112% |
| 1383 | GSE28737\_WT\_VS\_BCL6\_HET\_FOLLICULAR\_BCELL\_DN |  | 16 | -0.27 | -1.07 | 0.341 | 0.616 | 1.000 | 34 | tags=13%, list=2%, signal=13% |
| 1384 | GSE42021\_TREG\_PLN\_VS\_TREG\_PRECURSORS\_THYMUS\_DN |  | 31 | -0.20 | -1.07 | 0.373 | 0.616 | 1.000 | 458 | tags=52%, list=32%, signal=75% |
| 1385 | GSE44649\_WT\_VS\_MIR155\_KO\_NAIVE\_CD8\_TCELL\_UP |  | 15 | -0.26 | -1.07 | 0.368 | 0.618 | 1.000 | 236 | tags=33%, list=17%, signal=40% |
| 1386 | GSE15735\_CTRL\_VS\_HDAC\_INHIBITOR\_TREATED\_CD4\_TCELL\_2H\_UP |  | 22 | -0.22 | -1.07 | 0.374 | 0.619 | 1.000 | 886 | tags=91%, list=63%, signal=241% |
| 1387 | GSE17721\_LPS\_VS\_PAM3CSK4\_12H\_BMDC\_UP |  | 17 | -0.25 | -1.07 | 0.352 | 0.619 | 1.000 | 362 | tags=47%, list=26%, signal=63% |
| 1388 | GSE13484\_UNSTIM\_VS\_3H\_YF17D\_VACCINE\_STIM\_PBMC\_DN |  | 24 | -0.22 | -1.07 | 0.377 | 0.619 | 1.000 | 278 | tags=38%, list=20%, signal=46% |
| 1389 | GSE20754\_WT\_VS\_TCF1\_KO\_MEMORY\_CD8\_TCELL\_DN |  | 22 | -0.23 | -1.07 | 0.354 | 0.621 | 1.000 | 1015 | tags=95%, list=72%, signal=335% |
| 1390 | GSE37301\_RAG2\_KO\_VS\_RAG2\_AND\_ETS1\_KO\_NK\_CELL\_UP |  | 23 | -0.22 | -1.07 | 0.371 | 0.623 | 1.000 | 919 | tags=91%, list=65%, signal=258% |
| 1391 | GSE17721\_LPS\_VS\_CPG\_6H\_BMDC\_UP |  | 20 | -0.24 | -1.07 | 0.381 | 0.622 | 1.000 | 533 | tags=60%, list=38%, signal=95% |
| 1392 | GSE37301\_CD4\_TCELL\_VS\_RAG2\_KO\_NK\_CELL\_DN |  | 21 | -0.23 | -1.07 | 0.387 | 0.623 | 1.000 | 408 | tags=48%, list=29%, signal=66% |
| 1393 | GSE42021\_TREG\_PLN\_VS\_CD24INT\_TREG\_THYMUS\_DN |  | 23 | -0.22 | -1.07 | 0.372 | 0.623 | 1.000 | 562 | tags=65%, list=40%, signal=107% |
| 1394 | GSE17186\_BLOOD\_VS\_CORD\_BLOOD\_CD21LOW\_TRANSITIONAL\_BCELL\_UP |  | 17 | -0.24 | -1.07 | 0.365 | 0.625 | 1.000 | 252 | tags=35%, list=18%, signal=42% |
| 1395 | GSE27670\_CTRL\_VS\_BLIMP1\_TRANSDUCED\_GC\_BCELL\_DN |  | 22 | -0.22 | -1.07 | 0.364 | 0.626 | 1.000 | 564 | tags=68%, list=40%, signal=112% |
| 1396 | GSE21063\_3H\_VS\_16H\_ANTI\_IGM\_STIM\_NFATC1\_KOBCELL\_UP |  | 19 | -0.24 | -1.07 | 0.368 | 0.626 | 1.000 | 605 | tags=74%, list=43%, signal=127% |
| 1397 | GSE21670\_TGFB\_VS\_TGFB\_AND\_IL6\_TREATED\_STAT3\_KO\_CD4\_TCELL\_UP |  | 29 | -0.20 | -1.07 | 0.370 | 0.626 | 1.000 | 308 | tags=38%, list=22%, signal=48% |
| 1398 | GSE41867\_NAIVE\_VS\_DAY6\_LCMV\_EFFECTOR\_CD8\_TCELL\_DN |  | 17 | -0.24 | -1.07 | 0.381 | 0.626 | 1.000 | 125 | tags=24%, list=9%, signal=26% |
| 1399 | GSE2706\_R848\_VS\_LPS\_8H\_STIM\_DC\_UP |  | 16 | -0.25 | -1.07 | 0.374 | 0.625 | 1.000 | 197 | tags=31%, list=14%, signal=36% |
| 1400 | GSE22886\_UNSTIM\_VS\_IL2\_STIM\_NKCELL\_UP |  | 25 | -0.22 | -1.07 | 0.393 | 0.625 | 1.000 | 513 | tags=64%, list=36%, signal=99% |
| 1401 | GSE29949\_DC\_BRAIN\_VS\_MONOCYTE\_BONE\_MARROW\_UP |  | 26 | -0.21 | -1.06 | 0.384 | 0.628 | 1.000 | 379 | tags=46%, list=27%, signal=62% |
| 1402 | GSE20366\_EX\_VIVO\_VS\_HOMEOSTATIC\_CONVERSION\_NAIVE\_CD4\_TCELL\_UP |  | 26 | -0.21 | -1.06 | 0.383 | 0.628 | 1.000 | 218 | tags=31%, list=15%, signal=36% |
| 1403 | GSE46606\_IRF4\_KO\_VS\_WT\_UNSTIM\_BCELL\_DN |  | 20 | -0.23 | -1.06 | 0.397 | 0.628 | 1.000 | 214 | tags=30%, list=15%, signal=35% |
| 1404 | GSE3982\_BCELL\_VS\_TH1\_DN |  | 24 | -0.22 | -1.06 | 0.373 | 0.628 | 1.000 | 290 | tags=38%, list=21%, signal=46% |
| 1405 | GSE30083\_SP1\_VS\_SP3\_THYMOCYTE\_DN |  | 16 | -0.25 | -1.06 | 0.374 | 0.628 | 1.000 | 1057 | tags=100%, list=75%, signal=394% |
| 1406 | GSE2706\_R848\_VS\_R848\_AND\_LPS\_8H\_STIM\_DC\_UP |  | 19 | -0.24 | -1.06 | 0.392 | 0.628 | 1.000 | 233 | tags=37%, list=17%, signal=44% |
| 1407 | GSE40666\_WT\_VS\_STAT1\_KO\_CD8\_TCELL\_WITH\_IFNA\_STIM\_90MIN\_UP |  | 31 | -0.20 | -1.06 | 0.387 | 0.629 | 1.000 | 410 | tags=48%, list=29%, signal=67% |
| 1408 | GSE17186\_BLOOD\_VS\_CORD\_BLOOD\_NAIVE\_BCELL\_UP |  | 24 | -0.22 | -1.06 | 0.374 | 0.629 | 1.000 | 117 | tags=21%, list=8%, signal=22% |
| 1409 | GSE5679\_PPARG\_LIGAND\_ROSIGLITAZONE\_VS\_RARA\_AGONIST\_AM580\_TREATED\_DC\_DN |  | 25 | -0.21 | -1.06 | 0.377 | 0.629 | 1.000 | 456 | tags=52%, list=32%, signal=75% |
| 1410 | GSE46606\_IRF4HIGH\_VS\_WT\_CD40L\_IL2\_IL5\_DAY1\_STIMULATED\_BCELL\_DN |  | 23 | -0.22 | -1.06 | 0.391 | 0.629 | 1.000 | 381 | tags=43%, list=27%, signal=59% |
| 1411 | GSE9006\_HEALTHY\_VS\_TYPE\_2\_DIABETES\_PBMC\_AT\_DX\_DN |  | 18 | -0.24 | -1.06 | 0.374 | 0.630 | 1.000 | 237 | tags=33%, list=17%, signal=40% |
| 1412 | GSE22886\_CD4\_TCELL\_VS\_BCELL\_NAIVE\_DN |  | 18 | -0.24 | -1.06 | 0.350 | 0.630 | 1.000 | 504 | tags=61%, list=36%, signal=94% |
| 1413 | GSE32901\_NAIVE\_VS\_TH17\_ENRICHED\_CD4\_TCELL\_UP |  | 15 | -0.26 | -1.06 | 0.387 | 0.629 | 1.000 | 782 | tags=87%, list=55%, signal=192% |
| 1414 | GSE23321\_CENTRAL\_MEMORY\_VS\_NAIVE\_CD8\_TCELL\_DN |  | 15 | -0.26 | -1.06 | 0.363 | 0.629 | 1.000 | 898 | tags=93%, list=64%, signal=254% |
| 1415 | GSE39556\_CD8A\_DC\_VS\_NK\_CELL\_MOUSE\_3H\_POST\_POLYIC\_INJ\_UP |  | 21 | -0.23 | -1.06 | 0.369 | 0.629 | 1.000 | 114 | tags=19%, list=8%, signal=20% |
| 1416 | GSE339\_CD4POS\_VS\_CD4CD8DN\_DC\_DN |  | 22 | -0.22 | -1.06 | 0.352 | 0.631 | 1.000 | 532 | tags=64%, list=38%, signal=101% |
| 1417 | GSE35825\_UNTREATED\_VS\_IFNG\_STIM\_MACROPHAGE\_DN |  | 26 | -0.21 | -1.06 | 0.372 | 0.631 | 1.000 | 425 | tags=46%, list=30%, signal=65% |
| 1418 | GSE24574\_BCL6\_HIGH\_TFH\_VS\_NAIVE\_CD4\_TCELL\_DN |  | 25 | -0.21 | -1.06 | 0.361 | 0.631 | 1.000 | 84 | tags=12%, list=6%, signal=13% |
| 1419 | GSE17721\_LPS\_VS\_GARDIQUIMOD\_1H\_BMDC\_UP |  | 25 | -0.21 | -1.06 | 0.361 | 0.630 | 1.000 | 508 | tags=60%, list=36%, signal=92% |
| 1420 | GSE21033\_3H\_VS\_24H\_POLYIC\_STIM\_DC\_UP |  | 16 | -0.25 | -1.06 | 0.362 | 0.631 | 1.000 | 438 | tags=56%, list=31%, signal=81% |
| 1421 | GSE8868\_SPLEEN\_VS\_INTESTINE\_CD11B\_POS\_CD11C\_NEG\_DC\_UP |  | 18 | -0.25 | -1.06 | 0.382 | 0.632 | 1.000 | 374 | tags=44%, list=27%, signal=60% |
| 1422 | GSE19401\_PAM2CSK4\_VS\_RETINOIC\_ACID\_STIM\_FOLLICULAR\_DC\_UP |  | 20 | -0.23 | -1.06 | 0.393 | 0.632 | 1.000 | 240 | tags=35%, list=17%, signal=42% |
| 1423 | GSE22886\_NAIVE\_CD4\_TCELL\_VS\_12H\_ACT\_TH1\_UP |  | 20 | -0.24 | -1.06 | 0.382 | 0.632 | 1.000 | 35 | tags=15%, list=2%, signal=15% |
| 1424 | GSE43863\_DAY6\_EFF\_VS\_DAY150\_MEM\_TH1\_CD4\_TCELL\_DN |  | 21 | -0.23 | -1.06 | 0.378 | 0.632 | 1.000 | 214 | tags=29%, list=15%, signal=33% |
| 1425 | GSE4748\_CTRL\_VS\_LPS\_STIM\_DC\_3H\_UP |  | 22 | -0.23 | -1.06 | 0.369 | 0.633 | 1.000 | 430 | tags=50%, list=30%, signal=71% |
| 1426 | GSE9037\_WT\_VS\_IRAK4\_KO\_LPS\_4H\_STIM\_BMDM\_UP |  | 26 | -0.21 | -1.06 | 0.402 | 0.633 | 1.000 | 324 | tags=38%, list=23%, signal=49% |
| 1427 | GSE17721\_CTRL\_VS\_CPG\_2H\_BMDC\_DN |  | 19 | -0.23 | -1.05 | 0.417 | 0.633 | 1.000 | 224 | tags=32%, list=16%, signal=37% |
| 1428 | GSE9960\_HEALTHY\_VS\_GRAM\_POS\_SEPSIS\_PBMC\_DN |  | 20 | -0.22 | -1.05 | 0.397 | 0.634 | 1.000 | 926 | tags=90%, list=66%, signal=258% |
| 1429 | GSE43957\_UNTREATED\_VS\_NACL\_TREATED\_ANTI\_CD3\_CD28\_STIM\_CD4\_TCELL\_UP |  | 17 | -0.24 | -1.05 | 0.366 | 0.635 | 1.000 | 200 | tags=29%, list=14%, signal=34% |
| 1430 | GSE17721\_CPG\_VS\_GARDIQUIMOD\_24H\_BMDC\_UP |  | 29 | -0.20 | -1.05 | 0.382 | 0.637 | 1.000 | 584 | tags=69%, list=41%, signal=115% |
| 1431 | GSE360\_L\_MAJOR\_VS\_B\_MALAYI\_HIGH\_DOSE\_MAC\_DN |  | 23 | -0.22 | -1.05 | 0.388 | 0.638 | 1.000 | 172 | tags=26%, list=12%, signal=29% |
| 1432 | GSE43955\_TH0\_VS\_TGFB\_IL6\_TH17\_ACT\_CD4\_TCELL\_52H\_UP |  | 26 | -0.21 | -1.05 | 0.402 | 0.638 | 1.000 | 500 | tags=58%, list=35%, signal=88% |
| 1433 | GSE18893\_CTRL\_VS\_TNF\_TREATED\_TREG\_2H\_UP |  | 32 | -0.20 | -1.05 | 0.378 | 0.638 | 1.000 | 302 | tags=38%, list=21%, signal=47% |
| 1434 | GSE19374\_UNINF\_VS\_LISTERIA\_INFECTED\_MACROPHAGE\_DN |  | 18 | -0.24 | -1.05 | 0.393 | 0.640 | 1.000 | 516 | tags=67%, list=37%, signal=104% |
| 1435 | GSE21670\_UNTREATED\_VS\_TGFB\_IL6\_TREATED\_CD4\_TCELL\_UP |  | 17 | -0.25 | -1.05 | 0.377 | 0.639 | 1.000 | 290 | tags=41%, list=21%, signal=51% |
| 1436 | GSE31082\_DN\_VS\_DP\_THYMOCYTE\_DN |  | 16 | -0.25 | -1.05 | 0.405 | 0.639 | 1.000 | 414 | tags=50%, list=29%, signal=70% |
| 1437 | GSE2706\_2H\_VS\_8H\_LPS\_STIM\_DC\_DN |  | 16 | -0.24 | -1.05 | 0.361 | 0.642 | 1.000 | 273 | tags=38%, list=19%, signal=46% |
| 1438 | GSE16385\_MONOCYTE\_VS\_MACROPHAGE\_UP |  | 18 | -0.24 | -1.05 | 0.418 | 0.641 | 1.000 | 886 | tags=89%, list=63%, signal=236% |
| 1439 | GSE17186\_MEMORY\_VS\_CD21HIGH\_TRANSITIONAL\_BCELL\_DN |  | 16 | -0.25 | -1.05 | 0.388 | 0.641 | 1.000 | 817 | tags=88%, list=58%, signal=205% |
| 1440 | GSE20152\_HTNFA\_OVERXPRESS\_ANKLE\_VS\_CTRL\_SPHK1\_KO\_ANKLE\_DN |  | 20 | -0.23 | -1.05 | 0.404 | 0.642 | 1.000 | 222 | tags=30%, list=16%, signal=35% |
| 1441 | GSE17974\_0H\_VS\_2H\_IN\_VITRO\_ACT\_CD4\_TCELL\_UP |  | 19 | -0.24 | -1.05 | 0.403 | 0.644 | 1.000 | 168 | tags=26%, list=12%, signal=29% |
| 1442 | GSE39556\_UNTREATED\_VS\_3H\_POLYIC\_INJ\_MOUSE\_NK\_CELL\_UP |  | 22 | -0.22 | -1.05 | 0.409 | 0.644 | 1.000 | 132 | tags=23%, list=9%, signal=25% |
| 1443 | GSE11864\_CSF1\_PAM3CYS\_VS\_CSF1\_IFNG\_PAM3CYS\_IN\_MAC\_UP |  | 29 | -0.20 | -1.04 | 0.409 | 0.646 | 1.000 | 107 | tags=17%, list=8%, signal=18% |
| 1444 | GSE43955\_TH0\_VS\_TGFB\_IL6\_TH17\_ACT\_CD4\_TCELL\_42H\_UP |  | 26 | -0.21 | -1.04 | 0.425 | 0.646 | 1.000 | 781 | tags=81%, list=55%, signal=178% |
| 1445 | GSE18893\_TCONV\_VS\_TREG\_24H\_CULTURE\_DN |  | 23 | -0.21 | -1.04 | 0.397 | 0.646 | 1.000 | 469 | tags=52%, list=33%, signal=77% |
| 1446 | GSE19941\_LPS\_VS\_LPS\_AND\_IL10\_STIM\_IL10\_KO\_NFKBP50\_KO\_MACROPHAGE\_UP |  | 25 | -0.21 | -1.04 | 0.404 | 0.646 | 1.000 | 511 | tags=60%, list=36%, signal=92% |
| 1447 | GSE34006\_WT\_VS\_A2AR\_KO\_TREG\_DN |  | 22 | -0.22 | -1.04 | 0.401 | 0.646 | 1.000 | 315 | tags=36%, list=22%, signal=46% |
| 1448 | GSE43955\_TGFB\_IL6\_VS\_TGFB\_IL6\_IL23\_TH17\_ACT\_CD4\_TCELL\_52H\_UP |  | 18 | -0.24 | -1.04 | 0.411 | 0.646 | 1.000 | 984 | tags=94%, list=70%, signal=308% |
| 1449 | GSE369\_SOCS3\_KO\_VS\_WT\_LIVER\_POST\_IL6\_INJECTION\_UP |  | 19 | -0.23 | -1.04 | 0.405 | 0.646 | 1.000 | 190 | tags=26%, list=13%, signal=30% |
| 1450 | GSE12366\_GC\_VS\_MEMORY\_BCELL\_DN |  | 19 | -0.22 | -1.04 | 0.402 | 0.648 | 1.000 | 423 | tags=53%, list=30%, signal=74% |
| 1451 | GSE14308\_TH1\_VS\_NAIVE\_CD4\_TCELL\_DN |  | 20 | -0.23 | -1.04 | 0.408 | 0.648 | 1.000 | 220 | tags=30%, list=16%, signal=35% |
| 1452 | GSE30971\_CTRL\_VS\_LPS\_STIM\_MACROPHAGE\_WBP7\_KO\_2H\_DN |  | 17 | -0.24 | -1.04 | 0.388 | 0.649 | 1.000 | 558 | tags=65%, list=40%, signal=106% |
| 1453 | GSE18791\_CTRL\_VS\_NEWCASTLE\_VIRUS\_DC\_16H\_DN |  | 25 | -0.21 | -1.04 | 0.389 | 0.649 | 1.000 | 523 | tags=56%, list=37%, signal=87% |
| 1454 | GSE3039\_CD4\_TCELL\_VS\_NKT\_CELL\_DN |  | 17 | -0.25 | -1.04 | 0.393 | 0.649 | 1.000 | 183 | tags=29%, list=13%, signal=33% |
| 1455 | GSE42088\_UNINF\_VS\_LEISHMANIA\_INF\_DC\_4H\_DN |  | 23 | -0.22 | -1.04 | 0.376 | 0.649 | 1.000 | 546 | tags=70%, list=39%, signal=112% |
| 1456 | GSE2770\_TGFB\_AND\_IL4\_ACT\_VS\_ACT\_CD4\_TCELL\_2H\_UP |  | 17 | -0.24 | -1.04 | 0.392 | 0.649 | 1.000 | 557 | tags=65%, list=39%, signal=106% |
| 1457 | GSE24634\_IL4\_VS\_CTRL\_TREATED\_NAIVE\_CD4\_TCELL\_DAY5\_DN |  | 19 | -0.23 | -1.04 | 0.390 | 0.648 | 1.000 | 458 | tags=53%, list=32%, signal=77% |
| 1458 | GSE27786\_LSK\_VS\_MONO\_MAC\_DN |  | 17 | -0.23 | -1.04 | 0.412 | 0.648 | 1.000 | 77 | tags=18%, list=5%, signal=18% |
| 1459 | GSE5099\_CLASSICAL\_M1\_VS\_ALTERNATIVE\_M2\_MACROPHAGE\_UP |  | 15 | -0.26 | -1.04 | 0.395 | 0.649 | 1.000 | 445 | tags=53%, list=32%, signal=77% |
| 1460 | GSE22432\_MULTIPOTENT\_PROGENITOR\_VS\_CDC\_DN |  | 26 | -0.21 | -1.04 | 0.381 | 0.648 | 1.000 | 216 | tags=27%, list=15%, signal=31% |
| 1461 | GSE3337\_4H\_VS\_16H\_IFNG\_IN\_CD8POS\_DC\_UP |  | 25 | -0.20 | -1.04 | 0.379 | 0.648 | 1.000 | 618 | tags=68%, list=44%, signal=119% |
| 1462 | GSE7548\_NAIVE\_VS\_DAY7\_PCC\_IMMUNIZATION\_CD4\_TCELL\_DN |  | 26 | -0.20 | -1.04 | 0.415 | 0.648 | 1.000 | 513 | tags=54%, list=36%, signal=83% |
| 1463 | GSE17721\_PAM3CSK4\_VS\_CPG\_6H\_BMDC\_DN |  | 15 | -0.25 | -1.04 | 0.402 | 0.649 | 1.000 | 80 | tags=20%, list=6%, signal=21% |
| 1464 | GSE43955\_10H\_VS\_60H\_ACT\_CD4\_TCELL\_WITH\_TGFB\_IL6\_UP |  | 16 | -0.25 | -1.04 | 0.399 | 0.648 | 1.000 | 956 | tags=94%, list=68%, signal=287% |
| 1465 | GSE17580\_TREG\_VS\_TEFF\_DN |  | 17 | -0.24 | -1.04 | 0.394 | 0.648 | 1.000 | 455 | tags=53%, list=32%, signal=77% |
| 1466 | GSE17580\_TREG\_VS\_TEFF\_S\_MANSONI\_INF\_DN |  | 17 | -0.24 | -1.04 | 0.385 | 0.648 | 1.000 | 472 | tags=59%, list=33%, signal=87% |
| 1467 | GSE17721\_POLYIC\_VS\_PAM3CSK4\_0.5H\_BMDC\_DN |  | 19 | -0.23 | -1.04 | 0.418 | 0.648 | 1.000 | 314 | tags=37%, list=22%, signal=47% |
| 1468 | GSE40666\_WT\_VS\_STAT4\_KO\_CD8\_TCELL\_WITH\_IFNA\_STIM\_90MIN\_DN |  | 16 | -0.25 | -1.04 | 0.392 | 0.648 | 1.000 | 278 | tags=38%, list=20%, signal=46% |
| 1469 | GSE22935\_UNSTIM\_VS\_48H\_MBOVIS\_BCG\_STIM\_MACROPHAGE\_DN |  | 19 | -0.23 | -1.04 | 0.378 | 0.648 | 1.000 | 396 | tags=53%, list=28%, signal=72% |
| 1470 | GSE26669\_CD4\_VS\_CD8\_TCELL\_IN\_MLR\_UP |  | 23 | -0.21 | -1.04 | 0.419 | 0.647 | 1.000 | 253 | tags=30%, list=18%, signal=36% |
| 1471 | GSE17721\_CPG\_VS\_GARDIQUIMOD\_8H\_BMDC\_UP |  | 22 | -0.22 | -1.04 | 0.406 | 0.647 | 1.000 | 675 | tags=73%, list=48%, signal=137% |
| 1472 | GSE12392\_CD8A\_POS\_VS\_NEG\_SPLEEN\_IFNB\_KO\_DC\_DN |  | 17 | -0.24 | -1.04 | 0.406 | 0.649 | 1.000 | 573 | tags=65%, list=41%, signal=108% |
| 1473 | GSE7831\_UNSTIM\_VS\_INFLUENZA\_STIM\_PDC\_4H\_DN |  | 18 | -0.24 | -1.04 | 0.394 | 0.649 | 1.000 | 589 | tags=67%, list=42%, signal=113% |
| 1474 | GSE360\_LOW\_DOSE\_B\_MALAYI\_VS\_M\_TUBERCULOSIS\_MAC\_DN |  | 16 | -0.25 | -1.04 | 0.386 | 0.650 | 1.000 | 227 | tags=31%, list=16%, signal=37% |
| 1475 | GSE37605\_C57BL6\_VS\_NOD\_FOXP3\_FUSION\_GFP\_TCONV\_UP |  | 15 | -0.25 | -1.04 | 0.378 | 0.650 | 1.000 | 614 | tags=73%, list=44%, signal=128% |
| 1476 | GSE22886\_CTRL\_VS\_LPS\_24H\_DC\_DN |  | 27 | -0.21 | -1.04 | 0.384 | 0.650 | 1.000 | 557 | tags=67%, list=39%, signal=108% |
| 1477 | GSE46606\_DAY1\_VS\_DAY3\_CD40L\_IL2\_IL5\_STIMULATED\_IRF4\_KO\_BCELL\_DN |  | 22 | -0.22 | -1.03 | 0.403 | 0.652 | 1.000 | 245 | tags=32%, list=17%, signal=38% |
| 1478 | GSE3982\_MEMORY\_CD4\_TCELL\_VS\_BCELL\_DN |  | 21 | -0.22 | -1.03 | 0.415 | 0.653 | 1.000 | 205 | tags=24%, list=15%, signal=27% |
| 1479 | GSE22432\_CDC\_VS\_COMMON\_DC\_PROGENITOR\_DN |  | 28 | -0.20 | -1.03 | 0.423 | 0.654 | 1.000 | 216 | tags=25%, list=15%, signal=29% |
| 1480 | GSE22886\_TH1\_VS\_TH2\_12H\_ACT\_DN |  | 15 | -0.25 | -1.03 | 0.400 | 0.654 | 1.000 | 350 | tags=40%, list=25%, signal=53% |
| 1481 | GSE17580\_UNINFECTED\_VS\_S\_MANSONI\_INF\_TEFF\_UP |  | 15 | -0.25 | -1.03 | 0.405 | 0.655 | 1.000 | 821 | tags=87%, list=58%, signal=205% |
| 1482 | GSE2706\_UNSTIM\_VS\_8H\_LPS\_DC\_UP |  | 21 | -0.22 | -1.03 | 0.404 | 0.655 | 1.000 | 326 | tags=38%, list=23%, signal=49% |
| 1483 | GSE17721\_CTRL\_VS\_POLYIC\_4H\_BMDC\_UP |  | 28 | -0.20 | -1.03 | 0.418 | 0.656 | 1.000 | 196 | tags=25%, list=14%, signal=28% |
| 1484 | GSE17974\_0H\_VS\_1H\_IN\_VITRO\_ACT\_CD4\_TCELL\_UP |  | 16 | -0.25 | -1.03 | 0.425 | 0.657 | 1.000 | 140 | tags=25%, list=10%, signal=27% |
| 1485 | GSE2770\_UNTREATED\_VS\_ACT\_CD4\_TCELL\_6H\_DN |  | 23 | -0.21 | -1.03 | 0.414 | 0.658 | 1.000 | 188 | tags=26%, list=13%, signal=30% |
| 1486 | GSE10239\_NAIVE\_VS\_DAY4.5\_EFF\_CD8\_TCELL\_UP |  | 22 | -0.22 | -1.03 | 0.392 | 0.657 | 1.000 | 117 | tags=18%, list=8%, signal=20% |
| 1487 | GSE7460\_FOXP3\_MUT\_VS\_HET\_ACT\_TCONV\_UP |  | 17 | -0.24 | -1.03 | 0.404 | 0.658 | 1.000 | 550 | tags=71%, list=39%, signal=114% |
| 1488 | GSE21774\_CD62L\_POS\_CD56\_DIM\_VS\_CD62L\_NEG\_CD56\_DIM\_NK\_CELL\_UP |  | 16 | -0.25 | -1.03 | 0.420 | 0.658 | 1.000 | 256 | tags=38%, list=18%, signal=45% |
| 1489 | GSE13547\_CTRL\_VS\_ANTI\_IGM\_STIM\_BCELL\_12H\_DN |  | 18 | -0.23 | -1.03 | 0.400 | 0.659 | 1.000 | 67 | tags=17%, list=5%, signal=17% |
| 1490 | GSE5503\_PLN\_DC\_VS\_SPLEEN\_DC\_ACTIVATED\_ALLOGENIC\_TCELL\_UP |  | 15 | -0.25 | -1.03 | 0.410 | 0.659 | 1.000 | 156 | tags=27%, list=11%, signal=30% |
| 1491 | GSE17186\_CD21LOW\_VS\_CD21HIGH\_TRANSITIONAL\_BCELL\_DN |  | 16 | -0.24 | -1.03 | 0.411 | 0.659 | 1.000 | 75 | tags=19%, list=5%, signal=20% |
| 1492 | GSE5589\_UNSTIM\_VS\_180MIN\_LPS\_AND\_IL10\_STIM\_MACROPHAGE\_DN |  | 15 | -0.25 | -1.03 | 0.421 | 0.659 | 1.000 | 269 | tags=33%, list=19%, signal=41% |
| 1493 | GSE3994\_WT\_VS\_PAC1\_KO\_ACTIVATED\_MAST\_CELL\_DN |  | 18 | -0.24 | -1.03 | 0.421 | 0.659 | 1.000 | 60 | tags=17%, list=4%, signal=17% |
| 1494 | GSE34156\_TLR1\_TLR2\_LIGAND\_VS\_NOD2\_AND\_TLR1\_TLR2\_LIGAND\_24H\_TREATED\_MONOCYTE\_DN |  | 16 | -0.24 | -1.02 | 0.399 | 0.663 | 1.000 | 97 | tags=25%, list=7%, signal=27% |
| 1495 | GSE17721\_12H\_VS\_24H\_POLYIC\_BMDC\_UP |  | 25 | -0.20 | -1.02 | 0.392 | 0.665 | 1.000 | 221 | tags=32%, list=16%, signal=37% |
| 1496 | GSE22886\_NAIVE\_CD8\_TCELL\_VS\_MONOCYTE\_DN |  | 16 | -0.24 | -1.02 | 0.422 | 0.664 | 1.000 | 276 | tags=31%, list=20%, signal=38% |
| 1497 | GSE9960\_HEALTHY\_VS\_GRAM\_NEG\_SEPSIS\_PBMC\_UP |  | 26 | -0.20 | -1.02 | 0.429 | 0.664 | 1.000 | 521 | tags=58%, list=37%, signal=90% |
| 1498 | GSE37533\_PPARG1\_FOXP3\_VS\_PPARG2\_FOXP3\_TRANSDUCED\_CD4\_TCELL\_PIOGLITAZONE\_TREATED\_UP |  | 34 | -0.18 | -1.02 | 0.446 | 0.666 | 1.000 | 596 | tags=65%, list=42%, signal=109% |
| 1499 | GSE9509\_LPS\_VS\_LPS\_AND\_IL10\_STIM\_IL10\_KO\_MACROPHAGE\_20MIN\_DN |  | 15 | -0.25 | -1.02 | 0.429 | 0.667 | 1.000 | 414 | tags=53%, list=29%, signal=75% |
| 1500 | GSE24142\_EARLY\_THYMIC\_PROGENITOR\_VS\_DN3\_THYMOCYTE\_FETAL\_UP |  | 22 | -0.21 | -1.02 | 0.409 | 0.667 | 1.000 | 558 | tags=64%, list=40%, signal=104% |
| 1501 | GSE22886\_NAIVE\_TCELL\_VS\_NEUTROPHIL\_DN |  | 21 | -0.22 | -1.02 | 0.414 | 0.666 | 1.000 | 403 | tags=48%, list=29%, signal=66% |
| 1502 | GSE5960\_TH1\_VS\_ANERGIC\_TH1\_DN |  | 15 | -0.25 | -1.02 | 0.416 | 0.666 | 1.000 | 162 | tags=27%, list=11%, signal=30% |
| 1503 | GSE19772\_CTRL\_VS\_HCMV\_INF\_MONOCYTES\_AND\_PI3K\_INHIBITION\_UP |  | 18 | -0.23 | -1.02 | 0.411 | 0.666 | 1.000 | 686 | tags=78%, list=49%, signal=149% |
| 1504 | GSE17974\_IL4\_AND\_ANTI\_IL12\_VS\_UNTREATED\_48H\_ACT\_CD4\_TCELL\_DN |  | 15 | -0.24 | -1.02 | 0.417 | 0.666 | 1.000 | 572 | tags=73%, list=41%, signal=122% |
| 1505 | GSE36476\_CTRL\_VS\_TSST\_ACT\_16H\_MEMORY\_CD4\_TCELL\_YOUNG\_DN |  | 26 | -0.20 | -1.02 | 0.436 | 0.666 | 1.000 | 1054 | tags=96%, list=75%, signal=373% |
| 1506 | GSE10239\_NAIVE\_VS\_KLRG1HIGH\_EFF\_CD8\_TCELL\_UP |  | 21 | -0.22 | -1.02 | 0.456 | 0.666 | 1.000 | 67 | tags=14%, list=5%, signal=15% |
| 1507 | GSE360\_DC\_VS\_MAC\_T\_GONDII\_UP |  | 18 | -0.24 | -1.02 | 0.439 | 0.666 | 1.000 | 497 | tags=61%, list=35%, signal=93% |
| 1508 | GSE37301\_LYMPHOID\_PRIMED\_MPP\_VS\_RAG2\_KO\_NK\_CELL\_UP |  | 26 | -0.20 | -1.02 | 0.413 | 0.667 | 1.000 | 838 | tags=88%, list=59%, signal=214% |
| 1509 | GSE24972\_WT\_VS\_IRF8\_KO\_MARGINAL\_ZONE\_SPLEEN\_BCELL\_DN |  | 15 | -0.25 | -1.02 | 0.409 | 0.667 | 1.000 | 922 | tags=93%, list=65%, signal=266% |
| 1510 | GSE22033\_UNTREATED\_VS\_MRL24\_TREATED\_MEF\_UP |  | 19 | -0.22 | -1.02 | 0.425 | 0.667 | 1.000 | 1107 | tags=100%, list=78%, signal=458% |
| 1511 | GSE17721\_LPS\_VS\_GARDIQUIMOD\_1H\_BMDC\_DN |  | 17 | -0.23 | -1.02 | 0.403 | 0.668 | 1.000 | 1089 | tags=100%, list=77%, signal=433% |
| 1512 | GSE360\_CTRL\_VS\_L\_MAJOR\_MAC\_DN |  | 17 | -0.23 | -1.02 | 0.424 | 0.670 | 1.000 | 273 | tags=35%, list=19%, signal=43% |
| 1513 | GSE22601\_CD4\_SINGLE\_POSITIVE\_VS\_CD8\_SINGLE\_POSITIVE\_THYMOCYTE\_UP |  | 17 | -0.23 | -1.02 | 0.421 | 0.671 | 1.000 | 157 | tags=24%, list=11%, signal=26% |
| 1514 | GSE3982\_MAC\_VS\_BASOPHIL\_UP |  | 17 | -0.24 | -1.02 | 0.428 | 0.672 | 1.000 | 645 | tags=76%, list=46%, signal=139% |
| 1515 | GSE13547\_CTRL\_VS\_ANTI\_IGM\_STIM\_BCELL\_2H\_UP |  | 20 | -0.22 | -1.02 | 0.423 | 0.672 | 1.000 | 315 | tags=40%, list=22%, signal=51% |
| 1516 | GSE9006\_HEALTHY\_VS\_TYPE\_1\_DIABETES\_PBMC\_AT\_DX\_DN |  | 27 | -0.20 | -1.01 | 0.425 | 0.673 | 1.000 | 216 | tags=26%, list=15%, signal=30% |
| 1517 | GSE10325\_LUPUS\_CD4\_TCELL\_VS\_LUPUS\_MYELOID\_DN |  | 21 | -0.22 | -1.01 | 0.438 | 0.675 | 1.000 | 550 | tags=62%, list=39%, signal=100% |
| 1518 | GSE369\_PRE\_VS\_POST\_IL6\_INJECTION\_IFNG\_KO\_LIVER\_DN |  | 20 | -0.22 | -1.01 | 0.434 | 0.675 | 1.000 | 155 | tags=25%, list=11%, signal=28% |
| 1519 | GSE10147\_IL3\_VS\_IL3\_AND\_CPG\_STIM\_PDC\_DN |  | 19 | -0.22 | -1.01 | 0.424 | 0.674 | 1.000 | 199 | tags=26%, list=14%, signal=30% |
| 1520 | GSE3982\_MAC\_VS\_NKCELL\_UP |  | 19 | -0.23 | -1.01 | 0.435 | 0.674 | 1.000 | 469 | tags=58%, list=33%, signal=86% |
| 1521 | GSE21063\_CTRL\_VS\_ANTI\_IGM\_STIM\_BCELL\_3H\_DN |  | 17 | -0.24 | -1.01 | 0.449 | 0.674 | 1.000 | 352 | tags=41%, list=25%, signal=54% |
| 1522 | GSE20754\_WT\_VS\_TCF1\_KO\_MEMORY\_CD8\_TCELL\_UP |  | 28 | -0.19 | -1.01 | 0.446 | 0.675 | 1.000 | 557 | tags=64%, list=39%, signal=104% |
| 1523 | GSE35825\_IFNA\_VS\_IFNG\_STIM\_MACROPHAGE\_UP |  | 28 | -0.20 | -1.01 | 0.422 | 0.675 | 1.000 | 140 | tags=21%, list=10%, signal=23% |
| 1524 | GSE37301\_MULTIPOTENT\_PROGENITOR\_VS\_COMMON\_LYMPHOID\_PROGENITOR\_UP |  | 17 | -0.23 | -1.01 | 0.431 | 0.676 | 1.000 | 949 | tags=94%, list=67%, signal=284% |
| 1525 | GSE3982\_MAC\_VS\_BCELL\_UP |  | 20 | -0.22 | -1.01 | 0.432 | 0.676 | 1.000 | 533 | tags=65%, list=38%, signal=103% |
| 1526 | GSE17721\_POLYIC\_VS\_GARDIQUIMOD\_8H\_BMDC\_DN |  | 23 | -0.21 | -1.01 | 0.428 | 0.676 | 1.000 | 980 | tags=96%, list=69%, signal=308% |
| 1527 | GSE27786\_LSK\_VS\_CD4\_TCELL\_DN |  | 15 | -0.24 | -1.01 | 0.429 | 0.676 | 1.000 | 380 | tags=40%, list=27%, signal=54% |
| 1528 | GSE24634\_NAIVE\_CD4\_TCELL\_VS\_DAY3\_IL4\_CONV\_TREG\_UP |  | 16 | -0.24 | -1.01 | 0.421 | 0.678 | 1.000 | 252 | tags=38%, list=18%, signal=45% |
| 1529 | GSE37416\_CTRL\_VS\_12H\_F\_TULARENSIS\_LVS\_NEUTROPHIL\_DN |  | 21 | -0.22 | -1.01 | 0.431 | 0.677 | 1.000 | 319 | tags=38%, list=23%, signal=48% |
| 1530 | GSE20366\_EX\_VIVO\_VS\_HOMEOSTATIC\_CONVERSION\_TREG\_DN |  | 17 | -0.24 | -1.01 | 0.469 | 0.678 | 1.000 | 105 | tags=24%, list=7%, signal=25% |
| 1531 | GSE37605\_TREG\_VS\_TCONV\_C57BL6\_FOXP3\_FUSION\_GFP\_DN |  | 18 | -0.24 | -1.01 | 0.427 | 0.678 | 1.000 | 841 | tags=89%, list=60%, signal=217% |
| 1532 | GSE5455\_HEALTHY\_VS\_TUMOR\_BEARING\_MOUSE\_SPLEEN\_MONOCYTE\_DN |  | 22 | -0.21 | -1.01 | 0.423 | 0.679 | 1.000 | 455 | tags=55%, list=32%, signal=79% |
| 1533 | GSE15735\_2H\_VS\_12H\_HDAC\_INHIBITOR\_TREATED\_CD4\_TCELL\_UP |  | 18 | -0.23 | -1.01 | 0.436 | 0.679 | 1.000 | 532 | tags=67%, list=38%, signal=106% |
| 1534 | GSE36078\_UNTREATED\_VS\_AD5\_INF\_IL1R\_KO\_MOUSE\_LUNG\_DC\_UP |  | 24 | -0.21 | -1.01 | 0.446 | 0.682 | 1.000 | 276 | tags=29%, list=20%, signal=36% |
| 1535 | GSE26495\_NAIVE\_VS\_PD1HIGH\_CD8\_TCELL\_UP |  | 20 | -0.22 | -1.01 | 0.455 | 0.683 | 1.000 | 476 | tags=60%, list=34%, signal=89% |
| 1536 | GSE4748\_CTRL\_VS\_CYANOBACTERIUM\_LPSLIKE\_STIM\_DC\_1H\_DN |  | 18 | -0.23 | -1.01 | 0.423 | 0.683 | 1.000 | 320 | tags=39%, list=23%, signal=50% |
| 1537 | GSE14908\_RESTING\_VS\_HDM\_STIM\_CD4\_TCELL\_NONATOPIC\_PATIENT\_DN |  | 17 | -0.23 | -1.00 | 0.425 | 0.684 | 1.000 | 76 | tags=18%, list=5%, signal=18% |
| 1538 | GSE29618\_PRE\_VS\_DAY7\_FLU\_VACCINE\_MDC\_UP |  | 22 | -0.21 | -1.00 | 0.437 | 0.684 | 1.000 | 309 | tags=36%, list=22%, signal=46% |
| 1539 | GSE30971\_WBP7\_HET\_VS\_KO\_MACROPHAGE\_4H\_LPS\_STIM\_DN |  | 21 | -0.22 | -1.00 | 0.440 | 0.684 | 1.000 | 379 | tags=48%, list=27%, signal=64% |
| 1540 | GSE33513\_TCF7\_KO\_VS\_HET\_EARLY\_THYMIC\_PROGENITOR\_UP |  | 17 | -0.23 | -1.00 | 0.433 | 0.683 | 1.000 | 363 | tags=47%, list=26%, signal=63% |
| 1541 | GSE5679\_PPARG\_LIGAND\_ROSIGLITAZONE\_VS\_RARA\_AGONIST\_AM580\_TREATED\_DC\_UP |  | 16 | -0.23 | -1.00 | 0.433 | 0.684 | 1.000 | 1087 | tags=100%, list=77%, signal=431% |
| 1542 | GSE23114\_WT\_VS\_SLE2C1\_MOUSE\_SPLEEN\_B1A\_BCELL\_UP |  | 20 | -0.22 | -1.00 | 0.454 | 0.684 | 1.000 | 670 | tags=70%, list=47%, signal=131% |
| 1543 | GSE3337\_CTRL\_VS\_4H\_IFNG\_IN\_CD8POS\_DC\_UP |  | 18 | -0.23 | -1.00 | 0.433 | 0.685 | 1.000 | 543 | tags=61%, list=38%, signal=98% |
| 1544 | GSE3982\_MAC\_VS\_CENT\_MEMORY\_CD4\_TCELL\_UP |  | 16 | -0.24 | -1.00 | 0.424 | 0.684 | 1.000 | 1083 | tags=100%, list=77%, signal=425% |
| 1545 | GSE17721\_CTRL\_VS\_GARDIQUIMOD\_12H\_BMDC\_DN |  | 17 | -0.23 | -1.00 | 0.426 | 0.685 | 1.000 | 180 | tags=29%, list=13%, signal=33% |
| 1546 | GSE22589\_HEALTHY\_VS\_HIV\_AND\_SIV\_INFECTED\_DC\_UP |  | 21 | -0.21 | -1.00 | 0.463 | 0.684 | 1.000 | 124 | tags=19%, list=9%, signal=21% |
| 1547 | GSE41176\_UNSTIM\_VS\_ANTI\_IGM\_STIM\_TAK1\_KO\_BCELL\_1H\_DN |  | 15 | -0.25 | -1.00 | 0.466 | 0.686 | 1.000 | 891 | tags=93%, list=63%, signal=251% |
| 1548 | GSE27859\_DC\_VS\_CD11C\_INT\_F480\_HI\_MACROPHAGE\_UP |  | 23 | -0.21 | -1.00 | 0.486 | 0.686 | 1.000 | 1127 | tags=100%, list=80%, signal=489% |
| 1549 | GSE27786\_CD4\_TCELL\_VS\_NKCELL\_DN |  | 20 | -0.22 | -1.00 | 0.443 | 0.685 | 1.000 | 88 | tags=15%, list=6%, signal=16% |
| 1550 | GSE40277\_EOS\_AND\_LEF1\_TRANSDUCED\_VS\_GATA1\_AND\_SATB1\_TRANSDUCED\_CD4\_TCELL\_UP |  | 23 | -0.21 | -1.00 | 0.442 | 0.686 | 1.000 | 447 | tags=52%, list=32%, signal=75% |
| 1551 | GSE1432\_CTRL\_VS\_IFNG\_24H\_MICROGLIA\_DN |  | 22 | -0.22 | -1.00 | 0.454 | 0.688 | 1.000 | 527 | tags=64%, list=37%, signal=100% |
| 1552 | GSE41867\_DAY6\_VS\_DAY8\_LCMV\_CLONE13\_EFFECTOR\_CD8\_TCELL\_DN |  | 28 | -0.19 | -1.00 | 0.453 | 0.688 | 1.000 | 1013 | tags=93%, list=72%, signal=323% |
| 1553 | GSE21379\_TFH\_VS\_NON\_TFH\_CD4\_TCELL\_DN |  | 15 | -0.25 | -1.00 | 0.470 | 0.688 | 1.000 | 422 | tags=53%, list=30%, signal=75% |
| 1554 | GSE15330\_HSC\_VS\_MEGAKARYOCYTE\_ERYTHROID\_PROGENITOR\_UP |  | 16 | -0.23 | -1.00 | 0.448 | 0.688 | 1.000 | 259 | tags=38%, list=18%, signal=45% |
| 1555 | GSE26669\_CD4\_VS\_CD8\_TCELL\_IN\_MLR\_COSTIM\_BLOCK\_DN |  | 22 | -0.21 | -1.00 | 0.438 | 0.688 | 1.000 | 182 | tags=27%, list=13%, signal=31% |
| 1556 | GSE14308\_TH2\_VS\_NAIVE\_CD4\_TCELL\_UP |  | 26 | -0.19 | -1.00 | 0.456 | 0.688 | 1.000 | 361 | tags=38%, list=26%, signal=51% |
| 1557 | GSE6674\_CPG\_VS\_PL2\_3\_STIM\_BCELL\_DN |  | 27 | -0.19 | -1.00 | 0.454 | 0.688 | 1.000 | 697 | tags=74%, list=49%, signal=144% |
| 1558 | GSE16522\_MEMORY\_VS\_NAIVE\_ANTI\_CD3CD28\_STIM\_CD8\_TCELL\_DN |  | 18 | -0.23 | -1.00 | 0.455 | 0.688 | 1.000 | 315 | tags=39%, list=22%, signal=49% |
| 1559 | GSE14386\_UNTREATED\_VS\_IFNA\_TREATED\_ACT\_PBMC\_MS\_PATIENT\_DN |  | 26 | -0.20 | -1.00 | 0.445 | 0.689 | 1.000 | 456 | tags=46%, list=32%, signal=67% |
| 1560 | GSE30971\_WBP7\_HET\_VS\_KO\_MACROPHAGE\_2H\_LPS\_STIM\_UP |  | 19 | -0.22 | -1.00 | 0.458 | 0.690 | 1.000 | 563 | tags=63%, list=40%, signal=104% |
| 1561 | GSE41867\_DAY6\_VS\_DAY8\_LCMV\_ARMSTRONG\_EFFECTOR\_CD8\_TCELL\_UP |  | 15 | -0.24 | -1.00 | 0.442 | 0.690 | 1.000 | 66 | tags=20%, list=5%, signal=21% |
| 1562 | GSE5589\_LPS\_AND\_IL10\_VS\_LPS\_AND\_IL6\_STIM\_IL6\_KO\_MACROPHAGE\_45MIN\_DN |  | 23 | -0.21 | -1.00 | 0.442 | 0.690 | 1.000 | 448 | tags=52%, list=32%, signal=75% |
| 1563 | GSE25087\_TREG\_VS\_TCONV\_ADULT\_DN |  | 17 | -0.23 | -1.00 | 0.437 | 0.690 | 1.000 | 173 | tags=29%, list=12%, signal=33% |
| 1564 | GSE17721\_0.5H\_VS\_12H\_GARDIQUIMOD\_BMDC\_UP |  | 16 | -0.24 | -0.99 | 0.455 | 0.691 | 1.000 | 31 | tags=13%, list=2%, signal=13% |
| 1565 | GSE41867\_NAIVE\_VS\_DAY15\_LCMV\_ARMSTRONG\_EFFECTOR\_CD8\_TCELL\_DN |  | 20 | -0.21 | -0.99 | 0.437 | 0.693 | 1.000 | 827 | tags=85%, list=59%, signal=202% |
| 1566 | GSE42088\_UNINF\_VS\_LEISHMANIA\_INF\_DC\_24H\_DN |  | 24 | -0.20 | -0.99 | 0.474 | 0.694 | 1.000 | 215 | tags=29%, list=15%, signal=34% |
| 1567 | GSE22229\_RENAL\_TRANSPLANT\_VS\_HEALTHY\_PBMC\_UP |  | 19 | -0.23 | -0.99 | 0.456 | 0.694 | 1.000 | 353 | tags=47%, list=25%, signal=62% |
| 1568 | GSE29615\_CTRL\_VS\_LAIV\_FLU\_VACCINE\_PBMC\_UP |  | 16 | -0.23 | -0.99 | 0.463 | 0.694 | 1.000 | 313 | tags=44%, list=22%, signal=56% |
| 1569 | GSE15930\_NAIVE\_VS\_72H\_IN\_VITRO\_STIM\_IFNAB\_CD8\_TCELL\_DN |  | 24 | -0.20 | -0.99 | 0.474 | 0.698 | 1.000 | 766 | tags=83%, list=54%, signal=179% |
| 1570 | GSE15659\_CD45RA\_NEG\_CD4\_TCELL\_VS\_RESTING\_TREG\_UP |  | 18 | -0.23 | -0.99 | 0.460 | 0.697 | 1.000 | 238 | tags=33%, list=17%, signal=40% |
| 1571 | GSE13738\_RESTING\_VS\_TCR\_ACTIVATED\_CD4\_TCELL\_DN |  | 19 | -0.23 | -0.99 | 0.467 | 0.697 | 1.000 | 150 | tags=21%, list=11%, signal=23% |
| 1572 | GSE6674\_PL2\_3\_VS\_ANTI\_IGM\_AND\_CPG\_STIM\_BCELL\_DN |  | 23 | -0.21 | -0.99 | 0.456 | 0.697 | 1.000 | 96 | tags=17%, list=7%, signal=18% |
| 1573 | GSE4984\_GALECTIN1\_VS\_VEHICLE\_CTRL\_TREATED\_DC\_UP |  | 16 | -0.24 | -0.99 | 0.465 | 0.697 | 1.000 | 68 | tags=19%, list=5%, signal=19% |
| 1574 | GSE26928\_NAIVE\_VS\_EFF\_MEMORY\_CD4\_TCELL\_DN |  | 17 | -0.23 | -0.99 | 0.457 | 0.696 | 1.000 | 70 | tags=18%, list=5%, signal=18% |
| 1575 | GSE43955\_TH0\_VS\_TGFB\_IL6\_TH17\_ACT\_CD4\_TCELL\_4H\_DN |  | 17 | -0.22 | -0.99 | 0.474 | 0.697 | 1.000 | 482 | tags=59%, list=34%, signal=88% |
| 1576 | GSE24972\_MARGINAL\_ZONE\_BCELL\_VS\_FOLLICULAR\_BCELL\_DN |  | 15 | -0.25 | -0.99 | 0.424 | 0.697 | 1.000 | 339 | tags=47%, list=24%, signal=61% |
| 1577 | GSE11864\_UNTREATED\_VS\_CSF1\_IN\_MAC\_UP |  | 18 | -0.22 | -0.99 | 0.451 | 0.697 | 1.000 | 539 | tags=61%, list=38%, signal=98% |
| 1578 | GSE13522\_CTRL\_VS\_T\_CRUZI\_Y\_STRAIN\_INF\_SKIN\_IFNAR\_KO\_UP |  | 26 | -0.20 | -0.99 | 0.479 | 0.697 | 1.000 | 472 | tags=54%, list=33%, signal=79% |
| 1579 | GSE40655\_FOXO1\_KO\_VS\_WT\_NTREG\_UP |  | 22 | -0.21 | -0.99 | 0.483 | 0.698 | 1.000 | 196 | tags=27%, list=14%, signal=31% |
| 1580 | GSE360\_T\_GONDII\_VS\_B\_MALAYI\_HIGH\_DOSE\_DC\_DN |  | 24 | -0.20 | -0.99 | 0.482 | 0.697 | 1.000 | 326 | tags=38%, list=23%, signal=48% |
| 1581 | GSE24142\_ADULT\_VS\_FETAL\_DN2\_THYMOCYTE\_DN |  | 20 | -0.22 | -0.99 | 0.441 | 0.697 | 1.000 | 496 | tags=55%, list=35%, signal=84% |
| 1582 | GSE43863\_TH1\_VS\_LY6C\_LOW\_CXCR5NEG\_EFFECTOR\_CD4\_TCELL\_DN |  | 23 | -0.21 | -0.99 | 0.470 | 0.699 | 1.000 | 464 | tags=52%, list=33%, signal=76% |
| 1583 | GSE42724\_MEMORY\_VS\_B1\_BCELL\_UP |  | 20 | -0.22 | -0.99 | 0.466 | 0.699 | 1.000 | 147 | tags=20%, list=10%, signal=22% |
| 1584 | GSE360\_DC\_VS\_MAC\_T\_GONDII\_DN |  | 15 | -0.24 | -0.99 | 0.443 | 0.699 | 1.000 | 389 | tags=47%, list=28%, signal=64% |
| 1585 | GSE5589\_LPS\_AND\_IL10\_VS\_LPS\_AND\_IL6\_STIM\_MACROPHAGE\_45MIN\_DN |  | 23 | -0.20 | -0.99 | 0.474 | 0.700 | 1.000 | 266 | tags=30%, list=19%, signal=37% |
| 1586 | GSE14308\_INDUCED\_VS\_NATURAL\_TREG\_DN |  | 24 | -0.20 | -0.99 | 0.496 | 0.700 | 1.000 | 374 | tags=42%, list=27%, signal=56% |
| 1587 | KAECH\_DAY8\_EFF\_VS\_DAY15\_EFF\_CD8\_TCELL\_DN |  | 22 | -0.21 | -0.99 | 0.439 | 0.699 | 1.000 | 534 | tags=59%, list=38%, signal=94% |
| 1588 | GSE17721\_0.5H\_VS\_12H\_GARDIQUIMOD\_BMDC\_DN |  | 21 | -0.21 | -0.99 | 0.450 | 0.699 | 1.000 | 59 | tags=14%, list=4%, signal=15% |
| 1589 | GSE14308\_TH2\_VS\_NATURAL\_TREG\_DN |  | 18 | -0.22 | -0.98 | 0.451 | 0.700 | 1.000 | 769 | tags=83%, list=55%, signal=181% |
| 1590 | GSE15330\_LYMPHOID\_MULTIPOTENT\_VS\_MEGAKARYOCYTE\_ERYTHROID\_PROGENITOR\_DN |  | 23 | -0.20 | -0.98 | 0.461 | 0.700 | 1.000 | 309 | tags=35%, list=22%, signal=44% |
| 1591 | GSE17721\_0.5H\_VS\_12H\_POLYIC\_BMDC\_UP |  | 21 | -0.21 | -0.98 | 0.459 | 0.701 | 1.000 | 1120 | tags=100%, list=79%, signal=478% |
| 1592 | GSE14308\_TH1\_VS\_NATURAL\_TREG\_UP |  | 23 | -0.20 | -0.98 | 0.477 | 0.702 | 1.000 | 1035 | tags=96%, list=73%, signal=353% |
| 1593 | GSE360\_DC\_VS\_MAC\_UP |  | 22 | -0.21 | -0.98 | 0.466 | 0.702 | 1.000 | 1029 | tags=95%, list=73%, signal=347% |
| 1594 | GSE17721\_LPS\_VS\_GARDIQUIMOD\_2H\_BMDC\_DN |  | 16 | -0.23 | -0.98 | 0.441 | 0.703 | 1.000 | 980 | tags=94%, list=69%, signal=303% |
| 1595 | GSE17721\_0.5H\_VS\_12H\_POLYIC\_BMDC\_DN |  | 18 | -0.22 | -0.98 | 0.470 | 0.704 | 1.000 | 225 | tags=33%, list=16%, signal=39% |
| 1596 | GSE28237\_FOLLICULAR\_VS\_LATE\_GC\_BCELL\_DN |  | 19 | -0.23 | -0.98 | 0.481 | 0.703 | 1.000 | 1098 | tags=100%, list=78%, signal=445% |
| 1597 | GSE19888\_CTRL\_VS\_T\_CELL\_MEMBRANES\_ACT\_MAST\_CELL\_UP |  | 22 | -0.21 | -0.98 | 0.469 | 0.703 | 1.000 | 158 | tags=18%, list=11%, signal=20% |
| 1598 | GSE29949\_MICROGLIA\_BRAIN\_VS\_CD8\_POS\_DC\_SPLEEN\_UP |  | 21 | -0.21 | -0.98 | 0.501 | 0.703 | 1.000 | 84 | tags=14%, list=6%, signal=15% |
| 1599 | GSE10211\_UV\_INACT\_SENDAI\_VS\_LIVE\_SENDAI\_VIRUS\_TRACHEAL\_EPITHELIAL\_CELLS\_DN |  | 15 | -0.24 | -0.98 | 0.484 | 0.703 | 1.000 | 252 | tags=33%, list=18%, signal=40% |
| 1600 | GSE8685\_IL2\_STARVED\_VS\_IL21\_ACT\_IL2\_STARVED\_CD4\_TCELL\_UP |  | 24 | -0.20 | -0.98 | 0.487 | 0.709 | 1.000 | 249 | tags=29%, list=18%, signal=35% |
| 1601 | GSE21927\_SPLEEN\_C57BL6\_VS\_4T1\_TUMOR\_BALBC\_MONOCYTES\_DN |  | 26 | -0.19 | -0.98 | 0.493 | 0.708 | 1.000 | 828 | tags=85%, list=59%, signal=201% |
| 1602 | GSE2770\_TGFB\_AND\_IL4\_VS\_IL4\_TREATED\_ACT\_CD4\_TCELL\_48H\_DN |  | 22 | -0.20 | -0.98 | 0.458 | 0.708 | 1.000 | 500 | tags=55%, list=35%, signal=83% |
| 1603 | GSE3039\_CD4\_TCELL\_VS\_B1\_BCELL\_DN |  | 18 | -0.22 | -0.98 | 0.456 | 0.708 | 1.000 | 566 | tags=67%, list=40%, signal=110% |
| 1604 | GSE2770\_TGFB\_AND\_IL4\_ACT\_VS\_ACT\_CD4\_TCELL\_48H\_DN |  | 20 | -0.21 | -0.98 | 0.485 | 0.708 | 1.000 | 500 | tags=60%, list=35%, signal=92% |
| 1605 | GSE15271\_CXCR4\_POS\_VS\_NEG\_GC\_BCELL\_UP |  | 28 | -0.18 | -0.98 | 0.494 | 0.708 | 1.000 | 1023 | tags=93%, list=73%, signal=331% |
| 1606 | GSE37301\_HEMATOPOIETIC\_STEM\_CELL\_VS\_COMMON\_LYMPHOID\_PROGENITOR\_UP |  | 18 | -0.22 | -0.97 | 0.498 | 0.711 | 1.000 | 190 | tags=28%, list=13%, signal=32% |
| 1607 | GSE13229\_MATURE\_VS\_INTMATURE\_NKCELL\_DN |  | 17 | -0.23 | -0.97 | 0.468 | 0.711 | 1.000 | 1098 | tags=100%, list=78%, signal=445% |
| 1608 | GSE5679\_RARA\_AGONIST\_AM580\_VS\_AM580\_AND\_ROSIGLITAZONE\_TREATED\_DC\_DN |  | 18 | -0.23 | -0.97 | 0.485 | 0.713 | 1.000 | 217 | tags=28%, list=15%, signal=32% |
| 1609 | GSE29164\_DAY3\_VS\_DAY7\_UNTREATED\_MELANOMA\_DN |  | 19 | -0.22 | -0.97 | 0.499 | 0.715 | 1.000 | 391 | tags=47%, list=28%, signal=65% |
| 1610 | GSE3982\_NKCELL\_VS\_TH1\_UP |  | 15 | -0.23 | -0.97 | 0.479 | 0.716 | 1.000 | 104 | tags=20%, list=7%, signal=21% |
| 1611 | GSE17721\_POLYIC\_VS\_CPG\_1H\_BMDC\_DN |  | 20 | -0.21 | -0.97 | 0.458 | 0.718 | 1.000 | 513 | tags=60%, list=36%, signal=93% |
| 1612 | GSE22886\_NAIVE\_TCELL\_VS\_DC\_UP |  | 19 | -0.22 | -0.97 | 0.457 | 0.718 | 1.000 | 315 | tags=37%, list=22%, signal=47% |
| 1613 | GSE3337\_CTRL\_VS\_4H\_IFNG\_IN\_CD8POS\_DC\_DN |  | 20 | -0.21 | -0.97 | 0.466 | 0.717 | 1.000 | 512 | tags=60%, list=36%, signal=93% |
| 1614 | GSE24634\_TEFF\_VS\_TCONV\_DAY10\_IN\_CULTURE\_UP |  | 18 | -0.22 | -0.97 | 0.444 | 0.717 | 1.000 | 157 | tags=22%, list=11%, signal=25% |
| 1615 | GSE22886\_NAIVE\_CD4\_TCELL\_VS\_48H\_ACT\_TH2\_DN |  | 23 | -0.19 | -0.97 | 0.486 | 0.717 | 1.000 | 82 | tags=13%, list=6%, signal=14% |
| 1616 | GSE14415\_INDUCED\_TREG\_VS\_FOXP3\_KO\_INDUCED\_TREG\_IL2\_CULTURE\_UP |  | 17 | -0.23 | -0.97 | 0.468 | 0.717 | 1.000 | 532 | tags=65%, list=38%, signal=103% |
| 1617 | GSE23321\_CD8\_STEM\_CELL\_MEMORY\_VS\_EFFECTOR\_MEMORY\_CD8\_TCELL\_UP |  | 16 | -0.23 | -0.97 | 0.446 | 0.717 | 1.000 | 1095 | tags=100%, list=78%, signal=441% |
| 1618 | GSE19923\_WT\_VS\_E2A\_KO\_DP\_THYMOCYTE\_UP |  | 20 | -0.21 | -0.97 | 0.487 | 0.717 | 1.000 | 1118 | tags=100%, list=79%, signal=475% |
| 1619 | GSE37416\_12H\_VS\_48H\_F\_TULARENSIS\_LVS\_NEUTROPHIL\_UP |  | 24 | -0.19 | -0.97 | 0.472 | 0.717 | 1.000 | 398 | tags=42%, list=28%, signal=57% |
| 1620 | GSE5589\_UNSTIM\_VS\_45MIN\_LPS\_AND\_IL6\_STIM\_MACROPHAGE\_UP |  | 15 | -0.24 | -0.97 | 0.481 | 0.717 | 1.000 | 939 | tags=93%, list=67%, signal=276% |
| 1621 | GSE360\_DC\_VS\_MAC\_L\_DONOVANI\_DN |  | 23 | -0.21 | -0.97 | 0.486 | 0.717 | 1.000 | 322 | tags=35%, list=23%, signal=44% |
| 1622 | GSE16451\_CTRL\_VS\_WEST\_EQUINE\_ENC\_VIRUS\_IMMATURE\_NEURON\_CELL\_LINE\_DN |  | 26 | -0.19 | -0.97 | 0.505 | 0.717 | 1.000 | 391 | tags=42%, list=28%, signal=57% |
| 1623 | GSE17721\_PAM3CSK4\_VS\_GADIQUIMOD\_16H\_BMDC\_UP |  | 19 | -0.22 | -0.97 | 0.495 | 0.719 | 1.000 | 379 | tags=47%, list=27%, signal=64% |
| 1624 | GSE32986\_CURDLAN\_LOWDOSE\_VS\_GMCSF\_AND\_CURDLAN\_LOWDOSE\_STIM\_DC\_DN |  | 36 | -0.17 | -0.97 | 0.483 | 0.720 | 1.000 | 224 | tags=22%, list=16%, signal=26% |
| 1625 | GSE21033\_1H\_VS\_12H\_POLYIC\_STIM\_DC\_UP |  | 18 | -0.22 | -0.97 | 0.508 | 0.720 | 1.000 | 195 | tags=28%, list=14%, signal=32% |
| 1626 | GSE22025\_UNTREATED\_VS\_TGFB1\_TREATED\_CD4\_TCELL\_DN |  | 18 | -0.23 | -0.97 | 0.494 | 0.720 | 1.000 | 465 | tags=50%, list=33%, signal=74% |
| 1627 | GSE24634\_NAIVE\_CD4\_TCELL\_VS\_DAY5\_IL4\_CONV\_TREG\_UP |  | 20 | -0.21 | -0.97 | 0.510 | 0.720 | 1.000 | 351 | tags=45%, list=25%, signal=59% |
| 1628 | GSE39110\_DAY3\_VS\_DAY6\_POST\_IMMUNIZATION\_CD8\_TCELL\_UP |  | 27 | -0.19 | -0.96 | 0.503 | 0.720 | 1.000 | 538 | tags=59%, list=38%, signal=94% |
| 1629 | GSE17721\_0.5H\_VS\_12H\_CPG\_BMDC\_UP |  | 17 | -0.22 | -0.96 | 0.482 | 0.720 | 1.000 | 307 | tags=35%, list=22%, signal=45% |
| 1630 | GSE1460\_INTRATHYMIC\_T\_PROGENITOR\_VS\_DP\_THYMOCYTE\_UP |  | 23 | -0.20 | -0.96 | 0.511 | 0.721 | 1.000 | 30 | tags=13%, list=2%, signal=13% |
| 1631 | GSE21670\_UNTREATED\_VS\_IL6\_TREATED\_CD4\_TCELL\_DN |  | 19 | -0.22 | -0.96 | 0.497 | 0.722 | 1.000 | 712 | tags=79%, list=50%, signal=157% |
| 1632 | GSE5589\_WT\_VS\_IL6\_KO\_LPS\_AND\_IL10\_STIM\_MACROPHAGE\_45MIN\_DN |  | 24 | -0.20 | -0.96 | 0.482 | 0.722 | 1.000 | 318 | tags=38%, list=23%, signal=48% |
| 1633 | GSE43955\_1H\_VS\_10H\_ACT\_CD4\_TCELL\_UP |  | 23 | -0.20 | -0.96 | 0.497 | 0.721 | 1.000 | 227 | tags=30%, list=16%, signal=36% |
| 1634 | GSE21670\_STAT3\_KO\_VS\_WT\_CD4\_TCELL\_IL6\_TREATED\_UP |  | 15 | -0.23 | -0.96 | 0.500 | 0.722 | 1.000 | 1085 | tags=100%, list=77%, signal=428% |
| 1635 | GSE33425\_CD161\_INT\_VS\_NEG\_CD8\_TCELL\_UP |  | 21 | -0.21 | -0.96 | 0.510 | 0.722 | 1.000 | 385 | tags=38%, list=27%, signal=52% |
| 1636 | GSE22886\_TCELL\_VS\_BCELL\_NAIVE\_DN |  | 17 | -0.22 | -0.96 | 0.481 | 0.722 | 1.000 | 323 | tags=41%, list=23%, signal=53% |
| 1637 | GSE11924\_TFH\_VS\_TH2\_CD4\_TCELL\_DN |  | 15 | -0.23 | -0.96 | 0.489 | 0.722 | 1.000 | 955 | tags=93%, list=68%, signal=286% |
| 1638 | GSE16385\_IFNG\_TNF\_VS\_IL4\_STIM\_MACROPHAGE\_ROSIGLITAZONE\_TREATED\_UP |  | 16 | -0.23 | -0.96 | 0.482 | 0.723 | 1.000 | 472 | tags=56%, list=33%, signal=84% |
| 1639 | GSE9006\_TYPE\_1\_VS\_TYPE\_2\_DIABETES\_PBMC\_AT\_DX\_DN |  | 27 | -0.19 | -0.96 | 0.515 | 0.723 | 1.000 | 104 | tags=19%, list=7%, signal=20% |
| 1640 | GSE10240\_CTRL\_VS\_IL17\_STIM\_PRIMARY\_BRONCHIAL\_EPITHELIAL\_CELLS\_DN |  | 19 | -0.22 | -0.96 | 0.489 | 0.723 | 1.000 | 532 | tags=63%, list=38%, signal=100% |
| 1641 | GSE35435\_RESTING\_VS\_IL4\_TREATED\_MACROPHAGE\_UP |  | 31 | -0.18 | -0.96 | 0.499 | 0.723 | 1.000 | 188 | tags=23%, list=13%, signal=25% |
| 1642 | GSE22886\_CD8\_TCELL\_VS\_BCELL\_NAIVE\_UP |  | 17 | -0.22 | -0.96 | 0.472 | 0.724 | 1.000 | 119 | tags=18%, list=8%, signal=19% |
| 1643 | GSE17721\_CTRL\_VS\_LPS\_6H\_BMDC\_DN |  | 16 | -0.24 | -0.96 | 0.505 | 0.724 | 1.000 | 434 | tags=56%, list=31%, signal=80% |
| 1644 | GSE3982\_MAC\_VS\_BCELL\_DN |  | 26 | -0.20 | -0.96 | 0.538 | 0.724 | 1.000 | 188 | tags=27%, list=13%, signal=30% |
| 1645 | GSE10240\_CTRL\_VS\_IL17\_AND\_IL22\_STIM\_PRIMARY\_BRONCHIAL\_EPITHELIAL\_CELLS\_DN |  | 17 | -0.22 | -0.96 | 0.483 | 0.724 | 1.000 | 264 | tags=35%, list=19%, signal=43% |
| 1646 | GSE26030\_UNSTIM\_VS\_RESTIM\_TH17\_DAY15\_POST\_POLARIZATION\_DN |  | 21 | -0.21 | -0.96 | 0.513 | 0.724 | 1.000 | 919 | tags=90%, list=65%, signal=256% |
| 1647 | GSE20727\_CTRL\_VS\_DNFB\_ALLERGEN\_TREATED\_DC\_DN |  | 17 | -0.22 | -0.96 | 0.496 | 0.724 | 1.000 | 128 | tags=24%, list=9%, signal=26% |
| 1648 | GSE25123\_IL4\_VS\_IL4\_AND\_ROSIGLITAZONE\_STIM\_PPARG\_KO\_MACROPHAGE\_DAY10\_DN |  | 28 | -0.19 | -0.96 | 0.511 | 0.724 | 1.000 | 476 | tags=50%, list=34%, signal=74% |
| 1649 | GSE29618\_PRE\_VS\_DAY7\_POST\_LAIV\_FLU\_VACCINE\_MDC\_UP |  | 19 | -0.22 | -0.96 | 0.510 | 0.725 | 1.000 | 207 | tags=26%, list=15%, signal=30% |
| 1650 | GSE369\_SOCS3\_KO\_VS\_WT\_LIVER\_DN |  | 17 | -0.22 | -0.96 | 0.501 | 0.725 | 1.000 | 67 | tags=18%, list=5%, signal=18% |
| 1651 | GSE3982\_MAST\_CELL\_VS\_TH2\_DN |  | 19 | -0.21 | -0.96 | 0.501 | 0.725 | 1.000 | 737 | tags=79%, list=52%, signal=163% |
| 1652 | GSE25846\_IL10\_POS\_VS\_NEG\_CD8\_TCELL\_DAY7\_POST\_CORONAVIRUS\_BRAIN\_UP |  | 21 | -0.20 | -0.96 | 0.476 | 0.726 | 1.000 | 328 | tags=33%, list=23%, signal=43% |
| 1653 | GSE17721\_CTRL\_VS\_PAM3CSK4\_6H\_BMDC\_UP |  | 16 | -0.23 | -0.96 | 0.494 | 0.728 | 1.000 | 125 | tags=25%, list=9%, signal=27% |
| 1654 | GSE20727\_ROS\_INH\_VS\_ROS\_INH\_AND\_DNFB\_ALLERGEN\_TREATED\_DC\_UP |  | 23 | -0.20 | -0.96 | 0.528 | 0.727 | 1.000 | 221 | tags=30%, list=16%, signal=35% |
| 1655 | GSE33425\_CD161\_INT\_VS\_NEG\_CD8\_TCELL\_DN |  | 20 | -0.21 | -0.96 | 0.505 | 0.727 | 1.000 | 505 | tags=65%, list=36%, signal=100% |
| 1656 | GSE21670\_STAT3\_KO\_VS\_WT\_CD4\_TCELL\_DN |  | 37 | -0.17 | -0.95 | 0.518 | 0.727 | 1.000 | 301 | tags=30%, list=21%, signal=37% |
| 1657 | GSE17721\_12H\_VS\_24H\_POLYIC\_BMDC\_DN |  | 17 | -0.22 | -0.95 | 0.491 | 0.728 | 1.000 | 171 | tags=29%, list=12%, signal=33% |
| 1658 | GSE46606\_UNSTIM\_VS\_CD40L\_IL2\_IL5\_DAY1\_STIMULATED\_BCELL\_DN |  | 23 | -0.20 | -0.95 | 0.518 | 0.728 | 1.000 | 236 | tags=26%, list=17%, signal=31% |
| 1659 | GSE14308\_TH2\_VS\_INDUCED\_TREG\_UP |  | 20 | -0.21 | -0.95 | 0.465 | 0.729 | 1.000 | 577 | tags=65%, list=41%, signal=108% |
| 1660 | GSE11961\_FOLLICULAR\_BCELL\_VS\_MEMORY\_BCELL\_DAY40\_UP |  | 18 | -0.22 | -0.95 | 0.488 | 0.729 | 1.000 | 327 | tags=39%, list=23%, signal=50% |
| 1661 | GSE46606\_UNSTIM\_VS\_CD40L\_IL2\_IL5\_1DAY\_STIMULATED\_IRF4\_KO\_BCELL\_DN |  | 19 | -0.21 | -0.95 | 0.491 | 0.731 | 1.000 | 510 | tags=58%, list=36%, signal=89% |
| 1662 | GSE16266\_CTRL\_VS\_LPS\_STIM\_MEF\_DN |  | 16 | -0.23 | -0.95 | 0.485 | 0.731 | 1.000 | 287 | tags=38%, list=20%, signal=47% |
| 1663 | GSE22935\_UNSTIM\_VS\_12H\_MBOVIS\_BCG\_STIM\_MACROPHAGE\_UP |  | 20 | -0.21 | -0.95 | 0.507 | 0.731 | 1.000 | 158 | tags=25%, list=11%, signal=28% |
| 1664 | GSE17721\_PAM3CSK4\_VS\_CPG\_12H\_BMDC\_UP |  | 20 | -0.21 | -0.95 | 0.515 | 0.732 | 1.000 | 78 | tags=15%, list=6%, signal=16% |
| 1665 | GSE27859\_CD11C\_INT\_F480\_HI\_MACROPHAGE\_VS\_CD11C\_ING\_F480\_INT\_DC\_UP |  | 20 | -0.21 | -0.95 | 0.523 | 0.732 | 1.000 | 173 | tags=25%, list=12%, signal=28% |
| 1666 | GSE24292\_WT\_VS\_PPARG\_KO\_MACROPHAGE\_DN |  | 28 | -0.18 | -0.95 | 0.523 | 0.732 | 1.000 | 172 | tags=25%, list=12%, signal=28% |
| 1667 | GSE46143\_CTRL\_VS\_LMP2A\_TRANSDUCED\_CD10\_POS\_GC\_BCELL\_UP |  | 15 | -0.23 | -0.95 | 0.494 | 0.733 | 1.000 | 955 | tags=93%, list=68%, signal=286% |
| 1668 | GSE10325\_LUPUS\_BCELL\_VS\_LUPUS\_MYELOID\_DN |  | 16 | -0.22 | -0.95 | 0.563 | 0.734 | 1.000 | 562 | tags=69%, list=40%, signal=113% |
| 1669 | GSE2770\_TGFB\_AND\_IL4\_VS\_IL12\_TREATED\_ACT\_CD4\_TCELL\_6H\_UP |  | 22 | -0.20 | -0.95 | 0.519 | 0.735 | 1.000 | 412 | tags=45%, list=29%, signal=63% |
| 1670 | GSE37301\_COMMON\_LYMPHOID\_PROGENITOR\_VS\_CD4\_TCELL\_UP |  | 27 | -0.19 | -0.95 | 0.542 | 0.735 | 1.000 | 947 | tags=89%, list=67%, signal=265% |
| 1671 | GSE33292\_DN3\_THYMOCYTE\_VS\_TCELL\_LYMPHOMA\_FROM\_TCF1\_KO\_DN |  | 19 | -0.21 | -0.95 | 0.525 | 0.735 | 1.000 | 256 | tags=32%, list=18%, signal=38% |
| 1672 | GSE44955\_MCSF\_VS\_MCSF\_AND\_IL27\_STIM\_MACROPHAGE\_DN |  | 19 | -0.21 | -0.95 | 0.506 | 0.734 | 1.000 | 313 | tags=37%, list=22%, signal=47% |
| 1673 | GSE34205\_HEALTHY\_VS\_FLU\_INF\_INFANT\_PBMC\_DN |  | 16 | -0.23 | -0.95 | 0.502 | 0.736 | 1.000 | 588 | tags=75%, list=42%, signal=127% |
| 1674 | GSE14000\_UNSTIM\_VS\_16H\_LPS\_DC\_DN |  | 24 | -0.19 | -0.95 | 0.520 | 0.735 | 1.000 | 549 | tags=67%, list=39%, signal=107% |
| 1675 | GSE1925\_3H\_VS\_24H\_IFNG\_STIM\_IFNG\_PRIMED\_MACROPHAGE\_UP |  | 20 | -0.21 | -0.95 | 0.515 | 0.736 | 1.000 | 348 | tags=40%, list=25%, signal=52% |
| 1676 | GSE37301\_HEMATOPOIETIC\_STEM\_CELL\_VS\_CD4\_TCELL\_UP |  | 20 | -0.21 | -0.95 | 0.522 | 0.736 | 1.000 | 180 | tags=30%, list=13%, signal=34% |
| 1677 | GSE8921\_UNSTIM\_VS\_TLR1\_2\_STIM\_MONOCYTE\_3H\_DN |  | 18 | -0.21 | -0.95 | 0.516 | 0.735 | 1.000 | 13 | tags=11%, list=1%, signal=11% |
| 1678 | GSE17721\_LPS\_VS\_CPG\_8H\_BMDC\_UP |  | 23 | -0.20 | -0.95 | 0.522 | 0.736 | 1.000 | 301 | tags=30%, list=21%, signal=38% |
| 1679 | GSE23925\_LIGHT\_ZONE\_VS\_NAIVE\_BCELL\_UP |  | 20 | -0.21 | -0.95 | 0.526 | 0.736 | 1.000 | 433 | tags=55%, list=31%, signal=78% |
| 1680 | GSE411\_WT\_VS\_SOCS3\_KO\_MACROPHAGE\_IL6\_STIM\_400MIN\_UP |  | 31 | -0.17 | -0.95 | 0.528 | 0.736 | 1.000 | 573 | tags=68%, list=41%, signal=112% |
| 1681 | GSE26030\_TH1\_VS\_TH17\_RESTIMULATED\_DAY15\_POST\_POLARIZATION\_DN |  | 16 | -0.22 | -0.94 | 0.494 | 0.736 | 1.000 | 973 | tags=94%, list=69%, signal=299% |
| 1682 | GSE27786\_CD4\_TCELL\_VS\_MONO\_MAC\_DN |  | 16 | -0.22 | -0.94 | 0.504 | 0.736 | 1.000 | 467 | tags=56%, list=33%, signal=83% |
| 1683 | GSE11961\_FOLLICULAR\_BCELL\_VS\_GERMINAL\_CENTER\_BCELL\_DAY7\_UP |  | 24 | -0.19 | -0.94 | 0.546 | 0.736 | 1.000 | 78 | tags=17%, list=6%, signal=17% |
| 1684 | GSE4748\_CTRL\_VS\_LPS\_AND\_CYANOBACTERIUM\_LPSLIKE\_STIM\_DC\_3H\_UP |  | 20 | -0.21 | -0.94 | 0.507 | 0.736 | 1.000 | 712 | tags=80%, list=50%, signal=159% |
| 1685 | GSE4748\_LPS\_VS\_LPS\_AND\_CYANOBACTERIUM\_LPSLIKE\_STIM\_DC\_3H\_UP |  | 19 | -0.22 | -0.94 | 0.531 | 0.736 | 1.000 | 417 | tags=47%, list=30%, signal=66% |
| 1686 | GSE19825\_NAIVE\_VS\_IL2RALOW\_DAY3\_EFF\_CD8\_TCELL\_DN |  | 23 | -0.19 | -0.94 | 0.528 | 0.736 | 1.000 | 831 | tags=87%, list=59%, signal=208% |
| 1687 | GSE14769\_UNSTIM\_VS\_80MIN\_LPS\_BMDM\_UP |  | 27 | -0.19 | -0.94 | 0.526 | 0.735 | 1.000 | 109 | tags=19%, list=8%, signal=20% |
| 1688 | GSE3982\_EOSINOPHIL\_VS\_BCELL\_DN |  | 25 | -0.19 | -0.94 | 0.530 | 0.736 | 1.000 | 299 | tags=32%, list=21%, signal=40% |
| 1689 | GSE30962\_PRIMARY\_VS\_SECONDARY\_ACUTE\_LCMV\_INF\_CD8\_TCELL\_UP |  | 17 | -0.21 | -0.94 | 0.506 | 0.736 | 1.000 | 729 | tags=76%, list=52%, signal=156% |
| 1690 | GSE11961\_FOLLICULAR\_BCELL\_VS\_MEMORY\_BCELL\_DAY7\_DN |  | 18 | -0.22 | -0.94 | 0.522 | 0.739 | 1.000 | 78 | tags=17%, list=6%, signal=17% |
| 1691 | GSE20715\_0H\_VS\_24H\_OZONE\_TLR4\_KO\_LUNG\_DN |  | 25 | -0.20 | -0.94 | 0.515 | 0.739 | 1.000 | 549 | tags=64%, list=39%, signal=103% |
| 1692 | GSE43863\_TFH\_VS\_LY6C\_INT\_CXCR5POS\_EFFECTOR\_CD4\_TCELL\_DN |  | 17 | -0.22 | -0.94 | 0.535 | 0.741 | 1.000 | 140 | tags=24%, list=10%, signal=26% |
| 1693 | GSE360\_DC\_VS\_MAC\_L\_MAJOR\_DN |  | 25 | -0.19 | -0.94 | 0.510 | 0.740 | 1.000 | 266 | tags=32%, list=19%, signal=39% |
| 1694 | GSE5589\_LPS\_VS\_LPS\_AND\_IL10\_STIM\_IL6\_KO\_MACROPHAGE\_45MIN\_UP |  | 17 | -0.22 | -0.94 | 0.532 | 0.740 | 1.000 | 495 | tags=59%, list=35%, signal=90% |
| 1695 | GSE2770\_IL12\_AND\_TGFB\_VS\_IL4\_TREATED\_ACT\_CD4\_TCELL\_48H\_UP |  | 20 | -0.20 | -0.94 | 0.529 | 0.740 | 1.000 | 61 | tags=15%, list=4%, signal=15% |
| 1696 | GSE5679\_PPARG\_LIGAND\_ROSIGLITAZONE\_VS\_ROSIGLITAZONE\_AND\_RARA\_AGONIST\_AM580\_TREATED\_DC\_DN |  | 18 | -0.21 | -0.94 | 0.492 | 0.740 | 1.000 | 171 | tags=22%, list=12%, signal=25% |
| 1697 | GSE36392\_TYPE\_2\_MYELOID\_VS\_NEUTROPHIL\_IL25\_TREATED\_LUNG\_DN |  | 17 | -0.22 | -0.94 | 0.548 | 0.739 | 1.000 | 292 | tags=35%, list=21%, signal=44% |
| 1698 | GSE3982\_CENT\_MEMORY\_CD4\_TCELL\_VS\_TH2\_UP |  | 24 | -0.19 | -0.94 | 0.545 | 0.739 | 1.000 | 513 | tags=54%, list=36%, signal=84% |
| 1699 | GSE39152\_BRAIN\_VS\_SPLEEN\_CD103\_NEG\_MEMORY\_CD8\_TCELL\_UP |  | 24 | -0.19 | -0.94 | 0.543 | 0.739 | 1.000 | 823 | tags=83%, list=58%, signal=197% |
| 1700 | GSE5589\_WT\_VS\_IL6\_KO\_LPS\_AND\_IL10\_STIM\_MACROPHAGE\_45MIN\_UP |  | 15 | -0.22 | -0.94 | 0.490 | 0.743 | 1.000 | 1098 | tags=100%, list=78%, signal=446% |
| 1701 | GSE25846\_IL10\_POS\_VS\_NEG\_CD8\_TCELL\_DAY7\_POST\_CORONAVIRUS\_BRAIN\_DN |  | 16 | -0.22 | -0.94 | 0.531 | 0.744 | 1.000 | 862 | tags=88%, list=61%, signal=222% |
| 1702 | GSE40277\_EOS\_AND\_LEF1\_TRANSDUCED\_VS\_GATA1\_AND\_SATB1\_TRANSDUCED\_CD4\_TCELL\_DN |  | 21 | -0.20 | -0.94 | 0.559 | 0.744 | 1.000 | 238 | tags=29%, list=17%, signal=34% |
| 1703 | GSE22033\_UNTREATED\_VS\_ROSIGLITAZONE\_TREATED\_MEF\_DN |  | 26 | -0.19 | -0.94 | 0.539 | 0.743 | 1.000 | 410 | tags=46%, list=29%, signal=64% |
| 1704 | GSE22601\_IMMATURE\_CD4\_SINGLE\_POSITIVE\_VS\_CD4\_SINGLE\_POSITIVE\_THYMOCYTE\_DN |  | 16 | -0.22 | -0.94 | 0.540 | 0.743 | 1.000 | 534 | tags=63%, list=38%, signal=99% |
| 1705 | GSE6674\_UNSTIM\_VS\_PL2\_3\_STIM\_BCELL\_UP |  | 16 | -0.22 | -0.94 | 0.501 | 0.743 | 1.000 | 52 | tags=13%, list=4%, signal=13% |
| 1706 | GSE3982\_CTRL\_VS\_LPS\_1H\_NEUTROPHIL\_DN |  | 18 | -0.22 | -0.94 | 0.518 | 0.743 | 1.000 | 55 | tags=17%, list=4%, signal=17% |
| 1707 | GSE8685\_IL2\_ACT\_IL2\_STARVED\_VS\_IL15\_ACT\_IL2\_STARVED\_CD4\_TCELL\_DN |  | 16 | -0.22 | -0.94 | 0.514 | 0.743 | 1.000 | 325 | tags=38%, list=23%, signal=48% |
| 1708 | GSE17721\_PAM3CSK4\_VS\_GADIQUIMOD\_8H\_BMDC\_UP |  | 22 | -0.20 | -0.93 | 0.551 | 0.744 | 1.000 | 56 | tags=14%, list=4%, signal=14% |
| 1709 | GSE27786\_CD4\_TCELL\_VS\_NEUTROPHIL\_UP |  | 24 | -0.19 | -0.93 | 0.549 | 0.744 | 1.000 | 10 | tags=8%, list=1%, signal=8% |
| 1710 | GSE15330\_WT\_VS\_IKAROS\_KO\_GRANULOCYTE\_MONOCYTE\_PROGENITOR\_UP |  | 21 | -0.20 | -0.93 | 0.520 | 0.744 | 1.000 | 335 | tags=38%, list=24%, signal=49% |
| 1711 | GSE22501\_PERIPHERAL\_BLOOD\_VS\_CORD\_BLOOD\_TREG\_DN |  | 16 | -0.22 | -0.93 | 0.501 | 0.746 | 1.000 | 203 | tags=31%, list=14%, signal=36% |
| 1712 | GSE1925\_CTRL\_VS\_24H\_IFNG\_STIM\_IFNG\_PRIMED\_MACROPHAGE\_DN |  | 22 | -0.20 | -0.93 | 0.538 | 0.746 | 1.000 | 72 | tags=14%, list=5%, signal=14% |
| 1713 | GSE9988\_LOW\_LPS\_VS\_VEHICLE\_TREATED\_MONOCYTE\_DN |  | 20 | -0.20 | -0.93 | 0.552 | 0.747 | 1.000 | 1018 | tags=95%, list=72%, signal=336% |
| 1714 | GSE2585\_AIRE\_KO\_VS\_WT\_CD80\_LOW\_MTEC\_DN |  | 15 | -0.22 | -0.93 | 0.542 | 0.747 | 1.000 | 576 | tags=67%, list=41%, signal=111% |
| 1715 | GSE36392\_EOSINOPHIL\_VS\_NEUTROPHIL\_IL25\_TREATED\_LUNG\_DN |  | 17 | -0.22 | -0.93 | 0.532 | 0.747 | 1.000 | 455 | tags=47%, list=32%, signal=69% |
| 1716 | GSE40685\_TREG\_VS\_FOXP3\_KO\_TREG\_PRECURSOR\_UP |  | 18 | -0.21 | -0.93 | 0.564 | 0.749 | 1.000 | 527 | tags=61%, list=37%, signal=96% |
| 1717 | GSE17974\_1H\_VS\_72H\_UNTREATED\_IN\_VITRO\_CD4\_TCELL\_UP |  | 28 | -0.18 | -0.93 | 0.546 | 0.751 | 1.000 | 1093 | tags=96%, list=77%, signal=419% |
| 1718 | GSE34392\_ST2\_KO\_VS\_WT\_DAY8\_LCMV\_EFFECTOR\_CD8\_TCELL\_UP |  | 18 | -0.22 | -0.93 | 0.530 | 0.752 | 1.000 | 251 | tags=33%, list=18%, signal=40% |
| 1719 | GSE37416\_CTRL\_VS\_12H\_F\_TULARENSIS\_LVS\_NEUTROPHIL\_UP |  | 26 | -0.18 | -0.93 | 0.535 | 0.752 | 1.000 | 147 | tags=23%, list=10%, signal=25% |
| 1720 | GSE21360\_NAIVE\_VS\_TERTIARY\_MEMORY\_CD8\_TCELL\_UP |  | 16 | -0.22 | -0.93 | 0.528 | 0.752 | 1.000 | 475 | tags=56%, list=34%, signal=84% |
| 1721 | GSE22313\_HEALTHY\_VS\_SLE\_MOUSE\_CD4\_TCELL\_DN |  | 22 | -0.20 | -0.93 | 0.540 | 0.753 | 1.000 | 124 | tags=18%, list=9%, signal=20% |
| 1722 | GSE11961\_FOLLICULAR\_BCELL\_VS\_GERMINAL\_CENTER\_BCELL\_DAY40\_UP |  | 32 | -0.17 | -0.93 | 0.558 | 0.753 | 1.000 | 473 | tags=50%, list=34%, signal=74% |
| 1723 | GSE15330\_LYMPHOID\_MULTIPOTENT\_VS\_PRO\_BCELL\_UP |  | 21 | -0.20 | -0.93 | 0.568 | 0.753 | 1.000 | 317 | tags=38%, list=22%, signal=48% |
| 1724 | GSE25088\_WT\_VS\_STAT6\_KO\_MACROPHAGE\_IL4\_STIM\_UP |  | 16 | -0.23 | -0.93 | 0.553 | 0.752 | 1.000 | 321 | tags=44%, list=23%, signal=56% |
| 1725 | GSE3982\_BASOPHIL\_VS\_EFF\_MEMORY\_CD4\_TCELL\_UP |  | 36 | -0.17 | -0.93 | 0.577 | 0.753 | 1.000 | 99 | tags=14%, list=7%, signal=15% |
| 1726 | GSE19941\_UNSTIM\_VS\_LPS\_STIM\_IL10\_KO\_MACROPHAGE\_DN |  | 17 | -0.22 | -0.93 | 0.516 | 0.753 | 1.000 | 440 | tags=53%, list=31%, signal=76% |
| 1727 | GSE29949\_MICROGLIA\_BRAIN\_VS\_MONOCYTE\_BONE\_MARROW\_UP |  | 19 | -0.21 | -0.93 | 0.528 | 0.753 | 1.000 | 140 | tags=21%, list=10%, signal=23% |
| 1728 | GSE17721\_0.5H\_VS\_8H\_CPG\_BMDC\_DN |  | 25 | -0.19 | -0.93 | 0.568 | 0.753 | 1.000 | 514 | tags=60%, list=36%, signal=93% |
| 1729 | GSE15735\_2H\_VS\_12H\_HDAC\_INHIBITOR\_TREATED\_CD4\_TCELL\_DN |  | 17 | -0.22 | -0.92 | 0.552 | 0.753 | 1.000 | 411 | tags=47%, list=29%, signal=66% |
| 1730 | GSE22611\_NOD2\_TRANSDUCED\_VS\_CTRL\_HEK293T\_STIMULATED\_WITH\_MDP\_2H\_DN |  | 26 | -0.18 | -0.92 | 0.571 | 0.753 | 1.000 | 153 | tags=19%, list=11%, signal=21% |
| 1731 | GSE29164\_UNTREATED\_VS\_CD8\_TCELL\_TREATED\_MELANOMA\_DAY3\_DN |  | 16 | -0.22 | -0.92 | 0.526 | 0.755 | 1.000 | 918 | tags=94%, list=65%, signal=265% |
| 1732 | GSE17721\_CTRL\_VS\_GARDIQUIMOD\_2H\_BMDC\_DN |  | 21 | -0.19 | -0.92 | 0.569 | 0.755 | 1.000 | 806 | tags=81%, list=57%, signal=186% |
| 1733 | GSE23114\_PERITONEAL\_CAVITY\_B1A\_BCELL\_VS\_SPLEEN\_BCELL\_IN\_SLE2C1\_MOUSE\_UP |  | 16 | -0.22 | -0.92 | 0.550 | 0.755 | 1.000 | 175 | tags=25%, list=12%, signal=28% |
| 1734 | GOLDRATH\_NAIVE\_VS\_EFF\_CD8\_TCELL\_UP |  | 24 | -0.19 | -0.92 | 0.573 | 0.755 | 1.000 | 1145 | tags=100%, list=81%, signal=521% |
| 1735 | GSE22886\_NAIVE\_BCELL\_VS\_DC\_UP |  | 19 | -0.21 | -0.92 | 0.580 | 0.757 | 1.000 | 184 | tags=26%, list=13%, signal=30% |
| 1736 | GSE10239\_MEMORY\_VS\_DAY4.5\_EFF\_CD8\_TCELL\_DN |  | 17 | -0.21 | -0.92 | 0.549 | 0.757 | 1.000 | 983 | tags=94%, list=70%, signal=307% |
| 1737 | GSE2585\_CTEC\_VS\_MTEC\_THYMUS\_UP |  | 17 | -0.21 | -0.92 | 0.551 | 0.757 | 1.000 | 279 | tags=35%, list=20%, signal=43% |
| 1738 | GSE36476\_YOUNG\_VS\_OLD\_DONOR\_MEMORY\_CD4\_TCELL\_16H\_TSST\_ACT\_UP |  | 21 | -0.19 | -0.92 | 0.556 | 0.759 | 1.000 | 1048 | tags=95%, list=74%, signal=365% |
| 1739 | GSE11057\_CD4\_EFF\_MEM\_VS\_PBMC\_DN |  | 18 | -0.21 | -0.92 | 0.573 | 0.759 | 1.000 | 465 | tags=56%, list=33%, signal=82% |
| 1740 | GSE18791\_CTRL\_VS\_NEWCASTLE\_VIRUS\_DC\_2H\_DN |  | 23 | -0.19 | -0.92 | 0.540 | 0.762 | 1.000 | 508 | tags=57%, list=36%, signal=87% |
| 1741 | GSE18281\_SUBCAPSULAR\_VS\_CENTRAL\_CORTICAL\_REGION\_OF\_THYMUS\_DN |  | 20 | -0.20 | -0.92 | 0.542 | 0.765 | 1.000 | 547 | tags=60%, list=39%, signal=97% |
| 1742 | GSE21380\_NON\_TFH\_VS\_TFH\_CD4\_TCELL\_UP |  | 18 | -0.21 | -0.92 | 0.550 | 0.765 | 1.000 | 143 | tags=22%, list=10%, signal=24% |
| 1743 | GSE26495\_PD1HIGH\_VS\_PD1LOW\_CD8\_TCELL\_DN |  | 18 | -0.21 | -0.92 | 0.550 | 0.764 | 1.000 | 275 | tags=33%, list=19%, signal=41% |
| 1744 | GSE27786\_CD8\_TCELL\_VS\_MONO\_MAC\_UP |  | 28 | -0.17 | -0.91 | 0.586 | 0.765 | 1.000 | 225 | tags=21%, list=16%, signal=25% |
| 1745 | GSE5589\_WT\_VS\_IL6\_KO\_LPS\_STIM\_MACROPHAGE\_45MIN\_UP |  | 19 | -0.21 | -0.91 | 0.568 | 0.765 | 1.000 | 129 | tags=21%, list=9%, signal=23% |
| 1746 | GSE3982\_DC\_VS\_TH1\_UP |  | 19 | -0.20 | -0.91 | 0.559 | 0.765 | 1.000 | 306 | tags=37%, list=22%, signal=46% |
| 1747 | GSE28237\_EARLY\_VS\_LATE\_GC\_BCELL\_DN |  | 16 | -0.22 | -0.91 | 0.567 | 0.765 | 1.000 | 911 | tags=94%, list=65%, signal=262% |
| 1748 | GSE24972\_WT\_VS\_IRF8\_KO\_MARGINAL\_ZONE\_SPLEEN\_BCELL\_UP |  | 20 | -0.20 | -0.91 | 0.545 | 0.766 | 1.000 | 104 | tags=20%, list=7%, signal=21% |
| 1749 | GSE40666\_NAIVE\_VS\_EFFECTOR\_CD8\_TCELL\_DN |  | 17 | -0.22 | -0.91 | 0.563 | 0.766 | 1.000 | 180 | tags=29%, list=13%, signal=33% |
| 1750 | GSE7768\_OVA\_ALONE\_VS\_OVA\_WITH\_LPS\_IMMUNIZED\_MOUSE\_WHOLE\_SPLEEN\_6H\_DN |  | 25 | -0.18 | -0.91 | 0.565 | 0.769 | 1.000 | 341 | tags=40%, list=24%, signal=52% |
| 1751 | GSE37534\_UNTREATED\_VS\_PIOGLITAZONE\_TREATED\_CD4\_TCELL\_PPARG1\_AND\_FOXP3\_TRASDUCED\_DN |  | 27 | -0.17 | -0.91 | 0.569 | 0.770 | 1.000 | 370 | tags=37%, list=26%, signal=49% |
| 1752 | GSE32986\_UNSTIM\_VS\_GMCSF\_AND\_CURDLAN\_LOWDOSE\_STIM\_DC\_UP |  | 22 | -0.20 | -0.91 | 0.554 | 0.775 | 1.000 | 579 | tags=64%, list=41%, signal=106% |
| 1753 | GSE41176\_WT\_VS\_TAK1\_KO\_ANTI\_IGM\_STIM\_BCELL\_1H\_UP |  | 19 | -0.21 | -0.91 | 0.546 | 0.775 | 1.000 | 54 | tags=16%, list=4%, signal=16% |
| 1754 | GSE46242\_CTRL\_VS\_EGR2\_DELETED\_ANERGIC\_TH1\_CD4\_TCELL\_DN |  | 21 | -0.19 | -0.91 | 0.580 | 0.775 | 1.000 | 522 | tags=57%, list=37%, signal=89% |
| 1755 | GSE33374\_CD8\_ALPHAALPHA\_VS\_ALPHABETA\_CD161\_HIGH\_TCELL\_DN |  | 17 | -0.21 | -0.91 | 0.607 | 0.776 | 1.000 | 615 | tags=71%, list=44%, signal=124% |
| 1756 | GSE3982\_EFF\_MEMORY\_CD4\_TCELL\_VS\_NKCELL\_DN |  | 19 | -0.20 | -0.91 | 0.591 | 0.776 | 1.000 | 215 | tags=26%, list=15%, signal=31% |
| 1757 | GSE42021\_CD24INT\_VS\_CD24LOW\_TREG\_THYMUS\_DN |  | 31 | -0.17 | -0.91 | 0.570 | 0.777 | 1.000 | 828 | tags=81%, list=59%, signal=191% |
| 1758 | GSE5455\_HEALTHY\_VS\_TUMOR\_BEARING\_MOUSE\_SPLEEN\_MONOCYTE\_UP |  | 15 | -0.22 | -0.91 | 0.556 | 0.776 | 1.000 | 451 | tags=53%, list=32%, signal=78% |
| 1759 | GSE3039\_CD4\_TCELL\_VS\_B2\_BCELL\_DN |  | 19 | -0.20 | -0.90 | 0.574 | 0.777 | 1.000 | 618 | tags=68%, list=44%, signal=120% |
| 1760 | GSE15330\_LYMPHOID\_MULTIPOTENT\_VS\_GRANULOCYTE\_MONOCYTE\_PROGENITOR\_IKAROS\_KO\_UP |  | 24 | -0.18 | -0.90 | 0.577 | 0.777 | 1.000 | 111 | tags=21%, list=8%, signal=22% |
| 1761 | GSE12845\_IGD\_POS\_BLOOD\_VS\_PRE\_GC\_TONSIL\_BCELL\_UP |  | 19 | -0.20 | -0.90 | 0.564 | 0.781 | 1.000 | 33 | tags=11%, list=2%, signal=11% |
| 1762 | GSE37416\_CTRL\_VS\_6H\_F\_TULARENSIS\_LVS\_NEUTROPHIL\_UP |  | 27 | -0.17 | -0.90 | 0.608 | 0.781 | 1.000 | 154 | tags=19%, list=11%, signal=20% |
| 1763 | GSE46242\_CTRL\_VS\_EGR2\_DELETED\_TH1\_CD4\_TCELL\_DN |  | 18 | -0.20 | -0.90 | 0.607 | 0.782 | 1.000 | 212 | tags=28%, list=15%, signal=32% |
| 1764 | GSE9650\_GP33\_VS\_GP276\_LCMV\_SPECIFIC\_EXHAUSTED\_CD8\_TCELL\_DN |  | 21 | -0.19 | -0.90 | 0.580 | 0.782 | 1.000 | 1149 | tags=100%, list=81%, signal=531% |
| 1765 | GSE29618\_PRE\_VS\_DAY7\_POST\_LAIV\_FLU\_VACCINE\_MONOCYTE\_DN |  | 32 | -0.16 | -0.90 | 0.591 | 0.782 | 1.000 | 351 | tags=38%, list=25%, signal=49% |
| 1766 | GSE16451\_CTRL\_VS\_WEST\_EQUINE\_ENC\_VIRUS\_IMMATURE\_NEURON\_CELL\_LINE\_UP |  | 17 | -0.21 | -0.90 | 0.571 | 0.782 | 1.000 | 274 | tags=35%, list=19%, signal=43% |
| 1767 | GSE24142\_DN2\_VS\_DN3\_THYMOCYTE\_FETAL\_UP |  | 29 | -0.17 | -0.90 | 0.576 | 0.781 | 1.000 | 153 | tags=21%, list=11%, signal=23% |
| 1768 | GSE6875\_TCONV\_VS\_TREG\_UP |  | 17 | -0.21 | -0.90 | 0.568 | 0.782 | 1.000 | 137 | tags=24%, list=10%, signal=26% |
| 1769 | GSE360\_CTRL\_VS\_B\_MALAYI\_HIGH\_DOSE\_DC\_UP |  | 21 | -0.19 | -0.90 | 0.609 | 0.782 | 1.000 | 256 | tags=29%, list=18%, signal=34% |
| 1770 | GSE32423\_IL7\_VS\_IL4\_MEMORY\_CD8\_TCELL\_UP |  | 23 | -0.19 | -0.90 | 0.577 | 0.782 | 1.000 | 523 | tags=57%, list=37%, signal=88% |
| 1771 | GSE15330\_MEGAKARYOCYTE\_ERYTHROID\_PROGENITOR\_VS\_PRO\_BCELL\_UP |  | 20 | -0.20 | -0.90 | 0.597 | 0.783 | 1.000 | 944 | tags=90%, list=67%, signal=268% |
| 1772 | GSE14308\_TH1\_VS\_TH17\_UP |  | 21 | -0.19 | -0.90 | 0.575 | 0.783 | 1.000 | 140 | tags=19%, list=10%, signal=21% |
| 1773 | GSE14769\_UNSTIM\_VS\_40MIN\_LPS\_BMDM\_UP |  | 15 | -0.22 | -0.90 | 0.571 | 0.784 | 1.000 | 177 | tags=27%, list=13%, signal=30% |
| 1774 | GSE9006\_TYPE\_1\_DIABETES\_AT\_DX\_VS\_1MONTH\_POST\_DX\_PBMC\_UP |  | 22 | -0.19 | -0.90 | 0.601 | 0.784 | 1.000 | 569 | tags=64%, list=40%, signal=105% |
| 1775 | GSE25087\_TREG\_VS\_TCONV\_FETUS\_DN |  | 19 | -0.20 | -0.90 | 0.575 | 0.784 | 1.000 | 357 | tags=42%, list=25%, signal=56% |
| 1776 | GSE9037\_CTRL\_VS\_LPS\_4H\_STIM\_IRAK4\_KO\_BMDM\_DN |  | 29 | -0.17 | -0.90 | 0.587 | 0.786 | 1.000 | 303 | tags=31%, list=21%, signal=39% |
| 1777 | GSE16450\_IMMATURE\_VS\_MATURE\_NEURON\_CELL\_LINE\_12H\_IFNA\_STIM\_DN |  | 32 | -0.17 | -0.90 | 0.594 | 0.786 | 1.000 | 180 | tags=22%, list=13%, signal=25% |
| 1778 | GSE21670\_IL6\_VS\_TGFB\_AND\_IL6\_TREATED\_STAT3\_KO\_CD4\_TCELL\_UP |  | 17 | -0.21 | -0.90 | 0.584 | 0.786 | 1.000 | 571 | tags=65%, list=40%, signal=107% |
| 1779 | GSE29949\_CD8\_NEG\_DC\_SPLEEN\_VS\_MONOCYTE\_BONE\_MARROW\_UP |  | 25 | -0.18 | -0.89 | 0.569 | 0.788 | 1.000 | 417 | tags=44%, list=30%, signal=61% |
| 1780 | GSE41867\_NAIVE\_VS\_DAY8\_LCMV\_ARMSTRONG\_EFFECTOR\_CD8\_TCELL\_DN |  | 21 | -0.19 | -0.89 | 0.558 | 0.788 | 1.000 | 380 | tags=38%, list=27%, signal=51% |
| 1781 | GSE21063\_WT\_VS\_NFATC1\_KO\_3H\_ANTI\_IGM\_STIM\_BCELL\_UP |  | 19 | -0.20 | -0.89 | 0.580 | 0.789 | 1.000 | 480 | tags=53%, list=34%, signal=79% |
| 1782 | GSE8835\_HEALTHY\_VS\_CLL\_CD4\_TCELL\_UP |  | 18 | -0.21 | -0.89 | 0.607 | 0.791 | 1.000 | 228 | tags=33%, list=16%, signal=39% |
| 1783 | GSE27092\_WT\_VS\_HDAC7\_PHOSPHO\_DEFICIENT\_CD8\_TCELL\_DN |  | 16 | -0.21 | -0.89 | 0.614 | 0.791 | 1.000 | 59 | tags=13%, list=4%, signal=13% |
| 1784 | GSE13484\_12H\_UNSTIM\_VS\_YF17D\_VACCINE\_STIM\_PBMC\_DN |  | 19 | -0.20 | -0.89 | 0.599 | 0.791 | 1.000 | 372 | tags=42%, list=26%, signal=56% |
| 1785 | GSE21927\_SPLEEN\_MONOCYTE\_VS\_GMCSF\_GCSF\_BONE\_MARROW\_DN |  | 18 | -0.20 | -0.89 | 0.594 | 0.791 | 1.000 | 216 | tags=28%, list=15%, signal=32% |
| 1786 | GSE7831\_CPG\_VS\_INFLUENZA\_STIM\_PDC\_4H\_UP |  | 23 | -0.19 | -0.89 | 0.606 | 0.790 | 1.000 | 504 | tags=52%, list=36%, signal=80% |
| 1787 | GSE17186\_BLOOD\_VS\_CORD\_BLOOD\_CD21HIGH\_TRANSITIONAL\_BCELL\_DN |  | 16 | -0.22 | -0.89 | 0.576 | 0.790 | 1.000 | 256 | tags=31%, list=18%, signal=38% |
| 1788 | GSE2585\_CTEC\_VS\_THYMIC\_DC\_UP |  | 20 | -0.19 | -0.89 | 0.563 | 0.791 | 1.000 | 269 | tags=30%, list=19%, signal=37% |
| 1789 | GSE41978\_ID2\_KO\_VS\_BIM\_KO\_KLRG1\_LOW\_EFFECTOR\_CD8\_TCELL\_UP |  | 33 | -0.16 | -0.89 | 0.620 | 0.791 | 1.000 | 582 | tags=61%, list=41%, signal=101% |
| 1790 | GSE17721\_LPS\_VS\_GARDIQUIMOD\_24H\_BMDC\_DN |  | 20 | -0.20 | -0.89 | 0.578 | 0.794 | 1.000 | 207 | tags=25%, list=15%, signal=29% |
| 1791 | GSE29949\_CD8\_POS\_DC\_SPLEEN\_VS\_MONOCYTE\_BONE\_MARROW\_DN |  | 24 | -0.18 | -0.89 | 0.582 | 0.793 | 1.000 | 702 | tags=75%, list=50%, signal=147% |
| 1792 | GSE42724\_NAIVE\_VS\_MEMORY\_BCELL\_UP |  | 15 | -0.21 | -0.89 | 0.561 | 0.793 | 1.000 | 188 | tags=20%, list=13%, signal=23% |
| 1793 | GSE41978\_ID2\_KO\_AND\_BIM\_KO\_VS\_BIM\_KO\_KLRG1\_LOW\_EFFECTOR\_CD8\_TCELL\_DN |  | 24 | -0.18 | -0.89 | 0.587 | 0.794 | 1.000 | 153 | tags=21%, list=11%, signal=23% |
| 1794 | GSE14769\_UNSTIM\_VS\_360MIN\_LPS\_BMDM\_UP |  | 20 | -0.20 | -0.89 | 0.610 | 0.794 | 1.000 | 918 | tags=90%, list=65%, signal=254% |
| 1795 | GSE5589\_LPS\_VS\_LPS\_AND\_IL10\_STIM\_IL6\_KO\_MACROPHAGE\_180MIN\_DN |  | 21 | -0.19 | -0.89 | 0.591 | 0.794 | 1.000 | 54 | tags=14%, list=4%, signal=15% |
| 1796 | GSE21927\_UNTREATED\_VS\_GMCSF\_GCSF\_TREATED\_BONE\_MARROW\_DN |  | 19 | -0.20 | -0.89 | 0.575 | 0.793 | 1.000 | 225 | tags=32%, list=16%, signal=37% |
| 1797 | GSE6259\_FLT3L\_INDUCED\_VS\_WT\_SPLENIC\_DC\_33D1\_POS\_UP |  | 19 | -0.19 | -0.89 | 0.617 | 0.795 | 1.000 | 273 | tags=32%, list=19%, signal=39% |
| 1798 | GSE7509\_DC\_VS\_MONOCYTE\_UP |  | 22 | -0.19 | -0.88 | 0.598 | 0.797 | 1.000 | 173 | tags=23%, list=12%, signal=25% |
| 1799 | GSE27786\_LSK\_VS\_CD4\_TCELL\_UP |  | 19 | -0.20 | -0.88 | 0.592 | 0.797 | 1.000 | 24 | tags=11%, list=2%, signal=11% |
| 1800 | GSE25088\_CTRL\_VS\_IL4\_AND\_ROSIGLITAZONE\_STIM\_MACROPHAGE\_UP |  | 16 | -0.21 | -0.88 | 0.587 | 0.797 | 1.000 | 477 | tags=56%, list=34%, signal=84% |
| 1801 | GSE5503\_PLN\_DC\_VS\_SPLEEN\_DC\_ACTIVATED\_ALLOGENIC\_TCELL\_DN |  | 15 | -0.22 | -0.88 | 0.602 | 0.798 | 1.000 | 26 | tags=13%, list=2%, signal=13% |
| 1802 | GSE24574\_BCL6\_LOW\_TFH\_VS\_NAIVE\_CD4\_TCELL\_UP |  | 17 | -0.20 | -0.88 | 0.602 | 0.798 | 1.000 | 94 | tags=18%, list=7%, signal=19% |
| 1803 | GSE7509\_UNSTIM\_VS\_FCGRIIB\_STIM\_MONOCYTE\_UP |  | 16 | -0.21 | -0.88 | 0.595 | 0.798 | 1.000 | 620 | tags=69%, list=44%, signal=121% |
| 1804 | GSE11961\_MEMORY\_BCELL\_DAY7\_VS\_MEMORY\_BCELL\_DAY40\_UP |  | 19 | -0.20 | -0.88 | 0.598 | 0.798 | 1.000 | 433 | tags=47%, list=31%, signal=67% |
| 1805 | GSE37301\_HEMATOPOIETIC\_STEM\_CELL\_VS\_LYMPHOID\_PRIMED\_MPP\_DN |  | 21 | -0.19 | -0.88 | 0.617 | 0.798 | 1.000 | 127 | tags=19%, list=9%, signal=21% |
| 1806 | GSE9037\_CTRL\_VS\_LPS\_1H\_STIM\_BMDM\_DN |  | 32 | -0.16 | -0.88 | 0.594 | 0.798 | 1.000 | 400 | tags=44%, list=28%, signal=60% |
| 1807 | GSE17186\_NAIVE\_VS\_CD21HIGH\_TRANSITIONAL\_BCELL\_CORD\_BLOOD\_DN |  | 22 | -0.19 | -0.88 | 0.634 | 0.798 | 1.000 | 256 | tags=32%, list=18%, signal=38% |
| 1808 | GSE36392\_MAC\_VS\_NEUTROPHIL\_IL25\_TREATED\_LUNG\_UP |  | 21 | -0.19 | -0.88 | 0.616 | 0.798 | 1.000 | 263 | tags=29%, list=19%, signal=35% |
| 1809 | GSE29949\_CD8\_NEG\_DC\_SPLEEN\_VS\_DC\_BRAIN\_DN |  | 20 | -0.19 | -0.88 | 0.637 | 0.797 | 1.000 | 1010 | tags=95%, list=72%, signal=330% |
| 1810 | GSE36826\_WT\_VS\_IL1R\_KO\_SKIN\_STAPH\_AUREUS\_INF\_UP |  | 23 | -0.18 | -0.88 | 0.614 | 0.798 | 1.000 | 171 | tags=22%, list=12%, signal=24% |
| 1811 | GSE15139\_GMCSF\_TREATED\_VS\_UNTREATED\_NEUTROPHILS\_DN |  | 28 | -0.17 | -0.88 | 0.640 | 0.798 | 1.000 | 78 | tags=14%, list=6%, signal=15% |
| 1812 | GSE17721\_POLYIC\_VS\_GARDIQUIMOD\_24H\_BMDC\_DN |  | 15 | -0.21 | -0.88 | 0.597 | 0.798 | 1.000 | 83 | tags=20%, list=6%, signal=21% |
| 1813 | GSE11924\_TFH\_VS\_TH1\_CD4\_TCELL\_UP |  | 15 | -0.21 | -0.88 | 0.601 | 0.798 | 1.000 | 252 | tags=33%, list=18%, signal=40% |
| 1814 | GSE23505\_UNTREATED\_VS\_4DAY\_IL6\_IL1\_TGFB\_TREATED\_CD4\_TCELL\_DN |  | 18 | -0.21 | -0.88 | 0.601 | 0.798 | 1.000 | 284 | tags=33%, list=20%, signal=41% |
| 1815 | GSE39110\_DAY3\_VS\_DAY6\_POST\_IMMUNIZATION\_CD8\_TCELL\_WITH\_IL2\_TREATMENT\_DN |  | 27 | -0.17 | -0.88 | 0.634 | 0.798 | 1.000 | 104 | tags=15%, list=7%, signal=16% |
| 1816 | GSE22611\_NOD2\_TRANSD\_VS\_CTRL\_TRANSD\_HEK293\_MDP\_STIM\_6H\_UP |  | 16 | -0.21 | -0.88 | 0.608 | 0.798 | 1.000 | 216 | tags=31%, list=15%, signal=36% |
| 1817 | GSE3920\_IFNA\_VS\_IFNG\_TREATED\_FIBROBLAST\_UP |  | 18 | -0.20 | -0.88 | 0.617 | 0.798 | 1.000 | 485 | tags=56%, list=34%, signal=84% |
| 1818 | GSE3982\_DC\_VS\_MAC\_LPS\_STIM\_UP |  | 26 | -0.17 | -0.88 | 0.630 | 0.799 | 1.000 | 104 | tags=15%, list=7%, signal=16% |
| 1819 | GSE15330\_WT\_VS\_IKAROS\_KO\_MEGAKARYOCYTE\_ERYTHROID\_PROGENITOR\_DN |  | 22 | -0.19 | -0.88 | 0.612 | 0.799 | 1.000 | 563 | tags=59%, list=40%, signal=97% |
| 1820 | GSE27786\_BCELL\_VS\_NKCELL\_DN |  | 23 | -0.18 | -0.88 | 0.613 | 0.800 | 1.000 | 416 | tags=48%, list=29%, signal=67% |
| 1821 | GSE44649\_NAIVE\_VS\_ACTIVATED\_CD8\_TCELL\_MIR155\_KO\_UP |  | 31 | -0.16 | -0.88 | 0.589 | 0.803 | 1.000 | 876 | tags=84%, list=62%, signal=216% |
| 1822 | GSE18893\_TCONV\_VS\_TREG\_2H\_CULTURE\_DN |  | 16 | -0.21 | -0.88 | 0.597 | 0.803 | 1.000 | 419 | tags=50%, list=30%, signal=70% |
| 1823 | GSE18791\_CTRL\_VS\_NEWCASTLE\_VIRUS\_DC\_10H\_DN |  | 22 | -0.18 | -0.88 | 0.619 | 0.803 | 1.000 | 332 | tags=36%, list=24%, signal=47% |
| 1824 | GSE32986\_CURDLAN\_LOWDOSE\_VS\_GMCSF\_AND\_CURDLAN\_LOWDOSE\_STIM\_DC\_UP |  | 19 | -0.20 | -0.88 | 0.611 | 0.803 | 1.000 | 419 | tags=47%, list=30%, signal=66% |
| 1825 | GSE11961\_GERMINAL\_CENTER\_BCELL\_DAY7\_VS\_PLASMA\_CELL\_DAY7\_DN |  | 23 | -0.18 | -0.87 | 0.603 | 0.803 | 1.000 | 206 | tags=26%, list=15%, signal=30% |
| 1826 | GSE11864\_CSF1\_VS\_CSF1\_PAM3CYS\_IN\_MAC\_DN |  | 15 | -0.22 | -0.87 | 0.623 | 0.803 | 1.000 | 194 | tags=27%, list=14%, signal=31% |
| 1827 | GSE22432\_MULTIPOTENT\_VS\_COMMON\_DC\_PROGENITOR\_UP |  | 16 | -0.21 | -0.87 | 0.588 | 0.805 | 1.000 | 166 | tags=25%, list=12%, signal=28% |
| 1828 | GSE3982\_BCELL\_VS\_EFF\_MEMORY\_CD4\_TCELL\_UP |  | 23 | -0.18 | -0.87 | 0.621 | 0.805 | 1.000 | 607 | tags=65%, list=43%, signal=113% |
| 1829 | GSE24634\_TEFF\_VS\_TCONV\_DAY3\_IN\_CULTURE\_DN |  | 15 | -0.22 | -0.87 | 0.609 | 0.805 | 1.000 | 170 | tags=27%, list=12%, signal=30% |
| 1830 | GSE5542\_IFNA\_VS\_IFNA\_AND\_IFNG\_TREATED\_EPITHELIAL\_CELLS\_24H\_UP |  | 20 | -0.19 | -0.87 | 0.622 | 0.805 | 1.000 | 199 | tags=25%, list=14%, signal=29% |
| 1831 | GSE17721\_LPS\_VS\_PAM3CSK4\_8H\_BMDC\_UP |  | 20 | -0.19 | -0.87 | 0.607 | 0.806 | 1.000 | 362 | tags=45%, list=26%, signal=60% |
| 1832 | GSE22886\_IGG\_IGA\_MEMORY\_BCELL\_VS\_BM\_PLASMA\_CELL\_UP |  | 20 | -0.19 | -0.87 | 0.650 | 0.806 | 1.000 | 256 | tags=30%, list=18%, signal=36% |
| 1833 | GSE21379\_WT\_VS\_SAP\_KO\_CD4\_TCELL\_UP |  | 15 | -0.21 | -0.87 | 0.613 | 0.807 | 1.000 | 446 | tags=47%, list=32%, signal=68% |
| 1834 | GSE32986\_GMCSF\_VS\_GMCSF\_AND\_CURDLAN\_HIGHDOSE\_STIM\_DC\_UP |  | 23 | -0.18 | -0.87 | 0.601 | 0.807 | 1.000 | 383 | tags=39%, list=27%, signal=53% |
| 1835 | GSE9650\_EFFECTOR\_VS\_EXHAUSTED\_CD8\_TCELL\_DN |  | 20 | -0.19 | -0.87 | 0.656 | 0.810 | 1.000 | 303 | tags=35%, list=21%, signal=44% |
| 1836 | GSE26343\_UNSTIM\_VS\_LPS\_STIM\_NFAT5\_KO\_MACROPHAGE\_UP |  | 26 | -0.17 | -0.87 | 0.617 | 0.810 | 1.000 | 167 | tags=19%, list=12%, signal=21% |
| 1837 | GSE17974\_0H\_VS\_0.5H\_IN\_VITRO\_ACT\_CD4\_TCELL\_DN |  | 17 | -0.21 | -0.87 | 0.631 | 0.810 | 1.000 | 539 | tags=65%, list=38%, signal=103% |
| 1838 | GSE30083\_SP1\_VS\_SP4\_THYMOCYTE\_UP |  | 19 | -0.20 | -0.87 | 0.633 | 0.811 | 1.000 | 271 | tags=32%, list=19%, signal=39% |
| 1839 | GSE2405\_0H\_VS\_12H\_A\_PHAGOCYTOPHILUM\_STIM\_NEUTROPHIL\_UP |  | 20 | -0.19 | -0.87 | 0.620 | 0.811 | 1.000 | 557 | tags=60%, list=39%, signal=98% |
| 1840 | GSE15930\_NAIVE\_VS\_48H\_IN\_VITRO\_STIM\_IL12\_CD8\_TCELL\_UP |  | 17 | -0.20 | -0.87 | 0.638 | 0.811 | 1.000 | 207 | tags=29%, list=15%, signal=34% |
| 1841 | GSE5589\_IL6\_KO\_VS\_IL10\_KO\_LPS\_STIM\_MACROPHAGE\_180MIN\_DN |  | 17 | -0.20 | -0.87 | 0.640 | 0.811 | 1.000 | 80 | tags=18%, list=6%, signal=18% |
| 1842 | GSE39820\_CTRL\_VS\_IL1B\_IL6\_CD4\_TCELL\_UP |  | 23 | -0.18 | -0.87 | 0.642 | 0.813 | 1.000 | 1164 | tags=100%, list=82%, signal=562% |
| 1843 | GSE15930\_STIM\_VS\_STIM\_AND\_IFNAB\_48H\_CD8\_T\_CELL\_UP |  | 15 | -0.21 | -0.86 | 0.628 | 0.813 | 1.000 | 987 | tags=93%, list=70%, signal=307% |
| 1844 | GSE11924\_TH1\_VS\_TH17\_CD4\_TCELL\_DN |  | 21 | -0.18 | -0.86 | 0.623 | 0.813 | 1.000 | 951 | tags=90%, list=67%, signal=273% |
| 1845 | GSE22611\_UNSTIM\_VS\_6H\_MDP\_STIM\_NOD2\_TRANSDUCED\_HEK293T\_CELL\_DN |  | 28 | -0.17 | -0.86 | 0.659 | 0.814 | 1.000 | 45 | tags=11%, list=3%, signal=11% |
| 1846 | GSE22432\_UNTREATED\_VS\_TGFB1\_TREATED\_COMMON\_DC\_PROGENITOR\_UP |  | 21 | -0.18 | -0.86 | 0.603 | 0.814 | 1.000 | 456 | tags=52%, list=32%, signal=76% |
| 1847 | GSE12845\_IGD\_POS\_BLOOD\_VS\_NAIVE\_TONSIL\_BCELL\_DN |  | 35 | -0.15 | -0.86 | 0.633 | 0.815 | 1.000 | 430 | tags=46%, list=30%, signal=64% |
| 1848 | GSE23114\_WT\_VS\_SLE2C1\_MOUSE\_PERITONEAL\_CAVITY\_B1A\_BCELL\_UP |  | 15 | -0.21 | -0.86 | 0.623 | 0.814 | 1.000 | 482 | tags=53%, list=34%, signal=80% |
| 1849 | GSE17721\_CTRL\_VS\_PAM3CSK4\_8H\_BMDC\_UP |  | 20 | -0.19 | -0.86 | 0.614 | 0.814 | 1.000 | 376 | tags=40%, list=27%, signal=54% |
| 1850 | GSE15330\_GRANULOCYTE\_MONOCYTE\_PROGENITOR\_VS\_PRO\_BCELL\_DN |  | 18 | -0.20 | -0.86 | 0.630 | 0.814 | 1.000 | 1139 | tags=100%, list=81%, signal=512% |
| 1851 | GSE22025\_UNTREATED\_VS\_TGFB1\_AND\_PROGESTERONE\_TREATED\_CD4\_TCELL\_UP |  | 20 | -0.19 | -0.86 | 0.628 | 0.814 | 1.000 | 326 | tags=35%, list=23%, signal=45% |
| 1852 | GSE11864\_CSF1\_IFNG\_VS\_CSF1\_PAM3CYS\_IN\_MAC\_DN |  | 30 | -0.16 | -0.86 | 0.633 | 0.814 | 1.000 | 213 | tags=23%, list=15%, signal=27% |
| 1853 | GSE27786\_LSK\_VS\_NKCELL\_UP |  | 21 | -0.18 | -0.86 | 0.629 | 0.814 | 1.000 | 160 | tags=19%, list=11%, signal=21% |
| 1854 | GSE21063\_WT\_VS\_NFATC1\_KO\_BCELL\_UP |  | 18 | -0.20 | -0.86 | 0.646 | 0.814 | 1.000 | 503 | tags=56%, list=36%, signal=85% |
| 1855 | GSE41867\_NAIVE\_VS\_DAY15\_LCMV\_CONE13\_EFFECTOR\_CD8\_TCELL\_DN |  | 21 | -0.18 | -0.86 | 0.659 | 0.815 | 1.000 | 164 | tags=24%, list=12%, signal=27% |
| 1856 | GSE6681\_DELETED\_FOXP3\_VS\_WT\_TREG\_DN |  | 29 | -0.17 | -0.86 | 0.649 | 0.815 | 1.000 | 172 | tags=21%, list=12%, signal=23% |
| 1857 | GSE32533\_WT\_VS\_MIR17\_OVEREXPRESS\_ACT\_CD4\_TCELL\_DN |  | 16 | -0.21 | -0.86 | 0.588 | 0.815 | 1.000 | 184 | tags=25%, list=13%, signal=28% |
| 1858 | GSE25123\_IL4\_VS\_IL4\_AND\_ROSIGLITAZONE\_STIM\_MACROPHAGE\_DAY10\_UP |  | 15 | -0.21 | -0.86 | 0.601 | 0.817 | 1.000 | 360 | tags=47%, list=26%, signal=62% |
| 1859 | GSE40441\_NRP1\_POS\_INDUCED\_TREG\_VS\_NRP1\_NEG\_NATURAL\_TREG\_DN |  | 20 | -0.19 | -0.86 | 0.612 | 0.817 | 1.000 | 159 | tags=20%, list=11%, signal=22% |
| 1860 | GSE17721\_PAM3CSK4\_VS\_GADIQUIMOD\_1H\_BMDC\_DN |  | 19 | -0.19 | -0.86 | 0.638 | 0.817 | 1.000 | 145 | tags=21%, list=10%, signal=23% |
| 1861 | GSE17721\_LPS\_VS\_CPG\_2H\_BMDC\_UP |  | 27 | -0.17 | -0.86 | 0.640 | 0.820 | 1.000 | 124 | tags=19%, list=9%, signal=20% |
| 1862 | GSE43955\_1H\_VS\_10H\_ACT\_CD4\_TCELL\_WITH\_TGFB\_IL6\_DN |  | 17 | -0.20 | -0.86 | 0.626 | 0.821 | 1.000 | 38 | tags=12%, list=3%, signal=12% |
| 1863 | GSE26030\_TH1\_VS\_TH17\_DAY5\_POST\_POLARIZATION\_DN |  | 27 | -0.17 | -0.86 | 0.664 | 0.821 | 1.000 | 972 | tags=89%, list=69%, signal=280% |
| 1864 | GSE2770\_IL12\_ACT\_VS\_ACT\_CD4\_TCELL\_2H\_UP |  | 24 | -0.18 | -0.85 | 0.630 | 0.823 | 1.000 | 54 | tags=13%, list=4%, signal=13% |
| 1865 | GSE22443\_NAIVE\_VS\_ACT\_AND\_IL12\_TREATED\_CD8\_TCELL\_UP |  | 17 | -0.20 | -0.85 | 0.636 | 0.822 | 1.000 | 1135 | tags=100%, list=80%, signal=505% |
| 1866 | GSE46606\_UNSTIM\_VS\_CD40L\_IL2\_IL5\_3DAY\_STIMULATED\_IRF4MID\_SORTED\_BCELL\_UP |  | 18 | -0.19 | -0.85 | 0.623 | 0.822 | 1.000 | 443 | tags=50%, list=31%, signal=72% |
| 1867 | GSE37532\_WT\_VS\_PPARG\_KO\_LN\_TCONV\_UP |  | 28 | -0.16 | -0.85 | 0.680 | 0.823 | 1.000 | 492 | tags=54%, list=35%, signal=81% |
| 1868 | GSE39820\_CTRL\_VS\_TGFBETA3\_IL6\_CD4\_TCELL\_UP |  | 29 | -0.16 | -0.85 | 0.665 | 0.824 | 1.000 | 42 | tags=10%, list=3%, signal=10% |
| 1869 | GSE7831\_1H\_VS\_4H\_INFLUENZA\_STIM\_PDC\_UP |  | 19 | -0.19 | -0.85 | 0.633 | 0.825 | 1.000 | 538 | tags=63%, list=38%, signal=101% |
| 1870 | GSE22886\_NAIVE\_CD8\_TCELL\_VS\_NKCELL\_UP |  | 16 | -0.21 | -0.85 | 0.645 | 0.825 | 1.000 | 316 | tags=38%, list=22%, signal=48% |
| 1871 | GSE5542\_UNTREATED\_VS\_IFNA\_AND\_IFNG\_TREATED\_EPITHELIAL\_CELLS\_24H\_UP |  | 19 | -0.19 | -0.85 | 0.654 | 0.827 | 1.000 | 183 | tags=26%, list=13%, signal=30% |
| 1872 | GSE16385\_MONOCYTE\_VS\_12H\_ROSIGLITAZONE\_IFNG\_TNF\_TREATED\_MACROPHAGE\_DN |  | 23 | -0.17 | -0.85 | 0.629 | 0.827 | 1.000 | 177 | tags=22%, list=13%, signal=24% |
| 1873 | GSE11961\_MARGINAL\_ZONE\_BCELL\_VS\_GERMINAL\_CENTER\_BCELL\_DAY40\_DN |  | 21 | -0.19 | -0.85 | 0.634 | 0.827 | 1.000 | 915 | tags=90%, list=65%, signal=254% |
| 1874 | GSE43863\_DAY6\_EFF\_VS\_DAY150\_MEM\_TFH\_CD4\_TCELL\_DN |  | 17 | -0.20 | -0.85 | 0.641 | 0.826 | 1.000 | 84 | tags=18%, list=6%, signal=19% |
| 1875 | GSE29949\_CD8\_NEG\_DC\_SPLEEN\_VS\_CD8\_POS\_DC\_SPLEEN\_UP |  | 15 | -0.21 | -0.85 | 0.640 | 0.828 | 1.000 | 140 | tags=20%, list=10%, signal=22% |
| 1876 | GSE7460\_FOXP3\_MUT\_VS\_WT\_ACT\_TCONV\_DN |  | 21 | -0.19 | -0.85 | 0.641 | 0.829 | 1.000 | 455 | tags=52%, list=32%, signal=76% |
| 1877 | GSE7460\_CTRL\_VS\_TGFB\_TREATED\_ACT\_FOXP3\_MUT\_TCONV\_UP |  | 21 | -0.18 | -0.85 | 0.634 | 0.829 | 1.000 | 16 | tags=10%, list=1%, signal=9% |
| 1878 | GSE5679\_CTRL\_VS\_PPARG\_LIGAND\_ROSIGLITAZONE\_AND\_RARA\_AGONIST\_AM580\_TREATED\_DC\_UP |  | 21 | -0.18 | -0.85 | 0.651 | 0.829 | 1.000 | 286 | tags=33%, list=20%, signal=41% |
| 1879 | GSE43955\_1H\_VS\_20H\_ACT\_CD4\_TCELL\_DN |  | 24 | -0.18 | -0.85 | 0.648 | 0.828 | 1.000 | 571 | tags=63%, list=40%, signal=103% |
| 1880 | GSE17721\_LPS\_VS\_PAM3CSK4\_8H\_BMDC\_DN |  | 18 | -0.19 | -0.85 | 0.642 | 0.829 | 1.000 | 830 | tags=83%, list=59%, signal=200% |
| 1881 | GSE16385\_MONOCYTE\_VS\_12H\_ROSIGLITAZONE\_IFNG\_TNF\_TREATED\_MACROPHAGE\_UP |  | 18 | -0.19 | -0.85 | 0.657 | 0.829 | 1.000 | 949 | tags=89%, list=67%, signal=268% |
| 1882 | GSE3203\_UNTREATED\_VS\_IFNB\_TREATED\_LN\_BCELL\_UP |  | 19 | -0.19 | -0.85 | 0.663 | 0.829 | 1.000 | 562 | tags=63%, list=40%, signal=104% |
| 1883 | GSE2770\_UNTREATED\_VS\_IL4\_TREATED\_ACT\_CD4\_TCELL\_6H\_DN |  | 16 | -0.20 | -0.85 | 0.672 | 0.829 | 1.000 | 165 | tags=25%, list=12%, signal=28% |
| 1884 | GSE21063\_3H\_VS\_16H\_ANTI\_IGM\_STIM\_BCELL\_UP |  | 15 | -0.21 | -0.85 | 0.682 | 0.829 | 1.000 | 846 | tags=87%, list=60%, signal=214% |
| 1885 | GSE20198\_UNTREATED\_VS\_IL12\_TREATED\_ACT\_CD4\_TCELL\_DN |  | 23 | -0.18 | -0.85 | 0.626 | 0.829 | 1.000 | 357 | tags=39%, list=25%, signal=52% |
| 1886 | GSE40274\_FOXP3\_VS\_FOXP3\_AND\_GATA1\_TRANSDUCED\_ACTIVATED\_CD4\_TCELL\_DN |  | 24 | -0.17 | -0.85 | 0.656 | 0.828 | 1.000 | 469 | tags=50%, list=33%, signal=74% |
| 1887 | GSE46606\_UNSTIM\_VS\_CD40L\_IL2\_IL5\_DAY3\_STIMULATED\_BCELL\_UP |  | 18 | -0.19 | -0.85 | 0.640 | 0.828 | 1.000 | 220 | tags=28%, list=16%, signal=32% |
| 1888 | GSE21063\_CTRL\_VS\_ANTI\_IGM\_STIM\_BCELL\_3H\_UP |  | 15 | -0.20 | -0.84 | 0.674 | 0.829 | 1.000 | 1126 | tags=100%, list=80%, signal=490% |
| 1889 | GSE27786\_LIN\_NEG\_VS\_CD8\_TCELL\_UP |  | 20 | -0.18 | -0.84 | 0.648 | 0.830 | 1.000 | 78 | tags=15%, list=6%, signal=16% |
| 1890 | GSE5099\_CLASSICAL\_M1\_VS\_ALTERNATIVE\_M2\_MACROPHAGE\_DN |  | 16 | -0.20 | -0.84 | 0.641 | 0.830 | 1.000 | 295 | tags=38%, list=21%, signal=47% |
| 1891 | GSE21063\_CTRL\_VS\_ANTI\_IGM\_STIM\_BCELL\_NFATC1\_KO\_16H\_DN |  | 20 | -0.19 | -0.84 | 0.642 | 0.829 | 1.000 | 152 | tags=20%, list=11%, signal=22% |
| 1892 | GSE30971\_CTRL\_VS\_LPS\_STIM\_MACROPHAGE\_WBP7\_HET\_4H\_DN |  | 18 | -0.19 | -0.84 | 0.648 | 0.831 | 1.000 | 941 | tags=89%, list=67%, signal=263% |
| 1893 | GSE21927\_C26GM\_VS\_4T1\_TUMOR\_MONOCYTE\_BALBC\_DN |  | 22 | -0.18 | -0.84 | 0.705 | 0.831 | 1.000 | 513 | tags=55%, list=36%, signal=84% |
| 1894 | GSE3982\_MAC\_VS\_NEUTROPHIL\_DN |  | 21 | -0.18 | -0.84 | 0.658 | 0.831 | 1.000 | 528 | tags=62%, list=37%, signal=97% |
| 1895 | GSE43863\_TH1\_VS\_TFH\_MEMORY\_CD4\_TCELL\_UP |  | 22 | -0.18 | -0.84 | 0.669 | 0.831 | 1.000 | 24 | tags=9%, list=2%, signal=9% |
| 1896 | GSE13484\_UNSTIM\_VS\_12H\_YF17D\_VACCINE\_STIM\_PBMC\_DN |  | 19 | -0.19 | -0.84 | 0.665 | 0.832 | 1.000 | 549 | tags=63%, list=39%, signal=102% |
| 1897 | GSE27859\_CD11C\_INT\_F480\_HI\_MACROPHAGE\_VS\_CD11C\_ING\_F480\_INT\_DC\_DN |  | 17 | -0.20 | -0.84 | 0.657 | 0.832 | 1.000 | 562 | tags=71%, list=40%, signal=116% |
| 1898 | GSE6674\_ANTI\_IGM\_VS\_ANTI\_IGM\_AND\_CPG\_STIM\_BCELL\_DN |  | 35 | -0.15 | -0.84 | 0.672 | 0.833 | 1.000 | 1088 | tags=94%, list=77%, signal=402% |
| 1899 | GSE22886\_IGA\_VS\_IGM\_MEMORY\_BCELL\_UP |  | 17 | -0.20 | -0.84 | 0.663 | 0.834 | 1.000 | 451 | tags=47%, list=32%, signal=68% |
| 1900 | GSE46606\_IRF4MID\_VS\_WT\_CD40L\_IL2\_IL5\_DAY1\_STIMULATED\_BCELL\_DN |  | 22 | -0.18 | -0.84 | 0.667 | 0.834 | 1.000 | 393 | tags=45%, list=28%, signal=62% |
| 1901 | GSE23568\_ID3\_KO\_VS\_WT\_CD8\_TCELL\_UP |  | 22 | -0.18 | -0.84 | 0.645 | 0.836 | 1.000 | 114 | tags=14%, list=8%, signal=15% |
| 1902 | GSE40184\_HEALTHY\_VS\_HCV\_INFECTED\_DONOR\_PBMC\_UP |  | 21 | -0.18 | -0.84 | 0.668 | 0.835 | 1.000 | 194 | tags=24%, list=14%, signal=27% |
| 1903 | GSE5589\_LPS\_VS\_LPS\_AND\_IL10\_STIM\_IL10\_KO\_MACROPHAGE\_180MIN\_DN |  | 15 | -0.21 | -0.84 | 0.631 | 0.836 | 1.000 | 66 | tags=13%, list=5%, signal=14% |
| 1904 | GSE32986\_UNSTIM\_VS\_CURDLAN\_LOWDOSE\_STIM\_DC\_UP |  | 27 | -0.16 | -0.84 | 0.673 | 0.837 | 1.000 | 278 | tags=26%, list=20%, signal=32% |
| 1905 | GSE17721\_CTRL\_VS\_GARDIQUIMOD\_12H\_BMDC\_UP |  | 15 | -0.21 | -0.84 | 0.649 | 0.837 | 1.000 | 225 | tags=27%, list=16%, signal=31% |
| 1906 | GSE4142\_GC\_BCELL\_VS\_MEMORY\_BCELL\_UP |  | 19 | -0.19 | -0.84 | 0.681 | 0.837 | 1.000 | 20 | tags=11%, list=1%, signal=11% |
| 1907 | GSE13547\_WT\_VS\_ZFX\_KO\_BCELL\_ANTI\_IGM\_STIM\_2H\_DN |  | 20 | -0.19 | -0.84 | 0.673 | 0.837 | 1.000 | 182 | tags=20%, list=13%, signal=23% |
| 1908 | GSE10239\_MEMORY\_VS\_KLRG1INT\_EFF\_CD8\_TCELL\_DN |  | 27 | -0.16 | -0.83 | 0.700 | 0.838 | 1.000 | 480 | tags=48%, list=34%, signal=72% |
| 1909 | GSE12845\_NAIVE\_VS\_PRE\_GC\_TONSIL\_BCELL\_UP |  | 26 | -0.17 | -0.83 | 0.693 | 0.838 | 1.000 | 557 | tags=62%, list=39%, signal=100% |
| 1910 | GSE46606\_IRF4HIGH\_VS\_IRF4MID\_CD40L\_IL2\_IL5\_DAY3\_STIMULATED\_BCELL\_DN |  | 21 | -0.18 | -0.83 | 0.651 | 0.839 | 1.000 | 267 | tags=33%, list=19%, signal=41% |
| 1911 | GSE31082\_CD4\_VS\_CD8\_SP\_THYMOCYTE\_UP |  | 23 | -0.17 | -0.83 | 0.654 | 0.839 | 1.000 | 791 | tags=83%, list=56%, signal=185% |
| 1912 | GSE360\_T\_GONDII\_VS\_B\_MALAYI\_LOW\_DOSE\_DC\_DN |  | 19 | -0.19 | -0.83 | 0.675 | 0.838 | 1.000 | 326 | tags=37%, list=23%, signal=47% |
| 1913 | GSE45365\_NK\_CELL\_VS\_CD8\_TCELL\_DN |  | 15 | -0.20 | -0.83 | 0.640 | 0.840 | 1.000 | 1134 | tags=100%, list=80%, signal=504% |
| 1914 | GSE22935\_WT\_VS\_MYD88\_KO\_MACROPHAGE\_24H\_MBOVIS\_BCG\_STIM\_UP |  | 17 | -0.20 | -0.83 | 0.678 | 0.840 | 1.000 | 827 | tags=82%, list=59%, signal=197% |
| 1915 | GSE37301\_PRO\_BCELL\_VS\_RAG2\_KO\_NK\_CELL\_UP |  | 16 | -0.20 | -0.83 | 0.667 | 0.840 | 1.000 | 251 | tags=31%, list=18%, signal=38% |
| 1916 | GSE22886\_NEUTROPHIL\_VS\_MONOCYTE\_UP |  | 15 | -0.20 | -0.83 | 0.682 | 0.840 | 1.000 | 402 | tags=47%, list=28%, signal=65% |
| 1917 | GSE12392\_WT\_VS\_IFNB\_KO\_CD8A\_NEG\_SPLEEN\_DC\_DN |  | 18 | -0.19 | -0.83 | 0.682 | 0.840 | 1.000 | 897 | tags=89%, list=64%, signal=241% |
| 1918 | GSE15324\_ELF4\_KO\_VS\_WT\_ACTIVATED\_CD8\_TCELL\_UP |  | 25 | -0.17 | -0.83 | 0.664 | 0.840 | 1.000 | 393 | tags=40%, list=28%, signal=54% |
| 1919 | GSE9006\_1MONTH\_VS\_4MONTH\_POST\_TYPE\_1\_DIABETES\_DX\_PBMC\_UP |  | 27 | -0.16 | -0.83 | 0.687 | 0.840 | 1.000 | 588 | tags=63%, list=42%, signal=106% |
| 1920 | GSE25088\_IL4\_VS\_IL4\_AND\_ROSIGLITAZONE\_STIM\_MACROPHAGE\_DAY10\_UP |  | 16 | -0.20 | -0.83 | 0.683 | 0.840 | 1.000 | 893 | tags=88%, list=63%, signal=236% |
| 1921 | GSE17186\_NAIVE\_VS\_CD21LOW\_TRANSITIONAL\_BCELL\_UP |  | 18 | -0.19 | -0.83 | 0.670 | 0.841 | 1.000 | 620 | tags=72%, list=44%, signal=127% |
| 1922 | GSE27786\_LSK\_VS\_LIN\_NEG\_CELL\_UP |  | 18 | -0.19 | -0.83 | 0.691 | 0.841 | 1.000 | 357 | tags=44%, list=25%, signal=59% |
| 1923 | GSE27786\_NKCELL\_VS\_NKTCELL\_UP |  | 17 | -0.19 | -0.83 | 0.681 | 0.841 | 1.000 | 215 | tags=24%, list=15%, signal=27% |
| 1924 | GSE37534\_UNTREATED\_VS\_ROSIGLITAZONE\_TREATED\_CD4\_TCELL\_PPARG1\_AND\_FOXP3\_TRASDUCED\_UP |  | 19 | -0.19 | -0.83 | 0.677 | 0.842 | 1.000 | 207 | tags=26%, list=15%, signal=30% |
| 1925 | GSE10239\_NAIVE\_VS\_MEMORY\_CD8\_TCELL\_UP |  | 25 | -0.16 | -0.83 | 0.689 | 0.843 | 1.000 | 444 | tags=48%, list=31%, signal=69% |
| 1926 | GSE23568\_CTRL\_VS\_ID3\_TRANSDUCED\_CD8\_TCELL\_UP |  | 18 | -0.19 | -0.83 | 0.681 | 0.843 | 1.000 | 1044 | tags=94%, list=74%, signal=358% |
| 1927 | GSE26343\_WT\_VS\_NFAT5\_KO\_MACROPHAGE\_DN |  | 19 | -0.18 | -0.83 | 0.702 | 0.843 | 1.000 | 167 | tags=21%, list=12%, signal=24% |
| 1928 | GSE17721\_POLYIC\_VS\_GARDIQUIMOD\_16H\_BMDC\_DN |  | 21 | -0.17 | -0.83 | 0.699 | 0.843 | 1.000 | 1169 | tags=100%, list=83%, signal=574% |
| 1929 | GSE27786\_CD4\_TCELL\_VS\_ERYTHTROBLAST\_DN |  | 15 | -0.21 | -0.83 | 0.692 | 0.843 | 1.000 | 176 | tags=27%, list=12%, signal=30% |
| 1930 | GSE17721\_0.5H\_VS\_8H\_PAM3CSK4\_BMDC\_UP |  | 20 | -0.18 | -0.83 | 0.671 | 0.844 | 1.000 | 746 | tags=75%, list=53%, signal=157% |
| 1931 | GSE27786\_LIN\_NEG\_VS\_CD8\_TCELL\_DN |  | 18 | -0.18 | -0.83 | 0.690 | 0.843 | 1.000 | 80 | tags=17%, list=6%, signal=17% |
| 1932 | GSE14308\_NAIVE\_CD4\_TCELL\_VS\_NATURAL\_TREG\_UP |  | 20 | -0.18 | -0.83 | 0.671 | 0.843 | 1.000 | 147 | tags=20%, list=10%, signal=22% |
| 1933 | GSE36527\_CD69\_NEG\_VS\_POS\_TREG\_CD62L\_LOS\_KLRG1\_NEG\_DN |  | 22 | -0.17 | -0.82 | 0.689 | 0.844 | 1.000 | 173 | tags=23%, list=12%, signal=25% |
| 1934 | GSE17721\_LPS\_VS\_CPG\_16H\_BMDC\_UP |  | 17 | -0.19 | -0.82 | 0.674 | 0.843 | 1.000 | 293 | tags=29%, list=21%, signal=37% |
| 1935 | GSE19198\_CTRL\_VS\_IL21\_TREATED\_TCELL\_24H\_UP |  | 27 | -0.16 | -0.82 | 0.693 | 0.844 | 1.000 | 596 | tags=67%, list=42%, signal=113% |
| 1936 | GSE17721\_CTRL\_VS\_LPS\_8H\_BMDC\_DN |  | 27 | -0.16 | -0.82 | 0.673 | 0.843 | 1.000 | 209 | tags=22%, list=15%, signal=26% |
| 1937 | GSE7460\_CTRL\_VS\_TGFB\_TREATED\_ACT\_TCONV\_DN |  | 21 | -0.17 | -0.82 | 0.680 | 0.843 | 1.000 | 245 | tags=29%, list=17%, signal=34% |
| 1938 | GSE17721\_PAM3CSK4\_VS\_GADIQUIMOD\_4H\_BMDC\_DN |  | 20 | -0.18 | -0.82 | 0.667 | 0.843 | 1.000 | 489 | tags=55%, list=35%, signal=83% |
| 1939 | GSE15930\_NAIVE\_VS\_72H\_IN\_VITRO\_STIM\_TRICHOSTATINA\_CD8\_TCELL\_DN |  | 18 | -0.19 | -0.82 | 0.683 | 0.844 | 1.000 | 104 | tags=17%, list=7%, signal=18% |
| 1940 | GSE41867\_DAY6\_EFFECTOR\_VS\_DAY30\_EXHAUSTED\_CD8\_TCELL\_LCMV\_CLONE13\_UP |  | 17 | -0.20 | -0.82 | 0.666 | 0.844 | 1.000 | 140 | tags=18%, list=10%, signal=19% |
| 1941 | GSE1432\_CTRL\_VS\_IFNG\_1H\_MICROGLIA\_DN |  | 17 | -0.19 | -0.82 | 0.681 | 0.846 | 1.000 | 528 | tags=59%, list=37%, signal=93% |
| 1942 | GSE21670\_STAT3\_KO\_VS\_WT\_CD4\_TCELL\_TGFB\_TREATED\_UP |  | 19 | -0.18 | -0.82 | 0.684 | 0.846 | 1.000 | 1159 | tags=100%, list=82%, signal=552% |
| 1943 | GSE46242\_TH1\_VS\_ANERGIC\_TH1\_CD4\_TCELL\_WITH\_EGR2\_DELETED\_UP |  | 27 | -0.16 | -0.82 | 0.706 | 0.848 | 1.000 | 534 | tags=56%, list=38%, signal=88% |
| 1944 | GSE14308\_TH2\_VS\_INDUCED\_TREG\_DN |  | 23 | -0.17 | -0.82 | 0.698 | 0.847 | 1.000 | 1093 | tags=96%, list=77%, signal=418% |
| 1945 | GSE29164\_UNTREATED\_VS\_CD8\_TCELL\_AND\_IL12\_TREATED\_MELANOMA\_DAY3\_DN |  | 21 | -0.18 | -0.82 | 0.698 | 0.847 | 1.000 | 448 | tags=48%, list=32%, signal=69% |
| 1946 | GSE11961\_MARGINAL\_ZONE\_BCELL\_VS\_GERMINAL\_CENTER\_BCELL\_DAY40\_UP |  | 19 | -0.18 | -0.82 | 0.692 | 0.847 | 1.000 | 31 | tags=11%, list=2%, signal=11% |
| 1947 | GSE41176\_WT\_VS\_TAK1\_KO\_ANTI\_IGM\_STIM\_BCELL\_6H\_UP |  | 20 | -0.18 | -0.82 | 0.682 | 0.846 | 1.000 | 161 | tags=20%, list=11%, signal=22% |
| 1948 | GSE37534\_UNTREATED\_VS\_PIOGLITAZONE\_TREATED\_CD4\_TCELL\_PPARG1\_AND\_FOXP3\_TRASDUCED\_UP |  | 23 | -0.17 | -0.82 | 0.730 | 0.849 | 1.000 | 207 | tags=26%, list=15%, signal=30% |
| 1949 | GSE19772\_CTRL\_VS\_HCMV\_INF\_MONOCYTES\_AND\_PI3K\_INHIBITION\_DN |  | 15 | -0.20 | -0.82 | 0.660 | 0.852 | 1.000 | 122 | tags=20%, list=9%, signal=22% |
| 1950 | GSE38681\_WT\_VS\_LYL1\_KO\_LYMPHOID\_PRIMED\_MULTIPOTENT\_PROGENITOR\_DN |  | 18 | -0.19 | -0.81 | 0.722 | 0.853 | 1.000 | 544 | tags=61%, list=39%, signal=98% |
| 1951 | GSE17974\_IL4\_AND\_ANTI\_IL12\_VS\_UNTREATED\_72H\_ACT\_CD4\_TCELL\_UP |  | 18 | -0.18 | -0.81 | 0.715 | 0.853 | 1.000 | 436 | tags=44%, list=31%, signal=63% |
| 1952 | GSE35543\_IN\_VIVO\_NTREG\_VS\_CONVERTED\_EX\_ITREG\_UP |  | 22 | -0.17 | -0.81 | 0.678 | 0.853 | 1.000 | 792 | tags=77%, list=56%, signal=173% |
| 1953 | GSE11057\_CD4\_EFF\_MEM\_VS\_PBMC\_UP |  | 25 | -0.17 | -0.81 | 0.697 | 0.853 | 1.000 | 184 | tags=24%, list=13%, signal=27% |
| 1954 | GSE40274\_CTRL\_VS\_FOXP3\_AND\_EOS\_TRANSDUCED\_ACTIVATED\_CD4\_TCELL\_DN |  | 21 | -0.18 | -0.81 | 0.683 | 0.853 | 1.000 | 95 | tags=14%, list=7%, signal=15% |
| 1955 | GSE22886\_DAY1\_VS\_DAY7\_MONOCYTE\_IN\_CULTURE\_DN |  | 25 | -0.16 | -0.81 | 0.698 | 0.854 | 1.000 | 922 | tags=88%, list=65%, signal=249% |
| 1956 | GSE17186\_MEMORY\_VS\_NAIVE\_BCELL\_DN |  | 18 | -0.19 | -0.81 | 0.716 | 0.854 | 1.000 | 543 | tags=61%, list=38%, signal=98% |
| 1957 | GSE36078\_WT\_VS\_IL1R\_KO\_LUNG\_DC\_DN |  | 22 | -0.17 | -0.81 | 0.694 | 0.854 | 1.000 | 1178 | tags=100%, list=83%, signal=596% |
| 1958 | GSE18791\_CTRL\_VS\_NEWCASTLE\_VIRUS\_DC\_8H\_DN |  | 24 | -0.17 | -0.81 | 0.681 | 0.854 | 1.000 | 563 | tags=58%, list=40%, signal=95% |
| 1959 | GSE22282\_HYPOXIA\_VS\_NORMOXIA\_MYELOID\_DC\_UP |  | 24 | -0.16 | -0.81 | 0.714 | 0.855 | 1.000 | 217 | tags=25%, list=15%, signal=29% |
| 1960 | GSE360\_L\_MAJOR\_VS\_T\_GONDII\_MAC\_DN |  | 28 | -0.15 | -0.81 | 0.713 | 0.854 | 1.000 | 1048 | tags=93%, list=74%, signal=354% |
| 1961 | GSE36009\_UNSTIM\_VS\_LPS\_STIM\_DC\_UP |  | 19 | -0.18 | -0.81 | 0.667 | 0.854 | 1.000 | 412 | tags=47%, list=29%, signal=66% |
| 1962 | GSE1925\_CTRL\_VS\_IFNG\_PRIMED\_MACROPHAGE\_DN |  | 19 | -0.18 | -0.81 | 0.701 | 0.854 | 1.000 | 1057 | tags=95%, list=75%, signal=373% |
| 1963 | GSE26030\_UNSTIM\_VS\_RESTIM\_TH1\_DAY15\_POST\_POLARIZATION\_DN |  | 23 | -0.17 | -0.81 | 0.709 | 0.854 | 1.000 | 437 | tags=48%, list=31%, signal=68% |
| 1964 | GSE24634\_TEFF\_VS\_TCONV\_DAY7\_IN\_CULTURE\_DN |  | 21 | -0.18 | -0.81 | 0.698 | 0.856 | 1.000 | 562 | tags=67%, list=40%, signal=109% |
| 1965 | GSE40666\_UNTREATED\_VS\_IFNA\_STIM\_STAT1\_KO\_CD8\_TCELL\_90MIN\_DN |  | 19 | -0.18 | -0.81 | 0.692 | 0.855 | 1.000 | 1057 | tags=95%, list=75%, signal=373% |
| 1966 | GSE5099\_DAY3\_VS\_DAY7\_MCSF\_TREATED\_MACROPHAGE\_DN |  | 15 | -0.19 | -0.81 | 0.703 | 0.855 | 1.000 | 204 | tags=27%, list=14%, signal=31% |
| 1967 | GSE34006\_A2AR\_KO\_VS\_A2AR\_AGONIST\_TREATED\_TREG\_DN |  | 22 | -0.17 | -0.81 | 0.726 | 0.856 | 1.000 | 429 | tags=45%, list=30%, signal=64% |
| 1968 | GSE43956\_WT\_VS\_SGK1\_KO\_TH17\_DIFFERENTIATED\_CD4\_TCELL\_UP |  | 15 | -0.19 | -0.81 | 0.709 | 0.857 | 1.000 | 388 | tags=47%, list=27%, signal=64% |
| 1969 | GSE7348\_UNSTIM\_VS\_LPS\_STIM\_MACROPHAGE\_UP |  | 21 | -0.18 | -0.81 | 0.685 | 0.857 | 1.000 | 171 | tags=24%, list=12%, signal=27% |
| 1970 | GSE43955\_TH0\_VS\_TGFB\_IL6\_TH17\_ACT\_CD4\_TCELL\_10H\_UP |  | 23 | -0.17 | -0.81 | 0.706 | 0.858 | 1.000 | 505 | tags=57%, list=36%, signal=87% |
| 1971 | GSE45365\_CD8A\_DC\_VS\_CD11B\_DC\_IFNAR\_KO\_DN |  | 19 | -0.17 | -0.81 | 0.699 | 0.858 | 1.000 | 137 | tags=16%, list=10%, signal=17% |
| 1972 | GSE46606\_DAY1\_VS\_DAY3\_CD40L\_IL2\_IL5\_STIMULATED\_IRF4HIGH\_BCELL\_UP |  | 25 | -0.16 | -0.81 | 0.716 | 0.858 | 1.000 | 173 | tags=20%, list=12%, signal=22% |
| 1973 | GSE43863\_TH1\_VS\_LY6C\_INT\_CXCR5POS\_EFFECTOR\_CD4\_TCELL\_DN |  | 24 | -0.17 | -0.81 | 0.751 | 0.858 | 1.000 | 436 | tags=46%, list=31%, signal=65% |
| 1974 | GSE2770\_UNTREATED\_VS\_TGFB\_AND\_IL12\_TREATED\_ACT\_CD4\_TCELL\_48H\_DN |  | 21 | -0.17 | -0.81 | 0.725 | 0.857 | 1.000 | 1078 | tags=95%, list=76%, signal=398% |
| 1975 | GSE5589\_LPS\_AND\_IL10\_VS\_LPS\_AND\_IL6\_STIM\_IL10\_KO\_MACROPHAGE\_45MIN\_DN |  | 15 | -0.20 | -0.81 | 0.683 | 0.857 | 1.000 | 327 | tags=40%, list=23%, signal=52% |
| 1976 | GSE3982\_BCELL\_VS\_EFF\_MEMORY\_CD4\_TCELL\_DN |  | 15 | -0.20 | -0.80 | 0.672 | 0.859 | 1.000 | 212 | tags=27%, list=15%, signal=31% |
| 1977 | GSE41867\_DAY15\_EFFECTOR\_VS\_DAY30\_EXHAUSTED\_CD8\_TCELL\_LCMV\_CLONE13\_UP |  | 18 | -0.19 | -0.80 | 0.677 | 0.860 | 1.000 | 140 | tags=17%, list=10%, signal=18% |
| 1978 | GSE33425\_CD8\_ALPHAALPHA\_VS\_ALPHABETA\_CD161\_HIGH\_TCELL\_DN |  | 20 | -0.18 | -0.80 | 0.728 | 0.861 | 1.000 | 524 | tags=60%, list=37%, signal=94% |
| 1979 | GSE21380\_TFH\_VS\_GERMINAL\_CENTER\_TFH\_CD4\_TCELL\_DN |  | 15 | -0.20 | -0.80 | 0.733 | 0.861 | 1.000 | 1135 | tags=100%, list=80%, signal=506% |
| 1980 | GSE6259\_FLT3L\_INDUCED\_DEC205\_POS\_DC\_VS\_CD4\_TCELL\_UP |  | 17 | -0.19 | -0.80 | 0.705 | 0.861 | 1.000 | 124 | tags=18%, list=9%, signal=19% |
| 1981 | GSE40685\_NAIVE\_CD4\_TCELL\_VS\_FOXP3\_KO\_TREG\_PRECURSOR\_UP |  | 21 | -0.17 | -0.80 | 0.730 | 0.861 | 1.000 | 599 | tags=67%, list=42%, signal=114% |
| 1982 | GSE17721\_POLYIC\_VS\_GARDIQUIMOD\_2H\_BMDC\_DN |  | 20 | -0.17 | -0.80 | 0.714 | 0.862 | 1.000 | 210 | tags=25%, list=15%, signal=29% |
| 1983 | GSE1460\_DP\_THYMOCYTE\_VS\_THYMIC\_STROMAL\_CELL\_UP |  | 16 | -0.20 | -0.80 | 0.710 | 0.862 | 1.000 | 41 | tags=13%, list=3%, signal=13% |
| 1984 | GSE27786\_LIN\_NEG\_VS\_NKTCELL\_UP |  | 15 | -0.19 | -0.80 | 0.721 | 0.863 | 1.000 | 1143 | tags=100%, list=81%, signal=521% |
| 1985 | GSE43957\_UNTREATED\_VS\_NACL\_TREATED\_ANTI\_CD3\_CD28\_STIM\_CD4\_TCELL\_DN |  | 31 | -0.15 | -0.80 | 0.739 | 0.865 | 1.000 | 625 | tags=68%, list=44%, signal=119% |
| 1986 | GSE42088\_UNINF\_VS\_LEISHMANIA\_INF\_DC\_4H\_UP |  | 26 | -0.16 | -0.80 | 0.725 | 0.865 | 1.000 | 254 | tags=27%, list=18%, signal=32% |
| 1987 | GSE5463\_CTRL\_VS\_DEXAMETHASONE\_TREATED\_THYMOCYTE\_UP |  | 15 | -0.20 | -0.80 | 0.696 | 0.865 | 1.000 | 419 | tags=47%, list=30%, signal=66% |
| 1988 | GSE17721\_CTRL\_VS\_CPG\_12H\_BMDC\_UP |  | 16 | -0.19 | -0.80 | 0.729 | 0.865 | 1.000 | 355 | tags=38%, list=25%, signal=50% |
| 1989 | GSE9601\_UNTREATED\_VS\_PI3K\_INHIBITOR\_TREATED\_HCMV\_INF\_MONOCYTE\_DN |  | 15 | -0.19 | -0.80 | 0.723 | 0.865 | 1.000 | 341 | tags=40%, list=24%, signal=52% |
| 1990 | GSE22886\_UNSTIM\_VS\_STIM\_MEMORY\_TCELL\_DN |  | 23 | -0.17 | -0.80 | 0.730 | 0.864 | 1.000 | 902 | tags=87%, list=64%, signal=237% |
| 1991 | GSE5455\_EX\_VIVO\_VS\_POST\_24H\_INCUBATION\_MONOCYTES\_FROM\_TUMOR\_BEARING\_MOUSE\_DN |  | 22 | -0.17 | -0.80 | 0.751 | 0.864 | 1.000 | 75 | tags=14%, list=5%, signal=14% |
| 1992 | GSE43955\_TGFB\_IL6\_VS\_TGFB\_IL6\_IL23\_TH17\_ACT\_CD4\_TCELL\_60H\_DN |  | 15 | -0.19 | -0.80 | 0.727 | 0.865 | 1.000 | 198 | tags=27%, list=14%, signal=31% |
| 1993 | GSE19941\_UNSTIM\_VS\_LPS\_STIM\_IL10\_KO\_NFKBP50\_KO\_MACROPHAGE\_UP |  | 15 | -0.19 | -0.79 | 0.709 | 0.866 | 1.000 | 2 | tags=7%, list=0%, signal=7% |
| 1994 | GSE9650\_NAIVE\_VS\_EFF\_CD8\_TCELL\_UP |  | 19 | -0.18 | -0.79 | 0.708 | 0.866 | 1.000 | 78 | tags=16%, list=6%, signal=16% |
| 1995 | GSE5455\_HEALTHY\_VS\_TUMOR\_BEARING\_MOUSE\_SPLEEN\_MONOCYTE\_24H\_INCUBATION\_DN |  | 18 | -0.18 | -0.79 | 0.709 | 0.866 | 1.000 | 582 | tags=61%, list=41%, signal=103% |
| 1996 | GSE6259\_33D1\_POS\_DC\_VS\_CD4\_TCELL\_UP |  | 18 | -0.18 | -0.79 | 0.708 | 0.866 | 1.000 | 411 | tags=50%, list=29%, signal=70% |
| 1997 | GSE5455\_EX\_VIVO\_VS\_POST\_24H\_INCUBATION\_MONOCYTES\_FROM\_TUMOR\_BEARING\_MOUSE\_UP |  | 15 | -0.19 | -0.79 | 0.703 | 0.866 | 1.000 | 1140 | tags=100%, list=81%, signal=515% |
| 1998 | GSE360\_CTRL\_VS\_B\_MALAYI\_HIGH\_DOSE\_DC\_DN |  | 30 | -0.15 | -0.79 | 0.737 | 0.866 | 1.000 | 1206 | tags=100%, list=85%, signal=674% |
| 1999 | GSE360\_L\_MAJOR\_VS\_B\_MALAYI\_LOW\_DOSE\_DC\_UP |  | 23 | -0.16 | -0.79 | 0.705 | 0.866 | 1.000 | 23 | tags=9%, list=2%, signal=9% |
| 2000 | GSE339\_CD4POS\_VS\_CD8POS\_DC\_UP |  | 23 | -0.17 | -0.79 | 0.737 | 0.865 | 1.000 | 70 | tags=13%, list=5%, signal=14% |
| 2001 | GSE27786\_NEUTROPHIL\_VS\_MONO\_MAC\_DN |  | 22 | -0.17 | -0.79 | 0.728 | 0.870 | 1.000 | 575 | tags=59%, list=41%, signal=98% |
| 2002 | GSE16385\_IFNG\_TNF\_VS\_UNSTIM\_MACROPHAGE\_ROSIGLITAZONE\_TREATED\_DN |  | 21 | -0.17 | -0.79 | 0.729 | 0.870 | 1.000 | 415 | tags=43%, list=29%, signal=60% |
| 2003 | GSE45365\_NK\_CELL\_VS\_CD8\_TCELL\_MCMV\_INFECTION\_UP |  | 21 | -0.17 | -0.79 | 0.752 | 0.870 | 1.000 | 988 | tags=90%, list=70%, signal=297% |
| 2004 | GSE3982\_MAST\_CELL\_VS\_BCELL\_DN |  | 21 | -0.17 | -0.79 | 0.708 | 0.871 | 1.000 | 511 | tags=52%, list=36%, signal=81% |
| 2005 | GSE22432\_MULTIPOTENT\_PROGENITOR\_VS\_PDC\_UP |  | 17 | -0.18 | -0.79 | 0.737 | 0.871 | 1.000 | 362 | tags=41%, list=26%, signal=55% |
| 2006 | GSE6269\_FLU\_VS\_STREP\_PNEUMO\_INF\_PBMC\_UP |  | 18 | -0.18 | -0.79 | 0.727 | 0.871 | 1.000 | 926 | tags=89%, list=66%, signal=255% |
| 2007 | GSE1432\_1H\_VS\_24H\_IFNG\_MICROGLIA\_DN |  | 28 | -0.15 | -0.79 | 0.726 | 0.872 | 1.000 | 512 | tags=54%, list=36%, signal=82% |
| 2008 | GSE41867\_MEMORY\_VS\_EXHAUSTED\_CD8\_TCELL\_DAY30\_LCMV\_UP |  | 20 | -0.17 | -0.79 | 0.731 | 0.871 | 1.000 | 155 | tags=20%, list=11%, signal=22% |
| 2009 | GSE1460\_CD4\_THYMOCYTE\_VS\_THYMIC\_STROMAL\_CELL\_DN |  | 19 | -0.17 | -0.79 | 0.732 | 0.871 | 1.000 | 238 | tags=26%, list=17%, signal=31% |
| 2010 | GSE25123\_CTRL\_VS\_IL4\_AND\_ROSIGLITAZONE\_STIM\_PPARG\_KO\_MACROPHAGE\_UP |  | 22 | -0.16 | -0.79 | 0.728 | 0.871 | 1.000 | 242 | tags=23%, list=17%, signal=27% |
| 2011 | GSE11057\_CD4\_CENT\_MEM\_VS\_PBMC\_UP |  | 27 | -0.15 | -0.79 | 0.729 | 0.871 | 1.000 | 184 | tags=22%, list=13%, signal=25% |
| 2012 | GSE46242\_TH1\_VS\_ANERGIC\_TH1\_CD4\_TCELL\_DN |  | 17 | -0.18 | -0.79 | 0.730 | 0.871 | 1.000 | 85 | tags=18%, list=6%, signal=19% |
| 2013 | GSE3982\_BASOPHIL\_VS\_TH1\_DN |  | 25 | -0.16 | -0.78 | 0.753 | 0.873 | 1.000 | 350 | tags=32%, list=25%, signal=42% |
| 2014 | GSE46606\_UNSTIM\_VS\_CD40L\_IL2\_IL5\_1DAY\_STIMULATED\_IRF4MID\_SORTED\_BCELL\_UP |  | 24 | -0.16 | -0.78 | 0.748 | 0.873 | 1.000 | 253 | tags=25%, list=18%, signal=30% |
| 2015 | GSE33425\_CD161\_HIGH\_VS\_INT\_CD8\_TCELL\_DN |  | 20 | -0.17 | -0.78 | 0.740 | 0.873 | 1.000 | 702 | tags=75%, list=50%, signal=147% |
| 2016 | GSE360\_L\_MAJOR\_VS\_B\_MALAYI\_HIGH\_DOSE\_DC\_DN |  | 19 | -0.17 | -0.78 | 0.711 | 0.875 | 1.000 | 574 | tags=63%, list=41%, signal=105% |
| 2017 | GSE17721\_LPS\_VS\_POLYIC\_8H\_BMDC\_UP |  | 22 | -0.17 | -0.78 | 0.731 | 0.878 | 1.000 | 187 | tags=23%, list=13%, signal=26% |
| 2018 | GSE22196\_HEALTHY\_VS\_OBESE\_MOUSE\_SKIN\_GAMMADELTA\_TCELL\_UP |  | 25 | -0.16 | -0.78 | 0.746 | 0.878 | 1.000 | 160 | tags=20%, list=11%, signal=22% |
| 2019 | GSE29164\_CD8\_TCELL\_VS\_CD8\_TCELL\_AND\_IL12\_TREATED\_MELANOMA\_DAY3\_DN |  | 23 | -0.16 | -0.78 | 0.757 | 0.877 | 1.000 | 596 | tags=65%, list=42%, signal=111% |
| 2020 | GSE26290\_WT\_VS\_PDK1\_KO\_ANTI\_CD3\_AND\_IL2\_STIM\_CD8\_TCELL\_DN |  | 16 | -0.18 | -0.78 | 0.777 | 0.878 | 1.000 | 191 | tags=25%, list=14%, signal=29% |
| 2021 | GSE34179\_THPOK\_KO\_VS\_WT\_VA14I\_NKTCELL\_DN |  | 20 | -0.17 | -0.78 | 0.769 | 0.878 | 1.000 | 22 | tags=10%, list=2%, signal=10% |
| 2022 | GSE29618\_BCELL\_VS\_PDC\_DN |  | 20 | -0.17 | -0.78 | 0.723 | 0.880 | 1.000 | 249 | tags=30%, list=18%, signal=36% |
| 2023 | GSE5542\_IFNA\_VS\_IFNA\_AND\_IFNG\_TREATED\_EPITHELIAL\_CELLS\_6H\_UP |  | 21 | -0.17 | -0.78 | 0.735 | 0.880 | 1.000 | 295 | tags=33%, list=21%, signal=42% |
| 2024 | GSE17721\_PAM3CSK4\_VS\_CPG\_2H\_BMDC\_DN |  | 15 | -0.19 | -0.78 | 0.734 | 0.881 | 1.000 | 1019 | tags=93%, list=72%, signal=332% |
| 2025 | GSE15659\_NAIVE\_VS\_PTPRC\_NEG\_CD4\_TCELL\_DN |  | 15 | -0.19 | -0.78 | 0.732 | 0.881 | 1.000 | 1145 | tags=100%, list=81%, signal=525% |
| 2026 | GSE2770\_IL12\_AND\_TGFB\_ACT\_VS\_ACT\_CD4\_TCELL\_48H\_DN |  | 21 | -0.17 | -0.78 | 0.733 | 0.880 | 1.000 | 112 | tags=14%, list=8%, signal=15% |
| 2027 | GSE9509\_10MIN\_VS\_30MIN\_LPS\_STIM\_IL10\_KO\_MACROPHAGE\_DN |  | 16 | -0.18 | -0.78 | 0.749 | 0.880 | 1.000 | 4 | tags=6%, list=0%, signal=6% |
| 2028 | GSE17186\_MEMORY\_VS\_CD21HIGH\_TRANSITIONAL\_BCELL\_UP |  | 15 | -0.19 | -0.78 | 0.753 | 0.880 | 1.000 | 764 | tags=80%, list=54%, signal=173% |
| 2029 | GSE17721\_CTRL\_VS\_LPS\_4H\_BMDC\_UP |  | 21 | -0.16 | -0.78 | 0.751 | 0.880 | 1.000 | 1183 | tags=100%, list=84%, signal=610% |
| 2030 | GSE41867\_DAY6\_EFFECTOR\_VS\_DAY30\_MEMORY\_CD8\_TCELL\_LCMV\_ARMSTRONG\_DN |  | 18 | -0.18 | -0.78 | 0.753 | 0.881 | 1.000 | 360 | tags=39%, list=26%, signal=52% |
| 2031 | GSE37534\_PIOGLITAZONE\_VS\_ROSIGLITAZONE\_TREATED\_CD4\_TCELL\_PPARG1\_FOXP3\_TRANSDUCED\_DN |  | 17 | -0.18 | -0.77 | 0.714 | 0.881 | 1.000 | 247 | tags=29%, list=18%, signal=35% |
| 2032 | GSE37532\_VISCERAL\_ADIPOSE\_TISSUE\_VS\_LN\_DERIVED\_TREG\_CD4\_TCELL\_UP |  | 22 | -0.17 | -0.77 | 0.737 | 0.881 | 1.000 | 214 | tags=27%, list=15%, signal=32% |
| 2033 | GSE2770\_TGFB\_AND\_IL4\_VS\_IL4\_TREATED\_ACT\_CD4\_TCELL\_2H\_DN |  | 18 | -0.17 | -0.77 | 0.735 | 0.882 | 1.000 | 303 | tags=33%, list=21%, signal=42% |
| 2034 | GSE17721\_LPS\_VS\_CPG\_4H\_BMDC\_UP |  | 24 | -0.16 | -0.77 | 0.759 | 0.883 | 1.000 | 350 | tags=38%, list=25%, signal=49% |
| 2035 | GSE17721\_0.5H\_VS\_4H\_LPS\_BMDC\_UP |  | 20 | -0.17 | -0.77 | 0.783 | 0.883 | 1.000 | 985 | tags=90%, list=70%, signal=294% |
| 2036 | GSE38696\_LIGHT\_ZONE\_VS\_DARK\_ZONE\_BCELL\_UP |  | 22 | -0.16 | -0.77 | 0.767 | 0.883 | 1.000 | 510 | tags=50%, list=36%, signal=77% |
| 2037 | GSE411\_100MIN\_VS\_400MIN\_IL6\_STIM\_MACROPHAGE\_UP |  | 15 | -0.19 | -0.77 | 0.735 | 0.883 | 1.000 | 152 | tags=20%, list=11%, signal=22% |
| 2038 | GSE17721\_0.5H\_VS\_8H\_GARDIQUIMOD\_BMDC\_DN |  | 17 | -0.18 | -0.77 | 0.754 | 0.883 | 1.000 | 562 | tags=65%, list=40%, signal=106% |
| 2039 | GSE34515\_CD16\_POS\_MONOCYTE\_VS\_DC\_UP |  | 22 | -0.16 | -0.77 | 0.763 | 0.884 | 1.000 | 194 | tags=23%, list=14%, signal=26% |
| 2040 | GSE38681\_WT\_VS\_LYL1\_KO\_LYMPHOID\_PRIMED\_MULTIPOTENT\_PROGENITOR\_UP |  | 23 | -0.16 | -0.77 | 0.789 | 0.884 | 1.000 | 407 | tags=39%, list=29%, signal=54% |
| 2041 | GSE360\_T\_GONDII\_VS\_M\_TUBERCULOSIS\_DC\_UP |  | 19 | -0.17 | -0.77 | 0.751 | 0.885 | 1.000 | 807 | tags=79%, list=57%, signal=182% |
| 2042 | GSE21670\_STAT3\_KO\_VS\_WT\_CD4\_TCELL\_TGFB\_IL6\_TREATED\_UP |  | 18 | -0.17 | -0.77 | 0.752 | 0.885 | 1.000 | 109 | tags=17%, list=8%, signal=18% |
| 2043 | GSE5589\_WT\_VS\_IL10\_KO\_LPS\_AND\_IL10\_STIM\_MACROPHAGE\_45MIN\_UP |  | 16 | -0.18 | -0.77 | 0.756 | 0.890 | 1.000 | 367 | tags=38%, list=26%, signal=50% |
| 2044 | GSE8835\_CD4\_VS\_CD8\_TCELL\_CLL\_PATIENT\_UP |  | 17 | -0.18 | -0.77 | 0.748 | 0.890 | 1.000 | 542 | tags=59%, list=38%, signal=94% |
| 2045 | GSE9960\_GRAM\_POS\_VS\_GRAM\_NEG\_AND\_POS\_SEPSIS\_PBMC\_DN |  | 23 | -0.16 | -0.77 | 0.763 | 0.890 | 1.000 | 468 | tags=52%, list=33%, signal=77% |
| 2046 | GSE37532\_WT\_VS\_PPARG\_KO\_VISCERAL\_ADIPOSE\_TISSUE\_TREG\_DN |  | 17 | -0.18 | -0.76 | 0.762 | 0.891 | 1.000 | 183 | tags=24%, list=13%, signal=27% |
| 2047 | GSE8835\_CD4\_VS\_CD8\_TCELL\_CLL\_PATIENT\_DN |  | 16 | -0.18 | -0.76 | 0.783 | 0.891 | 1.000 | 56 | tags=13%, list=4%, signal=13% |
| 2048 | GSE41978\_WT\_VS\_ID2\_KO\_KLRG1\_LOW\_EFFECTOR\_CD8\_TCELL\_DN |  | 24 | -0.15 | -0.76 | 0.797 | 0.891 | 1.000 | 547 | tags=58%, list=39%, signal=94% |
| 2049 | GSE9006\_TYPE\_1\_DIABETES\_AT\_DX\_VS\_1MONTH\_POST\_DX\_PBMC\_DN |  | 19 | -0.17 | -0.76 | 0.765 | 0.891 | 1.000 | 248 | tags=26%, list=18%, signal=31% |
| 2050 | GSE27786\_NKTCELL\_VS\_ERYTHROBLAST\_DN |  | 20 | -0.17 | -0.76 | 0.776 | 0.892 | 1.000 | 269 | tags=30%, list=19%, signal=37% |
| 2051 | GSE339\_EX\_VIVO\_VS\_IN\_CULTURE\_CD4POS\_DC\_DN |  | 20 | -0.17 | -0.76 | 0.760 | 0.893 | 1.000 | 599 | tags=65%, list=42%, signal=111% |
| 2052 | GSE21927\_SPLEEN\_VS\_BONE\_MARROW\_MONOCYTE\_BALBC\_UP |  | 18 | -0.17 | -0.76 | 0.740 | 0.893 | 1.000 | 266 | tags=28%, list=19%, signal=34% |
| 2053 | GSE11864\_CSF1\_VS\_CSF1\_PAM3CYS\_IN\_MAC\_UP |  | 25 | -0.15 | -0.76 | 0.783 | 0.894 | 1.000 | 79 | tags=12%, list=6%, signal=12% |
| 2054 | GSE3039\_ALPHABETA\_CD8\_TCELL\_VS\_B2\_BCELL\_DN |  | 22 | -0.16 | -0.76 | 0.764 | 0.894 | 1.000 | 77 | tags=14%, list=5%, signal=14% |
| 2055 | GSE17721\_CPG\_VS\_GARDIQUIMOD\_12H\_BMDC\_DN |  | 20 | -0.17 | -0.76 | 0.740 | 0.895 | 1.000 | 466 | tags=50%, list=33%, signal=74% |
| 2056 | GSE8835\_CD4\_VS\_CD8\_TCELL\_UP |  | 26 | -0.15 | -0.76 | 0.774 | 0.894 | 1.000 | 176 | tags=19%, list=12%, signal=22% |
| 2057 | GSE3982\_EOSINOPHIL\_VS\_BCELL\_UP |  | 18 | -0.17 | -0.76 | 0.768 | 0.895 | 1.000 | 498 | tags=56%, list=35%, signal=85% |
| 2058 | GSE17721\_POLYIC\_VS\_GARDIQUIMOD\_12H\_BMDC\_DN |  | 24 | -0.16 | -0.76 | 0.785 | 0.895 | 1.000 | 170 | tags=21%, list=12%, signal=23% |
| 2059 | GSE17721\_POLYIC\_VS\_GARDIQUIMOD\_0.5H\_BMDC\_DN |  | 18 | -0.17 | -0.76 | 0.779 | 0.895 | 1.000 | 360 | tags=39%, list=26%, signal=52% |
| 2060 | GSE7348\_UNSTIM\_VS\_TOLERIZED\_AND\_LPS\_STIM\_MACROPHAGE\_UP |  | 18 | -0.17 | -0.76 | 0.768 | 0.896 | 1.000 | 973 | tags=89%, list=69%, signal=283% |
| 2061 | GSE17721\_LPS\_VS\_POLYIC\_4H\_BMDC\_DN |  | 17 | -0.18 | -0.76 | 0.756 | 0.897 | 1.000 | 734 | tags=76%, list=52%, signal=157% |
| 2062 | GSE45365\_CD8A\_DC\_VS\_CD11B\_DC\_UP |  | 17 | -0.17 | -0.76 | 0.787 | 0.896 | 1.000 | 1169 | tags=100%, list=83%, signal=576% |
| 2063 | GSE23308\_WT\_VS\_MINERALCORTICOID\_REC\_KO\_MACROPHAGE\_UP |  | 15 | -0.18 | -0.75 | 0.796 | 0.898 | 1.000 | 173 | tags=20%, list=12%, signal=23% |
| 2064 | GSE23568\_CTRL\_VS\_ID3\_TRANSDUCED\_CD8\_TCELL\_DN |  | 16 | -0.18 | -0.75 | 0.811 | 0.898 | 1.000 | 972 | tags=94%, list=69%, signal=298% |
| 2065 | GSE17721\_POLYIC\_VS\_PAM3CSK4\_12H\_BMDC\_DN |  | 17 | -0.18 | -0.75 | 0.770 | 0.901 | 1.000 | 683 | tags=71%, list=48%, signal=135% |
| 2066 | GSE17721\_LPS\_VS\_GARDIQUIMOD\_16H\_BMDC\_DN |  | 16 | -0.18 | -0.75 | 0.793 | 0.901 | 1.000 | 202 | tags=25%, list=14%, signal=29% |
| 2067 | GSE37301\_HEMATOPOIETIC\_STEM\_CELL\_VS\_RAG2\_KO\_NK\_CELL\_UP |  | 19 | -0.17 | -0.75 | 0.757 | 0.903 | 1.000 | 52 | tags=11%, list=4%, signal=11% |
| 2068 | GSE16266\_CTRL\_VS\_HEATSHOCK\_AND\_LPS\_STIM\_MEF\_UP |  | 17 | -0.18 | -0.75 | 0.768 | 0.903 | 1.000 | 1167 | tags=100%, list=83%, signal=571% |
| 2069 | GSE9960\_HEALTHY\_VS\_SEPSIS\_PBMC\_UP |  | 15 | -0.18 | -0.75 | 0.799 | 0.903 | 1.000 | 572 | tags=67%, list=41%, signal=111% |
| 2070 | GSE15750\_DAY6\_VS\_DAY10\_EFF\_CD8\_TCELL\_DN |  | 29 | -0.14 | -0.75 | 0.769 | 0.904 | 1.000 | 228 | tags=24%, list=16%, signal=28% |
| 2071 | GSE2585\_THYMIC\_MACROPHAGE\_VS\_MTEC\_UP |  | 15 | -0.18 | -0.75 | 0.789 | 0.904 | 1.000 | 372 | tags=40%, list=26%, signal=54% |
| 2072 | GSE37532\_TREG\_VS\_TCONV\_PPARG\_KO\_CD4\_TCELL\_FROM\_VISCERAL\_ADIPOSE\_TISSUE\_UP |  | 25 | -0.15 | -0.75 | 0.781 | 0.904 | 1.000 | 1199 | tags=100%, list=85%, signal=654% |
| 2073 | GSE360\_L\_DONOVANI\_VS\_L\_MAJOR\_MAC\_DN |  | 16 | -0.18 | -0.75 | 0.782 | 0.907 | 1.000 | 547 | tags=63%, list=39%, signal=101% |
| 2074 | GSE32423\_IL7\_VS\_IL4\_MEMORY\_CD8\_TCELL\_DN |  | 17 | -0.17 | -0.74 | 0.768 | 0.908 | 1.000 | 749 | tags=76%, list=53%, signal=161% |
| 2075 | GSE16522\_ANTI\_CD3CD28\_STIM\_VS\_UNSTIM\_NAIVE\_CD8\_TCELL\_UP |  | 25 | -0.15 | -0.74 | 0.785 | 0.908 | 1.000 | 1091 | tags=96%, list=77%, signal=416% |
| 2076 | GSE46606\_UNSTIM\_VS\_CD40L\_IL2\_IL5\_DAY3\_STIMULATED\_BCELL\_DN |  | 38 | -0.13 | -0.74 | 0.806 | 0.909 | 1.000 | 947 | tags=87%, list=67%, signal=257% |
| 2077 | GSE10273\_HIGH\_VS\_LOW\_IL7\_TREATED\_IRF4\_8\_NULL\_PRE\_BCELL\_UP |  | 21 | -0.16 | -0.74 | 0.779 | 0.909 | 1.000 | 152 | tags=19%, list=11%, signal=21% |
| 2078 | GSE39820\_CTRL\_VS\_TGFBETA3\_IL6\_IL23A\_CD4\_TCELL\_UP |  | 28 | -0.14 | -0.74 | 0.772 | 0.909 | 1.000 | 1217 | tags=100%, list=86%, signal=713% |
| 2079 | GSE21670\_TGFB\_VS\_IL6\_TREATED\_STAT3\_KO\_CD4\_TCELL\_DN |  | 20 | -0.16 | -0.74 | 0.787 | 0.909 | 1.000 | 514 | tags=55%, list=36%, signal=85% |
| 2080 | GSE34006\_UNTREATED\_VS\_A2AR\_AGONIST\_TREATED\_TREG\_UP |  | 21 | -0.15 | -0.74 | 0.792 | 0.909 | 1.000 | 317 | tags=38%, list=22%, signal=48% |
| 2081 | GSE22886\_TH1\_VS\_TH2\_12H\_ACT\_UP |  | 19 | -0.16 | -0.74 | 0.820 | 0.909 | 1.000 | 266 | tags=32%, list=19%, signal=38% |
| 2082 | GSE7460\_CD8\_TCELL\_VS\_CD4\_TCELL\_ACT\_DN |  | 20 | -0.16 | -0.74 | 0.821 | 0.909 | 1.000 | 269 | tags=30%, list=19%, signal=37% |
| 2083 | GSE15624\_CTRL\_VS\_6H\_HALOFUGINONE\_TREATED\_CD4\_TCELL\_DN |  | 26 | -0.15 | -0.74 | 0.753 | 0.910 | 1.000 | 1047 | tags=92%, list=74%, signal=351% |
| 2084 | GSE3565\_DUSP1\_VS\_WT\_SPLENOCYTES\_DN |  | 21 | -0.16 | -0.74 | 0.767 | 0.910 | 1.000 | 195 | tags=19%, list=14%, signal=22% |
| 2085 | GSE3982\_MAST\_CELL\_VS\_BCELL\_UP |  | 22 | -0.16 | -0.74 | 0.818 | 0.910 | 1.000 | 606 | tags=64%, list=43%, signal=110% |
| 2086 | GSE1460\_NAIVE\_CD4\_TCELL\_ADULT\_BLOOD\_VS\_THYMIC\_STROMAL\_CELL\_DN |  | 19 | -0.16 | -0.74 | 0.789 | 0.910 | 1.000 | 524 | tags=58%, list=37%, signal=91% |
| 2087 | GSE4811\_CLASSSICALY\_ACTIVATED\_VS\_TYPE\_2\_ACTIVATED\_MACROPHAGE\_UP |  | 20 | -0.16 | -0.74 | 0.801 | 0.911 | 1.000 | 265 | tags=30%, list=19%, signal=36% |
| 2088 | GSE22611\_NOD2\_TRANSD\_VS\_CTRL\_TRANSD\_HEK293\_MDP\_STIM\_2H\_UP |  | 17 | -0.18 | -0.74 | 0.793 | 0.911 | 1.000 | 177 | tags=24%, list=13%, signal=27% |
| 2089 | GSE39110\_UNTREATED\_VS\_IL2\_TREATED\_CD8\_TCELL\_DAY3\_POST\_IMMUNIZATION\_UP |  | 22 | -0.16 | -0.74 | 0.813 | 0.912 | 1.000 | 547 | tags=59%, list=39%, signal=95% |
| 2090 | GSE11961\_MEMORY\_BCELL\_DAY7\_VS\_GERMINAL\_CENTER\_BCELL\_DAY40\_DN |  | 18 | -0.17 | -0.74 | 0.808 | 0.913 | 1.000 | 383 | tags=44%, list=27%, signal=60% |
| 2091 | GSE3982\_MAST\_CELL\_VS\_NKCELL\_UP |  | 26 | -0.15 | -0.73 | 0.804 | 0.915 | 1.000 | 236 | tags=23%, list=17%, signal=27% |
| 2092 | GSE17721\_CTRL\_VS\_GARDIQUIMOD\_6H\_BMDC\_DN |  | 19 | -0.16 | -0.73 | 0.794 | 0.915 | 1.000 | 188 | tags=21%, list=13%, signal=24% |
| 2093 | GSE22025\_PROGESTERONE\_VS\_TGFB1\_AND\_PROGESTERONE\_TREATED\_CD4\_TCELL\_DN |  | 16 | -0.18 | -0.73 | 0.786 | 0.917 | 1.000 | 313 | tags=38%, list=22%, signal=48% |
| 2094 | GSE17721\_CTRL\_VS\_CPG\_2H\_BMDC\_UP |  | 21 | -0.16 | -0.73 | 0.813 | 0.917 | 1.000 | 219 | tags=24%, list=16%, signal=28% |
| 2095 | GSE7460\_WT\_VS\_FOXP3\_HET\_ACT\_WITH\_TGFB\_TCONV\_DN |  | 17 | -0.17 | -0.73 | 0.804 | 0.917 | 1.000 | 266 | tags=29%, list=19%, signal=36% |
| 2096 | GSE22935\_24H\_VS\_48H\_MBOVIS\_BCG\_STIM\_MACROPHAGE\_DN |  | 18 | -0.17 | -0.73 | 0.818 | 0.918 | 1.000 | 1065 | tags=94%, list=75%, signal=380% |
| 2097 | GSE24081\_CONTROLLER\_VS\_PROGRESSOR\_HIV\_SPECIFIC\_CD8\_TCELL\_DN |  | 21 | -0.16 | -0.73 | 0.835 | 0.918 | 1.000 | 325 | tags=33%, list=23%, signal=43% |
| 2098 | GSE44732\_UNSTIM\_VS\_IL27\_STIM\_IMATURE\_DC\_DN |  | 23 | -0.15 | -0.73 | 0.810 | 0.917 | 1.000 | 141 | tags=17%, list=10%, signal=19% |
| 2099 | GSE20198\_UNTREATED\_VS\_IL12\_IL18\_TREATED\_ACT\_CD4\_TCELL\_DN |  | 17 | -0.17 | -0.73 | 0.816 | 0.918 | 1.000 | 1175 | tags=100%, list=83%, signal=591% |
| 2100 | GSE17721\_LPS\_VS\_POLYIC\_24H\_BMDC\_UP |  | 19 | -0.16 | -0.73 | 0.833 | 0.918 | 1.000 | 2 | tags=5%, list=0%, signal=5% |
| 2101 | GSE21546\_SAP1A\_KO\_VS\_SAP1A\_KO\_AND\_ELK1\_KO\_ANTI\_CD3\_STIM\_DP\_THYMOCYTES\_DN |  | 17 | -0.17 | -0.73 | 0.813 | 0.919 | 1.000 | 355 | tags=41%, list=25%, signal=54% |
| 2102 | GSE42021\_CD24INT\_VS\_CD24LOW\_TCONV\_THYMUS\_DN |  | 31 | -0.14 | -0.73 | 0.819 | 0.918 | 1.000 | 99 | tags=13%, list=7%, signal=14% |
| 2103 | GSE2405\_HEAT\_KILLED\_VS\_LIVE\_A\_PHAGOCYTOPHILUM\_STIM\_NEUTROPHIL\_24H\_DN |  | 16 | -0.17 | -0.73 | 0.792 | 0.918 | 1.000 | 145 | tags=19%, list=10%, signal=21% |
| 2104 | GSE21927\_GMCSF\_IL6\_VS\_GMCSF\_GCSF\_TREATED\_BONE\_MARROW\_UP |  | 17 | -0.17 | -0.73 | 0.789 | 0.919 | 1.000 | 470 | tags=53%, list=33%, signal=78% |
| 2105 | GSE17721\_POLYIC\_VS\_CPG\_0.5H\_BMDC\_DN |  | 19 | -0.16 | -0.73 | 0.831 | 0.920 | 1.000 | 1185 | tags=100%, list=84%, signal=616% |
| 2106 | GSE32255\_UNSTIM\_VS\_4H\_LPS\_STIM\_DC\_UP |  | 15 | -0.18 | -0.72 | 0.832 | 0.921 | 1.000 | 646 | tags=67%, list=46%, signal=122% |
| 2107 | GSE32901\_NAIVE\_VS\_TH17\_ENRICHED\_CD4\_TCELL\_DN |  | 18 | -0.17 | -0.72 | 0.808 | 0.921 | 1.000 | 443 | tags=50%, list=31%, signal=72% |
| 2108 | GSE39152\_CD103\_NEG\_VS\_POS\_MEMORY\_CD8\_TCELL\_UP |  | 19 | -0.16 | -0.72 | 0.803 | 0.921 | 1.000 | 605 | tags=63%, list=43%, signal=109% |
| 2109 | GSE7459\_UNTREATED\_VS\_IL6\_TREATED\_ACT\_CD4\_TCELL\_DN |  | 15 | -0.18 | -0.72 | 0.823 | 0.920 | 1.000 | 315 | tags=33%, list=22%, signal=42% |
| 2110 | GSE14350\_TREG\_VS\_TEFF\_IN\_IL2RB\_KO\_UP |  | 29 | -0.14 | -0.72 | 0.823 | 0.921 | 1.000 | 985 | tags=86%, list=70%, signal=280% |
| 2111 | GSE9988\_ANTI\_TREM1\_AND\_LPS\_VS\_CTRL\_TREATED\_MONOCYTES\_DN |  | 20 | -0.16 | -0.72 | 0.839 | 0.922 | 1.000 | 220 | tags=25%, list=16%, signal=29% |
| 2112 | GSE45739\_NRAS\_KO\_VS\_WT\_ACD3\_ACD28\_STIM\_CD4\_TCELL\_UP |  | 43 | -0.12 | -0.72 | 0.843 | 0.922 | 1.000 | 831 | tags=77%, list=59%, signal=181% |
| 2113 | GSE21380\_NON\_TFH\_VS\_GERMINAL\_CENTER\_TFH\_CD4\_TCELL\_DN |  | 22 | -0.15 | -0.72 | 0.811 | 0.922 | 1.000 | 353 | tags=36%, list=25%, signal=48% |
| 2114 | GSE360\_CTRL\_VS\_B\_MALAYI\_LOW\_DOSE\_MAC\_DN |  | 16 | -0.17 | -0.72 | 0.819 | 0.923 | 1.000 | 280 | tags=31%, list=20%, signal=39% |
| 2115 | GSE35685\_CD34POS\_CD10NEG\_CD62LPOS\_VS\_CD34POS\_CD10POS\_BONE\_MARROW\_UP |  | 28 | -0.14 | -0.72 | 0.840 | 0.923 | 1.000 | 160 | tags=18%, list=11%, signal=20% |
| 2116 | GSE30083\_SP1\_VS\_SP2\_THYMOCYTE\_UP |  | 17 | -0.17 | -0.72 | 0.822 | 0.923 | 1.000 | 49 | tags=12%, list=3%, signal=12% |
| 2117 | GSE42021\_TREG\_PLN\_VS\_CD24LO\_TREG\_THYMUS\_UP |  | 15 | -0.18 | -0.72 | 0.813 | 0.923 | 1.000 | 53 | tags=13%, list=4%, signal=14% |
| 2118 | GSE43863\_NAIVE\_VS\_MEMORY\_LY6C\_INT\_CXCR5POS\_CD4\_TCELL\_D150\_LCMV\_DN |  | 25 | -0.15 | -0.72 | 0.833 | 0.923 | 1.000 | 24 | tags=8%, list=2%, signal=8% |
| 2119 | GSE14308\_TH1\_VS\_INDUCED\_TREG\_DN |  | 22 | -0.15 | -0.72 | 0.833 | 0.923 | 1.000 | 1204 | tags=100%, list=85%, signal=671% |
| 2120 | GSE22443\_NAIVE\_VS\_ACT\_AND\_IL2\_TREATED\_CD8\_TCELL\_DN |  | 17 | -0.16 | -0.72 | 0.804 | 0.923 | 1.000 | 563 | tags=65%, list=40%, signal=106% |
| 2121 | GSE17974\_CTRL\_VS\_ACT\_IL4\_AND\_ANTI\_IL12\_6H\_CD4\_TCELL\_DN |  | 32 | -0.13 | -0.72 | 0.855 | 0.923 | 1.000 | 49 | tags=9%, list=3%, signal=9% |
| 2122 | GSE21379\_TFH\_VS\_NON\_TFH\_SAP\_KO\_CD4\_TCELL\_DN |  | 20 | -0.16 | -0.72 | 0.850 | 0.925 | 1.000 | 233 | tags=25%, list=17%, signal=30% |
| 2123 | GSE46606\_IRF4HIGH\_VS\_IRF4MID\_CD40L\_IL2\_IL5\_DAY3\_STIMULATED\_BCELL\_UP |  | 22 | -0.15 | -0.72 | 0.824 | 0.925 | 1.000 | 251 | tags=27%, list=18%, signal=33% |
| 2124 | GSE43955\_1H\_VS\_60H\_ACT\_CD4\_TCELL\_WITH\_TGFB\_IL6\_UP |  | 20 | -0.16 | -0.72 | 0.807 | 0.924 | 1.000 | 483 | tags=50%, list=34%, signal=75% |
| 2125 | GSE37301\_COMMON\_LYMPHOID\_PROGENITOR\_VS\_CD4\_TCELL\_DN |  | 16 | -0.17 | -0.72 | 0.834 | 0.924 | 1.000 | 580 | tags=63%, list=41%, signal=105% |
| 2126 | GSE42021\_TREG\_VS\_TCONV\_PLN\_UP |  | 31 | -0.13 | -0.71 | 0.855 | 0.924 | 1.000 | 512 | tags=52%, list=36%, signal=79% |
| 2127 | GSE41867\_LCMV\_ARMSTRONG\_VS\_CLONE13\_DAY6\_EFFECTOR\_CD8\_TCELL\_DN |  | 27 | -0.14 | -0.71 | 0.828 | 0.924 | 1.000 | 24 | tags=7%, list=2%, signal=7% |
| 2128 | GSE1460\_DP\_THYMOCYTE\_VS\_NAIVE\_CD4\_TCELL\_ADULT\_BLOOD\_DN |  | 21 | -0.15 | -0.71 | 0.811 | 0.924 | 1.000 | 1073 | tags=95%, list=76%, signal=392% |
| 2129 | GSE17721\_12H\_VS\_24H\_GARDIQUIMOD\_BMDC\_DN |  | 17 | -0.17 | -0.71 | 0.851 | 0.924 | 1.000 | 356 | tags=35%, list=25%, signal=47% |
| 2130 | GSE36527\_CD62L\_HIGH\_VS\_CD62L\_LOW\_TREG\_CD69\_NEG\_KLRG1\_NEG\_DN |  | 20 | -0.15 | -0.71 | 0.823 | 0.924 | 1.000 | 184 | tags=20%, list=13%, signal=23% |
| 2131 | GSE1925\_CTRL\_VS\_24H\_IFNG\_STIM\_IFNG\_PRIMED\_MACROPHAGE\_UP |  | 21 | -0.15 | -0.71 | 0.841 | 0.924 | 1.000 | 418 | tags=43%, list=30%, signal=60% |
| 2132 | GSE5589\_WT\_VS\_IL10\_KO\_LPS\_AND\_IL6\_STIM\_MACROPHAGE\_45MIN\_DN |  | 16 | -0.16 | -0.71 | 0.853 | 0.928 | 1.000 | 1190 | tags=100%, list=84%, signal=631% |
| 2133 | GSE16385\_ROSIGLITAZONE\_VS\_UNTREATED\_IFNG\_TNF\_STIM\_MACROPHAGE\_UP |  | 17 | -0.17 | -0.71 | 0.846 | 0.928 | 1.000 | 402 | tags=41%, list=28%, signal=57% |
| 2134 | GSE5542\_IFNG\_VS\_IFNA\_AND\_IFNG\_TREATED\_EPITHELIAL\_CELLS\_6H\_DN |  | 19 | -0.16 | -0.71 | 0.842 | 0.928 | 1.000 | 614 | tags=63%, list=44%, signal=110% |
| 2135 | GSE18893\_CTRL\_VS\_TNF\_TREATED\_TREG\_24H\_UP |  | 23 | -0.15 | -0.71 | 0.836 | 0.928 | 1.000 | 689 | tags=70%, list=49%, signal=134% |
| 2136 | KAECH\_NAIVE\_VS\_DAY15\_EFF\_CD8\_TCELL\_UP |  | 17 | -0.16 | -0.71 | 0.825 | 0.929 | 1.000 | 56 | tags=12%, list=4%, signal=12% |
| 2137 | GSE29164\_UNTREATED\_VS\_CD8\_TCELL\_AND\_IL12\_TREATED\_MELANOMA\_DAY7\_UP |  | 27 | -0.14 | -0.71 | 0.839 | 0.929 | 1.000 | 438 | tags=44%, list=31%, signal=63% |
| 2138 | GSE24634\_NAIVE\_CD4\_TCELL\_VS\_DAY7\_IL4\_CONV\_TREG\_DN |  | 17 | -0.17 | -0.71 | 0.799 | 0.929 | 1.000 | 1052 | tags=94%, list=75%, signal=365% |
| 2139 | GSE17186\_NAIVE\_VS\_CD21HIGH\_TRANSITIONAL\_BCELL\_UP |  | 17 | -0.17 | -0.71 | 0.833 | 0.928 | 1.000 | 292 | tags=35%, list=21%, signal=44% |
| 2140 | GSE43955\_TH0\_VS\_TGFB\_IL6\_TH17\_ACT\_CD4\_TCELL\_4H\_UP |  | 27 | -0.14 | -0.71 | 0.851 | 0.928 | 1.000 | 643 | tags=70%, list=46%, signal=127% |
| 2141 | GSE22935\_UNSTIM\_VS\_12H\_MBOVIS\_BCG\_STIM\_MYD88\_KO\_MACROPHAGE\_DN |  | 17 | -0.17 | -0.71 | 0.834 | 0.928 | 1.000 | 112 | tags=18%, list=8%, signal=19% |
| 2142 | GSE3982\_MEMORY\_CD4\_TCELL\_VS\_TH2\_DN |  | 22 | -0.15 | -0.71 | 0.840 | 0.929 | 1.000 | 2 | tags=5%, list=0%, signal=4% |
| 2143 | GSE9988\_ANTI\_TREM1\_VS\_CTRL\_TREATED\_MONOCYTES\_DN |  | 20 | -0.16 | -0.71 | 0.812 | 0.930 | 1.000 | 220 | tags=25%, list=16%, signal=29% |
| 2144 | GSE21360\_PRIMARY\_VS\_TERTIARY\_MEMORY\_CD8\_TCELL\_UP |  | 25 | -0.14 | -0.70 | 0.837 | 0.930 | 1.000 | 973 | tags=88%, list=69%, signal=278% |
| 2145 | GSE10325\_CD4\_TCELL\_VS\_LUPUS\_CD4\_TCELL\_DN |  | 25 | -0.14 | -0.70 | 0.857 | 0.932 | 1.000 | 562 | tags=60%, list=40%, signal=98% |
| 2146 | GSE43955\_1H\_VS\_10H\_ACT\_CD4\_TCELL\_WITH\_TGFB\_IL6\_UP |  | 21 | -0.15 | -0.70 | 0.831 | 0.932 | 1.000 | 35 | tags=10%, list=2%, signal=10% |
| 2147 | GSE36078\_WT\_VS\_IL1R\_KO\_LUNG\_DC\_AFTER\_AD5\_T425A\_HEXON\_INF\_DN |  | 19 | -0.16 | -0.70 | 0.856 | 0.934 | 1.000 | 442 | tags=47%, list=31%, signal=68% |
| 2148 | GSE28130\_ACTIVATED\_VS\_INDUCEED\_TREG\_UP |  | 19 | -0.15 | -0.70 | 0.848 | 0.933 | 1.000 | 477 | tags=53%, list=34%, signal=78% |
| 2149 | GSE360\_T\_GONDII\_VS\_B\_MALAYI\_LOW\_DOSE\_MAC\_DN |  | 16 | -0.16 | -0.70 | 0.820 | 0.933 | 1.000 | 1183 | tags=100%, list=84%, signal=612% |
| 2150 | GSE22611\_MUTANT\_NOD2\_VS\_CTRL\_TRANSDUCED\_HEK293T\_CELL\_UP |  | 20 | -0.15 | -0.70 | 0.873 | 0.933 | 1.000 | 958 | tags=90%, list=68%, signal=276% |
| 2151 | GSE28737\_WT\_VS\_BCL6\_KO\_FOLLICULAR\_BCELL\_DN |  | 24 | -0.14 | -0.70 | 0.853 | 0.933 | 1.000 | 259 | tags=25%, list=18%, signal=30% |
| 2152 | GSE22140\_GERMFREE\_VS\_SPF\_MOUSE\_CD4\_TCELL\_DN |  | 22 | -0.14 | -0.70 | 0.814 | 0.934 | 1.000 | 293 | tags=27%, list=21%, signal=34% |
| 2153 | GSE14769\_UNSTIM\_VS\_240MIN\_LPS\_BMDM\_UP |  | 19 | -0.16 | -0.70 | 0.841 | 0.934 | 1.000 | 1193 | tags=100%, list=85%, signal=639% |
| 2154 | GSE17721\_12H\_VS\_24H\_CPG\_BMDC\_UP |  | 18 | -0.16 | -0.70 | 0.851 | 0.934 | 1.000 | 183 | tags=22%, list=13%, signal=25% |
| 2155 | GSE34515\_CD16\_NEG\_MONOCYTE\_VS\_DC\_DN |  | 18 | -0.16 | -0.70 | 0.854 | 0.934 | 1.000 | 2 | tags=6%, list=0%, signal=5% |
| 2156 | GSE34515\_CD16\_NEG\_VS\_POS\_MONOCYTE\_UP |  | 17 | -0.16 | -0.70 | 0.828 | 0.934 | 1.000 | 1184 | tags=100%, list=84%, signal=614% |
| 2157 | GSE9006\_HEALTHY\_VS\_TYPE\_1\_DIABETES\_PBMC\_4MONTH\_POST\_DX\_UP |  | 28 | -0.14 | -0.70 | 0.829 | 0.934 | 1.000 | 643 | tags=68%, list=46%, signal=122% |
| 2158 | GSE26343\_UNSTIM\_VS\_LPS\_STIM\_MACROPHAGE\_UP |  | 27 | -0.14 | -0.70 | 0.861 | 0.934 | 1.000 | 1220 | tags=100%, list=86%, signal=725% |
| 2159 | GSE5589\_WT\_VS\_IL10\_KO\_LPS\_STIM\_MACROPHAGE\_180MIN\_UP |  | 18 | -0.16 | -0.70 | 0.806 | 0.934 | 1.000 | 379 | tags=39%, list=27%, signal=52% |
| 2160 | GSE45365\_WT\_VS\_IFNAR\_KO\_CD8A\_DC\_DN |  | 23 | -0.15 | -0.70 | 0.836 | 0.934 | 1.000 | 572 | tags=61%, list=41%, signal=101% |
| 2161 | GSE43956\_WT\_VS\_SGK1\_KO\_TH17\_DIFFERENTIATED\_CD4\_TCELL\_DN |  | 29 | -0.13 | -0.69 | 0.856 | 0.935 | 1.000 | 513 | tags=52%, list=36%, signal=80% |
| 2162 | GSE17974\_CTRL\_VS\_ACT\_IL4\_AND\_ANTI\_IL12\_72H\_CD4\_TCELL\_DN |  | 24 | -0.14 | -0.69 | 0.842 | 0.935 | 1.000 | 1214 | tags=100%, list=86%, signal=704% |
| 2163 | GSE10856\_CTRL\_VS\_TNFRSF6B\_IN\_MACROPHAGE\_DN |  | 20 | -0.16 | -0.69 | 0.850 | 0.935 | 1.000 | 1093 | tags=95%, list=77%, signal=416% |
| 2164 | GSE3982\_NEUTROPHIL\_VS\_BASOPHIL\_DN |  | 16 | -0.17 | -0.69 | 0.857 | 0.936 | 1.000 | 726 | tags=75%, list=51%, signal=153% |
| 2165 | GSE29618\_MONOCYTE\_VS\_PDC\_DAY7\_FLU\_VACCINE\_DN |  | 17 | -0.17 | -0.69 | 0.821 | 0.936 | 1.000 | 207 | tags=24%, list=15%, signal=27% |
| 2166 | GSE20727\_CTRL\_VS\_ROS\_INH\_AND\_DNFB\_ALLERGEN\_TREATED\_DC\_UP |  | 15 | -0.17 | -0.69 | 0.851 | 0.936 | 1.000 | 171 | tags=20%, list=12%, signal=23% |
| 2167 | GSE557\_CIITA\_KO\_VS\_I\_AB\_KO\_DC\_DN |  | 16 | -0.17 | -0.69 | 0.849 | 0.936 | 1.000 | 1180 | tags=100%, list=84%, signal=604% |
| 2168 | GSE27786\_LSK\_VS\_BCELL\_DN |  | 16 | -0.17 | -0.69 | 0.818 | 0.937 | 1.000 | 146 | tags=19%, list=10%, signal=21% |
| 2169 | GSE9960\_GRAM\_POS\_VS\_GRAM\_NEG\_AND\_POS\_SEPSIS\_PBMC\_UP |  | 23 | -0.15 | -0.69 | 0.852 | 0.936 | 1.000 | 804 | tags=78%, list=57%, signal=179% |
| 2170 | GSE8621\_UNSTIM\_VS\_LPS\_STIM\_MACROPHAGE\_DN |  | 23 | -0.15 | -0.69 | 0.847 | 0.936 | 1.000 | 1207 | tags=100%, list=86%, signal=680% |
| 2171 | GSE21360\_NAIVE\_VS\_QUATERNARY\_MEMORY\_CD8\_TCELL\_UP |  | 27 | -0.13 | -0.69 | 0.855 | 0.936 | 1.000 | 1227 | tags=100%, list=87%, signal=752% |
| 2172 | GSE29618\_PRE\_VS\_DAY7\_POST\_TIV\_FLU\_VACCINE\_PDC\_UP |  | 16 | -0.16 | -0.69 | 0.854 | 0.936 | 1.000 | 420 | tags=44%, list=30%, signal=62% |
| 2173 | GSE30971\_CTRL\_VS\_LPS\_STIM\_MACROPHAGE\_WBP7\_HET\_2H\_DN |  | 15 | -0.17 | -0.69 | 0.843 | 0.936 | 1.000 | 558 | tags=60%, list=40%, signal=98% |
| 2174 | GSE43863\_TFH\_VS\_LY6C\_INT\_CXCR5POS\_EFFECTOR\_CD4\_TCELL\_UP |  | 22 | -0.15 | -0.69 | 0.872 | 0.936 | 1.000 | 1080 | tags=95%, list=77%, signal=401% |
| 2175 | GSE37533\_PPARG1\_FOXP3\_VS\_FOXP3\_TRANSDUCED\_CD4\_TCELL\_DN |  | 25 | -0.13 | -0.69 | 0.855 | 0.935 | 1.000 | 370 | tags=36%, list=26%, signal=48% |
| 2176 | GSE360\_HIGH\_DOSE\_B\_MALAYI\_VS\_M\_TUBERCULOSIS\_DC\_UP |  | 15 | -0.16 | -0.69 | 0.820 | 0.936 | 1.000 | 1183 | tags=100%, list=84%, signal=612% |
| 2177 | GSE33424\_CD161\_HIGH\_VS\_INT\_CD8\_TCELL\_UP |  | 23 | -0.14 | -0.69 | 0.843 | 0.936 | 1.000 | 659 | tags=70%, list=47%, signal=128% |
| 2178 | GSE7768\_OVA\_WITH\_LPS\_VS\_OVA\_WITH\_MPL\_IMMUNIZED\_MOUSE\_WHOLE\_SPLEEN\_6H\_DN |  | 21 | -0.15 | -0.69 | 0.871 | 0.936 | 1.000 | 498 | tags=52%, list=35%, signal=80% |
| 2179 | GSE16385\_MONOCYTE\_VS\_12H\_IFNG\_TNF\_TREATED\_MACROPHAGE\_UP |  | 23 | -0.14 | -0.69 | 0.852 | 0.936 | 1.000 | 97 | tags=13%, list=7%, signal=14% |
| 2180 | GSE41867\_DAY8\_EFFECTOR\_VS\_DAY30\_EXHAUSTED\_CD8\_TCELL\_LCMV\_CLONE13\_UP |  | 16 | -0.16 | -0.69 | 0.865 | 0.937 | 1.000 | 300 | tags=31%, list=21%, signal=39% |
| 2181 | GSE17721\_CTRL\_VS\_CPG\_24H\_BMDC\_DN |  | 15 | -0.17 | -0.69 | 0.875 | 0.937 | 1.000 | 5 | tags=7%, list=0%, signal=7% |
| 2182 | GSE9037\_WT\_VS\_IRAK4\_KO\_LPS\_4H\_STIM\_BMDM\_DN |  | 15 | -0.17 | -0.68 | 0.867 | 0.937 | 1.000 | 1024 | tags=93%, list=73%, signal=337% |
| 2183 | GSE2770\_UNTREATED\_VS\_TGFB\_AND\_IL4\_TREATED\_ACT\_CD4\_TCELL\_4H\_UP |  | 15 | -0.17 | -0.68 | 0.851 | 0.937 | 1.000 | 352 | tags=40%, list=25%, signal=53% |
| 2184 | GSE3720\_VD1\_VS\_VD2\_GAMMADELTA\_TCELL\_UP |  | 20 | -0.15 | -0.68 | 0.879 | 0.938 | 1.000 | 116 | tags=15%, list=8%, signal=16% |
| 2185 | GSE42021\_CD24HI\_VS\_CD24INT\_TREG\_THYMUS\_DN |  | 28 | -0.13 | -0.68 | 0.875 | 0.938 | 1.000 | 557 | tags=57%, list=39%, signal=93% |
| 2186 | GSE40274\_FOXP3\_VS\_FOXP3\_AND\_EOS\_TRANSDUCED\_ACTIVATED\_CD4\_TCELL\_DN |  | 20 | -0.15 | -0.68 | 0.862 | 0.938 | 1.000 | 180 | tags=20%, list=13%, signal=23% |
| 2187 | GSE25088\_WT\_VS\_STAT6\_KO\_MACROPHAGE\_ROSIGLITAZONE\_STIM\_DN |  | 17 | -0.16 | -0.68 | 0.860 | 0.939 | 1.000 | 398 | tags=41%, list=28%, signal=57% |
| 2188 | GSE2770\_IL4\_ACT\_VS\_ACT\_CD4\_TCELL\_2H\_DN |  | 33 | -0.12 | -0.68 | 0.855 | 0.940 | 1.000 | 266 | tags=27%, list=19%, signal=33% |
| 2189 | GSE42021\_CD24HI\_VS\_CD24INT\_TCONV\_THYMUS\_DN |  | 16 | -0.16 | -0.68 | 0.883 | 0.940 | 1.000 | 373 | tags=38%, list=26%, signal=50% |
| 2190 | GSE12392\_WT\_VS\_IFNB\_KO\_CD8A\_POS\_SPLEEN\_DC\_UP |  | 22 | -0.15 | -0.68 | 0.876 | 0.940 | 1.000 | 318 | tags=32%, list=23%, signal=40% |
| 2191 | GSE22611\_NOD2\_VS\_CTRL\_TRANSDUCED\_HEK293T\_CELL\_DN |  | 22 | -0.15 | -0.68 | 0.848 | 0.940 | 1.000 | 415 | tags=41%, list=29%, signal=57% |
| 2192 | GSE42724\_B1\_BCELL\_VS\_PLASMABLAST\_DN |  | 20 | -0.15 | -0.68 | 0.859 | 0.940 | 1.000 | 1200 | tags=100%, list=85%, signal=659% |
| 2193 | GSE25123\_WT\_VS\_PPARG\_KO\_MACROPHAGE\_IL4\_STIM\_UP |  | 25 | -0.14 | -0.68 | 0.870 | 0.940 | 1.000 | 415 | tags=40%, list=29%, signal=56% |
| 2194 | GSE6269\_STAPH\_AUREUS\_VS\_STREP\_PNEUMO\_INF\_PBMC\_DN |  | 15 | -0.17 | -0.68 | 0.845 | 0.941 | 1.000 | 313 | tags=33%, list=22%, signal=42% |
| 2195 | GSE14769\_UNSTIM\_VS\_60MIN\_LPS\_BMDM\_UP |  | 20 | -0.15 | -0.68 | 0.870 | 0.942 | 1.000 | 1200 | tags=100%, list=85%, signal=659% |
| 2196 | GSE11924\_TFH\_VS\_TH2\_CD4\_TCELL\_UP |  | 21 | -0.14 | -0.67 | 0.869 | 0.943 | 1.000 | 295 | tags=29%, list=21%, signal=36% |
| 2197 | GSE42021\_CD24HI\_VS\_CD24LOW\_TCONV\_THYMUS\_DN |  | 23 | -0.14 | -0.67 | 0.859 | 0.946 | 1.000 | 1001 | tags=91%, list=71%, signal=309% |
| 2198 | GSE17301\_ACD3\_ACD28\_VS\_ACD3\_ACD28\_AND\_IFNA2\_STIM\_CD8\_TCELL\_UP |  | 18 | -0.15 | -0.67 | 0.881 | 0.949 | 1.000 | 205 | tags=22%, list=15%, signal=26% |
| 2199 | GSE411\_UNSTIM\_VS\_100MIN\_IL6\_STIM\_MACROPHAGE\_DN |  | 19 | -0.15 | -0.67 | 0.878 | 0.949 | 1.000 | 438 | tags=47%, list=31%, signal=68% |
| 2200 | GSE14000\_TRANSLATED\_RNA\_VS\_MRNA\_16H\_LPS\_DC\_UP |  | 15 | -0.16 | -0.67 | 0.898 | 0.949 | 1.000 | 249 | tags=27%, list=18%, signal=32% |
| 2201 | GSE17721\_4\_VS\_24H\_GARDIQUIMOD\_BMDC\_DN |  | 24 | -0.13 | -0.67 | 0.877 | 0.950 | 1.000 | 509 | tags=50%, list=36%, signal=77% |
| 2202 | GSE1740\_UNSTIM\_VS\_IFNA\_STIMULATED\_MCSF\_IFNG\_DERIVED\_MACROPHAGE\_DN |  | 17 | -0.16 | -0.67 | 0.871 | 0.949 | 1.000 | 1192 | tags=100%, list=84%, signal=637% |
| 2203 | GSE17721\_PAM3CSK4\_VS\_GADIQUIMOD\_6H\_BMDC\_UP |  | 25 | -0.13 | -0.66 | 0.903 | 0.950 | 1.000 | 944 | tags=88%, list=67%, signal=261% |
| 2204 | GSE40274\_CTRL\_VS\_FOXP3\_AND\_PBX1\_TRANSDUCED\_ACTIVATED\_CD4\_TCELL\_DN |  | 15 | -0.16 | -0.66 | 0.872 | 0.950 | 1.000 | 92 | tags=13%, list=7%, signal=14% |
| 2205 | GSE27786\_NKCELL\_VS\_ERYTHROBLAST\_UP |  | 19 | -0.15 | -0.66 | 0.879 | 0.950 | 1.000 | 310 | tags=32%, list=22%, signal=40% |
| 2206 | GSE22886\_UNSTIM\_VS\_IL2\_STIM\_NKCELL\_DN |  | 33 | -0.12 | -0.66 | 0.896 | 0.950 | 1.000 | 24 | tags=6%, list=2%, signal=6% |
| 2207 | GSE10239\_KLRG1INT\_VS\_KLRG1HIGH\_EFF\_CD8\_TCELL\_UP |  | 16 | -0.16 | -0.66 | 0.890 | 0.950 | 1.000 | 1190 | tags=100%, list=84%, signal=631% |
| 2208 | GSE27786\_NKTCELL\_VS\_ERYTHROBLAST\_UP |  | 26 | -0.13 | -0.66 | 0.896 | 0.949 | 1.000 | 374 | tags=38%, list=27%, signal=51% |
| 2209 | GSE36009\_UNSTIM\_VS\_LPS\_STIM\_DC\_DN |  | 34 | -0.12 | -0.66 | 0.891 | 0.949 | 1.000 | 173 | tags=18%, list=12%, signal=20% |
| 2210 | GSE19888\_ADENOSINE\_A3R\_ACT\_VS\_TCELL\_MEMBRANES\_ACT\_AND\_A3R\_INH\_PRETREAT\_IN\_MAST\_CELL\_UP |  | 16 | -0.16 | -0.66 | 0.875 | 0.949 | 1.000 | 65 | tags=13%, list=5%, signal=13% |
| 2211 | GSE41867\_NAIVE\_VS\_DAY30\_LCMV\_ARMSTRONG\_MEMORY\_CD8\_TCELL\_UP |  | 24 | -0.13 | -0.66 | 0.892 | 0.949 | 1.000 | 155 | tags=17%, list=11%, signal=18% |
| 2212 | GSE33162\_HDAC3\_KO\_VS\_HDAC3\_KO\_4H\_LPS\_STIM\_MACROPHAGE\_UP |  | 21 | -0.14 | -0.66 | 0.885 | 0.949 | 1.000 | 177 | tags=19%, list=13%, signal=21% |
| 2213 | GSE16385\_MONOCYTE\_VS\_12H\_ROSIGLITAZONE\_IL4\_TREATED\_MACROPHAGE\_DN |  | 27 | -0.12 | -0.66 | 0.906 | 0.949 | 1.000 | 355 | tags=33%, list=25%, signal=44% |
| 2214 | GSE5589\_LPS\_AND\_IL10\_VS\_LPS\_AND\_IL6\_STIM\_MACROPHAGE\_45MIN\_UP |  | 17 | -0.15 | -0.66 | 0.889 | 0.951 | 1.000 | 50 | tags=12%, list=4%, signal=12% |
| 2215 | GSE360\_L\_DONOVANI\_VS\_B\_MALAYI\_LOW\_DOSE\_DC\_DN |  | 20 | -0.14 | -0.66 | 0.868 | 0.950 | 1.000 | 474 | tags=50%, list=34%, signal=74% |
| 2216 | GSE22611\_UNSTIM\_VS\_6H\_MDP\_STIM\_MUTANT\_NOD2\_TRANSDUCED\_HEK293T\_CELL\_UP |  | 16 | -0.16 | -0.65 | 0.902 | 0.956 | 1.000 | 442 | tags=50%, list=31%, signal=72% |
| 2217 | GSE25123\_CTRL\_VS\_IL4\_AND\_ROSIGLITAZONE\_STIM\_MACROPHAGE\_DN |  | 15 | -0.16 | -0.65 | 0.915 | 0.957 | 1.000 | 237 | tags=27%, list=17%, signal=32% |
| 2218 | GSE17721\_CTRL\_VS\_CPG\_6H\_BMDC\_UP |  | 17 | -0.15 | -0.65 | 0.877 | 0.959 | 1.000 | 1200 | tags=100%, list=85%, signal=661% |
| 2219 | GSE17721\_CTRL\_VS\_CPG\_4H\_BMDC\_UP |  | 16 | -0.15 | -0.65 | 0.890 | 0.959 | 1.000 | 1200 | tags=100%, list=85%, signal=661% |
| 2220 | GSE3982\_MAST\_CELL\_VS\_CENT\_MEMORY\_CD4\_TCELL\_DN |  | 19 | -0.15 | -0.65 | 0.891 | 0.959 | 1.000 | 473 | tags=47%, list=34%, signal=70% |
| 2221 | GSE1460\_CORD\_VS\_ADULT\_BLOOD\_NAIVE\_CD4\_TCELL\_DN |  | 21 | -0.14 | -0.65 | 0.893 | 0.960 | 1.000 | 180 | tags=19%, list=13%, signal=22% |
| 2222 | GSE17721\_CTRL\_VS\_POLYIC\_8H\_BMDC\_UP |  | 16 | -0.16 | -0.65 | 0.898 | 0.961 | 1.000 | 1195 | tags=100%, list=85%, signal=646% |
| 2223 | GSE17721\_CTRL\_VS\_GARDIQUIMOD\_4H\_BMDC\_UP |  | 26 | -0.13 | -0.64 | 0.917 | 0.963 | 1.000 | 769 | tags=73%, list=55%, signal=158% |
| 2224 | GSE36888\_STAT5\_AB\_KNOCKIN\_VS\_WT\_TCELL\_IL2\_TREATED\_6H\_UP |  | 24 | -0.13 | -0.64 | 0.898 | 0.963 | 1.000 | 1021 | tags=92%, list=72%, signal=326% |
| 2225 | GSE3720\_UNSTIM\_VS\_LPS\_STIM\_VD2\_GAMMADELTA\_TCELL\_UP |  | 16 | -0.15 | -0.64 | 0.886 | 0.963 | 1.000 | 1197 | tags=100%, list=85%, signal=652% |
| 2226 | GSE17186\_NAIVE\_VS\_CD21LOW\_TRANSITIONAL\_BCELL\_CORD\_BLOOD\_DN |  | 18 | -0.15 | -0.64 | 0.871 | 0.963 | 1.000 | 70 | tags=11%, list=5%, signal=12% |
| 2227 | GSE42724\_MEMORY\_BCELL\_VS\_PLASMABLAST\_UP |  | 21 | -0.13 | -0.64 | 0.912 | 0.963 | 1.000 | 483 | tags=52%, list=34%, signal=78% |
| 2228 | GSE6269\_STAPH\_AUREUS\_VS\_STREP\_PNEUMO\_INF\_PBMC\_UP |  | 24 | -0.13 | -0.64 | 0.891 | 0.963 | 1.000 | 395 | tags=38%, list=28%, signal=51% |
| 2229 | GSE14000\_UNSTIM\_VS\_16H\_LPS\_DC\_TRANSLATED\_RNA\_DN |  | 19 | -0.15 | -0.64 | 0.882 | 0.963 | 1.000 | 547 | tags=58%, list=39%, signal=93% |
| 2230 | GSE17721\_0.5H\_VS\_8H\_LPS\_BMDC\_DN |  | 19 | -0.14 | -0.64 | 0.902 | 0.963 | 1.000 | 538 | tags=58%, list=38%, signal=92% |
| 2231 | GSE12845\_NAIVE\_VS\_PRE\_GC\_TONSIL\_BCELL\_DN |  | 17 | -0.15 | -0.64 | 0.901 | 0.963 | 1.000 | 767 | tags=76%, list=54%, signal=166% |
| 2232 | GSE28737\_FOLLICULAR\_VS\_MARGINAL\_ZONE\_BCELL\_BCL6\_HET\_UP |  | 19 | -0.14 | -0.64 | 0.898 | 0.964 | 1.000 | 1089 | tags=95%, list=77%, signal=410% |
| 2233 | GOLDRATH\_EFF\_VS\_MEMORY\_CD8\_TCELL\_DN |  | 27 | -0.12 | -0.64 | 0.911 | 0.963 | 1.000 | 71 | tags=11%, list=5%, signal=11% |
| 2234 | GSE17721\_12H\_VS\_24H\_LPS\_BMDC\_DN |  | 19 | -0.14 | -0.64 | 0.916 | 0.963 | 1.000 | 1009 | tags=89%, list=72%, signal=310% |
| 2235 | GSE13485\_DAY3\_VS\_DAY7\_YF17D\_VACCINE\_PBMC\_DN |  | 22 | -0.13 | -0.64 | 0.890 | 0.963 | 1.000 | 546 | tags=55%, list=39%, signal=88% |
| 2236 | GSE3039\_ALPHAALPHA\_VS\_ALPHABETA\_CD8\_TCELL\_UP |  | 32 | -0.12 | -0.64 | 0.941 | 0.963 | 1.000 | 157 | tags=16%, list=11%, signal=17% |
| 2237 | GSE37605\_C57BL6\_VS\_NOD\_FOXP3\_IRES\_GFP\_TCONV\_DN |  | 18 | -0.14 | -0.64 | 0.898 | 0.963 | 1.000 | 1214 | tags=100%, list=86%, signal=707% |
| 2238 | GSE36009\_WT\_VS\_NLRP10\_KO\_DC\_UP |  | 21 | -0.14 | -0.64 | 0.897 | 0.963 | 1.000 | 105 | tags=14%, list=7%, signal=15% |
| 2239 | GSE360\_L\_MAJOR\_VS\_M\_TUBERCULOSIS\_DC\_UP |  | 22 | -0.13 | -0.64 | 0.906 | 0.963 | 1.000 | 66 | tags=9%, list=5%, signal=9% |
| 2240 | GSE32901\_NAIVE\_VS\_TH1\_CD4\_TCELL\_DN |  | 15 | -0.15 | -0.63 | 0.912 | 0.964 | 1.000 | 1201 | tags=100%, list=85%, signal=665% |
| 2241 | GSE25123\_IL4\_VS\_IL4\_AND\_ROSIGLITAZONE\_STIM\_MACROPHAGE\_DAY10\_DN |  | 17 | -0.15 | -0.63 | 0.912 | 0.964 | 1.000 | 57 | tags=12%, list=4%, signal=12% |
| 2242 | GSE16451\_IMMATURE\_VS\_MATURE\_NEURON\_CELL\_LINE\_WEST\_EQUINE\_ENC\_VIRUS\_UP |  | 24 | -0.13 | -0.63 | 0.913 | 0.965 | 1.000 | 395 | tags=38%, list=28%, signal=51% |
| 2243 | GSE16385\_UNTREATED\_VS\_12H\_ROSIGLITAZONE\_IFNG\_TNF\_TREATED\_MACROPHAGE\_DN |  | 21 | -0.14 | -0.63 | 0.884 | 0.965 | 1.000 | 67 | tags=10%, list=5%, signal=10% |
| 2244 | GSE40274\_FOXP3\_VS\_FOXP3\_AND\_XBP1\_TRANSDUCED\_ACTIVATED\_CD4\_TCELL\_DN |  | 15 | -0.16 | -0.63 | 0.898 | 0.964 | 1.000 | 430 | tags=47%, list=30%, signal=66% |
| 2245 | GSE339\_CD4POS\_VS\_CD4CD8DN\_DC\_UP |  | 18 | -0.15 | -0.63 | 0.919 | 0.964 | 1.000 | 79 | tags=11%, list=6%, signal=12% |
| 2246 | GSE13306\_TREG\_VS\_TCONV\_DN |  | 20 | -0.13 | -0.63 | 0.926 | 0.966 | 1.000 | 1225 | tags=100%, list=87%, signal=748% |
| 2247 | GSE17721\_LPS\_VS\_CPG\_8H\_BMDC\_DN |  | 16 | -0.15 | -0.63 | 0.899 | 0.965 | 1.000 | 1200 | tags=100%, list=85%, signal=661% |
| 2248 | GSE3982\_NKCELL\_VS\_TH1\_DN |  | 23 | -0.13 | -0.63 | 0.909 | 0.965 | 1.000 | 700 | tags=70%, list=50%, signal=136% |
| 2249 | GSE17721\_CTRL\_VS\_PAM3CSK4\_2H\_BMDC\_DN |  | 17 | -0.15 | -0.63 | 0.893 | 0.965 | 1.000 | 287 | tags=29%, list=20%, signal=36% |
| 2250 | GSE25123\_WT\_VS\_PPARG\_KO\_MACROPHAGE\_DN |  | 18 | -0.15 | -0.63 | 0.884 | 0.965 | 1.000 | 319 | tags=28%, list=23%, signal=35% |
| 2251 | GSE14908\_ATOPIC\_VS\_NONATOPIC\_PATIENT\_RESTING\_CD4\_TCELL\_DN |  | 20 | -0.14 | -0.63 | 0.928 | 0.965 | 1.000 | 1221 | tags=100%, list=87%, signal=732% |
| 2252 | GSE30962\_ACUTE\_VS\_CHRONIC\_LCMV\_SECONDARY\_INF\_CD8\_TCELL\_UP |  | 19 | -0.14 | -0.63 | 0.905 | 0.965 | 1.000 | 510 | tags=53%, list=36%, signal=81% |
| 2253 | GSE24972\_WT\_VS\_IRF8\_KO\_SPLEEN\_FOLLICULAR\_BCELL\_UP |  | 22 | -0.14 | -0.63 | 0.924 | 0.965 | 1.000 | 868 | tags=82%, list=62%, signal=209% |
| 2254 | GSE29615\_DAY3\_VS\_DAY7\_LAIV\_FLU\_VACCINE\_PBMC\_DN |  | 15 | -0.15 | -0.63 | 0.928 | 0.966 | 1.000 | 225 | tags=20%, list=16%, signal=24% |
| 2255 | GSE2405\_HEAT\_KILLED\_LYSATE\_VS\_LIVE\_A\_PHAGOCYTOPHILUM\_STIM\_NEUTROPHIL\_9H\_UP |  | 18 | -0.14 | -0.62 | 0.911 | 0.966 | 1.000 | 315 | tags=33%, list=22%, signal=42% |
| 2256 | GSE11961\_PLASMA\_CELL\_DAY7\_VS\_MEMORY\_BCELL\_DAY40\_UP |  | 19 | -0.14 | -0.62 | 0.919 | 0.966 | 1.000 | 1216 | tags=100%, list=86%, signal=714% |
| 2257 | GSE21063\_WT\_VS\_NFATC1\_KO\_BCELL\_DN |  | 24 | -0.13 | -0.62 | 0.916 | 0.966 | 1.000 | 52 | tags=8%, list=4%, signal=9% |
| 2258 | GSE40666\_UNTREATED\_VS\_IFNA\_STIM\_EFFECTOR\_CD8\_TCELL\_90MIN\_UP |  | 16 | -0.15 | -0.62 | 0.914 | 0.965 | 1.000 | 547 | tags=56%, list=39%, signal=91% |
| 2259 | GSE2826\_WT\_VS\_XID\_BCELL\_UP |  | 22 | -0.13 | -0.62 | 0.925 | 0.965 | 1.000 | 387 | tags=36%, list=27%, signal=49% |
| 2260 | GSE17721\_CTRL\_VS\_CPG\_8H\_BMDC\_UP |  | 22 | -0.13 | -0.62 | 0.933 | 0.965 | 1.000 | 385 | tags=36%, list=27%, signal=49% |
| 2261 | GSE7548\_NAIVE\_VS\_DAY28\_PCC\_IMMUNIZATION\_CD4\_TCELL\_UP |  | 28 | -0.12 | -0.62 | 0.897 | 0.965 | 1.000 | 391 | tags=39%, list=28%, signal=53% |
| 2262 | GSE21033\_CTRL\_VS\_POLYIC\_STIM\_DC\_12H\_UP |  | 17 | -0.14 | -0.62 | 0.901 | 0.965 | 1.000 | 210 | tags=24%, list=15%, signal=27% |
| 2263 | GSE37301\_LYMPHOID\_PRIMED\_MPP\_VS\_GRAN\_MONO\_PROGENITOR\_UP |  | 27 | -0.12 | -0.62 | 0.922 | 0.965 | 1.000 | 321 | tags=30%, list=23%, signal=38% |
| 2264 | GSE40274\_LEF1\_VS\_FOXP3\_AND\_LEF1\_TRANSDUCED\_ACTIVATED\_CD4\_TCELL\_DN |  | 29 | -0.12 | -0.62 | 0.912 | 0.965 | 1.000 | 1250 | tags=100%, list=89%, signal=858% |
| 2265 | GSE24142\_ADULT\_VS\_FETAL\_EARLY\_THYMIC\_PROGENITOR\_DN |  | 18 | -0.14 | -0.62 | 0.884 | 0.965 | 1.000 | 49 | tags=11%, list=3%, signal=11% |
| 2266 | GSE557\_WT\_VS\_I\_AB\_KO\_DC\_UP |  | 17 | -0.15 | -0.62 | 0.893 | 0.965 | 1.000 | 466 | tags=47%, list=33%, signal=69% |
| 2267 | GSE13484\_UNSTIM\_VS\_3H\_YF17D\_VACCINE\_STIM\_PBMC\_UP |  | 18 | -0.14 | -0.62 | 0.924 | 0.965 | 1.000 | 271 | tags=28%, list=19%, signal=34% |
| 2268 | GSE11961\_GERMINAL\_CENTER\_BCELL\_DAY7\_VS\_PLASMA\_CELL\_DAY7\_UP |  | 21 | -0.14 | -0.62 | 0.920 | 0.965 | 1.000 | 222 | tags=24%, list=16%, signal=28% |
| 2269 | GSE17974\_0.5H\_VS\_72H\_UNTREATED\_IN\_VITRO\_CD4\_TCELL\_UP |  | 24 | -0.13 | -0.62 | 0.906 | 0.965 | 1.000 | 528 | tags=54%, list=37%, signal=85% |
| 2270 | GSE8685\_IL2\_ACT\_IL2\_STARVED\_VS\_IL21\_ACT\_IL2\_STARVED\_CD4\_TCELL\_DN |  | 16 | -0.15 | -0.62 | 0.918 | 0.966 | 1.000 | 272 | tags=31%, list=19%, signal=38% |
| 2271 | GSE21927\_BALBC\_VS\_C57BL6\_MONOCYTE\_TUMOR\_DN |  | 20 | -0.13 | -0.61 | 0.921 | 0.968 | 1.000 | 67 | tags=10%, list=5%, signal=10% |
| 2272 | GSE17721\_CTRL\_VS\_PAM3CSK4\_24H\_BMDC\_DN |  | 20 | -0.13 | -0.61 | 0.930 | 0.967 | 1.000 | 373 | tags=35%, list=26%, signal=47% |
| 2273 | GSE37301\_MULTIPOTENT\_PROGENITOR\_VS\_RAG2\_KO\_NK\_CELL\_DN |  | 17 | -0.15 | -0.61 | 0.901 | 0.967 | 1.000 | 735 | tags=76%, list=52%, signal=158% |
| 2274 | GSE17721\_12H\_VS\_24H\_GARDIQUIMOD\_BMDC\_UP |  | 19 | -0.14 | -0.61 | 0.932 | 0.968 | 1.000 | 873 | tags=84%, list=62%, signal=218% |
| 2275 | GSE1432\_CTRL\_VS\_IFNG\_6H\_MICROGLIA\_UP |  | 20 | -0.13 | -0.61 | 0.940 | 0.968 | 1.000 | 270 | tags=25%, list=19%, signal=30% |
| 2276 | GSE21546\_WT\_VS\_SAP1A\_KO\_DP\_THYMOCYTES\_UP |  | 29 | -0.11 | -0.61 | 0.940 | 0.969 | 1.000 | 1179 | tags=97%, list=84%, signal=575% |
| 2277 | GSE20727\_CTRL\_VS\_DNFB\_ALLERGEN\_TREATED\_DC\_UP |  | 15 | -0.15 | -0.61 | 0.940 | 0.969 | 1.000 | 928 | tags=87%, list=66%, signal=250% |
| 2278 | GSE17721\_LPS\_VS\_PAM3CSK4\_6H\_BMDC\_UP |  | 21 | -0.13 | -0.61 | 0.934 | 0.969 | 1.000 | 538 | tags=57%, list=38%, signal=91% |
| 2279 | GSE32986\_UNSTIM\_VS\_CURDLAN\_HIGHDOSE\_STIM\_DC\_UP |  | 25 | -0.13 | -0.61 | 0.929 | 0.969 | 1.000 | 1132 | tags=96%, list=80%, signal=477% |
| 2280 | GSE5099\_UNSTIM\_VS\_MCSF\_TREATED\_MONOCYTE\_DAY7\_DN |  | 15 | -0.15 | -0.61 | 0.933 | 0.969 | 1.000 | 1200 | tags=100%, list=85%, signal=662% |
| 2281 | GSE19941\_IL10\_KO\_VS\_IL10\_KO\_AND\_NFKBP50\_KO\_LPS\_STIM\_MACROPHAGE\_DN |  | 16 | -0.15 | -0.61 | 0.913 | 0.969 | 1.000 | 833 | tags=81%, list=59%, signal=196% |
| 2282 | GSE22886\_NAIVE\_CD4\_TCELL\_VS\_48H\_ACT\_TH1\_DN |  | 22 | -0.13 | -0.61 | 0.932 | 0.970 | 1.000 | 849 | tags=82%, list=60%, signal=202% |
| 2283 | GSE3982\_EOSINOPHIL\_VS\_NEUTROPHIL\_DN |  | 20 | -0.13 | -0.61 | 0.946 | 0.970 | 1.000 | 1231 | tags=100%, list=87%, signal=773% |
| 2284 | GSE2706\_2H\_VS\_8H\_LPS\_STIM\_DC\_UP |  | 18 | -0.14 | -0.60 | 0.923 | 0.970 | 1.000 | 481 | tags=50%, list=34%, signal=75% |
| 2285 | GSE23308\_CTRL\_VS\_CORTICOSTERONE\_TREATED\_MACROPHAGE\_MINERALCORTICOID\_REC\_KO\_UP |  | 16 | -0.14 | -0.60 | 0.938 | 0.971 | 1.000 | 1050 | tags=94%, list=74%, signal=362% |
| 2286 | GSE17721\_0.5H\_VS\_24H\_GARDIQUIMOD\_BMDC\_DN |  | 20 | -0.13 | -0.60 | 0.944 | 0.971 | 1.000 | 753 | tags=75%, list=53%, signal=159% |
| 2287 | GSE17721\_LPS\_VS\_GARDIQUIMOD\_24H\_BMDC\_UP |  | 17 | -0.14 | -0.60 | 0.938 | 0.972 | 1.000 | 342 | tags=35%, list=24%, signal=46% |
| 2288 | GSE14699\_NAIVE\_VS\_ACT\_CD8\_TCELL\_DN |  | 27 | -0.12 | -0.60 | 0.917 | 0.972 | 1.000 | 443 | tags=41%, list=31%, signal=58% |
| 2289 | GSE29617\_DAY3\_VS\_DAY7\_TIV\_FLU\_VACCINE\_PBMC\_2008\_DN |  | 17 | -0.14 | -0.60 | 0.946 | 0.973 | 1.000 | 441 | tags=47%, list=31%, signal=68% |
| 2290 | GSE7460\_CTRL\_VS\_FOXP3\_OVEREXPR\_TCONV\_DN |  | 19 | -0.13 | -0.60 | 0.928 | 0.974 | 1.000 | 58 | tags=11%, list=4%, signal=11% |
| 2291 | GSE6259\_FLT3L\_INDUCED\_DEC205\_POS\_DC\_VS\_CD8\_TCELL\_DN |  | 18 | -0.13 | -0.59 | 0.923 | 0.975 | 1.000 | 217 | tags=22%, list=15%, signal=26% |
| 2292 | GSE14350\_TREG\_VS\_TEFF\_UP |  | 23 | -0.13 | -0.59 | 0.950 | 0.976 | 1.000 | 251 | tags=26%, list=18%, signal=31% |
| 2293 | GSE3039\_CD4\_TCELL\_VS\_ALPHAALPHA\_CD8\_TCELL\_UP |  | 20 | -0.13 | -0.59 | 0.950 | 0.976 | 1.000 | 1231 | tags=100%, list=87%, signal=773% |
| 2294 | GSE3982\_MAST\_CELL\_VS\_CENT\_MEMORY\_CD4\_TCELL\_UP |  | 19 | -0.13 | -0.59 | 0.955 | 0.977 | 1.000 | 742 | tags=74%, list=53%, signal=153% |
| 2295 | GSE39820\_CTRL\_VS\_IL1B\_IL6\_IL23A\_CD4\_TCELL\_UP |  | 26 | -0.12 | -0.59 | 0.942 | 0.979 | 1.000 | 806 | tags=77%, list=57%, signal=176% |
| 2296 | GSE2128\_C57BL6\_VS\_NOD\_THYMOCYTE\_DN |  | 15 | -0.14 | -0.59 | 0.922 | 0.978 | 1.000 | 1212 | tags=100%, list=86%, signal=702% |
| 2297 | GSE3337\_CTRL\_VS\_16H\_IFNG\_IN\_CD8POS\_DC\_DN |  | 21 | -0.12 | -0.58 | 0.965 | 0.979 | 1.000 | 602 | tags=62%, list=43%, signal=106% |
| 2298 | GSE15659\_RESTING\_TREG\_VS\_NONSUPPRESSIVE\_TCELL\_DN |  | 17 | -0.14 | -0.58 | 0.935 | 0.979 | 1.000 | 287 | tags=29%, list=20%, signal=36% |
| 2299 | GSE27291\_0H\_VS\_7D\_STIM\_GAMMADELTA\_TCELL\_DN |  | 27 | -0.11 | -0.58 | 0.968 | 0.979 | 1.000 | 1253 | tags=100%, list=89%, signal=876% |
| 2300 | GSE17721\_POLYIC\_VS\_CPG\_16H\_BMDC\_DN |  | 18 | -0.13 | -0.58 | 0.955 | 0.978 | 1.000 | 156 | tags=17%, list=11%, signal=18% |
| 2301 | GSE17721\_LPS\_VS\_GARDIQUIMOD\_12H\_BMDC\_UP |  | 18 | -0.13 | -0.58 | 0.932 | 0.978 | 1.000 | 398 | tags=39%, list=28%, signal=53% |
| 2302 | GSE27786\_CD8\_TCELL\_VS\_MONO\_MAC\_DN |  | 16 | -0.14 | -0.58 | 0.936 | 0.978 | 1.000 | 515 | tags=56%, list=36%, signal=88% |
| 2303 | GSE36078\_UNTREATED\_VS\_AD5\_INF\_MOUSE\_LUNG\_DC\_DN |  | 21 | -0.13 | -0.58 | 0.946 | 0.977 | 1.000 | 1235 | tags=100%, list=88%, signal=790% |
| 2304 | GSE2128\_C57BL6\_VS\_NOD\_THYMOCYTE\_UP |  | 20 | -0.13 | -0.58 | 0.956 | 0.977 | 1.000 | 126 | tags=15%, list=9%, signal=16% |
| 2305 | GSE16385\_UNTREATED\_VS\_12H\_ROSIGLITAZONE\_IL4\_TREATED\_MACROPHAGE\_UP |  | 23 | -0.12 | -0.58 | 0.943 | 0.977 | 1.000 | 1248 | tags=100%, list=88%, signal=852% |
| 2306 | GSE10240\_CTRL\_VS\_IL22\_STIM\_PRIMARY\_BRONCHIAL\_EPITHELIAL\_CELLS\_UP |  | 21 | -0.13 | -0.58 | 0.935 | 0.977 | 1.000 | 443 | tags=43%, list=31%, signal=62% |
| 2307 | GSE40274\_FOXP3\_VS\_FOXP3\_AND\_PBX1\_TRANSDUCED\_ACTIVATED\_CD4\_TCELL\_DN |  | 20 | -0.13 | -0.58 | 0.943 | 0.977 | 1.000 | 251 | tags=25%, list=18%, signal=30% |
| 2308 | GSE17721\_PAM3CSK4\_VS\_CPG\_8H\_BMDC\_DN |  | 22 | -0.12 | -0.58 | 0.945 | 0.977 | 1.000 | 557 | tags=55%, list=39%, signal=89% |
| 2309 | GSE21063\_WT\_VS\_NFATC1\_KO\_8H\_ANTI\_IGM\_STIM\_BCELL\_DN |  | 23 | -0.12 | -0.58 | 0.966 | 0.977 | 1.000 | 429 | tags=39%, list=30%, signal=55% |
| 2310 | GSE6269\_E\_COLI\_VS\_STREP\_PNEUMO\_INF\_PBMC\_UP |  | 15 | -0.14 | -0.58 | 0.951 | 0.977 | 1.000 | 1052 | tags=93%, list=75%, signal=363% |
| 2311 | GSE360\_CTRL\_VS\_L\_MAJOR\_DC\_UP |  | 16 | -0.14 | -0.58 | 0.949 | 0.977 | 1.000 | 907 | tags=88%, list=64%, signal=242% |
| 2312 | GSE15930\_STIM\_VS\_STIM\_AND\_IL12\_48H\_CD8\_T\_CELL\_UP |  | 17 | -0.13 | -0.58 | 0.957 | 0.977 | 1.000 | 517 | tags=53%, list=37%, signal=83% |
| 2313 | GSE17721\_PAM3CSK4\_VS\_GADIQUIMOD\_2H\_BMDC\_DN |  | 29 | -0.11 | -0.58 | 0.955 | 0.978 | 1.000 | 1148 | tags=97%, list=81%, signal=507% |
| 2314 | GSE22935\_WT\_VS\_MYD88\_KO\_MACROPHAGE\_48H\_MBOVIS\_BCG\_STIM\_UP |  | 20 | -0.13 | -0.57 | 0.956 | 0.978 | 1.000 | 500 | tags=50%, list=35%, signal=76% |
| 2315 | GSE10211\_UV\_INACT\_SENDAI\_VS\_LIVE\_SENDAI\_VIRUS\_TRACHEAL\_EPITHELIAL\_CELLS\_UP |  | 25 | -0.11 | -0.57 | 0.958 | 0.980 | 1.000 | 1254 | tags=100%, list=89%, signal=883% |
| 2316 | GSE20727\_CTRL\_VS\_ROS\_INHIBITOR\_TREATED\_DC\_DN |  | 24 | -0.12 | -0.57 | 0.960 | 0.982 | 1.000 | 42 | tags=8%, list=3%, signal=8% |
| 2317 | GSE360\_L\_DONOVANI\_VS\_T\_GONDII\_DC\_DN |  | 17 | -0.13 | -0.57 | 0.951 | 0.983 | 1.000 | 1226 | tags=100%, list=87%, signal=754% |
| 2318 | GSE13411\_IGM\_VS\_SWITCHED\_MEMORY\_BCELL\_DN |  | 28 | -0.11 | -0.56 | 0.952 | 0.982 | 1.000 | 1020 | tags=89%, list=72%, signal=316% |
| 2319 | GSE31082\_DP\_VS\_CD8\_SP\_THYMOCYTE\_UP |  | 19 | -0.12 | -0.56 | 0.962 | 0.982 | 1.000 | 332 | tags=32%, list=24%, signal=41% |
| 2320 | GSE40068\_CXCR5NEG\_BCL6NEG\_CD4\_TCELL\_VS\_CXCR5POS\_BCL6NEG\_TFH\_UP |  | 16 | -0.13 | -0.56 | 0.970 | 0.983 | 1.000 | 396 | tags=38%, list=28%, signal=52% |
| 2321 | GSE360\_L\_DONOVANI\_VS\_M\_TUBERCULOSIS\_DC\_DN |  | 18 | -0.12 | -0.56 | 0.973 | 0.984 | 1.000 | 218 | tags=22%, list=15%, signal=26% |
| 2322 | GSE5542\_IFNA\_VS\_IFNA\_AND\_IFNG\_TREATED\_EPITHELIAL\_CELLS\_6H\_DN |  | 25 | -0.11 | -0.56 | 0.956 | 0.984 | 1.000 | 316 | tags=28%, list=22%, signal=35% |
| 2323 | GSE3982\_EFF\_MEMORY\_VS\_CENT\_MEMORY\_CD4\_TCELL\_DN |  | 21 | -0.12 | -0.56 | 0.953 | 0.984 | 1.000 | 485 | tags=48%, list=34%, signal=71% |
| 2324 | GSE29164\_CD8\_TCELL\_VS\_CD8\_TCELL\_AND\_IL12\_TREATED\_MELANOMA\_DAY3\_UP |  | 30 | -0.11 | -0.56 | 0.946 | 0.984 | 1.000 | 438 | tags=40%, list=31%, signal=57% |
| 2325 | GSE360\_T\_GONDII\_VS\_M\_TUBERCULOSIS\_MAC\_DN |  | 22 | -0.12 | -0.56 | 0.958 | 0.984 | 1.000 | 432 | tags=41%, list=31%, signal=58% |
| 2326 | GSE2585\_THYMIC\_DC\_VS\_MTEC\_UP |  | 17 | -0.13 | -0.55 | 0.960 | 0.986 | 1.000 | 230 | tags=24%, list=16%, signal=28% |
| 2327 | GSE20151\_CTRL\_VS\_FUSOBACT\_NUCLEATUM\_NEUTROPHIL\_UP |  | 26 | -0.11 | -0.55 | 0.970 | 0.986 | 1.000 | 49 | tags=8%, list=3%, signal=8% |
| 2328 | GSE9988\_ANTI\_TREM1\_VS\_VEHICLE\_TREATED\_MONOCYTES\_DN |  | 22 | -0.11 | -0.55 | 0.963 | 0.986 | 1.000 | 173 | tags=18%, list=12%, signal=20% |
| 2329 | GSE37301\_MULTIPOTENT\_PROGENITOR\_VS\_GRAN\_MONO\_PROGENITOR\_UP |  | 30 | -0.10 | -0.55 | 0.967 | 0.986 | 1.000 | 1194 | tags=97%, list=85%, signal=615% |
| 2330 | GSE34156\_UNTREATED\_VS\_6H\_NOD2\_AND\_TLR1\_TLR2\_LIGAND\_TREATED\_MONOCYTE\_DN |  | 15 | -0.13 | -0.55 | 0.975 | 0.988 | 1.000 | 1227 | tags=100%, list=87%, signal=759% |
| 2331 | GSE10273\_HIGH\_VS\_LOW\_IL7\_TREATED\_IRF4\_8\_NULL\_PRE\_BCELL\_DN |  | 24 | -0.11 | -0.54 | 0.964 | 0.988 | 1.000 | 284 | tags=25%, list=20%, signal=31% |
| 2332 | GSE3982\_MAST\_CELL\_VS\_EFF\_MEMORY\_CD4\_TCELL\_UP |  | 29 | -0.10 | -0.54 | 0.969 | 0.989 | 1.000 | 535 | tags=52%, list=38%, signal=82% |
| 2333 | GSE21546\_UNSTIM\_VS\_ANTI\_CD3\_STIM\_SAP1A\_KO\_AND\_ELK1\_KO\_DP\_THYMOCYTES\_UP |  | 18 | -0.12 | -0.54 | 0.973 | 0.990 | 1.000 | 456 | tags=44%, list=32%, signal=65% |
| 2334 | GSE1448\_CTRL\_VS\_ANTI\_VALPHA2\_DP\_THYMOCYTE\_UP |  | 17 | -0.13 | -0.54 | 0.961 | 0.990 | 1.000 | 114 | tags=12%, list=8%, signal=13% |
| 2335 | GSE17721\_CPG\_VS\_GARDIQUIMOD\_6H\_BMDC\_DN |  | 23 | -0.11 | -0.54 | 0.968 | 0.990 | 1.000 | 54 | tags=9%, list=4%, signal=9% |
| 2336 | GSE13229\_IMM\_VS\_MATURE\_NKCELL\_UP |  | 19 | -0.12 | -0.54 | 0.977 | 0.990 | 1.000 | 147 | tags=16%, list=10%, signal=17% |
| 2337 | GSE2770\_TGFB\_AND\_IL4\_VS\_IL12\_TREATED\_ACT\_CD4\_TCELL\_6H\_DN |  | 18 | -0.12 | -0.53 | 0.972 | 0.989 | 1.000 | 438 | tags=44%, list=31%, signal=64% |
| 2338 | GSE13522\_CTRL\_VS\_T\_CRUZI\_Y\_STRAIN\_INF\_SKIN\_BALBC\_MOUSE\_UP |  | 18 | -0.12 | -0.53 | 0.972 | 0.989 | 1.000 | 13 | tags=6%, list=1%, signal=6% |
| 2339 | GSE13484\_UNSTIM\_VS\_12H\_YF17D\_VACCINE\_STIM\_PBMC\_UP |  | 19 | -0.12 | -0.53 | 0.967 | 0.990 | 1.000 | 1136 | tags=95%, list=81%, signal=480% |
| 2340 | GSE36009\_WT\_VS\_NLRP10\_KO\_DC\_DN |  | 35 | -0.09 | -0.53 | 0.974 | 0.990 | 1.000 | 294 | tags=26%, list=21%, signal=32% |
| 2341 | GSE22886\_NAIVE\_VS\_MEMORY\_TCELL\_DN |  | 18 | -0.12 | -0.53 | 0.954 | 0.990 | 1.000 | 213 | tags=22%, list=15%, signal=26% |
| 2342 | GSE16522\_ANTI\_CD3CD28\_STIM\_VS\_UNSTIM\_NAIVE\_CD8\_TCELL\_DN |  | 16 | -0.12 | -0.53 | 0.977 | 0.990 | 1.000 | 1240 | tags=100%, list=88%, signal=816% |
| 2343 | GSE18804\_SPLEEN\_MACROPHAGE\_VS\_BRAIN\_TUMORAL\_MACROPHAGE\_UP |  | 21 | -0.11 | -0.53 | 0.978 | 0.990 | 1.000 | 549 | tags=57%, list=39%, signal=92% |
| 2344 | GSE9878\_CTRL\_VS\_EBF\_TRANSDUCED\_PAX5\_KO\_PRO\_BCELL\_DN |  | 27 | -0.11 | -0.52 | 0.968 | 0.991 | 1.000 | 1264 | tags=100%, list=90%, signal=941% |
| 2345 | GSE34156\_UNTREATED\_VS\_6H\_TLR1\_TLR2\_LIGAND\_TREATED\_MONOCYTE\_DN |  | 19 | -0.12 | -0.52 | 0.973 | 0.991 | 1.000 | 759 | tags=74%, list=54%, signal=157% |
| 2346 | GSE11961\_MEMORY\_BCELL\_DAY40\_VS\_GERMINAL\_CENTER\_BCELL\_DAY40\_UP |  | 21 | -0.11 | -0.52 | 0.969 | 0.992 | 1.000 | 155 | tags=14%, list=11%, signal=16% |
| 2347 | GSE37533\_UNTREATED\_VS\_PIOGLIZATONE\_TREATED\_CD4\_TCELL\_PPARG1\_AND\_FOXP3\_TRASDUCED\_DN |  | 21 | -0.11 | -0.52 | 0.969 | 0.991 | 1.000 | 880 | tags=81%, list=62%, signal=212% |
| 2348 | GSE360\_L\_DONOVANI\_VS\_B\_MALAYI\_HIGH\_DOSE\_MAC\_UP |  | 26 | -0.10 | -0.52 | 0.985 | 0.992 | 1.000 | 1158 | tags=96%, list=82%, signal=526% |
| 2349 | GSE6259\_FLT3L\_INDUCED\_DEC205\_POS\_DC\_VS\_BCELL\_DN |  | 17 | -0.12 | -0.52 | 0.979 | 0.992 | 1.000 | 411 | tags=41%, list=29%, signal=57% |
| 2350 | GSE22611\_MUTANT\_NOD2\_TRANSDUCED\_VS\_CTRL\_HEK293T\_STIMULATED\_WITH\_MDP\_6H\_DN |  | 22 | -0.11 | -0.52 | 0.983 | 0.991 | 1.000 | 376 | tags=36%, list=27%, signal=49% |
| 2351 | GSE43863\_LY6C\_INT\_CXCR5POS\_VS\_LY6C\_LOW\_CXCR5NEG\_EFFECTOR\_CD4\_TCELL\_UP |  | 15 | -0.13 | -0.51 | 0.976 | 0.992 | 1.000 | 1097 | tags=93%, list=78%, signal=415% |
| 2352 | GSE17721\_CTRL\_VS\_GARDIQUIMOD\_24H\_BMDC\_DN |  | 19 | -0.11 | -0.51 | 0.991 | 0.993 | 1.000 | 1252 | tags=100%, list=89%, signal=875% |
| 2353 | GSE2770\_IL12\_ACT\_VS\_ACT\_CD4\_TCELL\_48H\_UP |  | 17 | -0.12 | -0.51 | 0.995 | 0.992 | 1.000 | 591 | tags=59%, list=42%, signal=100% |
| 2354 | GSE25123\_CTRL\_VS\_IL4\_AND\_ROSIGLITAZONE\_STIM\_PPARG\_KO\_MACROPHAGE\_DN |  | 26 | -0.10 | -0.51 | 0.988 | 0.992 | 1.000 | 292 | tags=27%, list=21%, signal=33% |
| 2355 | GSE14699\_NAIVE\_VS\_DELETIONAL\_TOLERANCE\_CD8\_TCELL\_DN |  | 22 | -0.11 | -0.51 | 0.980 | 0.992 | 1.000 | 455 | tags=41%, list=32%, signal=59% |
| 2356 | GSE25088\_ROSIGLITAZONE\_VS\_IL4\_AND\_ROSIGLITAZONE\_STIM\_STAT6\_KO\_MACROPHAGE\_DAY10\_DN |  | 24 | -0.10 | -0.51 | 0.980 | 0.992 | 1.000 | 66 | tags=8%, list=5%, signal=9% |
| 2357 | GSE26351\_UNSTIM\_VS\_BMP\_PATHWAY\_STIM\_HEMATOPOIETIC\_PROGENITORS\_UP |  | 17 | -0.12 | -0.51 | 0.987 | 0.992 | 1.000 | 105 | tags=12%, list=7%, signal=13% |
| 2358 | GSE14308\_TH17\_VS\_NATURAL\_TREG\_DN |  | 18 | -0.11 | -0.50 | 0.984 | 0.992 | 1.000 | 80 | tags=11%, list=6%, signal=12% |
| 2359 | GSE16450\_IMMATURE\_VS\_MATURE\_NEURON\_CELL\_LINE\_12H\_IFNA\_STIM\_UP |  | 20 | -0.11 | -0.50 | 0.978 | 0.993 | 1.000 | 1124 | tags=95%, list=80%, signal=460% |
| 2360 | GSE25088\_ROSIGLITAZONE\_VS\_IL4\_AND\_ROSIGLITAZONE\_STIM\_MACROPHAGE\_DAY10\_DN |  | 27 | -0.09 | -0.50 | 0.985 | 0.993 | 1.000 | 308 | tags=26%, list=22%, signal=33% |
| 2361 | GSE21546\_ELK1\_KO\_VS\_SAP1A\_KO\_AND\_ELK1\_KO\_DP\_THYMOCYTES\_UP |  | 33 | -0.09 | -0.50 | 0.992 | 0.993 | 1.000 | 1288 | tags=100%, list=91%, signal=1120% |
| 2362 | GSE19888\_ADENOSINE\_A3R\_INH\_PRETREAT\_AND\_ACT\_BY\_A3R\_VS\_A3R\_INH\_AND\_TCELL\_MEMBRANES\_ACT\_MAST\_CELL\_DN |  | 22 | -0.10 | -0.50 | 0.984 | 0.993 | 1.000 | 476 | tags=45%, list=34%, signal=68% |
| 2363 | GSE43863\_TFH\_VS\_LY6C\_LOW\_CXCR5NEG\_EFFECTOR\_CD4\_TCELL\_UP |  | 22 | -0.10 | -0.50 | 0.975 | 0.992 | 1.000 | 828 | tags=77%, list=59%, signal=184% |
| 2364 | GSE2770\_IL12\_VS\_IL4\_TREATED\_ACT\_CD4\_TCELL\_6H\_UP |  | 16 | -0.12 | -0.49 | 0.983 | 0.992 | 1.000 | 965 | tags=88%, list=68%, signal=274% |
| 2365 | GSE22601\_DOUBLE\_POSITIVE\_VS\_CD8\_SINGLE\_POSITIVE\_THYMOCYTE\_DN |  | 18 | -0.11 | -0.49 | 0.981 | 0.993 | 1.000 | 868 | tags=83%, list=62%, signal=214% |
| 2366 | GSE2770\_UNTREATED\_VS\_IL4\_TREATED\_ACT\_CD4\_TCELL\_2H\_UP |  | 17 | -0.12 | -0.49 | 0.982 | 0.994 | 1.000 | 904 | tags=82%, list=64%, signal=226% |
| 2367 | GSE21360\_PRIMARY\_VS\_SECONDARY\_MEMORY\_CD8\_TCELL\_UP |  | 20 | -0.11 | -0.48 | 0.993 | 0.993 | 1.000 | 319 | tags=30%, list=23%, signal=38% |
| 2368 | GSE41176\_UNSTIM\_VS\_ANTI\_IGM\_STIM\_TAK1\_KO\_BCELL\_6H\_UP |  | 24 | -0.10 | -0.48 | 0.980 | 0.994 | 1.000 | 1175 | tags=96%, list=83%, signal=563% |
| 2369 | GSE12845\_IGD\_NEG\_BLOOD\_VS\_PRE\_GC\_TONSIL\_BCELL\_UP |  | 18 | -0.11 | -0.48 | 0.986 | 0.994 | 1.000 | 18 | tags=6%, list=1%, signal=6% |
| 2370 | GSE23505\_UNTREATED\_VS\_4DAY\_IL6\_IL1\_IL23\_TREATED\_CD4\_TCELL\_DN |  | 23 | -0.10 | -0.48 | 0.995 | 0.994 | 1.000 | 1274 | tags=100%, list=90%, signal=1013% |
| 2371 | GSE41867\_NAIVE\_VS\_DAY8\_LCMV\_EFFECTOR\_CD8\_TCELL\_UP |  | 26 | -0.09 | -0.48 | 0.982 | 0.994 | 1.000 | 931 | tags=85%, list=66%, signal=244% |
| 2372 | GSE11961\_FOLLICULAR\_BCELL\_VS\_MEMORY\_BCELL\_DAY7\_UP |  | 19 | -0.10 | -0.48 | 0.998 | 0.993 | 1.000 | 220 | tags=21%, list=16%, signal=25% |
| 2373 | GSE14769\_UNSTIM\_VS\_20MIN\_LPS\_BMDM\_DN |  | 22 | -0.10 | -0.47 | 0.998 | 0.993 | 1.000 | 1154 | tags=95%, list=82%, signal=516% |
| 2374 | GSE17721\_LPS\_VS\_CPG\_4H\_BMDC\_DN |  | 23 | -0.10 | -0.47 | 0.990 | 0.993 | 1.000 | 556 | tags=52%, list=39%, signal=85% |
| 2375 | GSE11864\_CSF1\_VS\_CSF1\_IFNG\_PAM3CYS\_IN\_MAC\_DN |  | 24 | -0.09 | -0.46 | 0.993 | 0.995 | 1.000 | 131 | tags=13%, list=9%, signal=14% |
| 2376 | GSE11961\_FOLLICULAR\_BCELL\_VS\_GERMINAL\_CENTER\_BCELL\_DAY7\_DN |  | 16 | -0.10 | -0.44 | 0.993 | 0.997 | 1.000 | 25 | tags=6%, list=2%, signal=6% |
| 2377 | GSE21670\_IL6\_VS\_TGFB\_AND\_IL6\_TREATED\_CD4\_TCELL\_UP |  | 19 | -0.09 | -0.43 | 1.000 | 0.997 | 1.000 | 459 | tags=42%, list=33%, signal=62% |
| 2378 | GSE19888\_ADENOSINE\_A3R\_INH\_PRETREAT\_AND\_ACT\_BY\_A3R\_VS\_TCELL\_MEMBRANES\_ACT\_MAST\_CELL\_UP |  | 22 | -0.09 | -0.43 | 0.995 | 0.998 | 1.000 | 1288 | tags=100%, list=91%, signal=1129% |
| 2379 | GSE40685\_NAIVE\_CD4\_TCELL\_VS\_TREG\_DN |  | 19 | -0.10 | -0.43 | 1.000 | 0.997 | 1.000 | 1154 | tags=95%, list=82%, signal=513% |
Table: Gene sets enriched in phenotype **NCAPD3\_MUT (3 samples) **[plain text format]****
